# Dysregulation of ribosome-associated quality control elicits cognitive disorders via overaccumulation of TTC3

**DOI:** 10.1073/pnas.2211522120

**Published:** 2023-03-14

**Authors:** Ryo Endo, Yi-Kai Chen, John Burke, Noriko Takashima, Nayan Suryawanshi, Kelvin K. Hui, Tatsuhiko Miyazaki, Motomasa Tanaka

**Affiliations:** ^a^Laboratory for Protein Conformation Diseases, RIKEN Center for Brain Science, Wako, Saitama 351-0198, Japan; ^b^Department of Pathology, Gifu University Hospital, Gifu 501-1194, Japan

**Keywords:** ribosome-associated quality control, TTC3, UFMylation, ribosome stalling, cognitive disorders

## Abstract

Ribosome-associated quality control (RQC) pathway is responsible for degradation of nascent polypeptides in aberrantly stalled ribosomes, and its defects may lead to neurological diseases. However, the underlying molecular mechanism of how RQC dysfunction elicits neurological disorders remains poorly understood. Here we revealed that neurons with knockout (KO) of ubiquitin ligase LTN1, a key gene in the RQC pathway, show developmental defects in neurons via upregulation of TTC3 and UFMylation signaling proteins. The abnormally enhanced TTC3 protein in *Ltn1* KO neurons reduced further accumulation of translationally arrested products by preventing translation initiation of selective genes. However, the overaccumulated TTC3 protein in turn caused dendritic abnormalities and reduced surface-localized GABA_A_ receptors during neuronal development. *Ltn1* KO mice showed behavioral deficits associated with cognitive disorders, a subset of which were restored by TTC3 knockdown in medial prefrontal cortex. Together, the overactivated cellular compensatory mechanism against defective RQC through TTC3 overaccumulation induced synaptic and cognitive deficits. More broadly, these findings represent a novel cellular mechanism underlying neuronal dysfunctions triggered by exaggerated cellular stress response to accumulated abnormal translation products in neurons.

## Significance

Ribosome quality control (RQC) is a co-translational surveillance mechanism for degradation of nascent polypeptides in aberrantly stalled ribosomes during translation. Although dysfunction of RQC is suggested to elicit neurological disorders, molecular mechanisms therein have remained poorly understood. Here, we revealed that the loss of LTN1, an E3 ubiquitin ligase in the RQC pathway, significantly increases TTC3 and UFMylation signaling proteins in neurons. The abnormally accumulated TTC3 was stabilized by UFMylation signaling and prevented further accumulation of translationally arrested products by inhibiting translation initiation of selective genes. Instead, the aberrantly increased TTC3 protein caused dendritic and synaptic abnormalities associated with cognitive disorders. Thus, our data provide novel evidence of a possible molecular link between neuronal RQC dysfunction and cognitive disorders.

All organisms have evolved mechanisms to selectively survey and eliminate misfolded or misprocessed proteins by degradation (1–3). The ribosome-associated quality control (RQC) pathway is a co-translational quality control system that recognizes aberrantly stalled ribosomes and degrades nascent polypeptides remaining on 60S subunits (4–6). In the RQC pathway, stalled ribosomes on aberrant mRNAs are first sensed by ubiquitin ligase ZNF598, which binds to 40S ribosome subunit and ubiquitinates ribosomal proteins Rps10 and Rps20, leading to splitting of stalled 80S ribosome into 40S and 60S subunits (7, 8). Then, nuclear export mediator factor (NEMF) binds to the 60S subunit and adds C-terminal alanine and threonine residues (CAT-tails) to nascent polypeptides (9, 10). NEMF also recruits ubiquitin ligase LTN1, which polyubiquitinates the nascent polypeptides with CAT-tails for subsequent proteasomal degradation (11–13). A failure of RQC machinery due to genetic removal of a RQC factor such as LTN1 or NEMF is known to cause proteotoxic stress in cells (14, 15). Furthermore, recent studies show that NEMF variants are associated with neurological disorders including intellectual disability (16, 17), suggesting a link between dysfunction of neuronal RQC and human neurological diseases. However, our understanding of how impaired RQC function elicits neurological disorders and causative molecular mechanisms therein is highly limited. In addition to the RQC, a cellular pathway involving UFM1, which is a ubiquitin-like posttranslational modification and covalently attached to a lysine residue of substrates, also regulates co-translational quality control of nascent polypeptides in aberrantly stalled ribosomes (18, 19). As known for ubiquitination pathway, UFMylation involves E1-like UBA5, E2-like UFC1, and E3-like UFL1 (20, 21). UFL1 forms a complex with DDRGK and CDK5RAP3, both of which are the key components that mediate UFMylation of substrate proteins in the ER (20, 21). Importantly, missense mutations in UFMylation-related genes have been linked to human neuropathologies including defective brain development (22, 23). However, pathological roles of UFMylation in neurons remain poorly understood.

In this study, we established LTN1-deficient mice (*SI Appendix*, Fig. S1*A*) and performed multi-dimensional analyses at the molecular, cellular, and behavioral levels to uncover physiological consequences of neuronal RQC dysfunction. We report here that TTC3 and UFMylation signaling proteins are substantially accumulated in *Ltn1* KO neurons, which reduces further overload of translationally arrested products by inhibiting translation initiation. Instead, however, we found that the aberrantly accumulated TTC3 protein elicits defects of neurite outgrowth and underlie cognitive disorders in *Ltn1* KO mice.

## Results

### Increased TTC3 and UFMylation Signaling Proteins in *Ltn1* KO Neurons.

**Fig. 1.**
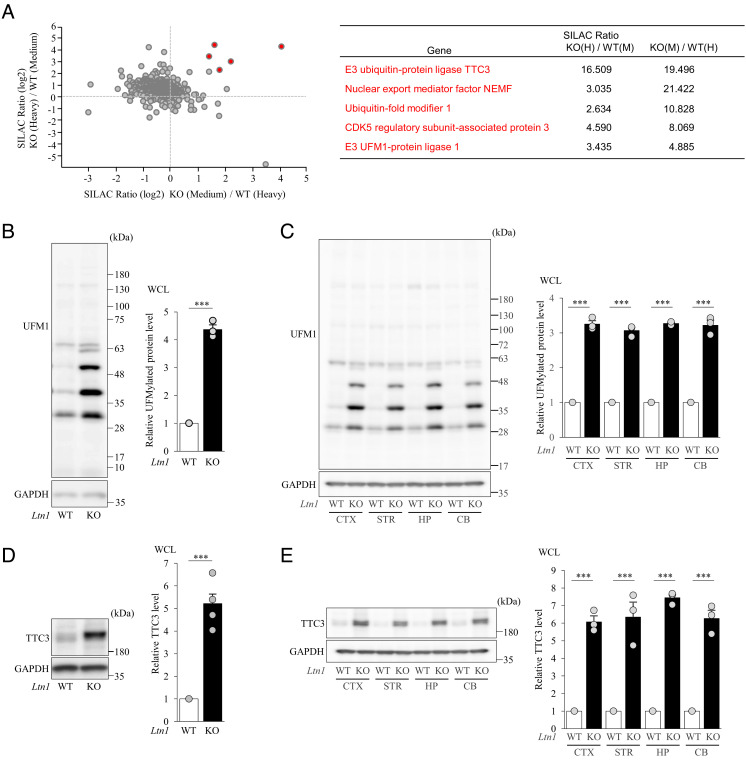
Quantitative proteomics approach using isolated 60S ribosome subunit fraction identified alterations of TTC3 and UFMylation signaling protein levels in LTN1-deficient neurons. (*A*) Quantitative proteomics data from isolated 60S ribosome subunit fraction (*Left*) and the list of candidate proteins with their SILAC scores (*Right*). (*B*) UFM1-conjugated proteins in neuronal lysates were detected by western blotting with an anti-UFM1 antibody. The signal intensities of UFMylated proteins were normalized to those of glyceraldehyde 3-phosphate dehydrogenase (GAPDH) and then expressed as the relative ratio of WT neurons (*Right*) (*n* = 3, ***P *= *0.000035, unpaired two-tailed Student’s *t* test). (*C*) UFMylated proteins in brain lysates from indicated regions were detected by western blotting. The signal intensities of UFMylated proteins were normalized to those of GAPDH and then expressed as the relative ratio of WT (*Right*). (*n* = 3, CTX: ***P *= *0.000002; STR; ***P *= *0.000002; HP: ***P *= *0.0000001; CB: ***P = 0.000098, unpaired two-tailed Student’s *t* test). (*D*) Protein level of TTC3 in neuronal lysates was detected by western blotting with an anti-TTC3 antibody. The signal intensities of TTC3 protein were normalized to those of GAPDH and then expressed as the relative ratio of WT neurons (*Right*) (*n *= 5, ***P = 0.000008, unpaired two-tailed Student’s *t* test). (*E*) Protein levels of TTC3 in indicated brain regions were detected by western blotting with an anti-TTC3 antibody, and the signal intensities of TTC3 protein were normalized to those of GAPDH and then expressed as the relative ratio of WT (*Right*) (*n* = 3, CTX: ***P *= *0.00012; STR; ***P *= *0.0032; HP: ***P *= *0.00001; CB: ***P = 0.00033, unpaired two-tailed Student’s *t* test). CTX: cortex, STR: striatum, HP: hippocampus, CB: cerebellum. Throughout the figures, data represent means ± SEM.

To reveal molecular alterations in *Ltn1* knockout (KO) neurons, we first performed an unbiased quantitative proteomic screen by combining sucrose density gradient centrifugation and stable isotope labeling by amino acids in cell culture (SILAC). Fractions of 60S ribosomal subunits were isolated from SILAC-labeled *Ltn1* wild-type (WT) and KO neurons and analyzed by LC-MS/MS (Fig. 1*A*). We specifically searched for proteins whose abundance was increased by the loss of LTN1 and identified several proteins that fit our criteria (SILAC ratio > 1.5) (Fig. 1*A*). Among these proteins, we were interested in tetratricopeptide repeat domain 3 (TTC3), ubiquitin-fold modifier 1 (UFM1), CDK5 regulatory subunit-associated protein 3 (CDK5RAP3), and E3 UFM1-protein ligase 1 (UFL1), because these proteins reproducibly showed large SILAC ratios and have not yet been investigated in the RQC pathway (Fig. 1*A*). Interestingly, UFM1, UFL1, and CDK5RAP3 are all key components of the UFMylation pathway which is an ubiquitin-like posttranslational protein modification (20, 21), while TTC3 is an E3 ubiquitin ligase highly expressed in the CNS and is closely involved in Down syndrome (24, 25). To corroborate these results, we investigated the levels of TTC3 and UFMylated proteins in both total and 60S fractions of WT and *Ltn1* KO cultured neurons and brains. TTC3 and UFMylated protein levels were significantly increased in both LTN1-deficient cultured neurons and mouse brain in various regions (Fig. 1 *B*–*E* and *SI Appendix*, Fig. S1*B*). Next we purified each 40S and 60S subunit by thoroughly eliminating ribosome-free proteins with ultracentrifugation. The protein levels of UFL1, CDK5RAP3, and DDRGK1 were substantially increased in isolated 60S fractions of *Ltn1* KO neurons (*SI Appendix*, Fig. S1*C*), while TTC3 was mainly detected in the 40S subunit (*SI Appendix*, Fig. S1*D*). Next, we performed quantitative PCR analysis to examine whether mRNA level of TTC3 is also abnormally increased in *Ltn1* KO neurons and found that mRNA level was not altered by LTN1 deletion (*SI Appendix*, Fig. S1*E*). Finally, we performed immunohistological analysis with frozen mouse brain sections and found that both UFM1 and TTC3 protein levels were substantially increased in cerebral cortices of *Ltn1* KO mice (*SI Appendix*, Fig. S1 *F* and *G*). These results were consistent with our quantitative MS analysis using cultured neurons.

### UFMylated RPL26 is increased in 60S Subunit of *Ltn1* KO Neurons.

We found by sucrose density gradient analysis that UFMylated proteins were mainly detected in 60S and, to a lesser degree, 80S and polyribosome fractions (*SI Appendix*, Fig. S2*A*). The ribosomal protein RPL26 was previously reported to be as a major substrate for UFMylation in non-neuronal cells (26). In *Ltn1* KO neurons, an anti-RPL26 antibody detected three clear bands at ~30, ~40, and ~50 kDa corresponding to non-UFMylated, 1× and 2× UFM1-conjugated RPL26, respectively (*SI Appendix*, Fig. S2*B*). Furthermore, immunoprecipitation of RPL26 from neuronal cell lysates, followed by immunoblotting with an anti-UFM1 antibody, demonstrated that RPL26 is UFMylated and its level was significantly increased in *Ltn1* KO neurons (*SI Appendix*, Fig. S2*C*).

Next, we examined how LTN1 deficiency contributed to the observed increase of UFMylated RPL26. When we expressed functional, WT LTN1 in *Ltn1* KO neurons, the level of UFMylated RPL26 was significantly decreased, demonstrating that the increase in UFMylated RPL26 is indeed LTN1-dependent (*SI Appendix*, Fig. S3). Unexpectedly, however, the expression of LTN1 ΔRing, which lacks a catalytic ring finger domain, also reduced the level of UFMylated RPL26 in *Ltn1* KO neurons. Next, to examine the effects of LTN1’s localization on 60S subunit, we employed the ΔN-DD LTN1 mutant which cannot bind to 60S subunit (8). The expression of ΔN-DD LTN1 failed to reduce UFMylated RPL26 in *Ltn1* KO neurons (*SI Appendix*, Fig. S3). These results indicate that the abnormally increased UFMylation of RPL26 in *Ltn1* KO neurons was not caused by the lack of E3 ligase activity of LTN1, but by its absence on the 60S subunit.

### TTC3 Protein is accumulated in 40S Subunit of *Ltn1* KO Neurons.

We investigated the mechanism that increases the protein level of TTC3 in *Ltn1* KO neurons. First, we found that an N-terminally truncated isoform designated as “TTC3 M402” with an initiation codon of Met 402, but not the full length (1979 amino acids) of TTC3, is predominantly expressed in neurons, and its abundance was substantially increased by LTN1 deletion (*SI Appendix*, Fig. S4*A*). Importantly, as we observed for UFMylated RPL26, the abnormal increase in TTC3 level in *Ltn1* KO neurons was also caused by the loss of LTN1’s localization on 60S subunit, but not by the lack of its E3 ligase activity (*SI Appendix*, Fig. S3).

**Fig. 2.**
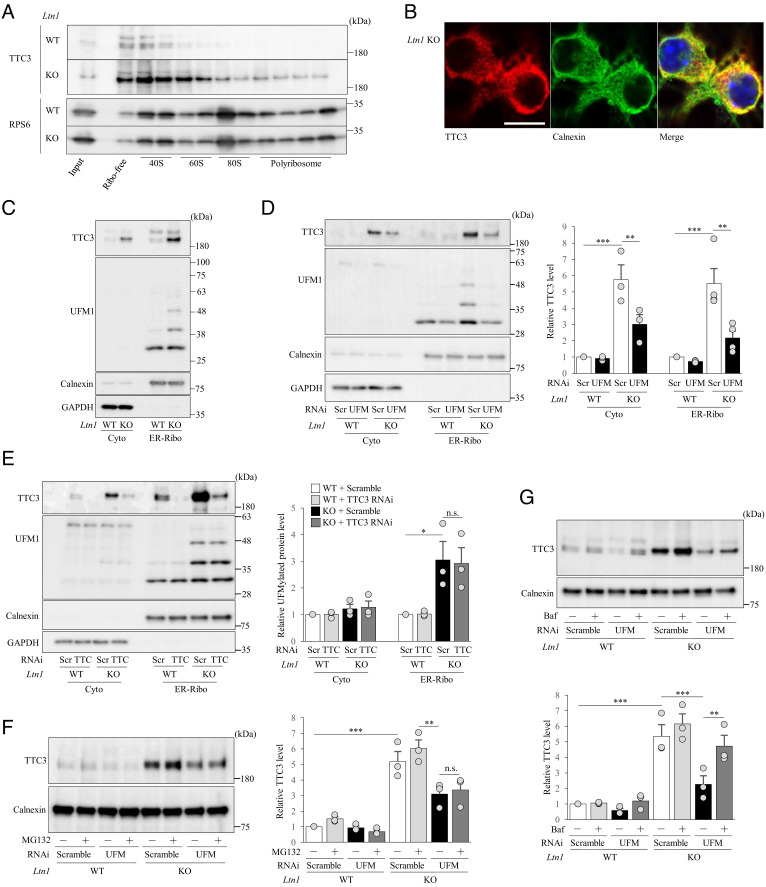
TTC3 is localized in 40S subunits and partially stabilized by UFMylation signal pathway. (*A*) Ribosome-free (Free), 40S, 60S, 80S monosome and polysome fractions were isolated from cultured cortical neurons by sucrose gradient centrifugation, and TTC3 and RPS6 (control) proteins were detected by western blotting. (*B*) Endogenous TTC3 was stained with an anti-TTC3 antibody (red), and ER was visualized with an anti-calnexin antibody (green). (Scale bar, 25 μm.) (*C*) Cytosolic (Cyto) and ER-associated ribosome-enriched (ER-Ribo) fractions were isolated from cultured cortical neurons by the centrifugation method and subjected to western blotting with an anti-TTC3 antibody. Calnexin was detected as an ER-bound ribosome-enriched associated membrane (ER-Ribo) fraction marker, and GAPDH was used as a cytosolic marker. (*D*) Cultured cortical neurons were infected with lentivirus encoding scramble RNAi or UFM1 RNAi, and cytosolic (Cyto) and ER-bound ribosome-enriched associated membrane (ER-Ribo) fractions were isolated. The signal intensities of TTC3 protein were normalized to those of GAPDH for cytosolic fraction or to calnexin for ER-associated ribosome-enriched fraction and then expressed as the relative ratio of WT neurons infected with scramble RNAi (*Right*) [Cyto: *n* = 3, *F*(3, 6) = 43.7, *P *= 0.0017; ER-Ribo: *n *= 4, *F*(3, 9) = 21.9, *P *= 0,0002, one-way ANOVA; **P < 0.01, ***P < 0.001, Bonferroni’s multiple comparison test post hoc]. (*E*) Cultured cortical neurons were infected with lentivirus encoding scramble RNAi or TTC3 RNAi, and cytosolic (Cyto) and ER-bound ribosome-enriched associated membrane (ER-Ribo) fractions were isolated. TTC3 and UFMylated proteins were detected by western blotting. The signal intensities of TTC3 protein were normalized to those of GAPDH for cytosolic fraction or calnexin for ER-Ribofraction and then expressed as the relative ratio of WT neurons (*Right*). [*n* = 3, Cyto: *F*(3, 6) = 1.59, *P* = 0.2877; ER-Ribo: *F*(3, 6) = 10.1, *P *= 0.0093, one-way ANOVA; *P < 0.05, Bonferroni’s multiple comparison test post hoc]. (*F*) Cultured cortical neurons with UFM1 KD were treated with or without 20 μM MG132 for 6 h, and ER-Ribo fraction was isolated. TTC3 was detected by western blotting. The signal intensities of TTC3 protein were normalized to those of calnexin and then expressed as the relative ratio of WT neurons infected with scramble RNAi and without MG132 treatment (*Right*). [*n* = 3, *F*(7, 14) = 41.7, *P *< 0.0001, one-way ANOVA; **P < 0.01, ***P < 0.001, Bonferroni’s multiple comparison test post hoc]. (*G*) Cultured cortical neurons infected with indicated lentivirus were treated with or without 200 nM bafilomycin A (Baf) for 6 h, and ER-Ribo fraction was isolated. The signal intensities of TTC3 protein were normalized to those of calnexin and then expressed as the relative ratio of WT neurons infected with scramble RNAi, without Baf treatment (*Right*) [*n *= 3, *F*(7, 14) = 43.7, *P *< 0.0001; one-way ANOVA; **P < 0.01, ***P < 0.001, Bonferroni’s multiple comparison test post hoc]. n.s., not significant. Throughout the figures, data represent means ± SEM.

Next, when we assessed the precise localization of TTC3 among 40S, 60S, monosomes, and polysomes in cultured neurons, TTC3 was predominantly detected in 40S subunit (Fig. 2*A* and *SI Appendix*, Fig. S1*D*). Since cells contain cytosolic and ER-associated ribosomes, we investigated whether TTC3 preferentially binds to either or both of the ribosomes in neurons. Previous studies with mammalian dividing cells showed that TTC3 is detected mainly in the nucleus (27, 28). In neurons, however, we found that TTC3 is localized to the cytoplasm, including neurites (Fig. 2*B* and *SI Appendix*, Fig. S1*B*), and our double-immunostaining analysis showed that TTC3 showed a mesh-like distribution and frequently co-localized with an ER marker, calnexin (Fig. 2*B*). This finding was in agreement with the perinuclear localization of TTC3 by the immunohistological analysis of cerebral cortex from *Ltn1* KO mice (*SI Appendix*, Fig. S1*G*). These immunostaining data were confirmed by subcellular fractionation of neuronal lysates for separation between cytosolic and ER-bound ribosome-enriched membrane (ER-Ribo) fractions (29) (Fig. 2*C*). Furthermore, our treatment of the isolated membrane fraction with proteinase K degraded TTC3, but not an ER luminal protein, protein disulfide isomerase, demonstrating that TTC3 is not an ER luminal protein but is associated with the cytosolic face of ER membrane (*SI Appendix*, Fig. S4*B*). Together, these results suggest that TTC3 plays a role in translation regulation in the 40S subunit of ER-associated ribosomes.

Given such a significant increase in both UFMylated RPL26 and TTC3 levels in *Ltn1* KO neurons, we next investigated the hierarchical relationships between UFMylation signaling and TTC3 in neurons. When we immunoprecipitated UFMylated proteins from cytoplasmic and ER-Ribo fractions of neuronal lysates (*SI Appendix*, Fig. S4*C*), TTC3 was detected in ER-Ribo fractions of *Ltn1* KO neurons. This result indicates that TTC3 itself is UFMylated or interacts with other UFMylated, ER-Ribo associated proteins (*SI Appendix*, Fig. S4*C*), although future studies are required to examine it. Importantly, the knockdown (KD) of UFM1 significantly decreased the protein level of TTC3 (Fig. 2*D*), whereas the KD of TTC3 showed no impact on the UFMylated RPL26 level (Fig. 2*E*). Therefore, the UFMylation signaling is located upstream of TTC3 and regulates the abundance of TTC3 protein. In UFM1 KD neurons, while the proteasome inhibitor MG132 had no effect on TTC3 levels (Fig. 2*F*), the lysosomal inhibitor bafilomycin A partially restored TTC3 protein levels (Fig. 2*G*). Together, these results show that UFMylation signaling at least partly stabilizes TTC3 protein and increases its abundance by protecting TTC3 from lysosomal degradation.

### TTC3 Protein Level Is Increased when Translationally Arrested K24 Products were Overloaded.

**Fig. 3.**
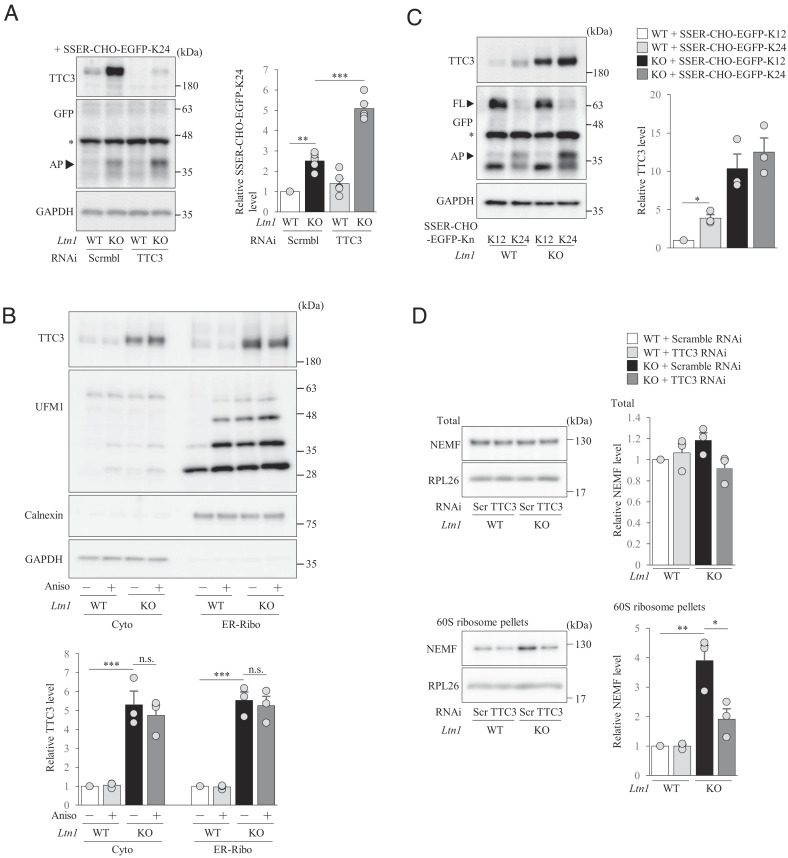
TTC3 protein level is increased by an overload of translationally arrested K24 products. (*A*) Cultured cortical neurons were first infected with lentivirus encoding scramble RNAi or TTC3 RNAi, followed by infection of AAV encoding SSER-CHO-EGFP-K24. Arrested products (AP; indicated by arrowhead) were detected by western blotting using an anti-GFP antibody. The signal intensities of arrested products were normalized to those of GAPDH and then expressed as the relative ratio of WT+Scramble RNAi neurons (*Right*) [*n *= 5, *F*(3, 12) = 73.2, *P* < 0.0001; one-way ANOVA; **P < 0.01, ***P < 0.001, Bonferroni’s multiple comparison test post hoc]. An asterisk indicates non-specific band. (*B*) Cultured cortical neurons were treated with anisomycin for 4 h, and cyotosolic (cyto) and ER-associated ribosome-enriched (ER-Ribo) fractions were separated. The signal intensities of TTC3 protein were normalized to those of GAPDH for cytosolic fraction or calnexin for ER-associated ribosome-enriched fraction and then expressed as the relative ratio of WT neurons without anisomycin treatment (*Bottom*). [*n* = 3, Cyto: *F*(3, 6) = 34, *P* = 0.0004; ER-Ribo: *F*(3, 6) = 87.7, *P *< 0.0001, one-way ANOVA; ***P < 0.001, Bonferroni’s multiple comparison test post hoc]. n.s., not significant. (*C*) Cultured cortical neurons were infected with indicated AAVs and TTC3 were detected by western blotting using anti-TTC3 antibody. Arrested products (AP) and full-length proteins (FL) are indicated by arrowheads. The signal intensities of TTC3 protein were normalized to those of GAPDH and then expressed as the relative ratio of WT neurons infected with control AAVs [*n *= 3, *F*(3, 6) = 30.1, *P *= 0.0005, one-way ANOVA; *P < 0.05, Bonferroni’s multiple comparison test post hoc]. An asterisk indicates non-specific band. (*D*) Total lysates (*Top*) or 60S ribosomal fractions (*Bottom*) were collected from cultured cortical neurons by sucrose gradient centrifugation, and ribosomes were further pelleted down (*Bottom*) and analyzed by western blotting with indicated antibodies. The signal intensities of NEMF were normalized to those of RPL26 and then expressed as the relative ratio of WT neurons infected with scramble RNAi [Total: *n* = 3, *F*(3, 8) = 2.21, *P* = 0.1649; 60S: *n* = 3, *F*(3, 6) = 26.3, *P* = 0.0008, one-way ANOVA; *P < 0.05, **P < 0.01, Bonferroni’s multiple comparison test post hoc]. Throughout the figures, data represent means ± SEM.

Since TTC3 abundance is significantly increased by disrupted RQC and TTC3 is localized in Cyto-Ribo and ER-Ribo, we next investigated whether TTC3 is involved in the RQC pathway by using the reporter constructs that induce translational stalling at either cytosolic (EGFP-K24) ribosomes or ER-associated (SSER-CHO-EGFP-K24) ribosomes that harbors an ER targeting signal sequence (SSER) and an *N*-glycosylation site (CHO) to monitor ER-inserted translationally arrested products (18) (*SI Appendix*, Fig. S5*A*). When either reporter construct was expressed, translationally arrested products were substantially accumulated in *Ltn1* KO neurons compared to WT neurons (*SI Appendix*, Figs. S3*A* and S5*B*), demonstrating that RQC function is disrupted by the loss of LTN1, as expected. Importantly, the KD of TTC3 further enhanced the accumulation of arrested products in *Ltn1* KO neurons, but not in WT neurons (Fig. 3*A* and *SI Appendix*, Fig. S5*B*). Therefore, when additional RQC functions are required due to the overload of arrested products in LTN1-deficient neurons, TTC3 may participate in RQC pathway to reduce the amount of arrested products.

Next, we explored whether the enhanced UFMylated RPL26 in stalled ribosomes is responsible for the abnormal increase in TTC3 levels in *Ltn1* KO neurons. To address it, we treated neurons with anisomycin, which stalls ribosomes and enhances UFMylation of RPL26 (20). We found that the UFMylated RPL26 level was significantly increased (Fig. 3*B*) in WT neurons, as reported in non-neuronal cells (18), but TTC3 protein level was unchanged (Fig. 3*B*). Similar results were obtained in *Ltn1* KO neurons (Fig. 3*B*). Therefore, it is not simply the UFMylated RPL26-associated ribosomal stalling that enhanced the TTC3 protein level.

We then examined whether RQC-associated ribosomal stalling increases TTC3 protein levels by expressing an excess of EGFP-K24 or SSER-CHO-EGFP-K24. Remarkably, compared to the non-stalling K12 control (*SI Appendix*, Fig. S5*A*), the expression of EGFP-K24 or SSER-CHO-EGFP-K24 induced significant accumulation of TTC3 not only in *Ltn1* KO neurons, but also in WT neurons (Fig. 3*C* and *SI Appendix*, Fig. S5*C*), demonstrating that TTC3 protein level is increased selectively in response to the overload of arrested products that need to be eliminated by RQC. These results suggest that TTC3 protein level is increased when the amount of arrested products exceeds the capacity of neurons to degrade by canonical RQC function.

### TTC3 inhibits further Accumulation of Arrested Products by preventing Translation Initiation.

We examined the mechanism by which TTC3 mediates the reduction of arrested products in *Ltn1* KO neurons. By using the K24 reporter construct, we found by immunoprecipitation experiments that TTC3 does not interact with nascent polypeptides of the K24 arrested products in stalled ribosomes (*SI Appendix*, Fig. S5*D*). This result indicates that TTC3 does not ubiquitinate nascent polypeptides in 60S subunit as an E3 ligase like LTN1 and is consistent with the observation that TTC3 is mainly localized to 40S (Fig. 2*A* and *SI Appendix*, Fig. S1*D*).

Next, we examined the possibility that TTC3 indirectly degrades arrested products in stalled ribosomes. Our proteomic analysis indicated that the NEMF protein level was enhanced in *Ltn1* KO neurons (Fig. 1*A*), suggesting an increase in NEMF-mediated CATylation of nascent polypeptides. This situation may promote LTN1-independent ubiquitination of nascent polypeptides for proteasomal degradation, as reported previously (30). To examine this possibility, we first investigated whether the binding of TTC3 to 40S subunits might enhance NEMF’s binding to 60S subunit and thereby facilitate NEMF-mediated, LTN1-independent degradation of arrested products (30). As was shown by our initial proteomic analysis, the abundance of NEMF in 60S subunit was increased in *Ltn1* KO neurons, although the total expression level of NEMF did not change by LTN1 deletion (Fig. 3*D*). Interestingly, TTC3 KD significantly reduced the binding of NEMF to 60S subunit in *Ltn1* KO neurons (Fig. 3*D*). These results suggest that in *Ltn1* KO neurons the accumulated TTC3 caused the increased binding of NEMF to 60S subunit, implying that TTC3 indirectly enhances NEMF-mediated ubiquitination of translationally arrested products for proteasomal degradation in a LTN1-independent manner (30).

To test this hypothesis, we examined how the proteasome inhibitor, MG132, stabilizes K24 arrested products in WT or *Ltn1* KO neurons with or without TTC3 KD. First, as expected from the notion that K24 arrested products in *Ltn1* KO neurons have already been stabilized by the lack of LTN1’s E3 ligase activity, the MG132 treatment in *Ltn1* KO neurons exhibited smaller stabilization effects on the arrested products than that in WT neurons. However, MG132 treatment partially stabilized arrested products in *Ltn1* KO neurons (*SI Appendix*, Fig. S5*E*), suggesting that arrested products are degraded to some extent even in the absence of LTN1 as indicated previously (30). Importantly, we found that the MG132 treatment in *Ltn1* KO neurons with TTC3 KD did not alter the stabilization effects on K24 arrested products, as compared with that in *Ltn1* KO neurons with scramble RNAi, indicating that although TTC3 promotes the binding of NEMF to 60S subunit, it does not enhance NEMF-mediated, LTN1-independent proteasomal degradation of K24 arrested products (*SI Appendix*, Fig. S5*E*). Together, it is unlikely that the increase in TTC3 abundance in *Ltn1* KO neurons plays a role in boosting cellular RQC function by directly or indirectly enhancing proteasomal degradation of nascent polypeptides in aberrantly stalled ribosomes.

**Fig. 4.**
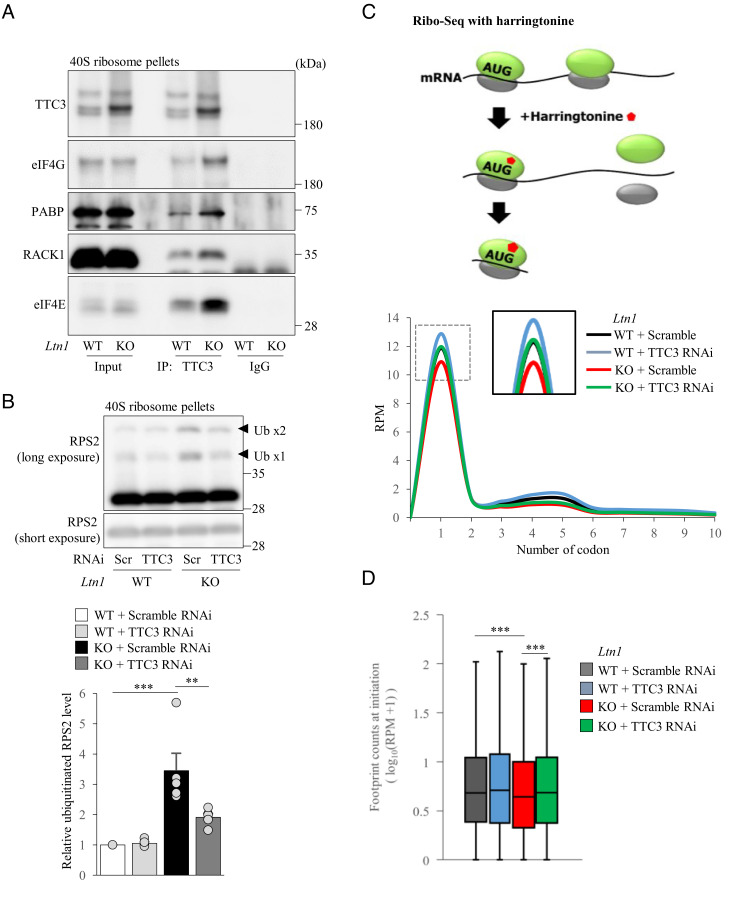
TTC3 reduces an overload of translationally arrested products by preventing translation initiation. (*A*) TTC3 was immunoprecipitated from pelleted 40S ribosome subunits, followed by western blotting using indicated antibodies. (*B*) 40S ribosome subunits pelleted from cultured cortical neurons were analyzed by western blotting with an anti-RPS2 antibody. The signal intensities of ubiquitinated RPS2 (arrowheads) were normalized to those of non-ubiquitinated RPS2 (*n *= 5, *F*(3, 12) = 16.0, *P *= 0.0002, one-way ANOVA; **P < 0.01, ***P < 0.001, Bonferroni’s multiple comparison test post hoc). (*C* and *D*) Ribosome profiling data of harringtonine-treated WT or *Ltn1* KO neurons. (*C*) Metagene analysis of footprint counts in the first ten codons in WT or *Ltn1* KO neurons with scramble or TTC3 RNAi. (*D*) Normalized footprint counts at the translation initiation (ATG) site (*P *= 2.571 × 10^−8^, Kruskal–Wallis test; ***P < 0.001, Bonferroni-corrected Wilcoxon–Mann–Whitney’s multiple comparison test post hoc). Throughout the figures, data represent means ± SEM.

Since TTC3 mainly localizes in the 40S subunit, we then hypothesized that the overaccumulated TTC3 might reduce the amount of arrested products by acting on small ribosomal subunits and inhibiting translation initiation. First, we examined phosphorylation of eIF2α in *Ltn1* KO neurons, as it is widely known to inhibit translation initiation (31). We found that phosphorylation of eIF2α was not altered in *Ltn1* KO neurons (*SI Appendix*, Fig. S6*A*), suggesting that TTC3 does not inhibit translation initiation simply by enhancing phosphorylation of eIF2α in the eIF2 ternary complex. Next, we performed co-immunoprecipitation experiments with isolated 40S fractions to examine whether TTC3 interacts with translation initiation factors on small ribosomal subunits. Interestingly, we found that TTC3 interacts with several translation initiation factors such as eIF4E, eIF4G, PABP, and RACK1 (Fig. 4*A*). Moreover, we found that ubiquitination of RPS2, which is triggered by inhibition of translation initiation (32, 33), was increased in 40S subunits of *Ltn1* KO neurons (Fig. 4*B*). Importantly, TTC3 KD significantly reduced ubiquitination of RPS2 in 40S subunit (Fig. 4*B*), suggesting that translation initiation in *Ltn1* KO neurons is impaired at least in part due to the increased TTC3 abundance. However, when we performed the experiments of BioOrthogonal Non-Canonical Amino acid Tagging (BONCAT) to examine whether total translation levels are altered in *Ltn1* KO neurons, they were not largely different between WT and *Ltn1* KO neurons (*SI Appendix*, Fig. S6*B*). Therefore, the defects of translation initiation by the loss of LTN1 may be selective for specific genes.

To further test our hypothesis that overaccumulated TTC3 inhibits translation initiation in *Ltn1* KO neurons, we employed a ribosome profiling (Ribo-seq) technique, which is based on deep sequencing of ribosome-protected mRNA fragments and allows us to investigate translation at single nucleotide resolution, for the neurons treated with harringtonine, an inhibitor of translation initiation (34). Since harringtonine induces accumulation of ribosomes at translation initiation sites (34), Ribo-seq with harringtonine is likely to provide detailed information on translation initiation in *Ltn1* KO neurons. Ribo-seq data revealed that *Ltn1* KO neurons showed a decrease in ribosome-protected mRNAs (footprints) at translation initiation sites suggesting that translation initiation is compromised by the loss of LTN1. When we sorted the genes by the difference in footprint number at translation initiation site between WT and *Ltn1* KO neurons, we found that a subset (46.3%), but not all, of genes showed reduced translation initiation in *Ltn1* KO neurons (*SI Appendix*, Fig. S6*C* and Table S1), indicating that translation initiation of selective mRNAs was affected by the deletion of LTN1. Furthermore, the Gene Ontology (GO) term analysis using the top 5% differential genes revealed that the reduction of translation initiation in *Ltn1* KO neurons was enriched for the genes associated with various metabolic processes required for maintenance of cellular homeostasis (*SI Appendix*, Fig. S6*D*). Strikingly, TTC3 KD in *Ltn1* KO neurons restored the ribosome footprint number at translation initiation site, indicating that the increased protein level of TTC3 is responsible for inhibition of translation initiation in *Ltn1* KO neurons (Fig. 4 *C* and *D*). Together, these results support the notion that the enhanced TTC3 protein in *Ltn1* KO neurons reduces further accumulation of arrested products by acting on 40S ribosomal subunits and preventing the process of translation initiation for selective genes.

### Overaccumulated TTC3 Protein induces Dendritic and Synaptic Defects in *Ltn1* KO Neurons.

**Fig. 5.**
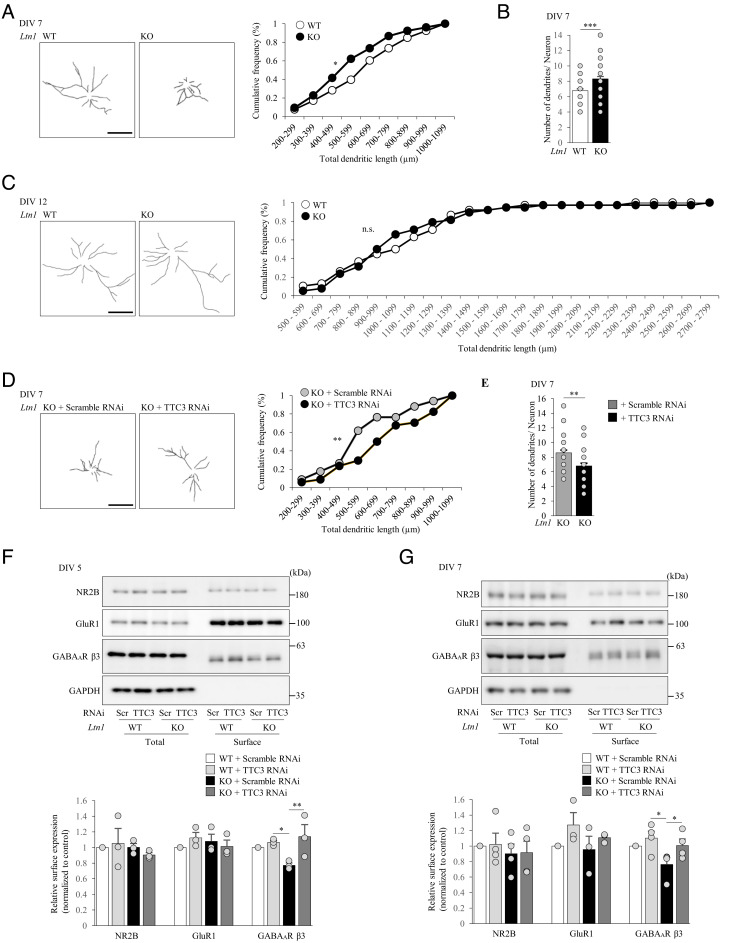
Dendritic outgrowth and synaptic homeostasis are impaired in *Ltn1* KO neurons partially due to the overaccumulation of TTC3 protein. (*A*) Dendrites of cultured cortical neurons at DIV 7 were stained with an anti-MAP2 antibody. *Left* panels show representative tracings of dendritic morphology. Total dendritic length was measured (*Right*) (*n *= 34, *P = 0.029, Kolmogorov–Smirnov test). (*B*) Cultured cortical neurons at DIV 7 were stained with an anti-MAP2 antibody, and the number of dendrites per neuron was counted (*n *= 34 **P = 0.001174, unpaired two-tailed Student’s *t* test). (*C*) Total dendritic length of neurons at DIV 12 was measured as in (*A*) (*n *= 38, *P *= 0.7306, Kolmogorov–Smirnov test). (*D* and *E*) Cultured cortical *Ltn1* KO neurons infected with indicated lentivirus were stained with an anti-MAP2 antibody at DIV 7. (*D*) Representative tracing of MAP2 positive dendrites is shown (*Left*). Total dendritic length was measured (*Right*) (*n *= 30, **P = 0.0062, Kolmogorov–Smirnov test). (*E*) The number of dendrites per neurons was counted (*n *= 30, *P = 0.0146, unpaired two-tailed Student’s *t* test). (*F*) Surface-localized proteins in cultured cortical neurons at DIV 5 were biotinylated, and the surface-biotinylated proteins were pulled down from 50 μg of total neuronal lysates by streptavidin-conjugated beads, followed by western blotting with indicated antibodies (*Left*). GAPDH was used as negative controls. The relative amounts of surface-localized proteins are shown (*Right*) (*n* = 3, NR2B: *F*(3, 6) = 0.535, *P* = 0.6749; GluR1: *F*(3, 6) = 1.75, *P *= 0.2495; GABA_A_β3: *F*(3, 6) = 5.95, *P* = 0.0313, one-way ANOVA; *P < 0.05, **P < 0.01, Bonferroni’s multiple comparison test post hoc). (*G*) Surface-localized proteins in cultured cortical neurons at DIV 7 were analyzed as in (*F*) (NR2B: *n *= 4, *F*(3, 9) = 0.575, *P *= 0.6458; GluR1: *F*(3, 8) = 1.40, *P *= 0.6458; GABA_A_β3: *F*(3, 9) = 4.27, *P *= 0.0391, one-way ANOVA; *P < 0.05, Bonferroni’s multiple comparison test post hoc). n.s., not significant. (Scale bars, 100 μm.) Throughout the figures, data represent means ± SEM.

A previous study suggested that LTN1 KD in neurons reduce neuritic outgrowth, albeit through unknown mechanisms (35). To gain insights into the effects of impaired RQC in neuronal morphology, we first investigated whether LTN1 deletion in neurons affects dendritic outgrowth. On both 4 and 7 days in vitro (DIV), total dendritic length in *Ltn1* KO was significantly shorter that in WT neurons (Fig. 5*A* and *SI Appendix*, Fig. S7*A*). Furthermore, the number of dendrites was increased in *Ltn1* KO neurons on 7 DIV (Fig. 5*B* and *SI Appendix*, Fig. S7*B*). In contrast, such alterations in *Ltn1* KO were not observed in mature DIV12 neurons (Fig. 5*C* and *SI Appendix*, Fig. S7*C*). These results suggest that RQC dysfunction by LTN1 deletion caused developmental abnormalities in neurons. Since previous studies implicated TTC3 in neurite extension (28, 36), we next examined the involvement of overaccumulated TTC3 protein in the reduced dendritic outgrowth observed in *Ltn1* KO neurons. Strikingly, the KD of TTC3 normalized both the dendritic outgrowth and the number of dendrites in *Ltn1* KO neurons (Fig. 5 *D* and *E*). Therefore, the increased TTC3 protein is responsible for neuronal developmental defects caused by the RQC deficiency.

Since the dendritic arbor is responsible for receiving and consolidating neuronal inputs, we next examined the impact of RQC deficiency on synaptic homeostasis. We investigated whether surface expression levels of neurotransmitter receptors, which are critical for synaptic functions, are affected in *Ltn1* KO neurons. On DIV 5 and 7, while no significant differences were observed for surface expression levels of AMPA and NMDA receptors, surface GABAAβ3 receptor levels were selectively decreased in *Ltn1* KO neurons without affecting total protein expression levels (Fig. 5 *F* and *G*). Importantly, the KD of TTC3 normalized the reduction of surface-localized GABAAβ3 receptor levels in *Ltn1* KO neurons (Fig. 5 *F* and *G*). These results show that TTC3 is crucial for maintaining the surface expression of GABAAβ3 receptors, suggesting that the excess protein level of TTC3 due to the LTN1 deficiency may disrupt synaptic functions in neurons.

### *Ltn1* KO Mice exhibit Cognitive Disorders via TTC3 Overaccumulation.

**Fig. 6.**
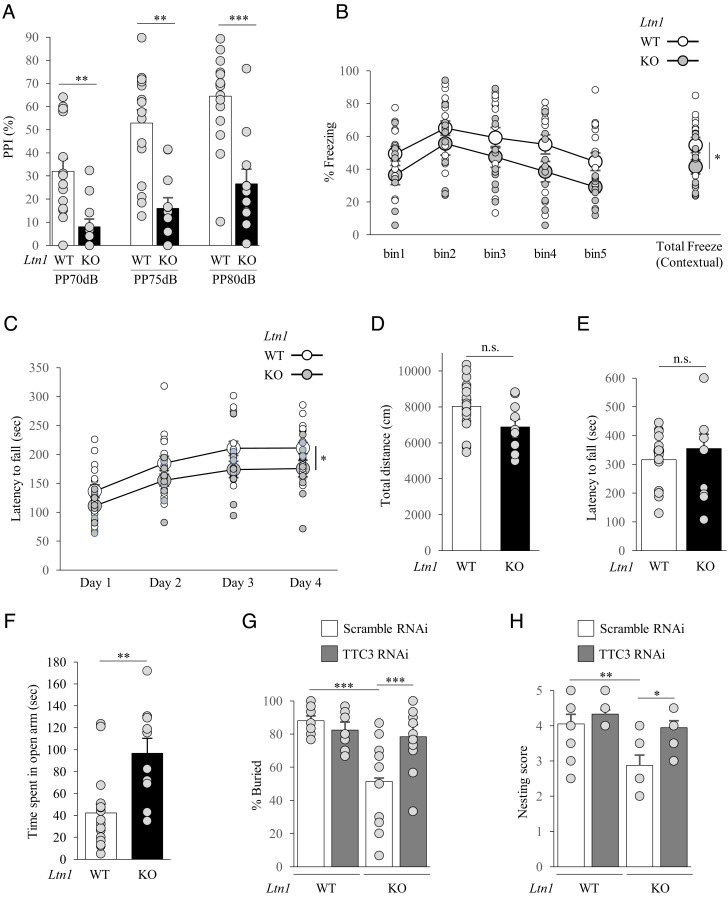
*Ltn1* KO mice show behavioral deficits associated with cognitive disorders partially though overaccumulation of TTC3 protein. (*A*) In PPI test, *Ltn1* KO mice showed a reduced PPI response. PPI values at three different prepulse intensities (70 dB, 75 dB and 80 dB) are shown. *n* = 17 (WT), *n* = 11 (*Ltn1* KO) (70 dB: *P* = 0.003; 75 dB: *P *= 0.001; 80 dB: *P *= 0.0006; two-tailed Mann–Whitney test). (*B*) In contextual fear conditioning test, *Ltn1* KO mice spent less time freezing than WT mice. Percent time of freezing during 5 min of the context test is shown. *n *= 17 (WT), *n *= 11 (*Ltn1* KO) (*P *= 0.0455; two-tailed Mann–Whitney test). (*C*) The latency to fall off the rotarod was recorded and *Ltn1* KO mice showed impaired motor function. *n *= 17 (WT), *n *= 11 (*Ltn1* KO) (*P *= 0.033; two-tailed Mann–Whitney test). (*D*) Distance traveled in the open field test. *n *= 14 (WT), *n *= 10 (*Ltn1* KO). No statistical differences were detected. (*P *= 0.8274; two-tailed Mann–Whitney test). (*E*) Measurement of forearm grip strength. Grip strength was comparable between the two mice groups. *n *= 14 (WT), *n *= 11 (*Ltn1* KO). No statistical differences were detected. (*P *= 0.459; two-tailed Mann–Whitney test). (*F*) In EPM test, *Ltn1* KO mice spent longer time in open arm and showed reduced anxiety. *n *= 17 (WT), *n *= 10 (*Ltn1* KO) (*P *= 0.0017; two-tailed Mann–Whitney test). (*G* and *H*) AAV encoding scramble or TTC3 RNAi was stereotaxically injected into mouse mPFC. (*G*) Marble-burying test showed that marble-burying behavior is reduced in *Ltn1* KO mice and exhibited a blunted response to the aversive situation that was normalized by KD of TTC3. *n *= 14, 14, 13, 14 for WT + Scramble RNAi, WT + TTC3 RNAi, KO+Scramble RNAi, and KO + TTC3 RNAi, respectively. [*F*(3,49) = 12.6, *P *< 0.0001, one-way ANOVA; ***P < 0.001, Bonferroni’s multiple comparison test post hoc]. (*H*) Defects in nesting behavior were observed for *Ltn1* KO mice that was rescued by KD of TTC3. *n *= 10, 9, 8, 9 for WT + Scramble RNAi, WT + TTC3 RNAi, KO + Scramble RNAi, and KO + TTC3 RNAi, respectively. [*F*(3, 32) = 7.09, *P *= 0.0009, one-way ANOVA; *P < 0.05, **P < 0.01, Bonferroni’s multiple comparison test post hoc]. n.s., not significant. Throughout the figures, data represent means ± SEM.

*Ltn1* KO neurons showed impairments of neuronal development and synaptic homeostasis. Therefore, we performed a series of behavioral tests for 3-mo-old *Ltn1* KO mice to examine how such neuronal defects may impact behaviors. In the prepulse inhibition (PPI) test, *Ltn1* KO mice showed significantly reduced PPI (Fig. 6*A*), which is a typical phenotype observed in both autism spectrum disorder (ASD) and schizophrenia. In the fear conditioning test, contextual fear memory was significantly affected in LTN1-deficient mice. *Ltn1* KO mice showed decreased levels of freezing (Fig. 6*B*), indicating that they exhibit cognitive dysfunction. In the rotarod test, *Ltn1* KO mice showed a reduction of motor performance (Fig. 6*C*). However, spontaneous locomotor activity in the open field was not affected (Fig. 6*D*), and the wire hang test showed that neuromuscular function was not altered in *Ltn1* KO mice (Fig. 6*E*). These results suggest that the reduced rotarod performance was not caused by impaired motor function itself, but possibly due to a learning impairment in motor coordination. Consistently, our immunohistological analysis did not reveal any obvious neuronal loss in the cerebral cortices of 3-mo-old *Ltn1* KO mice (*SI Appendix*, Fig. S8*A*). We next tested *Ltn1* KO mice for anxiety-like behaviors, first with the elevated plus maze (EPM) test. *Ltn1* KO mice spent more time on the open arms (Fig. 6*F*), suggesting reduced anxiety. With the marble-burying test, *Ltn1* KO mice buried fewer marbles compared to WT mice (Fig. 6*G*), showing a blunted response to the aversive situation. In the nest building test, *Ltn1* KO mice showed impaired nesting behaviors compared to WT mice (Fig. 6*H*), implying a decline in cognition and depressive-like behaviors.

In order to examine causal relationships between accumulated TTC3 protein and behavioral deficits of *Ltn1* KO mice, we performed stereotaxic injections of adeno-associated virus (AAV) encoding TTC3 or scramble shRNA into the medial prefrontal cortex (mPFC), where neural circuits of various higher brain functions are involved. Importantly, the decreased anxiety phenotype manifested by the marble-burying test and the defects in nest building capability in *Ltn1* KO mice were fully restored by the KD of TTC3 (Fig. 6 *G* and *H*). Furthermore, the TTC3 KD appeared to counteract the reduced PPI observed in *Ltn1* KO mice (*SI Appendix*, Fig. S8*B*). Together, these results indicate that *Ltn1* KO mice showed behavioral abnormalities associated with cognitive disorders and a cohort of the behavioral deficits is caused by the aberrantly increased TTC3 protein due to the loss of LTN1.

## Discussion

We revealed that TTC3 and UFMylation signaling proteins are abnormally increased in *Ltn1* KO neurons. The up-regulated TTC3 protein was suggested to prevent further accumulation of translationally arrested products in stalled ribosomes by inhibiting translation initiation, when neurons face an overload of the arrested products that need to be eliminated by RQC. Therefore, our findings indicate that TTC3 serves as a compensatory feedback loop to reduce further accumulation of arrested products, when neuronal RQC function cannot sufficiently cope with the overload of arrested products (*SI Appendix*, Fig. S9). In other words, our data suggest a novel cellular feedback mechanism by which neurons inhibit translation initiation by increasing the abundance of TTC3 protein on 40S subunit. Remarkably, however, we found that abnormally increased TTC3 protein induced various deleterious effects on neuronal functions; the overaccumulated TTC3 protein in *Ltn1* KO neurons and mice results in dendritic and synaptic abnormalities and behavioral deficits associated with cognitive disorders.

Our results provide insights into how RQC dysfunction elicits disorders in the central nervous system. Notably, our findings were unexpected as *Ltn1* KO mice were phenotypically distinct from the previously reported *lister* mice, which were found through a genome-wide ENU mutagenesis screen and exhibited severe signs of neurodegeneration within 3 wk of age (37). Instead, *Ltn1* KO mice showed phenotypes associated with cognitive disorders without showing obvious deficits in locomotor activity, neuromuscular function, and neuronal loss at 4 to 5 mo of age. Although *lister* mice express the mutant *LTN1* with a deletion of 14 amino acid residues, it remains unclear what functions of LTN1 are altered by this deletion. Moreover, since ENU can randomly introduce mutations throughout the genome, one cannot rule out the possibility that mutations in the genes other than *LTN1* are also responsible for the severe neurodegeneration in *lister* mice. Nonetheless, the phenotypic differences might be caused by the downstream molecular pathways, such as those involving TTC3 and UFMylation signaling found in this study, which are potentially distinct between *Ltn1* KO mice and *lister* mice. Interestingly, TTC3 is one of the key genes in Down syndrome critical region on 21q22.2, as multiple Down syndrome model mice with *TTC3* triplication have been shown to commonly exhibit developmental and cognitive dysfunctions (38, 39). Intriguingly, a variant of the deUFMylation enzyme Ufsp2 that enhances UFMylated protein levels was recently identified as a factor contributing to neurodevelopmental disorders in humans (23). In addition, NEMF variants, which were hypothesized to reduce RQC functions, are associated with intellectual disability (16). Together, these results support our observation that *Ltn1* KO mice show behavioral deficits associated with cognitive disorders rather than severe neurodegenerative disorders.

Our results resolved the previous puzzling observations that *Ltn1* KO leads to neurite outgrowth defects and behavioral deficits (35, 37). First, although the previous study indicates that neurite outgrowth defects in LTN1 KD neurons were rescued by additional KD of NEMF (35), the underlying mechanism had remained unclear. Here, we revealed that TTC3 KD in *Ltn1* KO neurons normalized the enhanced binding of NEMF to 60S subunit and restored the dendritic abnormalities. Therefore, our findings suggest that the overaccumulated TTC3-mediated increase in NEMF abundance in 60S subunit is at least partly involved in the neurite outgrowth defects observed in *Ltn1* KO neurons. Next, our data provide possible molecular explanations for the cognitive deficits of *Ltn1* KO mice. The enhanced protein level of TTC3 reduced not only dendritic outgrowth but also surface-localized GABA_A_β3 receptor levels (Fig. 5 *F* and *G*), which would at least partly account for the abnormal behaviors associated with cognitive disorders in *Ltn1* KO mice, as suggested previously (40). Notably, since increased TTC3 protein levels induce fragmentation of the Golgi apparatus (28, 36), the overaccumulated TTC3 by LTN1 deletion might impair Golgi-mediated transport of GABA_A_β3 receptors to the plasma membrane, resulting in reduced surface-localized GABA_A_β3 receptors. Furthermore, the increased UFMylation signaling proteins in *Ltn1* KO may offer mechanistic insights into the selectivity of GABA_A_ receptor. An E1-like enzyme UBA5 in UFMylation pathway interacts with GABA receptor-associated proteins (GABARAPs) (41, 42), which regulate trafficking of GABA_A_ receptors (43). Therefore, dysfunction of GABARAPs leads to reduced expression of surface GABA_A_β3 (44). Likewise, aberrantly enhanced levels of UFMylation factors in *Ltn1* KO neurons might disturb the trafficking function of GABARAPs, resulting in reduction of surface GABA_A_β3 receptor levels (Fig. 5 *F* and *G*). Although further mechanistic studies are required, the present findings suggest a possible molecular link between RQC dysfunction, dendritic and synaptic defects, and behavioral deficits in *Ltn1* KO mice.

Recent studies indicate that dysregulation of various processes in translation, including initiation, elongation, and termination, is associated with a wide range of neurological diseases (45–47). Importantly, our study provides another layer of evidence that not only dysregulation of each translation process itself, but also impaired quality control of nascent polypeptides during translation, could lead to cognitive disorders. Notably, since TTC3 is highly expressed in the central nervous system, it is tempting to speculate that neurons have evolved to maintain highly regulated, balanced mechanisms, such as the TTC3-mediated feedback loop, in order to ensure translation by preventing an overload of translationally arrested products. By contrast, our findings provide a novel concept that overactivation of such a proteostasis maintenance mechanism that responds to ribosome stalling may elicit cognitive disorders. Beyond these mechanistic insights, our study suggests that TTC3 and UFMylation signaling factors may represent novel therapeutic targets for neurological diseases in which arrested translation is involved. More broadly, our findings that developmental defects in neurons are triggered by overaccumulated TTC3 protein provide therapeutic implications for cognitive, neurodevelopmental disorders including Down syndrome.

## Supplementary Material

Appendix 01 (PDF)

Dataset S01 (PDF)

## Data Availability

All study data are included in the article and/or supporting information.

## References

[1] F. U. Hartl, Protein misfolding diseases. Annu. Rev. Biochem. **86**, 21–26 (2017). 28441058 10.1146/annurev-biochem-061516-044518

[2] M. S. Hipp, R. Kasturi, F. U. Hartl, The proteostasis network and its decline in ageing. Nat. Rev. Mol. Cell Biol. **20**, 421–435 (2017). 10.1038/s41580-019-0101-y 30733602

[3] D. Balchin, M. Hayer-Hartl, F. U. Hartl, In vivo aspects of protein folding and quality control. Science **353**, aac4354 (2016). 27365453 10.1126/science.aac4354

[4] O. Brandman , A Ribosome-bound quality control complex triggers degradation of nascent peptides and signals translation stress. Cell **151**, 1042–1054 (2012). 23178123 10.1016/j.cell.2012.10.044 PMC3534965

[5] C. A. P. Joazeiro, Mechanisms and functions of ribosome-associated protein quality control. Nat. Rev. Mol. Cell Biol. **20**, 368–383 (2019). 30940912 10.1038/s41580-019-0118-2 PMC7138134

[6] T. Inada, Quality controls induced by aberrant translation. Nucleic Acids Res. **48**, 1084–1096 (2020). 31950154 10.1093/nar/gkz1201 PMC7026593

[7] E. Sandaramoorthy , ZNF598 and RACK1 regulate mammalian ribosome-associated quality control function by mediating regulatory 40S ribosomal ubiquitylation. Mol. Cell **4**, 751–764 (2016). 10.1016/j.molcel.2016.12.026 PMC5321136 28132843

[8] A. Garzia , The ubiquitin ligase and RNA-binding protein ZNF598 orchestrates ribosome quality control of premature polyadenylated mRNAs. Nat. Commun. **8**, 16056 (2017). 28685749 10.1038/ncomms16056 PMC5504347

[9] K. K. Kostova , CAT-tailing as a fail-safe mechanism for efficient degradation of stalled nascent polypeptides. Science **357**, 414–417 (2017). 28751611 10.1126/science.aam7787 PMC5673106

[10] B. A. Osuna, C. J. Howard, S. Kc, A. Frost, D. E. Weinberg, In vitro analysis of RQC activities provides insights into the mechanism and function of CAT tailing. Elife **6**, e27949 (2017), 10.7554/eLife.27949. 28718767 PMC5562442

[11] M. H. Bengston, C. A. P. Joazeiro, Role of a ribosome-associated E3 ubiquitin ligase in protein quality control. Nature **467**, 470–473 (2010). 20835226 10.1038/nature09371 PMC2988496

[12] S. Shao, A. Brown, B. Santhanam, R. S. Hegde, Structure and assembly pathway of the ribosome quality control complex. Mol. Cell **57**, 433–444 (2015). 25578875 10.1016/j.molcel.2014.12.015 PMC4321881

[13] P. S. Shen , Rqc2p and 60S ribosomal subunits mediate mRNA-independent elongation of nascent chains. Science **347**, 75–78 (2015). 25554787 10.1126/science.1259724 PMC4451101

[14] R. Yonashiro , The Rqc2/Tae2 subunit of the ribosome-associated quality control (RQC) complex marks ribosome-stalled nascent polypeptide chains for aggregation. Elife **5**, e11794 (2016), 10.7554/eLife.11794. 26943317 PMC4805532

[15] Y. J. Choe , Failure of RQC machinery causes protein aggregation and proteotoxic stress. Nature **531**, 191–195 (2016). 26934223 10.1038/nature16973

[16] S. Anazi , Clinical genomics expands the morbid genome of intellectual disability and offers a high diagnostic yield. Mol. Psychiatry **22**, 615–624 (2017). 27431290 10.1038/mp.2016.113

[17] P. B. Martin , NEMF mutations that impair ribosome-associated quality control are associated with neuromuscular disease. Nat. Commun. **11**, 4625 (2020). 32934225 10.1038/s41467-020-18327-6 PMC7494853

[18] L. Wang , UFMylation of RPL26 links translation-associated quality control to endoplasmic reticulum protein homeostasis. Cell Res. **30**, 5–20 (2020). 31595041 10.1038/s41422-019-0236-6 PMC6951344

[19] L. Wang, Y. Ye, Clearing traffic jam during protein translocation across membrane. Front. Cell Dev. Biol. **8**, 610689 (2021), 10.3389/fcell.2020.610689. 33490075 PMC7820333

[20] Y. Gerakis, M. Quinter, H. Li, C. Hetz, The UFMylation system in proteostasis and beyond. Trends Cell Biol. **29**, 974–986 (2019). 31703843 10.1016/j.tcb.2019.09.005 PMC6917045

[21] S. Banerjee, M. Kumar, R. Wiener, Decrypting UFMylation: How proteins are modified with UFM1. Biomolecules **10**, 1442 (2020), 10.3390/biom10101442. 33066455 PMC7602216

[22] M. S. Nahorski , Biallelic UFM1 and UFC1 mutations expand the essential role of ufmylation in brain development. Brain **7**, 1934–1945 (2018). 10.1093/brain/awy135 PMC6022668 29868776

[23] M. Ni , A pathogenic UFSP2 variant in an autosomal recessive form of pediatric neurodevelopmental anomalies and epilepsy. Genet. Med. **23**, 900–908 (2021). 33473208 10.1038/s41436-020-01071-z PMC8105169

[24] M. Ohira , Gene identification in 1.6-Mb region of the Down Syndrome region on chromosome 21. Genome Res. **7**, 47–58 (1997). 9037601 10.1101/gr.7.1.47

[25] R. H. Reeves, L. L. Baxter, T. Richtsmeier, Too much of a good thing: Mechanisms of gene action in Down syndrome. Trends Genet. **17**, 83–88 (2001). 11173117 10.1016/s0168-9525(00)02172-7

[26] C. P. Walczak , Ribosomal protein RPL26 is the principal target of UFMylation. Proc. Natl. Acad. Sci. U.S.A. **116**, 1299–1308 (2019). 30626644 10.1073/pnas.1816202116 PMC6347690

[27] F. Suizu , The E3 ligase TTC3 facilitates ubiquitination and degradation of phosphorylated Akt. Dev. Cell **17**, 800–810 (2019). 10.1016/j.devcel.2009.09.007 20059950

[28] G. Berto , The Down syndrome critical region protein TTC3 inhibits neuronal differentiation via RhoA and Citron kinase. J. Cell Sci. **120**, 1859–1867 (2007). 17488780 10.1242/jcs.000703

[29] S. Jagannathan, C. Nwosu, C. P. Nicchitta, Analyzing subcellular mRNA localization via cell fractionation. Methods Mol. Bio. **714**, 301–321 (2011). 21431749 10.1007/978-1-61779-005-8_19 PMC3718476

[30] A. Thrun , Convergence of mammalian RQC and C-end rule proteolytic pathways via alanine tailing. Mol. Cell **81**, 2112–2122 (2021). 33909987 10.1016/j.molcel.2021.03.004 PMC8141035

[31] M. Costa-Mattioli, P. Walter, The integrated stress response: From mechanism to disease. Science **368**, aat5314 (2020), 10.1126/science.aat5314. PMC8997189 32327570

[32] R. Higgins , The unfolded protein response triggers site-specific regulatory ubiquitylation of 40S ribosomal proteins. Mol. Cell **59**, 35–49 (2015). 26051182 10.1016/j.molcel.2015.04.026 PMC4491043

[33] D. M. Garshott , iRQC, a surveillance pathway for 40S ribosomal quality control during mRNA translation initiation. Cell Rep. **36**, 109642 (2021), 10.1016/j.celrep.2021.109642. 34469731 PMC8997904

[34] N. T. Ingolia, L. F. Lareau, J. S. Weissman, Ribosome profiling of mouse embryonic stem cells reveals the complexity and dynamics of mammalian proteomes. Cell **147**, 789–802 (2011). 22056041 10.1016/j.cell.2011.10.002 PMC3225288

[35] T. Udagawa , Failure to degrade CAT-tailed proteins disrupts neuronal morphogenesis and cell survival. Cell Rep. **34**, 108599 (2021), 10.1016/j.celrep.2020.108599. 33406423

[36] G. E. Berto , The DCR protein TTC3 affects differentiation and Golgi compactness in neurons through specific actin-regulating pathways. PLos One **9**, e93721 (2014), 10.1371/journal.pone.0093721. 24695496 PMC3973554

[37] J. Chu , A mouse forward genetics screen identifies LISTERIN as an E3 ubiquitin ligase involved in neurodegeneration. Proc. Natl. Acad. Sci. U.S.A. **106**, 2097–2103 (2009). 19196968 10.1073/pnas.0812819106 PMC2650114

[38] F. Guedj , An integrated human/murine transcriptome and pathway approach to identify prenatal treatments for Down syndrome. Scientific Rep. (2016), 10.1038/srep32353. PMC5009456 27586445

[39] S. E. Antonarakis, Down syndrome and the complexity of genome dosage imbalance. Nat. Rev. Genet. **18**, 147–163 (2017). 28029161 10.1038/nrg.2016.154

[40] S. Braat, R. F. Kooy, The GABA_A_ receptor as a therapeutic target for neurodevelopmental disorders. Neuron **86**, 1119–1130 (2015). 26050032 10.1016/j.neuron.2015.03.042

[41] S. Habisov , Structural and functional analysis of a novel interaction motif within UFM1-activating enzyme 5 (UBA5) required for binding to ubiquitin-like proteins and ufmylation. J. Biol. Chem. **291**, 9025–9041 (2016). 26929408 10.1074/jbc.M116.715474 PMC4861472

[42] J. Huber , An atypical LIR motif within UBA5 (ubiquitin like modifier activating enzyme 5) interacts with GABARAP proteins and mediates membrane localization of UBA5. Autophagy **16**, 256–270 (2020). 30990354 10.1080/15548627.2019.1606637 PMC6984602

[43] T. A. Leil, Z. W. Chen, C. S. S. Chang, R. W. Olsen, GABA_A_ receptor-associated protein traffics GABA_A_ receptors to the plasma membranes in neurons. J. Neurosci. **24**, 11429–11438 (2004). 15601949 10.1523/JNEUROSCI.3355-04.2004 PMC6730371

[44] K. K. Hui , GABARAPs dysfunction by autophagy deficiency in adolescent brain impairs GABA_A_ receptor trafficking and social behavior. Sci. Adv. **5**, eaau8237 (2019). 30989111 10.1126/sciadv.aau8237 PMC6457945

[45] J. D. Richter, J. Coller, Pausing on polyribosomes: Make way for elongation in translational control. Cell **163**, 292–300 (2015). 26451481 10.1016/j.cell.2015.09.041 PMC4600128

[46] F. B. Gao, J. D. Richter, D. W. Cleveland, Rethinking unconventional translation in neurodegeneration. Cell **171**, 994–1000 (2017). 29149615 10.1016/j.cell.2017.10.042 PMC5728172

[47] F. Longo, E. Klann, Reciprocal control of translation and transcription in autism spectrum disorder. EMBO Rep. **22**, e52110 (2021). 33977633 10.15252/embr.202052110 PMC8183409

