# Supplementary material for: Dysregulation of ribosome-associated quality control elicits cognitive disorders via overaccumulation of TTC3
Source: Proc Natl Acad Sci U S A. 2023 Mar 14;120(12):e2211522120. doi: 10.1073/pnas.2211522120 (PMC10041068; doi:10.1073/pnas.2211522120)
Supplement: Supplementary file 1 — Appendix 01 (PDF) [file pnas.2211522120.sapp.pdf]

Fig. S1

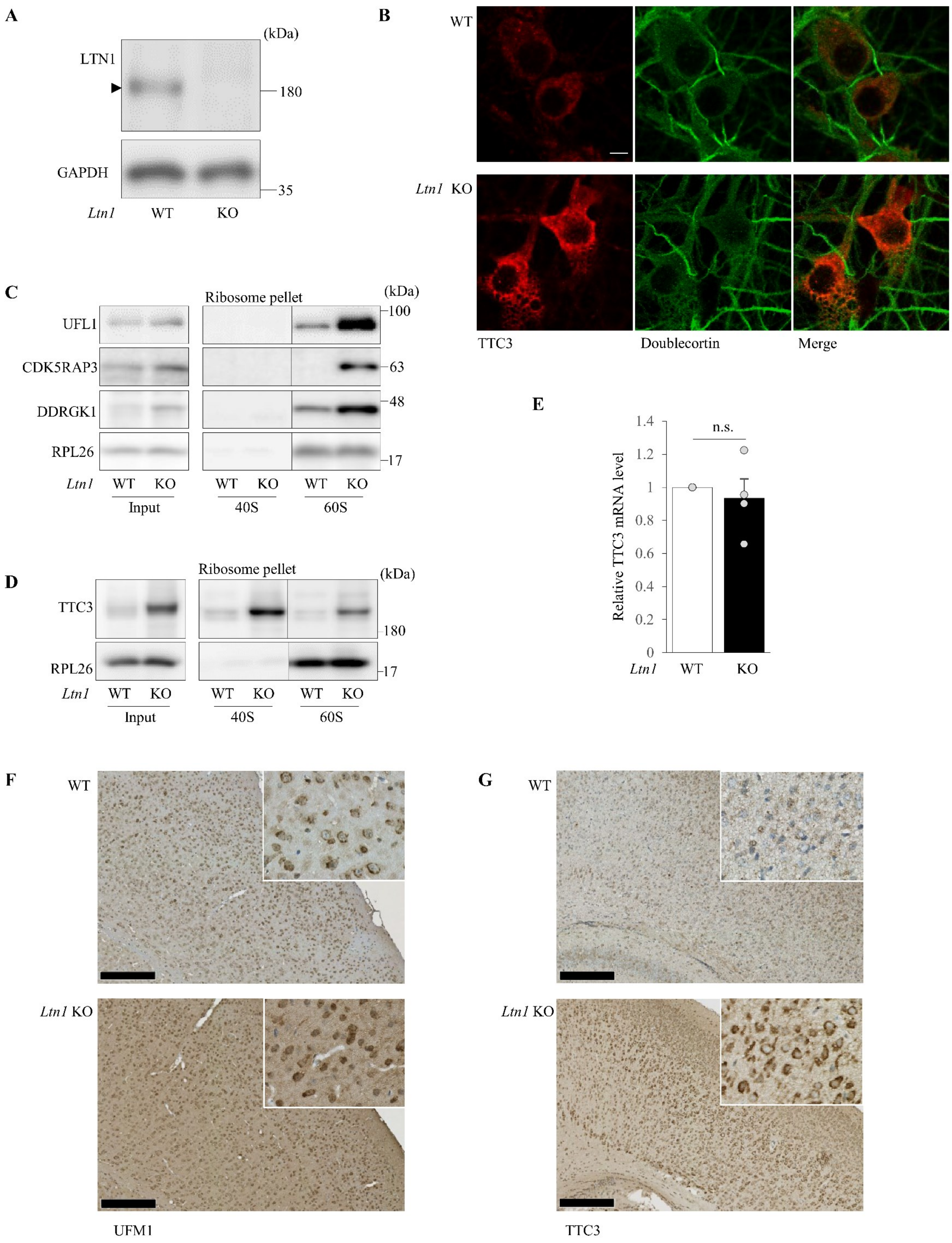

A
(kDa)
LTN1
180
GAPDH
35
Ltn1 WT KO
B
WT
Ltn1 KO
TTC3
Doublecortin
Merge
C
Ribosome pellet
(kDa)
UFL1
100
CDK5RAP3
63
48
DDRGK1
RPL26
17
Ltn1 WT KO WT KO WT KO
Input 40S 60S
D
Ribosome pellet
(kDa)
TTC3
180
RPL26
17
Ltn1 WT KO WT KO WT KO
Input 40S 60S
E
n.s.
Relative TTC3 mRNA level
1.4
1.2
1
0.8
0.6
0.4
0.2
0
Ltn1 WT KO
F
WT
Ltn1 KO
UFM1
G
WT
Ltn1 KO
TTC3

Fig. S1. TTC3 and UFMylation signaling protein levels are increased in *Ltn1* KO neurons. (*A*) LTN1 in cultured cortical neurons from WT and *Ltn1* KO mice were detected by western blotting with an antibody against LTN1. An arrowhead indicates full length LTN1 in WT neurons. (*B*) Cultured cortical neurons were immunostained with anti-TTC3 (red) and neuronal cell marker anti-doublecortin (green) antibodies. Scale bar represents 25 μm. (*C* and *D*) 40S and 60S subunit fractions were collected from cultured cortical neurons by sucrose gradient centrifugation and the ribosome subunits were further pelleted down and analyzed by western blotting with (*C*) anti-UFL1, anti-CDK5RAP3, anti-DDRGK1, anti-RPL26 antibodies or (D) anti-TTC3 and anti-RPL26 antibodies. (*E*) Total RNAs were isolated from mouse cortex and subjected to RT-qPCR for quantitative analysis of mRNA levels of TTC3. mRNA levels of TTC3 were normalized to those of β-actin, and then expressed as the relative ratio of WT. (n=4, P=0.612472, unpaired two-tailed Student's t-test). (*F* and *G*)**,** Coronal sections from WT or *Ltn1* KO mouse brains were immunostained with (*F*) an anti-UFM1 antibody or (*G*) an anti-TTC3 antibody. Representative images of cerebral cortex are shown. Insets represent zoom-up views. Scale bars represent 250 μm. Immunohistochemistry against UFM1 and TTC3 showed markedly enhanced protein expression of UFM1 and TTC3 in cortical neuronal cells of *Ltn1* KO mouse brains. Data represent means ± S.E.M.

Fig. S2

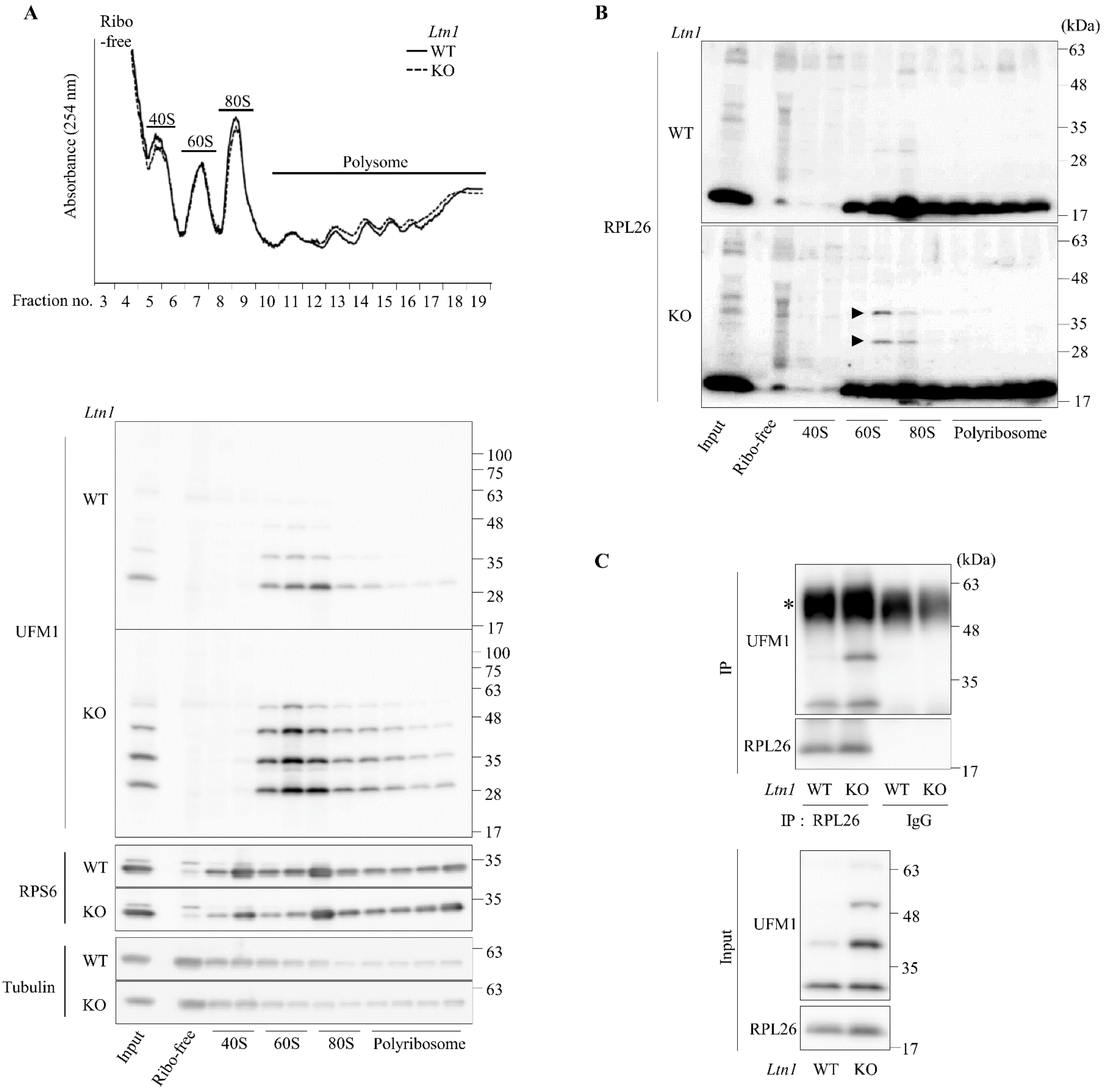
A
Ribo -free
Ltn1
WT
KO
40S
60S
80S
Polysome
Absorbance (254 nm)
Fraction no. 3 4 5 6 7 8 9 10 11 12 13 14 15 16 17 18 19
Ltn1
WT
KO
UFM1
RPS6
Tubulin
100
75
63
48
35
28
17
Input
Ribo-free
40S
60S
80S
Polyribosome
B
Ltn1
(kDa)
WT
KO
RPL26
63
48
35
28
17
Input
Ribo-free
40S
60S
80S
Polyribosome
C
(kDa)
IP
UFM1
RPL26
*
63
48
35
17
Ltn1 WT KO WT KO
IP : RPL26 IgG
Input
UFM1
RPL26
Ltn1 WT KO

Fig. S2. UFMylated RPL26 proteins are upregulated in *Ltn1* KO neurons. (*A* and *B*) UFMylated RPL26 proteins are enriched in 60S ribosome fraction in LTN1-deficient neurons. Ribosome-free (Free), 40S, 60S, 80S monosome and polysome fractions were obtained from cultured cortical neurons by sucrose gradient centrifugation and detected with (*A*) an anti-UFM1 antibody or (*B*) an anti-RPL26 antibody. UFMylated .RPL26 in *Ltn1* KO neurons are indicated by arrowheads. A representative absorption profile of sucrose gradient at 254 nm is shown (*A:*top). (*C*) RPL26 was immunoprecipitated with an anti-RPL26 antibody from neuronal lysates, followed by western blotting with an anti-UFM1 antibody. Asterisk denotes a heavy chain of IgG.

Fig. S3

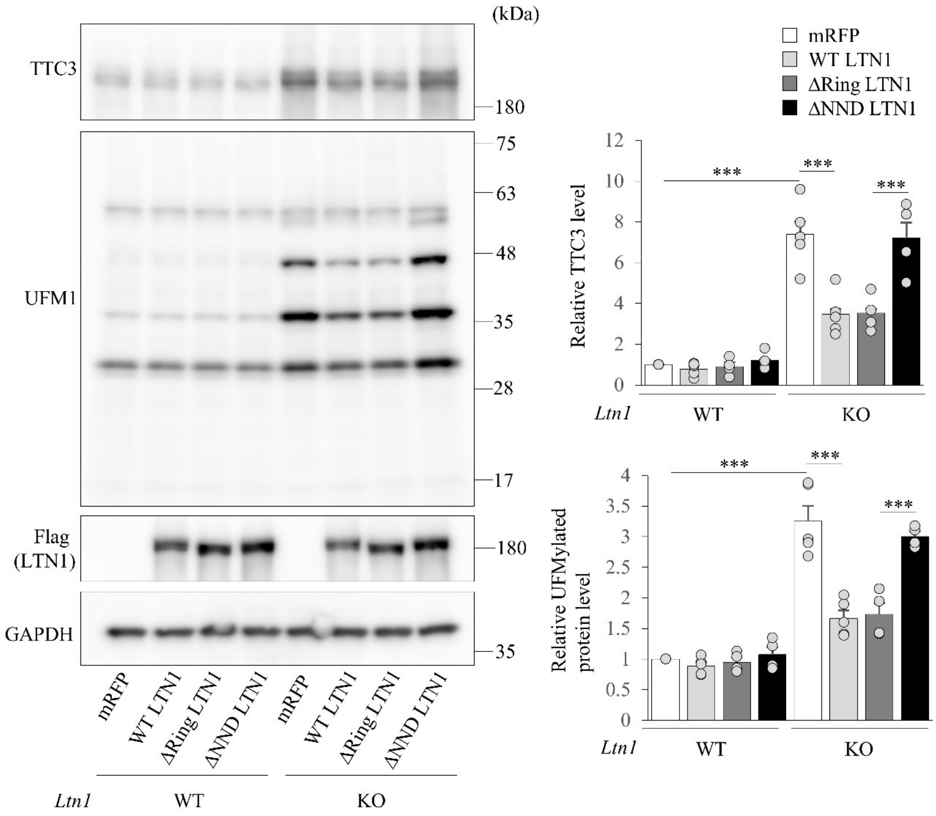
(kDa)
TTC3
180
UFM1
75
63
48
35
28
17
Flag
(LTN1)
180
GAPDH
35
mRFP
WT LTN1
ΔRing LTN1
ΔNND LTN1
mRFP
WT LTN1
ΔRing LTN1
ΔNND LTN1
Ltn1
WT
KO
mRFP
WT LTN1
ΔRing LTN1
ΔNND LTN1
Relative TTC3 level
12
10
8
6
4
2
0
***
***
***
Ltn1
WT
KO
Relative UFMylated protein level
4
3.5
3
2.5
2
1.5
1
0.5
0
***
***
***
Ltn1
WT
KO

Fig. S3. An increase in UFMylated RPL26 andTTC3 protein levles in *Ltn1* KO neurons depends on LTN1's localization on 60S subunit but not its E3 ligase activity. Cultured cortical neurons were infected with lentivirus encoding Flagx3-tagged mRFP, WT LTN1, ΔRing LTN1or ΔNND LTN1, and TTC3 and UFMylated proteins were detected by western blotting. The levels of TTC3 (top) and UFMylated proteins (bottom) relative to those in control (WT+mRFP) neurons are shown (n=4, TTC3: $F(7,28)=35.0$, $P<0.0001$; UFM1: $F(7,28)=46.2$, $P<0.0001$; one-way ANOVA; ***$P<0.001$, Bonferroni's multiple comparison test *post hoc*). Data represent means ± S.E.M.

Fig. S4

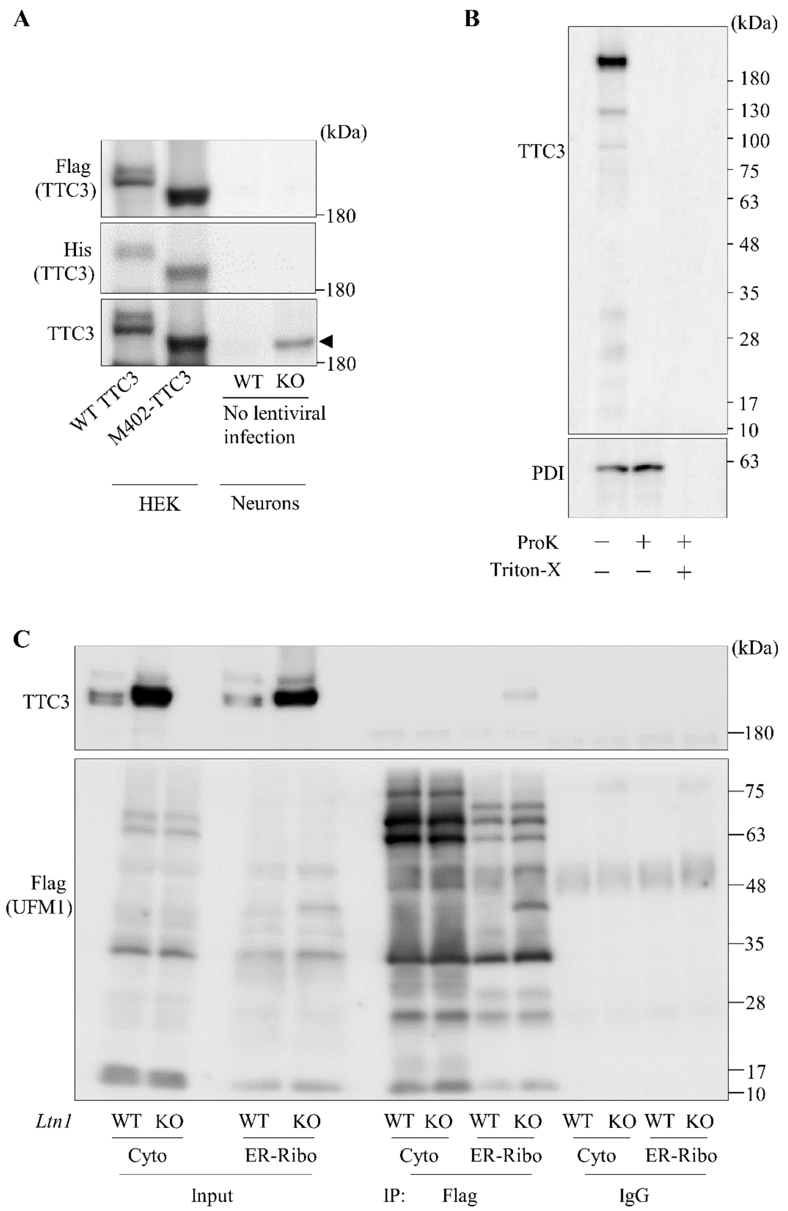
A
(kDa)
Flag
(TTC3)
180
His
(TTC3)
180
TTC3
180
WT TTC3
M402-TTC3
WT KO
No lentiviral
infection
HEK
Neurons
B
(kDa)
TTC3
180
130
100
75
63
48
35
28
17
10
PDI
63
ProK
Triton-X
C
(kDa)
TTC3
180
Flag
(UFM1)
75
63
48
35
28
17
10
Ltn1
WT KO
Cyto
WT KO
ER-Ribo
Input
IP:
WT KO
Cyto
WT KO
ER-Ribo
Flag
WT KO
Cyto
WT KO
ER-Ribo
IgG

Fig. S4. An N-terminally truncated form of TTC3 is dominantly expressed in neurons and localized in 40S subunits. (*A*) Overexpressed full-length Flagx3 TTC3-Hisx6 and N-terminally truncated Flagx3-M402-TTC3-Hisx6 in HEK 293T cells were detected by an anti-Flag antibody (top) and an anti-His antibody (middle). Detection by both antibodies indicate that the M402-TTC3 protein is fully expressed. Both overexpressed TTC3 in HEK293T cells and endogenous TTC3 in cultured cortical neurons were detected by an anti-TTC3 antibody (bottom) by western blotting. Endogenous TTC3 in cultured cortical neurons and overexpressed M402-TTC3 are indicated by an arrowhead. (*B*) Isolated ER-membrane fraction was treated with 0.5 mg/ml Proteinase K in the absence or presence of 1% Triton-X and western blotting was performed with indicated antibodies. TTC3 was not protected from Proteinase K digestion, while PDI is resistant to Proteinase K digestion unless membrane is solubilized with Triton-X. (*C*) Cultured cortical neurons were infected with lentivirus encoding Flagx3-tagged C-terminally truncated (Δsc) UFM1 to expose the reactive C-terminal Gly (Flagx3-TEV-UFM1Δsc). UFMylted proteins were immunoprecipitated using an anti-Flag antibody from cytosolic and ER-Ribo fractions, followed by detection with indicated antibodies.

Fig. S5

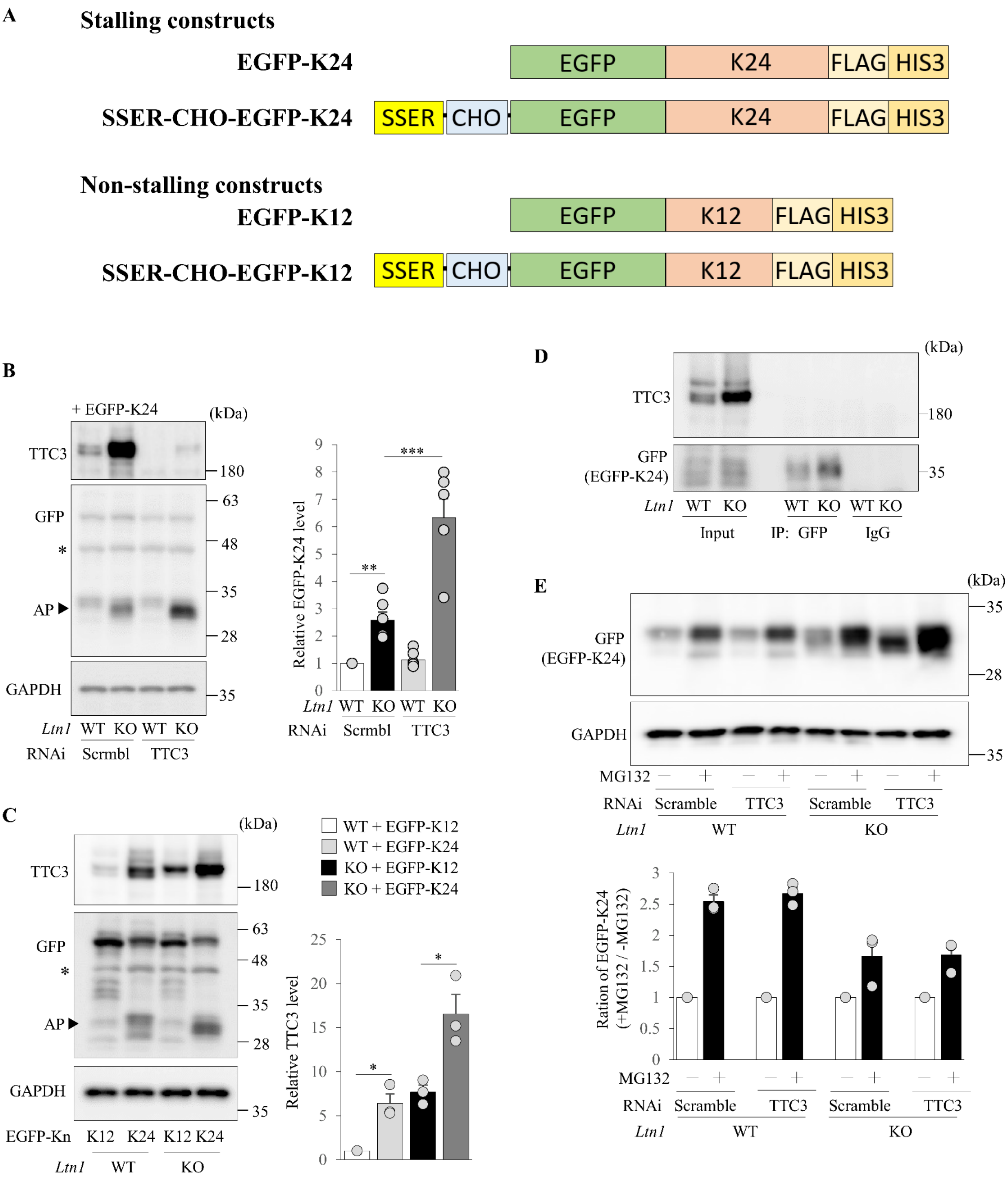

A
Stalling constructs
EGFP-K24
EGFP
K24
FLAG
HIS3
SSER-CHO-EGFP-K24
SSER
CHO
Non-stalling constructs
EGFP-K12
K12
SSER-CHO-EGFP-K12
B
+ EGFP-K24
(kDa)
TTC3
180
GFP
63
48
*
AP ▶
35
28
GAPDH
35
Ltn1 WT KO WT KO
RNAi Scrmbl TTC3
Relative EGFP-K24 level
**
***
C
WT + EGFP-K12
WT + EGFP-K24
KO + EGFP-K12
KO + EGFP-K24
EGFP-Kn K12 K24 K12 K24
Ltn1 WT KO
Relative TTC3 level
*
D
TTC3
GFP
(EGFP-K24)
Ltn1 WT KO WT KO WT KO
Input
IP: GFP
IgG
E
GFP
(EGFP-K24)
GAPDH
MG132 − + − + − + − +
RNAi Scramble TTC3 Scramble TTC3
Ltn1 WT KO
Ration of EGFP-K24
(+MG132 / -MG132)

Fig. S5. TTC3 protein is abnormally accumulated by an overload of translationally arrested K24 products and is not involved in degradation of translationally arrested products in stalled ribosomes. (*A*) Reporter constructs for detection of ribosome stalling used in this study. (*B*) Cultured cortical neurons were first infected with lentivirus encoding scramble RNAi or TTC3 RNAi, followed by infection of AAV encoding EGFP-K24. Arrested products (AP; indicated by arrowhead) were detected by western blotting using an anti-GFP antibody. The signal intensities of arrested products were normalized to those of GAPDH, and then expressed as the relative ratio of WT+scramble RNAi neurons (right) (n=6, $F(3,15)=51.5$, $P<0.0001$; one-way ANOVA; **$P<0.01$, ***$P<0.001$, Bonferroni's multiple comparison test *post hoc*). Asterisk indicates non-specific band. (*C*) Cultured cortical neurons were infected with indicated AAVs, and TTC3 protein level was detected by western blotting. Arrested products (AP) are indicated by arrowhead and asterisk denotes non-specific band. The signal intensities of TTC3 were normalized to those of GAPDH, and then expressed as the relative ratio of WT neurons infected with control AAVs (n=3, $F(3,6)=19.5$, $P=0.0017$, one-way ANOVA; *$P<0.05$, Bonferroni's multiple comparison test *post hoc*). (*D*) Cultured cortical neurons were infected with AAV encoding SSER-CHO-EGFP-K24. Arrested products were immunoprecipitated using anti-GFP antibody from neuronal lysates, followed by detection with indicated antibodies. (*E*) Cultured cortical neurons were first infected with lentivirus encoding scramble or TTC3 RNAi, followed by infection of AAV encoding EGFP-K24. The neurons were treated with or without a proteasome inhibitor, MG132, and arrested products were detected by western blotting using anti-GFP antibody. Stabilization of arrested products by MG132 is shown as a ratio of with MG132 treatment to control treatment. Data represent means ± S.E.M.

Fig. S6

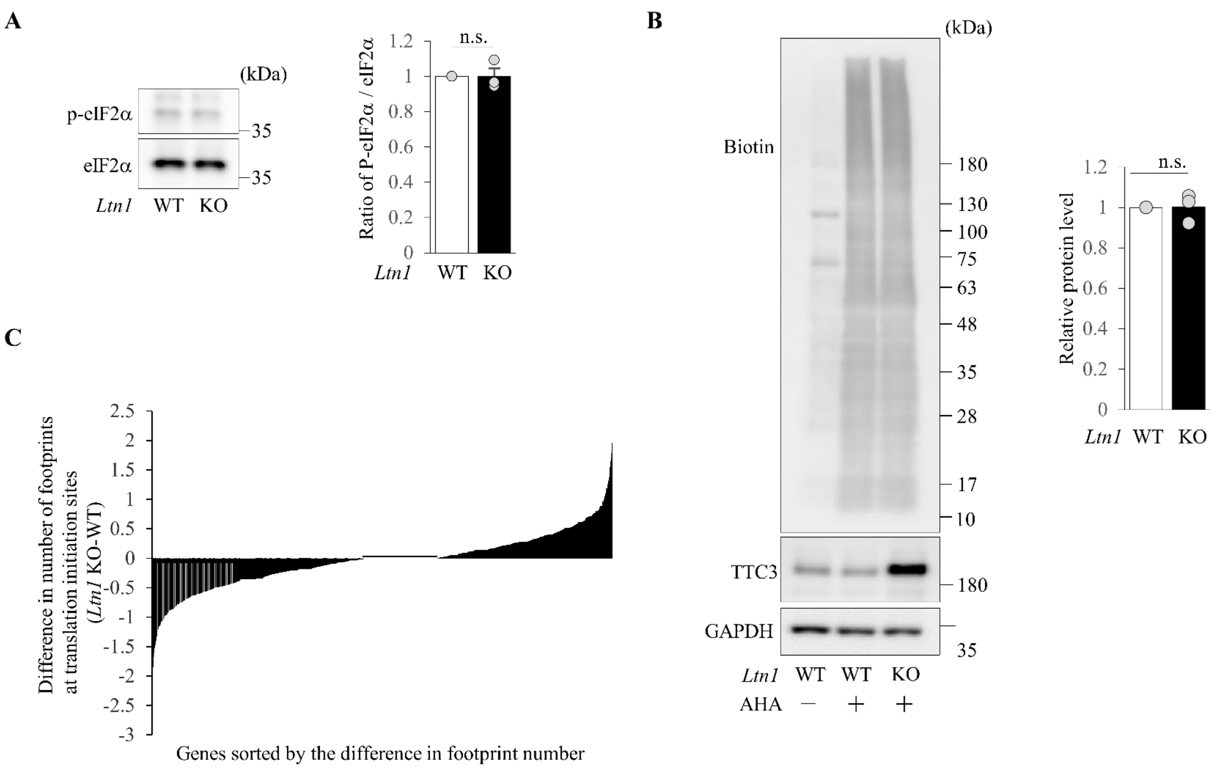

D

GO Molecular Function

Term name	Term ID	P_{adj}
protein binding	GO:0005515	4.457×10^{-13}
catalytic activity	GO:0003824	8.549×10^{-10}
binding	GO:0005488	3.041×10^{-9}
ion binding	GO:0043167	1.208×10^{-4}
enzyme binding	GO:0019899	1.269×10^{-4}
ubiquitin-like protein ligase binding	GO:0044389	4.527×10^{-4}
nucleoside phosphate binding	GO:1901265	5.870×10^{-4}
nucleotide binding	GO:0000166	5.870×10^{-4}
catalytic activity, acting on a protein	GO:0140096	1.266×10^{-3}
transcription coactivator activity	GO:0003713	1.584×10^{-3}

-log10(P_{adj})

GO Biological Process

Term name	Term ID	P_{adj}
organonitrogen compound metabolic process	GO:1901564	2.962×10^{-18}
cellular macromolecule metabolic process	GO:0044260	1.920×10^{-14}
protein metabolic process	GO:0019538	5.062×10^{-14}
cellular protein metabolic process	GO:0044267	5.379×10^{-14}
cellular process	GO:0009987	3.495×10^{-14}
protein modification process	GO:0036211	4.549×10^{-9}
cellular protein modification process	GO:0006464	4.549×10^{-9}
cellular metabolic process	GO:0044237	5.660×10^{-9}
macromolecule modification	GO:0043412	1.097×10^{-8}
metabolic process	GO:0008152	1.542×10^{-7}

-log10(P_{adj})

Fig. S6. A Subset of mRNAs showed reduced translation initiation by the loss of LTN1. (*A*) Phosphorylation of eIF2α is not increased in *Ltn1* KO neurons. Phosphorylated and total eIF2α proteins were detected by western blotting with indicated antibodies (n=3, P=0.9846, unpaired two-tailed Student's t-test). n.s., not significant. (*B*) Cultured cortical neurons were treated with or without 2 mM AHA for 6 hours, followed by click chemistry and AHA-incorporated polypeptides were detected by western blotting using anti-biotin antibody. (n=3, P=0.9573, unpaired two-tailed Student's t-test). n.s., not significant. (*C*) Ranked genes sorted by the difference in footprint number in translation initiation between harringtonine treated WT and *Ltn1* KO neurons. (*D*) GO term analysis by g:Profiler for top 5% genes which showed decreased translation initiation. Top ten GO terms for molecular function and biological process are shown. Data represent means ± S.E.M.

Fig. S7

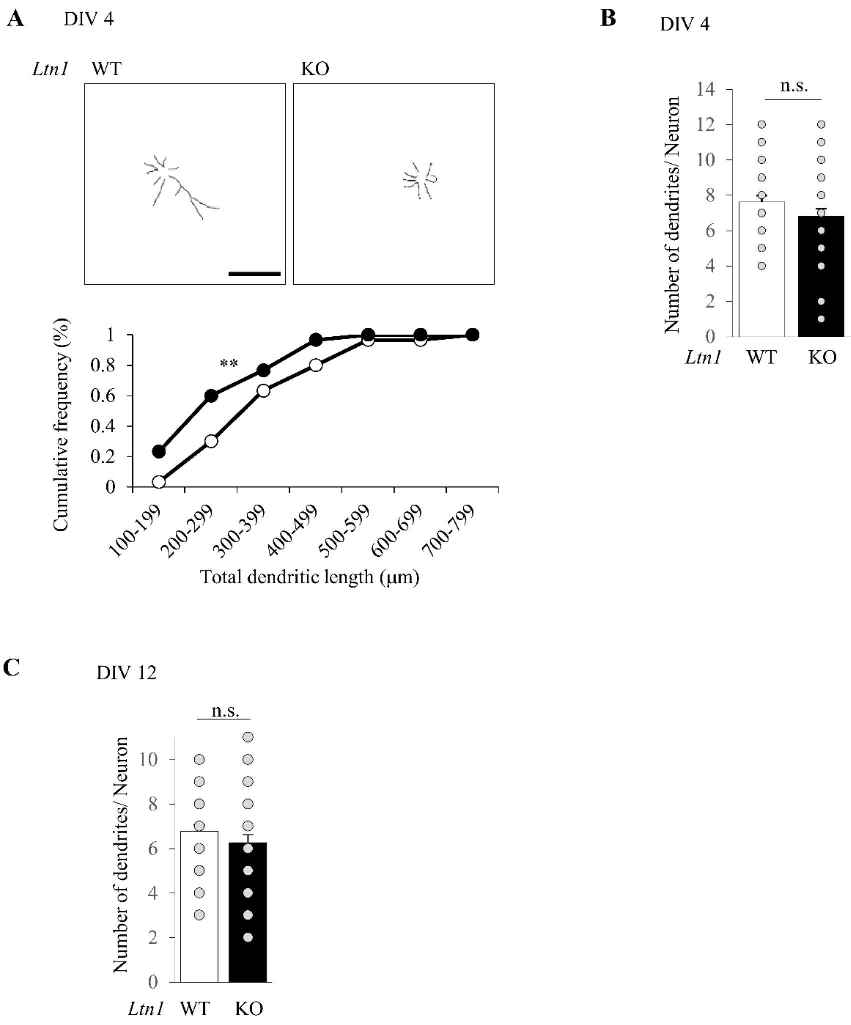
A
DIV 4
Ltn1 WT
KO
Cumulative frequency (%)
1
0.8
0.6
0.4
0.2
0
**
100-199
200-299
300-399
400-499
500-599
600-699
700-799
Total dendritic length (µm)
B
DIV 4
Number of dendrites/ Neuron
14
12
10
8
6
4
2
0
n.s.
Ltn1 WT KO
C
DIV 12
Number of dendrites/ Neuron
10
8
6
4
2
0
n.s.
Ltn1 WT KO

Fig. S7. Dendritic outgrowth is impaired in pre-matured, but not matured, cortical neurons by the loss of LTN1. (*A*-*C*) Dendrites of cultured cortical neurons at DIV 4 (*A* and *B*) or at DIV 12 (*C*) were stained with an anti-MAP2 antibody and evaluated. (*A*) Top panels show representative tracings of dendritic morphology. Total dendritic length was measured (bottom) (n=30, **P=0.0062, Kolmogorov-Smirnov test). Scale bar represents 100 μm. (*B*) The number of dendrites per neuron was counted (n=30, P=0.146, unpaired two-tailed Student's t-test). (*C*) The number of dendrites per neuron at DIV 12 was counted (n=38, P=0.2611, unpaired two-tailed Student's t-test). n.s., not significant. Data represent means ± S.E.M.

Fig. S8

A

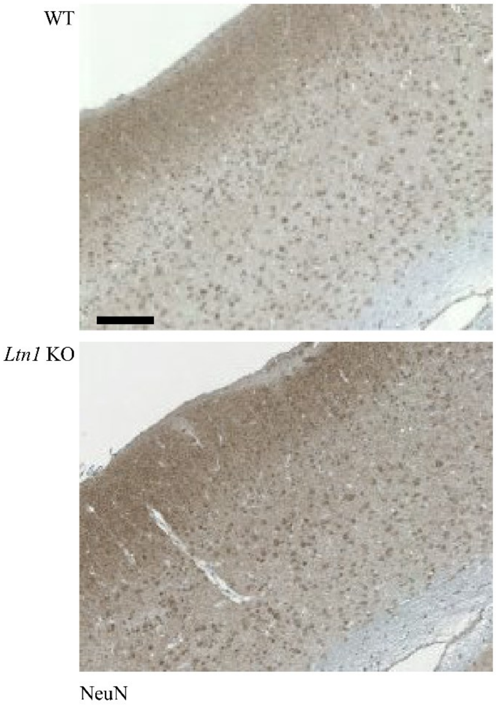

WT
Ltn1 KO
NeuN

B

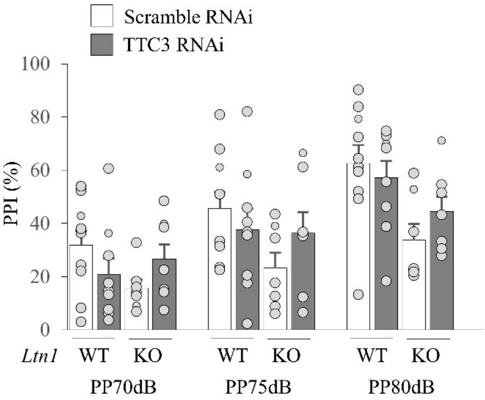

Scramble RNAi
TTC3 RNAi
100
80
60
40
20
0
PPI (%)
Ltn1
WT
KO
WT
KO
WT
KO
PP70dB
PP75dB
PP80dB

Fig. S8. Immunohistological analysis revealed that *Ltn1* KO mice do not show any obvious neuronal loss and KD of TTC3 partly counteracted the reduced PPI in *Ltn1* KO mice. (*A*) Mouse brain coronal sections were immunostained with an anti-NeuN antibody. No obvious loss of NeuN positive neurons were observed in *Ltn1* KO cortex. Scale bar represents 250 μm. (*B*) AAV encoding scramble or TTC3 RNAi was stereotaxically injected into mouse medial prefrontal cortex and PPI test was performed as in Fig. 5a. n=14 (WT+Scramble RNAi), n=14 (WT+TTC3 RNAi), n=12 (KO+Scramble RNAi), n=13 (KO+TTC3 RNAi). (70dB: $F(3,29)=1.66$, $P=0.1975$; 75 dB: $F(3,29)=1.62$, $P=0.2068$; 80 dB: $F(3,29)=3.80$, $P=0.0206$, one-way ANOVA; Bonferroni's multiple comparison test *post hoc*). n.s., not significant. Data represent means ± S.E.M.

Fig. S9

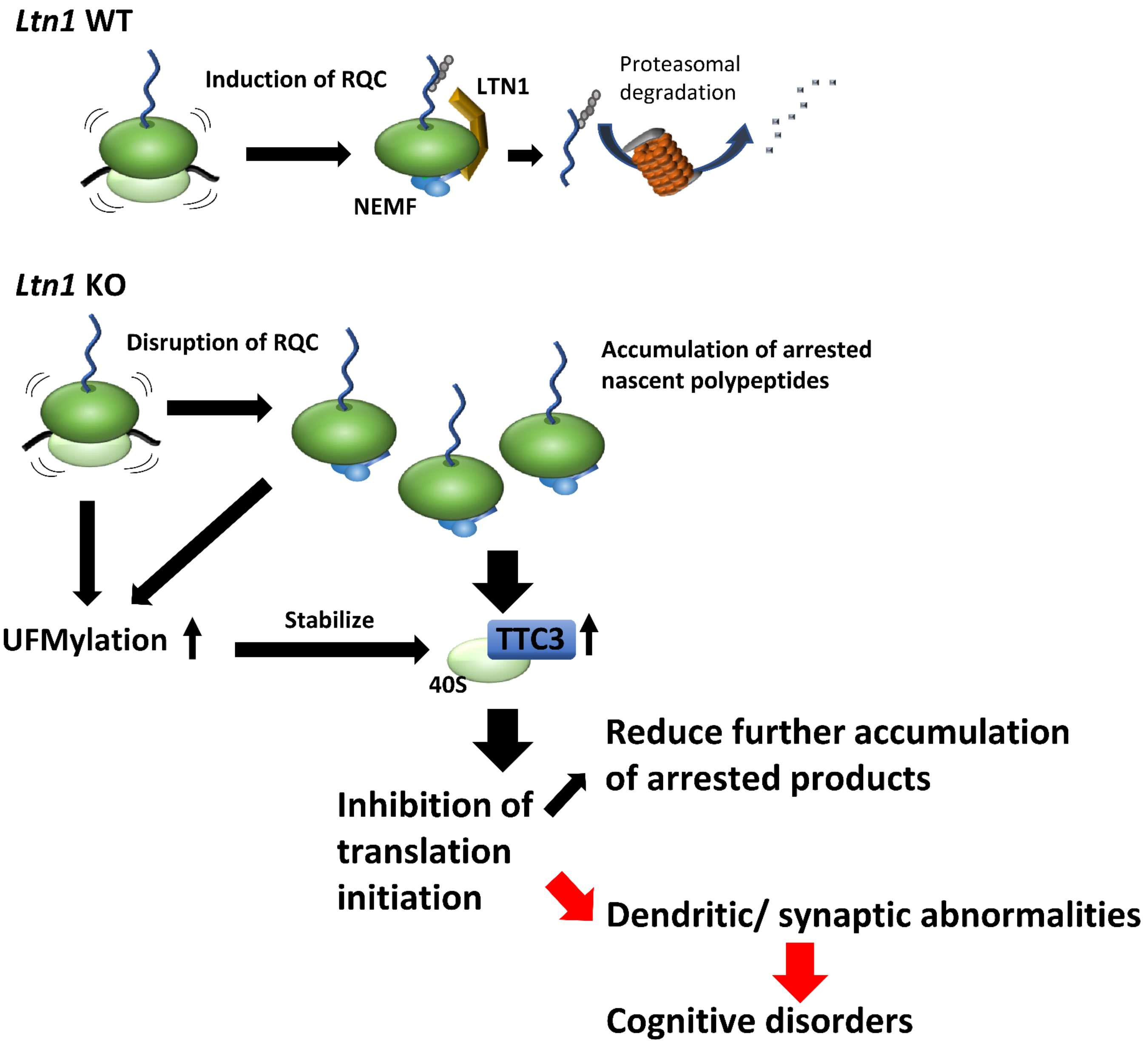

Ltn1 WT
Induction of RQC
LTN1
NEMF
Proteasomal
degradation
Ltn1 KO
Disruption of RQC
Accumulation of arrested
nascent polypeptides
UFMylation
Stabilize
TTC3
40S
Reduce further accumulation
of arrested products
Inhibition of
translation
initiation
Dendritic/ synaptic abnormalities
Cognitive disorders

Fig. S9. Schematic model of TTC3-mediated impairments of dendritic outgrowth and behavioral deficits associated with cognitive disorders in *Ltn1* KO mice. In WT neurons, LTN1 polyubiquitinates nascent polypeptides for their proteasomal degradation. In *Ltn1* KO neurons, TTC3 protein level is substantially increased in response to overload of translationally arrested products caused by the disruption of RQC. TTC3 is partially stabilized by increased UFMylation signaling and prevents further accumulation of translationally arrested products possibly by preventing translation initiation, thereby further translation. However, abnormally overaccumulated TTC3 in *Ltn1* KO neurons in turn causes dendritic and synaptic abnormalities and behavioral deficits associated with cognitive disorders.

Supporting Information

METHODS

Generation of *Ltn1* KO mouse

Three single guide RNAs (sgRNAs) sequences targeting *Exon1* (sgRNA1, *5'-gaagaacaagcagcggacta-3'*) and *Exon2* (sgRNA2, *5'-tgttccggctgttcaaggcg-3'*; sgRNA3, *5'-tgtagattccgat ttccgaa-3'*) of the mouse LTN1 gene, respectively, were first subcloned into pX330-sgRNA plasmid (a gift from Dr. T. Takumi) and transcribed *in vitro* using the MEGAshortscript T7 Kit (Life Technologies) according to manufacturer's instructions. *Ltn1* KO mice were generated using CRISPR/Cas9 system, as previously described by Yang and colleagues (1). Briefly, mixture of Cas9 mRNA (10 ng/μl) and sgRNAs (3.5 ng/μl each) were microinjected into the cytoplasm of C57BL/6J zygotes and were planted into pseudopregnant ICR recipients. 10 bp-deletion in *Exon1* of the LTN1 gene was confirmed through PCR genotyping of tail DNA. PCR products were further verified by Sanger sequencing analyses. The following primers were used for genotyping: Forward, *5'-ggctcaacgctggtttcttat-3'*; Reverse, *5'-gtgctcactgtcaccgtttc3'* ; Sequencing, *5'-agcggatggtcagagtcct-3'*. *Ltn1* KO mice were backcrossed to C57BL/6J background for at least 12 generations.

Animals

All animals used in this study were housed at a maximum of 5 per cage and in a temperature-controlled room under a 12-h light/12-h dark cycle with free access to food and water. All of the experiments were performed in compliance with relevant laws and guidelines issued by RIKEN.

Antibodies

TTC3 rabbit polyclonal antibody C2 was raised against the recombinant TTC3 protein fragment residues 1801-1979 in mouse TTC3 (Research Resources Division in RIKEN Center for Brain Science). TTC3 mouse monoclonal antibodies 3D4 and 2F6 were raised against the recombinant mouse TTC3 protein fragments; residues 1-129 and residues 1801-1979 in mouse TTC3, respectively. In this study, we used the antibodies against UFM1 (rabbit polyclonal, Abnova, ab109305), UFL1 (rabbit polyclonal, Bethyl Labs, A303-456A), RPL26 (rabbit polyclonal, Bethyl Labs, A300-686A), Histone H3 (rabbit polyclonal, Cell Signaling, 9715), GAPDH (mouse monoclonal, SantaCruz, sc32233), CDK5RAP3 (Rabbit polyclonal, Atlas Antibodies, HPA022882), DDRGK1 (Rabbit polyclonal, Proteintech, 21445-1-AP), GFP (rabbit polyclonal, MLB, 598), puromycin (mouse monoclonal, Millipore, MABE343), Gephyrin (mouse monoclonal, Synaptic Systems, 147-111), vGlut1 (mouse monoclonal, NeuroMab, 75-066), Synaptotagmin-1 (SYT1) (mouse monoclonal, SantaCruz, sc136480), NR2B (mouse monoclonal, NeuroMab, 75-028), PSD95 (mouse monoclonal, NeuroMab, 75-028), Snapin (mouse monoclonal, NeuroMab, 75-045), VGAT (Rabbit polyclonal, Millipore, AB5062P), $GABA_A\beta3$ (mouse monoclonal, NeuroMab, 75-149), GluR1 (mouse monoclonal, NeuroMab, 75-002), SV2 (mouse monoclonal, DSHB, SV2) MAP2a/b (mouse monoclonal, NeoMarker).

Production of lentivirus and AAV

Lentiviruses were produced by co-transfection of pCSII-CMV-mRFP, pCSII-CMV-flagx3-ΔNDD LTN1, pCSII-CMV-HAx3-LTN1, pCSII-CMV-HAx3-ΔRing LTN1 with pCAG-HIVgp and pCAG-VSV-G plasmids or expression vectors of pSIH1-copGFP-Scramble RNAi, pSIH1-copGFP-TTC3 RNAi or pSIH1-copGFP-UFM1 RNAi with

helper constructs VSV-G and delta8.9 into Lenti-X 293T cells grown in 10 ml of Dubecco's Modified Eagle's Medium (DMEM, Gibco) supplemented with 10% fetal bovine serum. Infectious lentiviruses were precipitated from cultured medium using Lenti-X concentrator (Clontech) according to manufacturer's protocol. Resulting pellets were resuspended with 500 μl of MACS Neuro Medium (Miltenyi Biotec) supplemented with Neuro Brew-21 (Miltenyi Biotec), 0.5 mM L-glutamine, penicillin and streptomycin (Nacalai). For AAV production, pAAV2 plasmid encoding Scramble RNAi-IRES-mRFP or TTC3 RNAi-IRES-mRFP was co-transfected with pAAV2-2/1 and pAd delta F6 helper plasmids into AAV-293 cells. AAVs were purified by iodixanol gradient centrifugation as explained elsewhere (2).

Infection of AAV into mice

AAV encoding Scramble RNAi or TTC3 RNAi was bilaterally injected into the frontal cortex by using the following coordinates from Bregma: AP 1.8, ML +/-0.35, DV -2.85. 1 μl of AAV was injected with the rate of 0.1 μl/min by a microinjector (Narishige). In 2 weeks after the injection, a range of behavioral tests were performed as described below.

Mouse behavioral experiments

All behavioral experiments were conducted using male mice during the light phase. All experiments were blinded; the operators responsible for the experimental procedures were blinded and unaware of group allocation throughout the experiment.

The elevated plus maze was performed as previously described (3). Briefly, a single channel of EPM (closed arms: 25x5x15 cm (H); open arms: 25x5x0.3 cm (H)) was placed in a soundproof room. Arms were arranged orthogonally 60 cm above the floor. Mice were individually placed on the central platform facing an open arm and were then allowed to move freely in the maze for 5 min. % of time spent in the open arms was measured.

PPI test was performed according to a previously described method (3). Briefly, mice were placed into the soundproof chamber [60x50x67 (H)] containing a sound generator and movement detector (O'Hara) and habituated to the background experimental environment with 65 dB of background white noise. PPI was tested by applying 120 dB (40 ms) startle stimulus alone or preceded by a prepulse stimulus of 70, 75 or 80 dB (20 ms, lead time 100 ms). Each trial was performed 10 times in quasi-random order and with random inter-trial intervals (10 to 20 sec). Eventually, 120 dB startle stimuli (40 ms) was presented to the mice 10 times with random inter-trial intervals (10 to 20 sec).

Fear conditioning test was performed as described previously (3). Briefly, fear conditioning was performed in a conditioning chamber equipped with a stainless-steel grid floor [34×26×30 (H) cm] connected to a shock generator. Mice were fear conditioned with two conditioned stimulus (CS, 30 sec, 65 dB white noise) to unconditioned stimulus (US, 2 sec, 0.5 mA foot shock) pairing separated by 2 min. Mice were subjected to a context test twenty-four hours after the fear conditioning. The context test was performed in the same conditioning chamber for 3 min in the absence of the white noise. A cued test was also performed 24 hours after the context test in the

test chamber different from the conditioning chamber in terms of luminance (about 0 to 1 lx), color (white), floor structure [no grid but with thin bedding material (Alpha-Dri: Shepherd, TN, USA)], and shape (triangular). The CS tone (no foot shock) sounded for 2 min after exploration period of 2 min (no CS). Behavior was recorded by an overhead CCD camera and the rate of freezing (i.e., absence of movement except for breathing and heartbeat). Freezing (i.e., absence of movement except for breathing) was measured as an index of fear memory.

In Marble burying test, fresh bedding was added to a new cage to a depth of 3.5-4.0 cm, and 15 glass marbles (1.5 cm diameter) were placed with equal spacing (3 × 6 grid) on the bedding. Mice were placed individually in the cage and the number of buried marbles was manually counted. Marbles that surface area was covered more than 20% by bedding was defined as buried marble.

For Rotarod test, mice were placed on a rotating rod (Muromachi Kikai), and the latency time for which the mouse was able to run on the rod was measured. The speed of rotation was 4 rpm on day 1 and was increased from 4 to 40 rpm over a 4-minute period and then kept at 40 rpm for another 1 minute from day 2 to day 5. Mice were tested in 1 trial for 2 minutes on day 1 and in 4 trials with a maximum time of 300 seconds (intervals between trials were 20–30 seconds) on days 2–5. The latency time of mice on the rotating rod was recorded by the instrument.

The open field test was performed by placing mouse in the center of an open field apparatus (50x50x40 cm) illuminated by light emitting diode (LEDs; 70 lx at the center

of the field). Mouse was allowed to move freely for 15 min and distance traveled (cm) was adopted as indices and the relevant data were acquired every 1 min. Data were collected and analyzed by using TimeOFCR4 (O'Hara & Co.).

The nest building test was performed according to the scoring system as described previously (4). Briefly, mice were placed into individual cages and were given one piece of cotton fiber as a bedding material. Photos of the cages were taken 72 hours later, and a score was assigned according to the rating scale.

Immunoprecipitation and western blotting

Mouse brains or cultured cortical neurons were lysed in immunoprecipitation buffer (50 mM Tris-Cl pH7.4, 150 mM NaCl, 1 mM EDTA, 1 mM $MgCl_2$, 1% Nonidet P-40, 1 mM dithiothreitol (DTT)) supplemented with complete EDTA-free protease inhibitor cocktail (Nacalai tesque) and centrifuged at 1500×*g* for 5 min and the supernatants were collected. Typically, Protein G-dynabeads (Invitrogen) were incubated with an antibody against UFM1 (abnova), TTC3 (C2, 3D4) or Flag (Sigma) for 2 hours at 4°C and cell or brain lysates were incubated for additional 2 hours at 4°C. The immunoprecipitates were subjected to western blotting using following antibodies: anti-TTC3 (C2, 3D4), anti-RPL26 (Bethyl Labs), anti-Myc (MBL) and anti-HA (MBL) antibodies.

Primary cortical neuron culture and viral infection

Primary cortical neurons were prepared from embryonic day 14 to 15 mouse embryos. Briefly, brain cortices were dissected from embryos, trypsinized and plated at a density of 2.0×10^5 cells/cm^2 in polyethyleneimine (PEI, Sigma)-coated 6 well plates or at a

density of 0.5×10^5 cells/cm^2 in 24 well plates with coverslips in Dubecco's Modified Eagle's Medium (DMEM, Gibco) supplemented with 10% fetal bovine serum. The medium was then changed to Neuro Medium (MiltyniBiotec) supplemented with 0.5 mM L-glutamine and Neuro Brew-21 (MiltyniBiotec). Typically, for 6 well plates, 40 to 50 µl of lentivirus was infected at day 2 to 3 *in vitro* (DIV) for knockdown of TTC3 and UFM1 or at 6 to 7 DIV for overexpression. For biochemical assays, neurons were harvested with cold phosphate buffered saline (PBS) by centrifugation at 1000×*g* for 5 min and the cell pellets were stored at -80°C until use.

Immunocytochemistry and measurement of total dendritic length

Cortical neurons cultured on coverslips were fixed with 4% paraformaldehyde, 4% sucrose in PBS. Cells were permeabilized with 0.1% triton X-100 in PBS for 15 min prior to blocking with 5% normal goat serum. Then, cells were incubated with primary antibodies against TTC3 (2F6) or MAP2a/b for 12 hours at 4°C followed by incubation with fluorescently labeled secondary antibodies. Fluorescent images were acquired by a Nikon C2 confocal laser microscope. For measurement of total dendritic length, length of every MAP2a/b positive dendrite of single neuron was measured using NeuronJ plugin of ImageJ software.

Surface receptor biotinylation

Primary cultured neurons were washed twice with PBS with 0,5 mM $MgCl_2$ and 1 mM $CaCl_2$ ($PBS^{+/+}$). Surface-localized proteins were biotinylated with 0.25 mg/ml EZ-Link-Sulfo-NHS-SS-Biotin (Thermo Scientific) for 15 min at 4°C and the reaction was quenched by washing neurons with $PBS^{+/+}$ containing 50 mM glycine and 0.5% BSA

and twice with $PBS^{+/+}$. The neurons were lysed with RIPA buffer (50 mM Tris-Cl pH7.4, 150 mM NaCl, 1% NP-40, 1% Sodium Deoxycholate, 0.1% SDS supplemented with complete EDTA-free protease inhibitor cocktail) and 25 μl of streptavidin-conjugated beads (Invitrogen) were added to the 50 μg of total protein and incubated for 2 hours at 4°C. Proteins pulled down by the Streptavidin-conjugated beads were used for western blotting with an antibody against NR2B, GluR1, $GABA_A\beta3$ and GAPDH. Surface-localized NR2B, GluR1 and $GABA_A\beta3$ protein levels are expressed as the percent of those of control neurons.

Polysome gradient analysis

Cultured cortical neurons or mouse cerebral cortex were homogenized with the polysome buffer (10 mM Hepes pH7.4, 150 mM NaCl, 10 mM $MgCl_2$, 1 mM DTT, 50 μg/ml cyclohexamide) supplemented with a complete EDTA-free protease inhibitor cocktail. Homogenates were passed through a 27-gauge needle 10 times and centrifuged at 1000×g for 10 min. The supernatant (S1) was recovered and NP-40 was added to the final concentration of 1.0 % and incubated on ice for 5 min. The S1 fraction was then centrifuged at 14,000×g for 10 min and the supernatant (S2) fraction was recovered. The S2 fraction was loaded onto 20-50% of the linear sucrose density gradient. The gradients were centrifuged at 40,000 rpm for 2 hours at 4°C in a Beckman Instruments with the SW41 rotor. Continuous fractions of 0.57 ml volume were collected using Gradient station (BIOCOMP) and Micro Collector (ATTA) monitoring absorbance at 254 nm with Bio-Mini-UV-Monitor. Proteins in each fraction was precipitated by addition of 100% ethanol and incubated at -30°C for overnight followed by centrifugation at 20,000x*g* for 45 min and the resulting pellets were used for western

blotting with following antibodies: anti-TTC3 (C2), anti-UFM1, anti-RPL26, anti-RS6 and anti-αTubulin antibodies. To further isolate 40S and 60S ribosome enriched fraction, 40S and 60S ribosome fraction from cultured cortical neurons were obtained by sucrose gradient assay as described above and further centrifuged at 150,000xg for 3 hours at 4°C and resulting pellets were used for western blotting.

Isolation of cytosolic and ER-Ribo fractions

Cultured cortical neurons were homogenized with the permeabilization buffer (25 mM Hepes pH7.4, 110 mM KAc, 2.5 mM $MgAC_2$, 1 mM EGTA, 1 mM DTT, 0.05% digitonin and 50 μg/ml cyclohexamide) supplemented with a complete EDTA-free protease inhibitor cocktail and incubated on ice for 10 min. Homogenates were 3,000xg for 5 min and the supernatant (S1) was recovered as pre-cytosolic fraction. The pellet (P1) was washed twice with washing buffer (25 mM Hepes pH 7.4, 2.5 mM $MgAC_2$, 110 mM KAc, 1 mM EGTA, 1 mM DTT, 0.004% digitonin and 50 μg/ml cyclohexamide) supplemented with a complete EDTA-free protease inhibitor cocktail and centrifuged at 3000×*g* for 5 min. The resulting pellet (P2) was resuspended with lysis buffer (25 mM Hepes pH7.5, 400 mM KAc, 15 mM $MgAC_2$, 1% NP-40, 0.5% Sodium Deoxycholate, 1 mM DTT and 50 μg/ml cyclohexamide) supplemented with a complete EDTA-free protease inhibitor cocktail. Both pre-cytosolic fraction and (P2) fraction were then centrifuged at 7,500xg for 10 min. Supernatant from pre-cytosolic fraction and (P2) fraction was used as Cytosolic and ER-Ribo fractions respectively.

Sample preparation for ribosome profiling

Mice primarily cultured cortical neurons were treated with harringtonine (5 μg/ml) for 3 minutes and rapidly lysed in polysome buffer (20mM Tris-Cl, pH 7.5, 150mM NaCl, 5 mM $MgCl_2$, 1 mM DTT and 100 μg/ml cycloheximide) supplemented with 1% TritonX-100 and DNase I 10units/mL (Roche). After centrifugation at 15000x*g* for 10 min, the cleared supernatant was treated with RNase I (Invitrogen, AM2294) (133.3U/A_{260} unit) at 4°C for 45 min. The mono-ribosomes were isolated on 10-50% sucrose gradient by Gradient Station (Biocomp). Collected mono-ribosomes were extracted by Sepasol-RNA I Super G (Nacalai Tesque) to isolate the ribosome-associated mRNA footprints. Libraries were prepared as described previously (5) and sequenced on Illumina Novaseq 6000 platform. Sequencing data were processed following the published protocol from (6) with modifications. After clipping the adaptor sequence from 3' and trimming one nucleotide from 5', sequences of ribosomal RNA were filtered with bowtie. Unaligned reads were retained and aligned to the mouse mm10 canonical transcripts using bowtie. Position of ribosome P-site of the transcriptome-mapped reads were determined as the 12-15th nt from 5' end using custom R scripts.

The above processes were done by the following shell scripts,

```
zcat -k -d < $raw.fastq.gz | fastx_clipper -a CTGTAGGCACCATCAAT -c -n -l 12 -v | fastx_trimmer -f 2 | bowtie --seedlen=23 --un=$non_rrna.fastq $mm10_rrna_index - > /dev/null

bowtie -S --seedlen=23 $mm10_canonical_index $non_rrna.fastq | samtools view -b -S - | bedtools bamtobed -i | cut -f 1,2,3 | sort | custom_psite_position.Rscript
```

Mapped reads ranging from 25 to 31 nt in length were used. According to the reading-frame relative to translation initiation and length of each mapped reads, the read count of each p-site codon were further aggregated as the formula described in (7). After normalization (RPM) to total read count, read depth plot of ribosome p-site along each transcript were generated by custom R script. For metagene analysis, only the CDS not shorter than 150 aa are used and ribosome footprints on initiation (codon 1) were analyzed.

SILAC labeling and sample preparation for MASS analysis

Cultured cortical neurons were plated on 6 well plates in Dubecco's Modified Eagle's Medium (DMEM, Gibco) supplemented with 10% fetal bovine serum as described above. Cultured cortical neurons were then differentially labeled with medium isotope amino acids [0.398 mM $^{13}C_6$-arginine/ 0.798 mM D_4-lysine (Lys4/Arg6) (Cambridge Isotope Laboratories Inc.)] or heavy isotope amino acids [0.398 mM $^{13}C_6{}^{15}N_2$-lysine and 0.798 mM $^{13}C_6{}^{15}N_4$-arginine (Lys8/Arg10) (Cambridge Isotope Laboratories Inc.)] in Neurobasal Media absence of Methionine, Lysine and Arginine (custom-made, Invitrogen) supplemented with 2 mM Methionine, 0.5 mM L-glutamine and Neuro Brew-21 for 12 to 13 days. Lysates from (Lys4/Arg6) labeled LTN1 WT neurons and (Lys8/Arg10) *Ltn1* KO neurons and lysates from (Lys4/Arg6) labeled *Ltn1* KO neurons and (Lys8/Arg10) labeled LTN1 WT neurons were mixed at 1:1 ratio, respectively and used for polysome gradient analysis as described above to obtain 60S ribosome fraction. Proteins in 60S ribosome fraction were precipitated by ethanol and separated by 5-20% Tris-Tricine gel followed by Coomassie Brilliant Blue (CBB) staining. The gel was

separated into 4 regions according to molecular weights and each region was excised and cut into small pieces. The gel pieces were then briefly washed and destained with 50 mM ammonium bicarbonate containing 50% acetonitrile. After dehydration, proteins in gel pieces were treated with 10 mM dithiothreitol (DTT) in 100 mM ammonium bicarbonate to reduce cysteine residues, followed by alkylation with 0.25 M iodoacetamide (IAA) in 100 mM ammonium bicarbonate. After dehydration, proteins in gel pieces were digested with 20 ng/ml Trypsin/Lys-c (Promega) at 37°C for 12 hours. The enzymatically digested protein fragments were extracted from the gel pieces with 50-80% acetonitrile containing 1% trifluoroacetic acid, dried by vacuum centrifugation and applied for LC-MS/MS.

Protein analysis by LC-MS/MS

The enzymatically digested protein fragments were applied to a liquid chromatograph (LC) (EASY-nLC 1000; Thermo Fisher Scientific Inc.) coupled to a Q Exactive hybrid quadrupole-orbitrap mass spectrometer (Thermo Fisher Scientific Inc.) with a nanospray ion source in positive mode. The peptides derived from protein fragments were separated on a Nano high performance LC capillary column C18 (inside diameter: 0.075 mm x length: 150 mm, particle size: 3 μm, Nikkyo Technos Co. Ltd.). The mobile phases “A” and “B” were water with 0.1% formic acid and acetonitrile with 0.1% formic acid, respectively. Two different slopes were used for a 60 min gradient at a flow rate of 300 ml/min: 5 to 35% B in 48 min, and then 35 to 65% in 12 min. The Q Exactive MS was operated in top 10 data-dependent scan mode. The parameters of Q Exactive were as follows: spray voltage (2.3 kV), capillary temperature (275°C), mass

range (350-1800 m/z), normalized collision energy (28%). Raw data were acquired with Xcalibur software.

Protein identification

The MS and MS/MS data were searched against the Swiss-Prot database using Proteome Discoverer (Thermo Fisher Scientific Inc.). The search parameters were as follows: Enzyme: Lys-C protease static modifications, carbamidomethyl (Cys); Dynamic modifications: oxidation (Met); Precursor mass tolerance: ±6 ppm (parts per million); fragment mass tolerance: ±20 mDa; maximum missed cleavages: 1. The proteins were considered identified when their false discovery rates were less than 5%.

Statistical analysis

The statistical significance of the data was examined by *t* test, Kolmogorov-Smirnov test Mann Whitney test, for analyses of two groups ($n \geq 3$ for cell biology data unless otherwise indicated) as indicated in figure legends. For analysis of three or more groups, one-way ANOVA with a Bonferroni's multiple comparison tests were used. For ribosome profiling data, ribosome footprints on initiation (codon 1) were analyzed by Kruskal-Wallis test and pairwise compared by Bonferroni-corrected Wilcoxon-Mann-Whitney test. The *p* values of <0.05 (*), <0.01 (**) and <0.001 (***) were considered to be statistically significant.

REFERENCES

1. H. Yang, H. Wang, R. Jaenisch, Generating genetically modified mice using CRISPR/Cas-mediated genome engineering. *Nat. Prot*. **9**, doi:10.1038/nprot.2014.134 (2014).
2. S. Zolotukhin et al., Recombinant adeno-associated virus purification using novel methods improves infections titer and yield. *Gene Ther*. **6**, 973-985 (1999).
3. R.M.J. Deacon, Assessing nest building in mice. *Nat. Protoc.* **1**, 1117-1119 (2006).
4. N. Takashima N et al., Impaired cognitive function and altered hippocampal synapse morphology in mice lacking Lrrtm1, a gene associated with schizophrenia. *PLoS One.* doi: 10.1371/journal.pone.0022716. (2011).
5. C.W. Chen, M. Tanaka, Genome-wide Translation Profiling by Ribosome-Bound tRNA Capture. *Cell Rep.* **23,** 608-621 (2018).
6. N.T. Ingolia, L.F. Lareau, J.S. Weissman, Ribosome profiling of mouse embryonic stem cells reveals the complexity and dynamics of mammalian proteomes. *Cell* **147**, 789-802. (2011).
7. R. Ishimura et al., Ribosome stalling induced by mutation of a CNS-specific tRNA causes neurodegeneration. *Science* **345**, 455-459 (2014).
