# Supplementary material for: Dysregulation of ribosome-associated quality control elicits cognitive disorders via overaccumulation of TTC3
Source: Proc Natl Acad Sci U S A. 2023 Mar 14;120(12):e2211522120. doi: 10.1073/pnas.2211522120 (PMC10041068; doi:10.1073/pnas.2211522120)
Supplement: Supplementary file 2 — Dataset S01 (PDF) [file pnas.2211522120.sd01.pdf]

Dataset S1. Translation initiation of selective mRNAs was affected by the deletion of LTN1

A list of ranked genes sorted by the difference in footprint number at translation initiation site between harringtonine treated WT and *Ltn1* KO neurons (*Ltn1* KO-WT).

ID	(*Ltn1* KO - WT)	MGI.Gene/Marker.ID	Gene Symbol
ENSMUST00000034756.14	-2.385549114	MGI:88246	Anxa2
ENSMUST00000048184.3	-2.308250021	MGI:1859170	Pdcd7
ENSMUST00000107376.7	-2.22657363	MGI:1919216	Nkiras2
ENSMUST00000023362.14	-2.183933329	MGI:108471	Ntan1
ENSMUST00000077485.10	-2.139994267	MGI:99157	Zfp12
ENSMUST00000067444.9	-2.009807435	MGI:95697	Gfap
ENSMUST00000022115.13	-1.999527654	MGI:1333799	Xrcc4
ENSMUST00000022705.6	-1.94949253	MGI:1914631	Med4
ENSMUST00000123251.8	-1.897659563	MGI:96925	Mbp
ENSMUST00000204930.2	-1.897659563	MGI:1918910	Rarres2
ENSMUST00000044567.3	-1.843894724	MGI:1913498	Alg5
ENSMUST00000183425.7	-1.788048413	MGI:2446108	Hmgcll1
ENSMUST00000229696.1	-1.788048413	MGI:98509	Tfcp2
ENSMUST00000021810.2	-1.729952952	MGI:99414	Id4
ENSMUST00000028199.11	-1.729952952	MGI:1353605	Tor1b
ENSMUST00000216152.1	-1.729952952	MGI:1919093	R3hcc1
ENSMUST00000003154.6	-1.669419546	MGI:102707	Efna2
ENSMUST00000033800.12	-1.669419546	MGI:97623	Plp1
ENSMUST00000050630.13	-1.669419546	MGI:1923723	Supt3
ENSMUST00000027157.9	-1.606234583	MGI:1913896	Rpe
ENSMUST00000030317.13	-1.606234583	MGI:103098	Pdpn
ENSMUST00000037099.8	-1.606234583	MGI:1352754	Clic4
ENSMUST00000057497.12	-1.606234583	MGI:2155345	Col26a1
ENSMUST00000059562.13	-1.606234583	MGI:1920048	Lhfp
ENSMUST00000031122.8	-1.584823034	MGI:95619	Gabrb1
ENSMUST00000202447.3	-1.552854943	MGI:1924223	Fbxo21
ENSMUST00000013886.8	-1.540155087	MGI:1924258	Ppp1r12c
ENSMUST00000022586.1	-1.540155087	MGI:1351474	Nufip1
ENSMUST00000029649.2	-1.540155087	MGI:2139628	Ctso
ENSMUST00000038626.9	-1.540155087	MGI:2681846	Mpv17l2
ENSMUST00000113102.9	-1.540155087	MGI:1914361	Naaa
ENSMUST00000108634.8	-1.521950517	MGI:2681835	Nlgn2

ENSMUST00000034989.14	-1.511728424	MGI:97043	Me1
ENSMUST00000097822.9	-1.49640751	MGI:1916366	Ubr4
ENSMUST00000055104.5	-1.479906682	MGI:2385317	Tceal1
ENSMUST00000021903.2	-1.470903073	MGI:1346325	Gadd45g
ENSMUST00000035222.5	-1.470903073	MGI:1928738	Slc25a20
ENSMUST00000043722.9	-1.470903073	MGI:99554	Lgals3bp
ENSMUST00000048642.14	-1.470903073	MGI:1277152	Parl
ENSMUST00000056370.12	-1.470903073	MGI:1914287	Pmf1
ENSMUST00000060447.12	-1.470903073	MGI:1922672	Mettl5
ENSMUST00000037827.9	-1.459729871	MGI:2653235	Slc45a1
ENSMUST00000048073.8	-1.433075272	MGI:2687325	Pigs
ENSMUST00000003501.8	-1.430189623	MGI:109157	Elavl3
ENSMUST00000029907.5	-1.410090644	MGI:1915303	Ubxn2b
ENSMUST00000045301.8	-1.401936759	MGI:107502	H1f3
ENSMUST00000020023.8	-1.398158481	MGI:88930	Reep3
ENSMUST00000020849.8	-1.398158481	MGI:1919193	Tom1l1
ENSMUST00000024957.6	-1.398158481	MGI:99462	Pigf
ENSMUST00000061890.7	-1.398158481	MGI:2141413	Zfp282
ENSMUST00000101426.10	-1.398158481	MGI:1913308	Tmem176a
ENSMUST00000125544.2	-1.398158481	MGI:5141896	Gm20431
ENSMUST00000129635.7	-1.398158481	MGI:1891341	Lpin2
ENSMUST00000161211.7	-1.398158481	MGI:1917042	Med6
ENSMUST00000011981.4	-1.386796361	MGI:1914861	Snapc2
ENSMUST00000034015.10	-1.3742772	MGI:1913484	Msmo1
ENSMUST00000026142.7	-1.340847245	MGI:1930187	Maged1
ENSMUST00000033770.12	-1.330365505	MGI:99918	Mecp2
ENSMUST00000025217.10	-1.328736592	MGI:96245	Hspa9
ENSMUST00000025739.13	-1.328700899	MGI:1923718	Uhrf2
ENSMUST00000042546.3	-1.328700899	MGI:2143103	Ano10
ENSMUST00000005431.5	-1.326740663	MGI:1932557	Letm1
ENSMUST00000026434.12	-1.321550231	MGI:3583942	Chmp6
ENSMUST00000186733.6	-1.321550231	MGI:2446126	Phf3
ENSMUST00000004375.15	-1.295693639	MGI:102520	Phb2
ENSMUST00000030367.14	-1.274519194	MGI:1919725	Ssbp3
ENSMUST00000048653.9	-1.268167493	MGI:2387578	Cpne2
ENSMUST00000020922.7	-1.254277519	MGI:2145021	Trib2
ENSMUST00000040344.6	-1.243056202	MGI:1922862	Gns
ENSMUST00000032840.4	-1.24257354	MGI:1915244	Mrps11
ENSMUST00000000939.14	-1.240644771	MGI:1352504	Hip1r

ENSMUST00000025161.9	-1.240644771	MGI:1201689	Tapbp
ENSMUST00000035094.13	-1.240644771	MGI:2143333	Exog
ENSMUST00000060188.13	-1.240644771	MGI:1914099	Ppp1r2
ENSMUST00000064848.6	-1.240644771	MGI:2143854	Nup107
ENSMUST00000095759.4	-1.240644771	MGI:99252	Egr4
ENSMUST00000115168.8	-1.240644771	MGI:1914244	Cep19
ENSMUST00000192512.5	-1.240644771	MGI:1298406	Pcdha4
ENSMUST00000212075.1	-1.234647324	MGI:1925350	Msantd4
ENSMUST00000124482.2	-1.219422851	MGI:1351608	Mrpl17
ENSMUST00000084125.9	-1.208892932	MGI:2387629	Tardbp
ENSMUST00000015137.9	-1.203196435	MGI:104572	Limk1
ENSMUST00000057503.6	-1.203196435	MGI:1923411	Lamp5
ENSMUST00000042312.13	-1.194862069	MGI:1923551	Trafd1
ENSMUST00000102735.9	-1.193664326	MGI:2150150	Slc4a10
ENSMUST00000103193.4	-1.193664326	MGI:96622	Itpa
ENSMUST00000223553.1	-1.191409153	MGI:97587	Pim2
ENSMUST00000001304.8	-1.186318214	MGI:88407	Ckb
ENSMUST00000067530.5	-1.167812375	MGI:1928744	Vapb
ENSMUST00000038791.14	-1.157570585	MGI:1891827	Gde1
ENSMUST00000090227.5	-1.157407879	MGI:2442773	Marchf6
ENSMUST00000043966.7	-1.156407084	MGI:1921850	Mrpl47
ENSMUST00000011029.11	-1.154931211	MGI:1914963	Dnajc19
ENSMUST00000017090.5	-1.154931211	MGI:1916509	Kctd5
ENSMUST00000020040.4	-1.154931211	MGI:1328351	Nts
ENSMUST00000022272.13	-1.154931211	MGI:1918643	Kctd6
ENSMUST00000030432.7	-1.154931211	MGI:96158	Hmgcl
ENSMUST00000041558.14	-1.154931211	MGI:1276124	Ep400
ENSMUST00000052529.3	-1.154931211	MGI:2444211	Ppp1r15b
ENSMUST00000103194.9	-1.154931211	MGI:1096574	Car4
ENSMUST00000109943.9	-1.154931211	MGI:1921552	Mtmr3
ENSMUST00000119664.1	-1.154931211	MGI:1916796	Mapk1ip1
ENSMUST00000020856.5	-1.149784132	MGI:1914162	Bzw2
ENSMUST00000028222.12	-1.139171609	MGI:95835	Hspa5
ENSMUST00000024887.5	-1.139139415	MGI:1920944	Ndufaf7
ENSMUST00000032362.11	-1.138903035	MGI:1889679	Slco1c1
ENSMUST00000077798.12	-1.138903035	MGI:107912	Ssrp1
ENSMUST00000081591.6	-1.138903035	MGI:1919747	Zc3h15
ENSMUST00000007797.9	-1.136618121	MGI:95620	Gabrb2
ENSMUST00000023189.14	-1.132214268	MGI:1921272	Glyr1

ENSMUST00000015011.9	-1.129049518	MGI:98445	Surf4
ENSMUST00000022296.6	-1.124039057	MGI:107411	Ube2e1
ENSMUST00000013970.8	-1.122378725	MGI:2152214	Pip4k2c
ENSMUST00000022563.8	-1.122378725	MGI:2145637	Mtmr6
ENSMUST00000211115.1	-1.121857464	MGI:107739	Casp3
ENSMUST00000003450.14	-1.11866456	MGI:1921601	Ddx23
ENSMUST00000114989.8	-1.108049254	MGI:1859026	Cnot4
ENSMUST00000237400.1	-1.104000291	MGI:1917473	Nars
ENSMUST00000114516.7	-1.099782578	MGI:1917433	Dzip3
ENSMUST00000102926.4	-1.094140059	MGI:1914878	Anp32b
ENSMUST00000032419.8	-1.092705364	MGI:1337124	Cmas
ENSMUST00000108904.9	-1.092705364	MGI:894291	Acsl6
ENSMUST00000161154.1	-1.090709216	MGI:104630	Wars
ENSMUST00000030361.10	-1.082487864	MGI:1929278	Tmem59
ENSMUST00000026635.7	-1.073203208	MGI:2446173	Farp1
ENSMUST00000114007.7	-1.073203208	MGI:1924317	Cacfd1
ENSMUST00000039534.10	-1.069651021	MGI:1098222	Resp18
ENSMUST00000041725.13	-1.069651021	MGI:2143974	Mgat4b
ENSMUST00000043325.8	-1.069651021	MGI:1346049	Hs2st1
ENSMUST00000022993.6	-1.066187131	MGI:1915069	Derl1
ENSMUST00000005810.8	-1.063801766	MGI:1338850	Mthfd2
ENSMUST00000009435.11	-1.063801766	MGI:2652132	Pttg1ip
ENSMUST00000018851.13	-1.063801766	MGI:103147	Dync1h1
ENSMUST00000022345.6	-1.063801766	MGI:1915326	Dnajc9
ENSMUST00000024967.13	-1.063801766	MGI:101816	Msh2
ENSMUST00000027404.11	-1.063801766	MGI:102765	Ptprn
ENSMUST00000029752.14	-1.063801766	MGI:1919057	Tars2
ENSMUST00000034227.5	-1.063801766	MGI:1915051	Pllp
ENSMUST00000037360.7	-1.063801766	MGI:2444227	Rhov
ENSMUST00000049503.9	-1.063801766	MGI:1926048	Eml4
ENSMUST00000051605.8	-1.063801766	MGI:1346051	Dut
ENSMUST00000054399.5	-1.063801766	MGI:1349481	Ing1
ENSMUST00000068031.7	-1.063801766	MGI:98790	Top2a
ENSMUST00000095938.9	-1.063801766	MGI:2141510	AI854703
ENSMUST00000114471.2	-1.063801766	MGI:2146430	Cblb
ENSMUST00000115661.4	-1.063801766	MGI:5649001	Gm42416
ENSMUST00000207505.1	-1.063801766	MGI:5011595	Gm19410
ENSMUST00000107237.7	-1.063557736	MGI:1201670	Psmd4
ENSMUST00000029727.7	-1.058014129	MGI:1354695	Fbxw7

ENSMUST00000071816.6	-1.057507763	MGI:88259	Camk2g
ENSMUST00000056882.6	-1.051723684	MGI:1355334	Olig1
ENSMUST00000004378.14	-1.048280338	MGI:95394	Eno2
ENSMUST00000013766.12	-1.045886895	MGI:1918918	Atraid
ENSMUST00000027111.14	-1.038031395	MGI:2443241	Ndufs1
ENSMUST00000219993.1	-1.038031395	MGI:1917754	Galnt16
ENSMUST00000019616.5	-1.026625868	MGI:109430	Icam5
ENSMUST00000021514.9	-1.026625868	MGI:99878	Ppm1a
ENSMUST00000072186.11	-1.026625868	MGI:104626	Strbp
ENSMUST00000028926.12	-1.022088162	MGI:104562	Napb
ENSMUST00000021018.10	-1.018494938	MGI:1917689	Taf15
ENSMUST00000006254.5	-1.015107747	MGI:1913661	Tbcb
ENSMUST00000020118.4	-1.015107747	MGI:1914853	Dusp6
ENSMUST00000165443.3	-1.015107747	MGI:1351502	Nup50
ENSMUST00000043584.4	-1.014669765	MGI:1915472	Tubb4b
ENSMUST00000005606.7	-1.011281752	MGI:97592	Prkaca
ENSMUST00000048016.2	-1.0084265	MGI:1920318	Fut11
ENSMUST00000109381.8	-1.0084265	MGI:1924161	Brd1
ENSMUST00000051431.9	-1.006181162	MGI:1261890	Fbxo28
ENSMUST00000115567.7	-1.006181162	MGI:95824	Nr3c1
ENSMUST00000026576.4	-1.003957103	MGI:1274789	Taldo1
ENSMUST00000072740.12	-0.999059803	MGI:107771	Abr
ENSMUST00000040518.5	-0.997057927	MGI:2386251	Eif3l
ENSMUST00000029116.13	-0.996906428	MGI:1923927	Pcmtd2
ENSMUST00000114763.2	-0.988538721	MGI:102793	Fstl1
ENSMUST00000166734.9	-0.985342838	MGI:1923749	Clasp2
ENSMUST00000023444.10	-0.982185085	MGI:1914113	Lztr1
ENSMUST00000086978.11	-0.982185085	MGI:1858227	Cdkl2
ENSMUST00000031004.10	-0.977800315	MGI:107793	Crmp1
ENSMUST00000103017.3	-0.973175507	MGI:2679787	Nploc4
ENSMUST00000002840.8	-0.967093505	MGI:2385054	Pex6
ENSMUST00000030381.7	-0.967093505	MGI:1858304	Ctps
ENSMUST00000030731.10	-0.96652558	MGI:1913714	Taf12
ENSMUST00000037141.8	-0.96652558	MGI:1920501	Setd7
ENSMUST00000046463.9	-0.96652558	MGI:109443	Gtpbp1
ENSMUST00000055125.4	-0.96652558	MGI:2183442	Diras1
ENSMUST00000065111.14	-0.96652558	MGI:101850	Usp45
ENSMUST00000070173.8	-0.96652558	MGI:1913772	Pgpep1
ENSMUST00000071703.5	-0.96652558	MGI:2387179	Tut7

ENSMUST00000087333.8	-0.96652558	MGI:2148020	Tmem164
ENSMUST00000103006.3	-0.96652558	MGI:2147931	Nudt10
ENSMUST00000108023.9	-0.96652558	MGI:88316	Ccne1
ENSMUST00000108702.7	-0.96652558	MGI:97631	Pmp22
ENSMUST00000105447.10	-0.962711544	MGI:1353654	Vps26a
ENSMUST00000051454.3	-0.96115085	MGI:2444579	Fam171b
ENSMUST00000095737.4	-0.96115085	MGI:3050566	Akap6
ENSMUST00000103079.3	-0.96115085	MGI:99695	Cttn
ENSMUST00000109608.8	-0.96115085	MGI:2386621	Cpne1
ENSMUST00000077290.8	-0.960949875	MGI:104864	Pdia4
ENSMUST00000037299.14	-0.957373854	MGI:1917431	Ythdf1
ENSMUST00000069041.14	-0.957373854	MGI:1889383	Ap1s2
ENSMUST00000106391.7	-0.95457434	MGI:1915013	Prpsap1
ENSMUST00000118466.7	-0.95457434	MGI:1917814	Prxl2a
ENSMUST00000191471.6	-0.95457434	MGI:1915671	Lmbrd1
ENSMUST00000179353.7	-0.952416323	MGI:97298	Septin2
ENSMUST00000179353.7	-0.952416323	MGI:97298	Septin2
ENSMUST00000179353.7	-0.952416323	MGI:97298	Septin2
ENSMUST00000179353.7	-0.952416323	MGI:97298	Septin2
ENSMUST00000023509.4	-0.949307222	MGI:1923035	Klhl24
ENSMUST00000014370.10	-0.948908186	MGI:1270839	Cacybp
ENSMUST00000058109.8	-0.947175199	MGI:1354367	Mrps7
ENSMUST00000057921.9	-0.930782783	MGI:99595	Arf2
ENSMUST00000031445.4	-0.928520642	MGI:1926144	Aacs
ENSMUST00000114874.4	-0.927926314	MGI:2141402	Clec2l
ENSMUST00000045692.8	-0.926386156	MGI:2448488	Fbxl16
ENSMUST00000018274.9	-0.925363452	MGI:1355272	Csnk1d
ENSMUST00000060991.5	-0.925363452	MGI:1914375	Tspan31
ENSMUST00000167201.1	-0.925363452	MGI:1929266	Snd1
ENSMUST00000021201.5	-0.924089623	MGI:107265	Cpd
ENSMUST00000165307.7	-0.922459226	MGI:1913721	Anp32e
ENSMUST00000030626.11	-0.922086054	MGI:1919067	Tmem50a
ENSMUST00000035105.6	-0.920298178	MGI:105381	Rpsa
ENSMUST00000022808.13	-0.917297054	MGI:1859158	Pabpn1
ENSMUST00000030110.14	-0.917297054	MGI:1915518	Zdhhc21
ENSMUST00000044166.8	-0.917297054	MGI:1918963	Cdc40
ENSMUST00000075444.7	-0.917297054	MGI:108101	Ddn
ENSMUST00000162819.8	-0.917297054	MGI:1923825	Wdr26
ENSMUST00000217198.1	-0.917297054	MGI:3045350	Olfm2

ENSMUST00000059229.15	-0.91664403	MGI:1919792	Pgam5
ENSMUST00000004134.10	-0.914090298	MGI:1309466	Gstm5
ENSMUST00000175858.8	-0.91270999	MGI:1920590	Nptxr
ENSMUST00000026222.10	-0.906917632	MGI:1914514	Ndufb8
ENSMUST00000059339.5	-0.900666848	MGI:105308	Pnoc
ENSMUST00000019229.14	-0.896570013	MGI:1915269	Med8
ENSMUST00000033763.14	-0.896570013	MGI:1915255	Naa10
ENSMUST00000042052.8	-0.896570013	MGI:2384768	Hectd1
ENSMUST00000026156.7	-0.891389378	MGI:894275	Rfng
ENSMUST00000053355.5	-0.891389378	MGI:1928333	Creg2
ENSMUST00000088744.11	-0.891389378	MGI:1340046	Adam22
ENSMUST00000021595.9	-0.887666042	MGI:106054	Psmc1
ENSMUST00000199300.4	-0.886428876	MGI:1341265	Camk2d
ENSMUST00000166972.8	-0.88496027	MGI:101921	Ap2a1
ENSMUST00000034689.7	-0.882713849	MGI:1346036	Pin1
ENSMUST00000033720.11	-0.882396648	MGI:1194910	Rbbp7
ENSMUST00000074371.12	-0.882195093	MGI:107490	Pdcd4
ENSMUST00000036033.13	-0.879029382	MGI:2385920	Dip2a
ENSMUST00000022009.9	-0.877376241	MGI:1097706	Cetn3
ENSMUST00000071555.12	-0.876986512	MGI:87906	Actg1
ENSMUST00000027503.13	-0.876405495	MGI:108396	Dtymk
ENSMUST00000048934.14	-0.875437291	MGI:107404	Tbr1
ENSMUST00000062058.4	-0.875437291	MGI:3036267	Lix1l
ENSMUST00000066983.12	-0.875437291	MGI:2442422	Abhd10
ENSMUST00000075190.4	-0.875437291	MGI:99217	Cdh11
ENSMUST00000077672.11	-0.875437291	MGI:1915268	Col4a3bp
ENSMUST00000105439.1	-0.875437291	MGI:2389177	Lrrtm3
ENSMUST00000203619.2	-0.875437291	MGI:99534	Cntn3
ENSMUST00000030637.13	-0.874794563	MGI:1347351	Ncdn
ENSMUST00000029770.7	-0.871598614	MGI:1349216	Abcd3
ENSMUST00000015950.11	-0.869876106	MGI:97836	Qdpr
ENSMUST00000096057.4	-0.868897992	MGI:1926784	Tagln3
ENSMUST00000023489.10	-0.864564567	MGI:1917955	Fyttd1
ENSMUST00000018315.9	-0.863556217	MGI:1923159	Vmp1
ENSMUST00000030296.8	-0.863556217	MGI:1913323	Txndc12
ENSMUST00000047714.13	-0.863556217	MGI:1922855	Kdm5b
ENSMUST00000078945.11	-0.863556217	MGI:106479	Grsf1
ENSMUST00000006037.12	-0.862213087	MGI:1276533	Ncoa2
ENSMUST00000009102.8	-0.862213087	MGI:1202305	Vps72

ENSMUST00000018212.12	-0.862213087	MGI:1917672	Ints2
ENSMUST00000028389.3	-0.862213087	MGI:892032	Frzb
ENSMUST00000029316.15	-0.862213087	MGI:1916889	Exosc8
ENSMUST00000029588.9	-0.862213087	MGI:107634	Larp7
ENSMUST00000030311.10	-0.862213087	MGI:1914944	Ift74
ENSMUST00000034981.13	-0.862213087	MGI:891964	Xrn1
ENSMUST00000035264.8	-0.862213087	MGI:1920334	Pak7
ENSMUST00000037709.15	-0.862213087	MGI:1914873	Tm7sf3
ENSMUST00000038665.5	-0.862213087	MGI:2685233	Ptchd1
ENSMUST00000039812.15	-0.862213087	MGI:3041170	Zmym5
ENSMUST00000040986.14	-0.862213087	MGI:1915160	Rubcn
ENSMUST00000045243.14	-0.862213087	MGI:1891434	Pde4dip
ENSMUST00000053855.7	-0.862213087	MGI:1920534	Ddit4l
ENSMUST00000057495.9	-0.862213087	MGI:1919995	Tmem161b
ENSMUST00000064314.9	-0.862213087	MGI:1928379	Asna1
ENSMUST00000065537.8	-0.862213087	MGI:1913096	Jmy
ENSMUST00000102994.9	-0.862213087	MGI:1914740	Ufl1
ENSMUST00000103084.3	-0.862213087	MGI:2388656	Zfp334
ENSMUST00000110855.7	-0.862213087	MGI:2446186	Lpgat1
ENSMUST00000126319.7	-0.862213087	MGI:2384863	Tbl3
ENSMUST00000192511.1	-0.862213087	MGI:1935219	Pcdhga7
ENSMUST00000222435.1	-0.862213087	MGI:3779109	Ftl1-ps1
ENSMUST00000237602.1	-0.862213087	MGI:1926421	Tcerg1
ENSMUST00000010007.8	-0.86069199	MGI:1914930	Sdhb
ENSMUST00000004054.12	-0.857198439	MGI:103560	Kpna1
ENSMUST00000028683.13	-0.85649581	MGI:95834	Pdia3
ENSMUST00000021532.5	-0.85578876	MGI:1922877	Snapc1
ENSMUST00000026750.14	-0.853207212	MGI:2661175	Cnksr2
ENSMUST00000043484.7	-0.853207212	MGI:2385070	Reep2
ENSMUST00000033189.5	-0.848833881	MGI:99479	Cckbr
ENSMUST00000200659.1	-0.848833881	MGI:5663875	Gm43738
ENSMUST00000006435.7	-0.842285373	MGI:109618	Atp6v1b2
ENSMUST00000052332.14	-0.841728481	MGI:106913	Abi2
ENSMUST00000032497.6	-0.839392718	MGI:107893	D6Wsu163e
ENSMUST00000058496.7	-0.839392718	MGI:1914490	Taok1
ENSMUST00000089559.10	-0.839392718	MGI:1924256	Ddrgk1
ENSMUST00000103140.4	-0.839392718	MGI:1930751	Trpc4ap
ENSMUST00000112636.7	-0.839392718	MGI:2139447	Galnt13
ENSMUST00000113403.7	-0.839392718	MGI:99654	Epha5

ENSMUST00000033036.6	-0.838474551	MGI:1354952	Dkk3
ENSMUST00000034051.6	-0.838474551	MGI:1913679	Ufsp2
ENSMUST00000057288.5	-0.837966838	MGI:1919103	Pdia6
ENSMUST00000113677.2	-0.837966838	MGI:2442511	Zer1
ENSMUST00000062045.3	-0.834757749	MGI:1931527	H1f4
ENSMUST00000027131.5	-0.83070291	MGI:1914515	Slc39a10
ENSMUST00000091064.7	-0.83070291	MGI:1915691	Rraga
ENSMUST00000060500.8	-0.827993201	MGI:1933735	Eif5a2
ENSMUST00000239150.1	-0.827993201	MGI:98970	Xbp1
ENSMUST00000035220.11	-0.825309882	MGI:108025	Prkar2a
ENSMUST00000074127.13	-0.825309882	MGI:1353452	Aldh3a2
ENSMUST00000107172.7	-0.825309882	MGI:1919599	Dusp3
ENSMUST00000118557.7	-0.825309882	MGI:1338882	Pex11b
ENSMUST00000030117.4	-0.822997622	MGI:1915546	Smu1
ENSMUST00000034612.6	-0.822997622	MGI:1353582	Ddx25
ENSMUST00000001818.4	-0.820020868	MGI:1914127	Crnkl1
ENSMUST00000003320.13	-0.820020868	MGI:2446176	Eif2b5
ENSMUST00000037337.9	-0.820020868	MGI:1914499	Tbc1d19
ENSMUST00000089250.8	-0.820020868	MGI:104908	Ptk2b
ENSMUST00000102801.7	-0.820020868	MGI:1345184	Tenm2
ENSMUST00000066469.13	-0.818819963	MGI:1891702	Cope
ENSMUST00000048112.12	-0.815752303	MGI:107320	Sgsm1
ENSMUST00000236085.1	-0.814071614	MGI:1929601	Ndfip1
ENSMUST00000041623.8	-0.811670045	MGI:109610	Enc1
ENSMUST00000114297.4	-0.809213343	MGI:2141658	Vopp1
ENSMUST00000095664.5	-0.808840609	MGI:2684999	Tmf1
ENSMUST00000019722.11	-0.808021011	MGI:1913780	Ubxn6
ENSMUST00000022765.13	-0.803609621	MGI:1923588	Rab2b
ENSMUST00000005923.6	-0.803531617	MGI:1098257	Psmb4
ENSMUST00000049285.9	-0.801939676	MGI:106038	Lrrn1
ENSMUST00000109594.7	-0.801939676	MGI:2135601	Slc1a4
ENSMUST00000025546.16	-0.800371254	MGI:1913304	Cndp2
ENSMUST00000030734.4	-0.800371254	MGI:1922312	Sf3a3
ENSMUST00000001836.10	-0.799512727	MGI:1914735	Pwp1
ENSMUST00000080210.9	-0.798725126	MGI:101931	Slc1a2
ENSMUST00000003759.10	-0.796217173	MGI:1346998	Ciao1
ENSMUST00000029780.11	-0.796217173	MGI:1860489	Ptbp2
ENSMUST00000023225.7	-0.79548633	MGI:1913418	Grina
ENSMUST00000036381.9	-0.795069043	MGI:1913922	Chmp7

ENSMUST00000120638.7	-0.791177487	MGI:1351334	Syn3
ENSMUST00000009538.11	-0.785847687	MGI:103020	Syn2
ENSMUST00000025218.7	-0.784307845	MGI:2385071	Etf1
ENSMUST00000032335.12	-0.784307845	MGI:1858965	Atf7ip
ENSMUST00000087654.4	-0.784307845	MGI:1920701	Zfp763
ENSMUST00000094331.4	-0.784307845	MGI:1913897	Nsmce3
ENSMUST00000110534.7	-0.784307845	MGI:2137512	Arid4b
ENSMUST00000111742.7	-0.784307845	MGI:104861	Bcat1
ENSMUST00000121369.7	-0.784307845	MGI:2442859	Rnft2
ENSMUST00000156249.1	-0.784307845	MGI:98889	Ubc
ENSMUST00000004202.16	-0.780462619	MGI:94912	Dnmt1
ENSMUST00000007803.11	-0.780462619	MGI:88139	Bcl2l1
ENSMUST00000058265.7	-0.780462619	MGI:1913493	C1galt1c1
ENSMUST00000164930.7	-0.780462619	MGI:97178	Map4
ENSMUST00000216063.1	-0.780462619	MGI:2143340	Zfp445
ENSMUST00000004050.6	-0.778947905	MGI:1914718	Mmd
ENSMUST00000039894.12	-0.777941145	MGI:1917680	Tbce
ENSMUST00000049093.7	-0.777941145	MGI:1889549	Txnip
ENSMUST00000020754.9	-0.776160234	MGI:1913994	Cfap36
ENSMUST00000030074.7	-0.776160234	MGI:1332243	Ugcg
ENSMUST00000020886.8	-0.774835404	MGI:1929865	Nampt
ENSMUST00000089770.10	-0.774835404	MGI:1923760	Trappc9
ENSMUST00000182659.7	-0.774835404	MGI:1933204	Rbm5
ENSMUST00000222941.1	-0.774835404	MGI:1924730	Kidins220
ENSMUST00000033915.8	-0.764492697	MGI:107671	Gpm6a
ENSMUST00000027425.15	-0.763659415	MGI:1927594	Itm2c
ENSMUST00000033176.6	-0.762518144	MGI:1914253	Uqcrc2
ENSMUST00000049053.8	-0.758844838	MGI:2442372	Fam168a
ENSMUST00000055242.10	-0.758805485	MGI:1927155	Clptm1
ENSMUST00000146292.7	-0.757656842	MGI:2444386	Tmem63c
ENSMUST00000160289.8	-0.757656842	MGI:1915122	Nsmce4a
ENSMUST00000025477.14	-0.756057868	MGI:106019	St8sia3
ENSMUST00000038163.7	-0.756057868	MGI:1918941	Pnmal1
ENSMUST00000066668.13	-0.756057868	MGI:1278328	Dnpep
ENSMUST00000070533.4	-0.756057868	MGI:3528744	Xkr4
ENSMUST00000110708.3	-0.756057868	MGI:3714357	Srp54b
ENSMUST00000119374.7	-0.756057868	MGI:2444830	Nkain3
ENSMUST00000121469.1	-0.756057868	MGI:1098827	Reep1
ENSMUST00000171649.7	-0.756057868	MGI:1298392	Bscl2

ENSMUST00000160835.8	-0.754002929	MGI:2448501	Pip4p1
ENSMUST00000003318.12	-0.753679158	MGI:108100	Dvl3
ENSMUST00000031278.5	-0.753679158	MGI:88179	Bmp3
ENSMUST00000038014.10	-0.753679158	MGI:2442146	Dnajc16
ENSMUST00000046110.15	-0.753679158	MGI:1098567	Astn1
ENSMUST00000055454.13	-0.753679158	MGI:1922460	Prr3
ENSMUST00000071095.13	-0.753679158	MGI:1289184	Setd3
ENSMUST00000087879.10	-0.753679158	MGI:2148050	Nexmif
ENSMUST00000020049.8	-0.749766444	MGI:1289302	Ccdc59
ENSMUST00000020258.9	-0.749766444	MGI:1914595	Herc4
ENSMUST00000020268.6	-0.749766444	MGI:1914750	Ccar1
ENSMUST00000020931.5	-0.749766444	MGI:1914491	Smc6
ENSMUST00000020969.4	-0.749766444	MGI:99830	Cmpk2
ENSMUST00000021285.13	-0.749766444	MGI:1890156	Stx8
ENSMUST00000022709.5	-0.749766444	MGI:1345138	Spry2
ENSMUST00000025702.13	-0.749766444	MGI:1916274	Snx15
ENSMUST00000026831.13	-0.749766444	MGI:2384891	Rhbdl1
ENSMUST00000031607.6	-0.749766444	MGI:1352744	Dtx1
ENSMUST00000032492.8	-0.749766444	MGI:88348	Cd9
ENSMUST00000037923.4	-0.749766444	MGI:3610364	Rbm24
ENSMUST00000050078.12	-0.749766444	MGI:108079	Sdf4
ENSMUST00000061260.7	-0.749766444	MGI:3045256	Fat4
ENSMUST00000065211.8	-0.749766444	MGI:1351636	Srr
ENSMUST00000068387.10	-0.749766444	MGI:1276116	Ep300
ENSMUST00000069098.6	-0.749766444	MGI:2444575	Soga1
ENSMUST00000070864.13	-0.749766444	MGI:88296	Cacna1b
ENSMUST00000076383.7	-0.749766444	MGI:2144023	Fbxw11
ENSMUST00000076921.6	-0.749766444	MGI:1917567	Arl16
ENSMUST00000081497.12	-0.749766444	MGI:2151221	Pop5
ENSMUST00000111063.7	-0.749766444	MGI:1924265	Mpped2
ENSMUST00000153488.8	-0.749766444	MGI:1922259	Naa30
ENSMUST00000159645.7	-0.749766444	MGI:2445220	Dcaf1
ENSMUST00000113913.7	-0.744605958	MGI:107745	Dctn1
ENSMUST00000163666.2	-0.744605958	MGI:95303	Eif4a1
ENSMUST00000019911.13	-0.742933973	MGI:1097691	Hdac2
ENSMUST00000052885.13	-0.739222732	MGI:2177957	Tmem259
ENSMUST00000148178.7	-0.739222732	MGI:1926465	Hnrnpm
ENSMUST00000034834.15	-0.735622498	MGI:97591	Pkm
ENSMUST00000065504.16	-0.735081467	MGI:88031	Anxa7

ENSMUST00000106054.2	-0.734291759	MGI:2147627	Yars
ENSMUST00000018990.7	-0.732535151	MGI:2387464	Pank3
ENSMUST00000021329.13	-0.732395223	MGI:1927204	Gosr2
ENSMUST00000034458.8	-0.732395223	MGI:894694	Galnt2
ENSMUST00000029654.14	-0.72993658	MGI:95751	Glrb
ENSMUST00000165125.7	-0.728744419	MGI:1913491	Tmem9
ENSMUST00000055858.13	-0.728046053	MGI:1914226	2410002F23Rik
ENSMUST00000081111.13	-0.728046053	MGI:109367	Impdh2
ENSMUST00000088392.8	-0.727115551	MGI:1201408	Srpk2
ENSMUST00000111566.8	-0.727115551	MGI:1928401	Clip1
ENSMUST00000163119.7	-0.727115551	MGI:1347360	Cul3
ENSMUST00000165594.3	-0.727115551	MGI:1335094	Septin7
ENSMUST00000019517.9	-0.725815364	MGI:1349409	Cops3
ENSMUST00000229206.1	-0.724906016	MGI:96617	Cd47
ENSMUST00000032398.14	-0.722395395	MGI:1277973	Thumpd3
ENSMUST00000115242.8	-0.722395395	MGI:1921590	Ahcyl2
ENSMUST00000162097.7	-0.722395395	MGI:107562	Arpp21
ENSMUST00000066070.6	-0.720050125	MGI:1921080	Eif3k
ENSMUST00000169032.7	-0.717807493	MGI:96413	Idh1
ENSMUST00000003468.9	-0.715708376	MGI:95818	Grik5
ENSMUST00000021929.9	-0.715708376	MGI:1891713	Habp4
ENSMUST00000024123.8	-0.715708376	MGI:2183446	Agap3
ENSMUST00000133181.1	-0.715708376	MGI:1098815	Cdc23
ENSMUST00000168129.9	-0.715708376	MGI:2445089	Trappc12
ENSMUST00000025027.9	-0.714756182	MGI:1914925	Cuta
ENSMUST00000020027.10	-0.713758727	MGI:1926228	Serinc1
ENSMUST00000025567.8	-0.713605772	MGI:1930079	Fads2
ENSMUST00000043269.13	-0.711858879	MGI:99894	Hnrnpk
ENSMUST00000150727.7	-0.709499752	MGI:2384997	Ube2e2
ENSMUST00000106398.8	-0.706972518	MGI:2442484	Rnf157
ENSMUST00000037205.10	-0.70416529	MGI:1920974	Mcee
ENSMUST00000002529.6	-0.703854369	MGI:1922946	Prpf6
ENSMUST00000003550.10	-0.703854369	MGI:1891700	Ncstn
ENSMUST00000045153.10	-0.703854369	MGI:1917016	Pik3ip1
ENSMUST00000100467.9	-0.703854369	MGI:2153272	Trrap
ENSMUST00000109315.4	-0.703854369	MGI:1923055	Nln
ENSMUST00000109869.1	-0.703854369	MGI:1346072	Psmf1
ENSMUST00000127638.7	-0.703854369	MGI:103038	Stat3
ENSMUST00000039697.13	-0.702780518	MGI:95621	Gabrb3

ENSMUST00000007865.6	-0.701620352	MGI:1916403	Ccdc124
ENSMUST00000033331.6	-0.701620352	MGI:1925843	Nrip3
ENSMUST00000044556.11	-0.701620352	MGI:2385326	Tbc1d13
ENSMUST00000111338.9	-0.700737249	MGI:1923036	Ckap5
ENSMUST00000115663.9	-0.700737249	MGI:1345297	Slc35a2
ENSMUST00000015620.6	-0.697714231	MGI:1932118	Prrt1
ENSMUST00000013304.7	-0.697503255	MGI:1201778	Atp6v0d1
ENSMUST00000023449.10	-0.697123435	MGI:1914724	Snap29
ENSMUST00000032399.11	-0.697123435	MGI:96680	Kras
ENSMUST00000089950.10	-0.697123435	MGI:99537	Rabggtb
ENSMUST00000073822.5	-0.694734704	MGI:97397	Opcml
ENSMUST00000026125.2	-0.692784532	MGI:1341044	Alyref
ENSMUST00000113979.9	-0.692784532	MGI:1336997	Lancl1
ENSMUST00000026459.5	-0.692753183	MGI:107801	Atp5b
ENSMUST00000017920.13	-0.692394571	MGI:88508	Crk
ENSMUST00000235196.1	-0.690731482	MGI:95588	Fth1
ENSMUST00000000804.6	-0.690501802	MGI:103064	Ddx3x
ENSMUST00000029005.3	-0.690501802	MGI:1913654	Rtf2
ENSMUST00000120389.7	-0.689754922	MGI:1919331	Snx12
ENSMUST00000003386.6	-0.687031659	MGI:2137210	Mrpl4
ENSMUST00000020315.12	-0.687031659	MGI:1261820	Cand1
ENSMUST00000024657.11	-0.687031659	MGI:1919307	Phf10
ENSMUST00000032728.8	-0.687031659	MGI:2444486	Tarsl2
ENSMUST00000039956.5	-0.687031659	MGI:2442940	Slc41a2
ENSMUST00000040336.11	-0.687031659	MGI:1920352	Slc22a23
ENSMUST00000049784.16	-0.687031659	MGI:1100511	Myt1l
ENSMUST00000056889.14	-0.687031659	MGI:1344380	Chd4
ENSMUST00000114415.9	-0.687031659	MGI:2679336	Satb2
ENSMUST00000026551.14	-0.685802544	MGI:1349764	Dpysl4
ENSMUST00000028826.3	-0.685802544	MGI:88395	Chgb
ENSMUST00000000430.13	-0.683313275	MGI:894693	Galnt1
ENSMUST00000045738.4	-0.683313275	MGI:1194488	Slc32a1
ENSMUST00000052725.14	-0.683313275	MGI:1353611	Sc5d
ENSMUST00000161241.7	-0.683313275	MGI:99542	Usf1
ENSMUST00000210656.1	-0.682423249	MGI:1929214	Ap3m2
ENSMUST00000039568.10	-0.681127271	MGI:1306800	Pcdh8
ENSMUST00000045262.10	-0.681127271	MGI:2677491	Ak5
ENSMUST00000100219.4	-0.681127271	MGI:2677836	Dolk
ENSMUST00000165315.7	-0.681127271	MGI:2155664	Snx14

ENSMUST00000016401.14	-0.679688222	MGI:1913640	Prelid3b
ENSMUST00000021930.9	-0.679688222	MGI:2137677	Sfxn1
ENSMUST00000174850.7	-0.679688222	MGI:106582	Rfx3
ENSMUST00000079205.13	-0.678669144	MGI:106576	Chpf
ENSMUST00000105363.7	-0.678669144	MGI:1098221	Gamt
ENSMUST00000002735.8	-0.677909601	MGI:1858213	Clpp
ENSMUST00000066819.10	-0.675456467	MGI:3036236	Tceal5
ENSMUST00000094934.10	-0.668834987	MGI:95632	Gad1
ENSMUST00000010239.5	-0.667561777	MGI:96955	Slc3a2
ENSMUST00000054442.10	-0.667561777	MGI:1915018	N6amt1
ENSMUST00000037290.11	-0.666549084	MGI:1345633	Mars1
ENSMUST00000069712.8	-0.666549084	MGI:1913564	Tpd52l2
ENSMUST00000122424.7	-0.665039745	MGI:2443990	Fam20b
ENSMUST00000038775.5	-0.66455097	MGI:97290	Ndn
ENSMUST00000027297.10	-0.664300621	MGI:2385825	Plekhb2
ENSMUST00000061071.12	-0.664300621	MGI:106530	Plppr4
ENSMUST00000006559.13	-0.662549713	MGI:1341264	Tpbg
ENSMUST00000014892.7	-0.662549713	MGI:1096575	Tex261
ENSMUST00000033498.9	-0.662549713	MGI:1343176	Timm17b
ENSMUST00000040430.7	-0.662549713	MGI:1349450	Vat1
ENSMUST00000060311.11	-0.662549713	MGI:1099804	Hip1
ENSMUST00000069324.6	-0.662549713	MGI:1916242	Zfp580
ENSMUST00000069837.3	-0.662549713	MGI:1315202	Slit3
ENSMUST00000080975.5	-0.662549713	MGI:1924301	Os9
ENSMUST00000034848.13	-0.661590631	MGI:1347060	Psma4
ENSMUST00000000349.10	-0.65854704	MGI:105386	Dbt
ENSMUST00000075069.10	-0.657478273	MGI:2446259	Ntm
ENSMUST00000030158.10	-0.65428217	MGI:1859251	Dctn3
ENSMUST00000170489.1	-0.65428217	MGI:104976	Ddx6
ENSMUST00000020653.5	-0.649322517	MGI:1913647	Sar1b
ENSMUST00000022511.9	-0.648676687	MGI:1923257	Zmym2
ENSMUST00000026254.13	-0.648676687	MGI:1861607	Gbf1
ENSMUST00000030849.12	-0.648676687	MGI:2149839	Fam126a
ENSMUST00000033430.2	-0.648676687	MGI:109493	Rab33a
ENSMUST00000038552.12	-0.648676687	MGI:1926135	Coro7
ENSMUST00000114261.8	-0.648676687	MGI:2442831	Paxx
ENSMUST00000136342.8	-0.648676687	MGI:1858303	Srrm1
ENSMUST00000238930.1	-0.648676687	MGI:3045495	Tmem205
ENSMUST00000000608.7	-0.648251167	MGI:95762	Gm2a

ENSMUST00000097281.3	-0.64581586	MGI:2444098	Heatr5b
ENSMUST00000025036.10	-0.644409427	MGI:2442355	Kdm4b
ENSMUST00000005671.9	-0.641401992	MGI:96433	Igf1r
ENSMUST00000029490.14	-0.641401992	MGI:2385184	Ahcyl1
ENSMUST00000053024.7	-0.641401992	MGI:1914328	Pgp
ENSMUST00000076265.12	-0.641401992	MGI:1915384	Upf3b
ENSMUST00000103120.4	-0.641401992	MGI:88437	Cnp
ENSMUST00000109514.7	-0.641401992	MGI:106190	Bcl11a
ENSMUST00000164744.7	-0.641401992	MGI:1341217	Uba3
ENSMUST00000033276.10	-0.640951565	MGI:98917	Uros
ENSMUST00000047463.14	-0.640951565	MGI:2144423	Arhgap44
ENSMUST00000154132.7	-0.640951565	MGI:1913451	Vta1
ENSMUST00000199754.4	-0.640951565	MGI:2443225	Gatad2b
ENSMUST00000099353.5	-0.640653348	MGI:1915038	Sfr1
ENSMUST00000047534.11	-0.638259853	MGI:2448487	Fam168b
ENSMUST00000109734.7	-0.638143502	MGI:109486	Prdx2
ENSMUST00000102758.7	-0.638099112	MGI:106919	Vdac1
ENSMUST00000020312.12	-0.63708311	MGI:3026965	Mcu
ENSMUST00000034904.13	-0.636028843	MGI:1916051	Elovl5
ENSMUST00000059539.4	-0.635362348	MGI:1923555	Nap1l5
ENSMUST00000069064.6	-0.633938473	MGI:1916351	Ydjc
ENSMUST00000021063.12	-0.63181376	MGI:1914247	Psmd12
ENSMUST00000029266.13	-0.629653098	MGI:106008	Anxa5
ENSMUST00000159283.7	-0.629653098	MGI:1922090	Manf
ENSMUST00000005503.4	-0.627808962	MGI:1343961	Msh6
ENSMUST00000010434.7	-0.627808962	MGI:2138299	AI597479
ENSMUST00000014221.12	-0.627808962	MGI:1927185	Chp1
ENSMUST00000020826.5	-0.627808962	MGI:1354709	Sap30l
ENSMUST00000023029.14	-0.627808962	MGI:2443584	L3mbtl2
ENSMUST00000024869.7	-0.627808962	MGI:1858896	Spast
ENSMUST00000026410.1	-0.627808962	MGI:1921580	Dnajc14
ENSMUST00000030189.13	-0.627808962	MGI:2654325	Gba2
ENSMUST00000031170.9	-0.627808962	MGI:99700	Cenpc1
ENSMUST00000035899.7	-0.627808962	MGI:2178598	Bloc1s5
ENSMUST00000037484.14	-0.627808962	MGI:1922075	Senp6
ENSMUST00000040561.5	-0.627808962	MGI:2144506	Rundc1
ENSMUST00000041364.12	-0.627808962	MGI:1922666	Nop14
ENSMUST00000043983.10	-0.627808962	MGI:1926158	Igsf3
ENSMUST00000044053.12	-0.627808962	MGI:1917581	Shprh

ENSMUST00000050556.10	-0.627808962	MGI:2444804	Bod1l
ENSMUST00000053177.13	-0.627808962	MGI:1096875	Wdfy3
ENSMUST00000058060.13	-0.627808962	MGI:1916806	Bod1
ENSMUST00000072835.6	-0.627808962	MGI:1918800	Ccdc112
ENSMUST00000102911.9	-0.627808962	MGI:2140592	Slc44a1
ENSMUST00000102929.1	-0.627808962	MGI:2140279	Tdrd7
ENSMUST00000105831.8	-0.627808962	MGI:1923935	Eif4g3
ENSMUST00000108075.8	-0.627808962	MGI:2144475	Usp32
ENSMUST00000116273.8	-0.627808962	MGI:1196256	Kdm1a
ENSMUST00000122421.1	-0.627808962	MGI:1920670	Ccsap
ENSMUST00000161046.8	-0.627808962	MGI:2182061	Usp7
ENSMUST00000166489.7	-0.627808962	MGI:2153608	Ube2j2
ENSMUST00000171165.7	-0.627808962	MGI:1915171	Ube2f
ENSMUST00000171337.9	-0.627808962	MGI:1924574	Sorbs2
ENSMUST00000177768.2	-0.627808962	MGI:1920635	Fam177a
ENSMUST00000178092.1	-0.627808962	MGI:97365	Pnp
ENSMUST00000178098.7	-0.627808962	MGI:2150386	Dcun1d1
ENSMUST00000187510.6	-0.627808962	MGI:88398	Chrm3
ENSMUST00000031419.5	-0.626872202	MGI:1916198	Fam216a
ENSMUST00000046777.10	-0.626872202	MGI:1921166	Ift57
ENSMUST00000184322.7	-0.626872202	MGI:1916892	Mlip
ENSMUST00000017620.9	-0.622948909	MGI:1923275	Cant1
ENSMUST00000021046.5	-0.622948909	MGI:1919297	Ddx42
ENSMUST00000040182.12	-0.622948909	MGI:1925177	Ccdc88a
ENSMUST00000052266.14	-0.622948909	MGI:1096376	Exoc4
ENSMUST00000053663.10	-0.622948909	MGI:1917819	Wdr36
ENSMUST00000085715.6	-0.622948909	MGI:1920955	Mark4
ENSMUST00000110909.8	-0.622948909	MGI:1860493	Arhgef7
ENSMUST00000207484.1	-0.622948909	MGI:2385902	Picalm
ENSMUST00000042235.14	-0.622366621	MGI:1096881	Eef1a1
ENSMUST00000058667.14	-0.622366621	MGI:3027390	Lrrc4b
ENSMUST00000035925.6	-0.620593864	MGI:1916951	Slc7a6os
ENSMUST00000201352.3	-0.620593864	MGI:1333875	Babam2
ENSMUST00000021864.7	-0.619203947	MGI:105082	Ssr1
ENSMUST00000057188.6	-0.619203947	MGI:104615	Cnr1
ENSMUST00000123948.7	-0.619203947	MGI:104913	Abi1
ENSMUST00000165576.7	-0.618267207	MGI:107892	Fam3c
ENSMUST00000065364.4	-0.616026167	MGI:1913950	Chmp3
ENSMUST00000160379.3	-0.615071224	MGI:1330828	Cdk5r2

ENSMUST00000072406.4	-0.615013612	MGI:2441932	Prepl
ENSMUST00000082365.5	-0.60951157	MGI:1888971	Sult4a1
ENSMUST00000023391.15	-0.608453623	MGI:1332635	Mrpl40
ENSMUST00000031560.13	-0.608453623	MGI:1924947	Mmab
ENSMUST00000036615.6	-0.608453623	MGI:1889341	Hacd3
ENSMUST00000025290.6	-0.606705025	MGI:1098233	Impact
ENSMUST00000027451.12	-0.606705025	MGI:98277	Epha4
ENSMUST00000029092.12	-0.606705025	MGI:2183559	Arfgap1
ENSMUST00000031390.9	-0.606705025	MGI:1346076	Mmp17
ENSMUST00000036210.6	-0.606705025	MGI:2444232	Poglut1
ENSMUST00000045247.8	-0.606705025	MGI:2158400	Wdr18
ENSMUST00000220449.1	-0.606705025	MGI:5012260	Gm20075
ENSMUST00000047328.10	-0.605339787	MGI:96437	Igfbp2
ENSMUST00000018184.9	-0.603261733	MGI:1922028	Rrp7a
ENSMUST00000021390.8	-0.603261733	MGI:95636	Galc
ENSMUST00000022699.9	-0.603261733	MGI:1195462	Gfra2
ENSMUST00000025243.4	-0.603261733	MGI:1920723	Iws1
ENSMUST00000025805.7	-0.603261733	MGI:1277225	Cnih2
ENSMUST00000026859.10	-0.603261733	MGI:1919425	Mfsd8
ENSMUST00000028752.7	-0.603261733	MGI:2443189	Vps39
ENSMUST00000035766.12	-0.603261733	MGI:1919654	Wdr44
ENSMUST00000045235.7	-0.603261733	MGI:2141466	Bmt2
ENSMUST00000047257.14	-0.603261733	MGI:2387638	Katnal1
ENSMUST00000067918.11	-0.603261733	MGI:2442087	Ppm1h
ENSMUST00000068233.10	-0.603261733	MGI:1913272	Kcnmb4
ENSMUST00000077737.12	-0.603261733	MGI:1914632	Brd3
ENSMUST00000085814.4	-0.603261733	MGI:1276523	Ncoa1
ENSMUST00000109741.8	-0.603261733	MGI:1861901	Mast1
ENSMUST00000117061.7	-0.603261733	MGI:894310	Septin8
ENSMUST00000143783.8	-0.603261733	MGI:96785	Lhx2
ENSMUST00000156481.7	-0.603261733	MGI:1914249	Med28
ENSMUST00000166820.7	-0.603261733	MGI:1919000	R3hdm2
ENSMUST00000200535.5	-0.603261733	MGI:1888998	Usp29
ENSMUST00000224797.1	-0.603261733	MGI:1860775	Actr8
ENSMUST00000238569.1	-0.603261733	MGI:1340044	Lrp8
ENSMUST00000231335.1	-0.602627765	MGI:6215011	Gm49601
ENSMUST00000114666.8	-0.601880908	MGI:1201780	Atp6v1a
ENSMUST00000020804.7	-0.597361368	MGI:1913819	Gdpd1
ENSMUST00000021554.15	-0.597361368	MGI:2137706	Actn1

ENSMUST00000044766.14	-0.597361368	MGI:1923029	4930402H24Rik
ENSMUST00000064107.6	-0.597361368	MGI:1921944	Tbc1d30
ENSMUST00000076372.4	-0.597361368	MGI:109580	Sf3b4
ENSMUST00000089215.11	-0.597361368	MGI:1858223	Cntn6
ENSMUST00000102781.9	-0.597361368	MGI:96628	Jak1
ENSMUST00000025356.3	-0.59630625	MGI:2146021	Mal2
ENSMUST00000017534.14	-0.596038331	MGI:101863	Aldoc
ENSMUST00000001042.9	-0.59573104	MGI:1915031	Ilf2
ENSMUST00000007130.14	-0.595467777	MGI:88276	Ctnnb1
ENSMUST00000021062.11	-0.594534132	MGI:105037	Ddx5
ENSMUST00000022601.6	-0.592295152	MGI:109568	Wbp4
ENSMUST00000034326.6	-0.592295152	MGI:2180801	Atp13a1
ENSMUST00000110857.4	-0.592295152	MGI:107450	Dld
ENSMUST00000177955.7	-0.592295152	MGI:1913353	Naxd
ENSMUST00000180086.2	-0.592295152	MGI:95893	H1f0
ENSMUST00000190571.6	-0.592295152	MGI:1923957	Clasp1
ENSMUST00000215683.1	-0.592295152	MGI:1924113	Dcun1d5
ENSMUST00000004508.12	-0.592089781	MGI:1915070	Tmed4
ENSMUST00000033497.8	-0.592089781	MGI:1859638	Pqbp1
ENSMUST00000030787.8	-0.589468931	MGI:97912	Rheb
ENSMUST00000028509.10	-0.58870454	MGI:2135962	Gorasp2
ENSMUST00000049357.9	-0.58870454	MGI:1917838	Pnrc1
ENSMUST00000155671.7	-0.58870454	MGI:1928344	Vps29
ENSMUST00000049509.6	-0.587536362	MGI:2142534	Vat1l
ENSMUST00000025862.14	-0.587186727	MGI:99603	Smarca2
ENSMUST00000170788.8	-0.587186727	MGI:1353557	Schip1
ENSMUST00000042477.12	-0.586382103	MGI:106354	Vps25
ENSMUST00000033540.5	-0.584231901	MGI:1333804	Vbp1
ENSMUST00000022577.5	-0.582719167	MGI:1914552	Zc3h13
ENSMUST00000031354.10	-0.582719167	MGI:1861729	Abcb9
ENSMUST00000036187.8	-0.582719167	MGI:2387194	Qsox2
ENSMUST00000040853.10	-0.582719167	MGI:1917378	Oxsr1
ENSMUST00000044672.10	-0.582719167	MGI:1925584	Cdk19
ENSMUST00000097593.8	-0.582719167	MGI:1918552	Arhgap26
ENSMUST00000110000.7	-0.582719167	MGI:1915127	Naa20
ENSMUST00000115577.8	-0.582719167	MGI:1329042	Sgce
ENSMUST00000161659.7	-0.582719167	MGI:1332247	Slc33a1
ENSMUST00000166793.9	-0.58136892	MGI:1298379	Matr3
ENSMUST00000031099.3	-0.579946122	MGI:1334417	Grpel1

ENSMUST00000011058.8	-0.579424673	MGI:1351627	Pdhx
ENSMUST00000023150.6	-0.579424673	MGI:1916303	1810013L24Rik
ENSMUST00000082094.4	-0.579424673	MGI:1917206	Ptcd3
ENSMUST00000210812.1	-0.579424673	MGI:1916249	Anapc10
ENSMUST00000028825.4	-0.577820131	MGI:1915465	Fam98b
ENSMUST00000034856.14	-0.577820131	MGI:97075	Mpi
ENSMUST00000103195.4	-0.577820131	MGI:3051596	Znhit3
ENSMUST00000130418.7	-0.577820131	MGI:1340024	Aldh1l1
ENSMUST00000167256.7	-0.577820131	MGI:1888697	Taf9
ENSMUST00000237871.1	-0.577820131	MGI:1914262	Tm9sf3
ENSMUST00000020705.4	-0.576870648	MGI:1890613	Pes1
ENSMUST00000162538.8	-0.576870648	MGI:1922083	Ciapin1
ENSMUST00000063694.9	-0.576243082	MGI:1354948	Klf13
ENSMUST00000068054.8	-0.576243082	MGI:1917777	Stambp
ENSMUST00000038228.10	-0.571377789	MGI:106558	Tmx4
ENSMUST00000163821.2	-0.569960295	MGI:1918903	Shtn1
ENSMUST00000237651.1	-0.567991747	MGI:1278334	Zfand5
ENSMUST00000000058.6	-0.567858977	MGI:107571	Cav2
ENSMUST00000015712.14	-0.567858977	MGI:96820	Lpl
ENSMUST00000028900.10	-0.567858977	MGI:2136772	Vps16
ENSMUST00000031051.7	-0.567858977	MGI:1915817	Cgref1
ENSMUST00000034472.15	-0.567858977	MGI:1933825	Jam3
ENSMUST00000042405.7	-0.567858977	MGI:95486	Fbl
ENSMUST00000154460.7	-0.567858977	MGI:1929212	Ap3m1
ENSMUST00000168410.8	-0.567858977	MGI:2443456	Tbc1d24
ENSMUST00000205573.1	-0.567858977	MGI:99137	Xrcc1
ENSMUST00000223272.1	-0.567858977	MGI:1914596	Daam1
ENSMUST00000232873.1	-0.567858977	MGI:109489	Snrpc
ENSMUST00000111089.7	-0.567677628	MGI:97314	Nefm
ENSMUST00000000287.8	-0.565273527	MGI:1921867	Scpep1
ENSMUST00000002048.7	-0.565273527	MGI:1917457	Taco1
ENSMUST00000003677.10	-0.565273527	MGI:1918923	Rnf215
ENSMUST00000029374.7	-0.565273527	MGI:1347075	Nbea
ENSMUST00000029570.8	-0.565273527	MGI:1201609	Mfsd14a
ENSMUST00000031795.7	-0.565273527	MGI:1350921	Fkbp9
ENSMUST00000038523.14	-0.565273527	MGI:2682307	Ric8b
ENSMUST00000047091.13	-0.565273527	MGI:1916065	Btbd10
ENSMUST00000052185.4	-0.565273527	MGI:2152819	B3galt6
ENSMUST00000091257.10	-0.565273527	MGI:1914664	Mfn1

ENSMUST00000092915.11	-0.565273527	MGI:1921398	Cluh
ENSMUST00000102549.9	-0.565273527	MGI:1921802	Nipal3
ENSMUST00000105682.8	-0.565273527	MGI:2683486	Rere
ENSMUST00000108493.2	-0.565273527	MGI:3654828	Dact3
ENSMUST00000166743.8	-0.565273527	MGI:2444022	Mapk1ip1l
ENSMUST00000038446.9	-0.563641245	MGI:107494	Myl12b
ENSMUST00000082437.9	-0.563641245	MGI:1927947	Selenof
ENSMUST00000226053.1	-0.563460573	MGI:1194492	Hdgfl2
ENSMUST00000021959.10	-0.563015378	MGI:1916922	Txndc15
ENSMUST00000061483.8	-0.563015378	MGI:88184	Bmyc
ENSMUST00000029684.14	-0.561829581	MGI:1346346	Scamp3
ENSMUST00000033182.9	-0.561636442	MGI:1195267	Ilk
ENSMUST00000028511.7	-0.561153739	MGI:1859652	Mtx2
ENSMUST00000032874.13	-0.561153739	MGI:700011	Sh3gl3
ENSMUST00000109905.4	-0.559167151	MGI:1914761	Tmed9
ENSMUST00000121820.8	-0.559167151	MGI:1891351	Spock2
ENSMUST00000005509.10	-0.558515387	MGI:109355	Stx1a
ENSMUST00000002289.7	-0.557408902	MGI:1355274	Uchl3
ENSMUST00000025893.6	-0.557408902	MGI:1928393	Arl2
ENSMUST00000028250.8	-0.557408902	MGI:1915121	Mrrf
ENSMUST00000120285.7	-0.557408902	MGI:1929464	Sec11a
ENSMUST00000148843.9	-0.557408902	MGI:1891692	Hnrnpr
ENSMUST00000167496.7	-0.557408902	MGI:1925927	Rnf20
ENSMUST00000195014.5	-0.554519287	MGI:1913506	Ssr2
ENSMUST00000021297.5	-0.553707078	MGI:1919592	Lsm12
ENSMUST00000058418.7	-0.553630985	MGI:2385330	Slc29a4
ENSMUST00000001280.13	-0.550272546	MGI:105490	Gramd1a
ENSMUST00000014421.14	-0.550272546	MGI:1932101	Ankrd17
ENSMUST00000018795.12	-0.550272546	MGI:2144471	Tada2a
ENSMUST00000030771.11	-0.550272546	MGI:99470	Dnajc2
ENSMUST00000032920.4	-0.550272546	MGI:105491	Cdipt
ENSMUST00000036682.8	-0.550272546	MGI:1289230	Pxk
ENSMUST00000038356.12	-0.550272546	MGI:1917343	Ube2q1
ENSMUST00000043150.5	-0.550272546	MGI:1919129	Acsl5
ENSMUST00000051145.14	-0.550272546	MGI:2139593	Wdr47
ENSMUST00000060253.4	-0.550272546	MGI:1335089	Fem1a
ENSMUST00000065797.6	-0.550272546	MGI:1924219	Chst1
ENSMUST00000068860.12	-0.550272546	MGI:108034	Epha6
ENSMUST00000082424.10	-0.550272546	MGI:894288	Selenop

ENSMUST00000115716.8	-0.550272546	MGI:1921252	Psd2
ENSMUST00000121759.7	-0.550272546	MGI:1354961	Synj1
ENSMUST00000141722.7	-0.550272546	MGI:1926058	Stxbp5
ENSMUST00000143850.7	-0.550272546	MGI:2151483	Derl2
ENSMUST00000210032.1	-0.550272546	MGI:1915522	Lingo1
ENSMUST00000212459.1	-0.550272546	MGI:1342270	Large1
ENSMUST00000034441.7	-0.550147118	MGI:2384560	Aars
ENSMUST00000021177.14	-0.547920217	MGI:1921386	Sec14l1
ENSMUST00000026568.9	-0.547920217	MGI:1351664	Ptdss2
ENSMUST00000030894.14	-0.547920217	MGI:1920196	Lrrc47
ENSMUST00000058351.15	-0.54677035	MGI:97565	Pgm1
ENSMUST00000099092.7	-0.54677035	MGI:1862037	Slc12a5
ENSMUST00000153060.7	-0.546088621	MGI:108054	Rit2
ENSMUST00000074072.4	-0.545316897	MGI:3642298	Rps18-ps6
ENSMUST00000036928.11	-0.541624787	MGI:95405	Ephx1
ENSMUST00000037636.3	-0.54029284	MGI:96568	Ina
ENSMUST00000081677.11	-0.54029284	MGI:1917475	Ppil3
ENSMUST00000019638.14	-0.539265907	MGI:1349439	Cops6
ENSMUST00000027444.14	-0.539265907	MGI:1270843	Pde6d
ENSMUST00000033673.6	-0.539265907	MGI:1855692	Nono
ENSMUST00000045366.9	-0.539265907	MGI:2142792	Dcun1d2
ENSMUST00000064910.6	-0.539265907	MGI:1329037	Strap
ENSMUST00000119797.7	-0.539265907	MGI:1929459	Rabgef1
ENSMUST00000126641.1	-0.539265907	MGI:1333805	Srsf10
ENSMUST00000178691.1	-0.539265907	MGI:95049	Ubl4a
ENSMUST00000048613.13	-0.539236137	MGI:2444149	A830018L16Rik
ENSMUST00000039144.6	-0.538070402	MGI:1929895	Clstn1
ENSMUST00000003572.9	-0.537235349	MGI:2449057	Gars
ENSMUST00000018651.13	-0.537235349	MGI:1341836	Trpv2
ENSMUST00000031985.12	-0.537235349	MGI:1859353	Mkrn1
ENSMUST00000108883.9	-0.537235349	MGI:88255	Anxa6
ENSMUST00000204702.2	-0.537235349	MGI:107422	Hspa4l
ENSMUST00000206984.1	-0.537235349	MGI:1339975	Pak1
ENSMUST00000173114.7	-0.53619568	MGI:88548	Csnk2b
ENSMUST00000022962.7	-0.534230347	MGI:1913986	Emc2
ENSMUST00000034903.6	-0.534230347	MGI:1309515	Gsta4
ENSMUST00000030541.12	-0.531340416	MGI:109369	Hp1bp3
ENSMUST00000031556.13	-0.531340416	MGI:1919150	Tmem106b
ENSMUST00000168361.7	-0.531340416	MGI:1931751	Pum2

ENSMUST00000061446.7	-0.52895433	MGI:3607706	Tmem130
ENSMUST00000053766.13	-0.528544176	MGI:1345634	Amfr
ENSMUST00000067327.10	-0.525841966	MGI:104565	Cdkn1b
ENSMUST00000010348.6	-0.525799565	MGI:1915415	Fdx2
ENSMUST00000022787.7	-0.525799565	MGI:1355323	Slc7a8
ENSMUST00000023882.13	-0.525799565	MGI:1915438	Sympk
ENSMUST00000053336.7	-0.525799565	MGI:1919440	2510009E07Rik
ENSMUST00000102742.10	-0.525799565	MGI:1915312	Btf3l4
ENSMUST00000105617.7	-0.525799565	MGI:2444159	Ipcef1
ENSMUST00000130643.8	-0.525799565	MGI:106908	Srpk1
ENSMUST00000192503.1	-0.525799565	MGI:2447313	Pcdha3
ENSMUST00000069066.13	-0.525471425	MGI:1261783	Fam49a
ENSMUST00000027916.12	-0.52452465	MGI:1338800	Bpnt1
ENSMUST00000016638.7	-0.524055292	MGI:88329	Cd34
ENSMUST00000028408.2	-0.524055292	MGI:96013	Hat1
ENSMUST00000068045.13	-0.524055292	MGI:1890773	Actn4
ENSMUST00000026743.13	-0.523690356	MGI:107876	Uqcrc1
ENSMUST00000029075.4	-0.523200301	MGI:1924079	Dok5
ENSMUST00000063417.10	-0.523200301	MGI:1926232	Srsf7
ENSMUST00000094329.10	-0.523200301	MGI:2153045	Elmo2
ENSMUST00000062356.6	-0.522557856	MGI:97143	Marcksl1
ENSMUST00000094657.9	-0.522356442	MGI:1915848	Dnajc8
ENSMUST00000167662.7	-0.519682036	MGI:1914708	Ergic1
ENSMUST00000049126.12	-0.519247764	MGI:2152889	Dner
ENSMUST00000031058.14	-0.518168684	MGI:2140967	Mapre3
ENSMUST00000110105.9	-0.518168684	MGI:1919824	Zc3h14
ENSMUST00000188346.6	-0.518168684	MGI:1928739	Dnajb2
ENSMUST00000022517.8	-0.517335855	MGI:1915881	Cryl1
ENSMUST00000017408.13	-0.515686902	MGI:1355322	Exosc10
ENSMUST00000022163.14	-0.515686902	MGI:1202875	Btf3
ENSMUST00000031121.9	-0.515686902	MGI:95616	Gabra4
ENSMUST00000089302.10	-0.515686902	MGI:894681	Usp9x
ENSMUST00000105369.7	-0.515686902	MGI:1354170	Cbarp
ENSMUST00000110036.10	-0.515686902	MGI:95481	Ptk2
ENSMUST00000081834.9	-0.513422704	MGI:1918953	Armcx3
ENSMUST00000174548.7	-0.513422704	MGI:104816	Hnrnpl
ENSMUST00000024486.13	-0.508938686	MGI:1928138	Mrps23
ENSMUST00000110388.9	-0.508938686	MGI:109602	Gphn
ENSMUST00000165175.7	-0.508938686	MGI:1927338	Sv2b

ENSMUST00000040381.14	-0.50668008	MGI:2385165	Ncoa5
ENSMUST00000075657.7	-0.50668008	MGI:1337060	Ap3s2
ENSMUST00000114787.7	-0.50668008	MGI:2443815	Stxbp5l
ENSMUST00000117598.7	-0.50668008	MGI:2146407	Vps8
ENSMUST00000178636.1	-0.50668008	MGI:1890165	Larp1
ENSMUST00000040656.7	-0.505349248	MGI:1915271	Bphl
ENSMUST00000087933.9	-0.505349248	MGI:102724	Tpp2
ENSMUST00000001127.10	-0.499980999	MGI:1915061	Poldip2
ENSMUST00000031788.8	-0.499980999	MGI:1889802	Hibadh
ENSMUST00000094651.3	-0.499980999	MGI:1924095	Eid2b
ENSMUST00000031513.13	-0.498606963	MGI:104896	Srsf9
ENSMUST00000049658.13	-0.498606963	MGI:1197524	Pitpnm1
ENSMUST00000057578.15	-0.498606963	MGI:1917297	Trnt1
ENSMUST00000090929.11	-0.498606963	MGI:1919546	Rusc1
ENSMUST00000161576.7	-0.498606963	MGI:1927406	Herpud1
ENSMUST00000069718.14	-0.498541638	MGI:1347093	Fto
ENSMUST00000034740.14	-0.496857929	MGI:97297	Nedd4
ENSMUST00000102874.10	-0.496244049	MGI:97051	Mdh1
ENSMUST00000029256.8	-0.495050751	MGI:1916526	Sec62
ENSMUST00000105365.8	-0.494973409	MGI:893588	Cirbp
ENSMUST00000017946.5	-0.494581867	MGI:1915248	Retreg3
ENSMUST00000021066.3	-0.494581867	MGI:1859167	Cacng4
ENSMUST00000021183.3	-0.494581867	MGI:1889295	Eral1
ENSMUST00000024078.14	-0.494581867	MGI:1915207	Marchf5
ENSMUST00000025264.7	-0.494581867	MGI:1921570	Wdr33
ENSMUST00000029060.10	-0.494581867	MGI:1330826	Atp9a
ENSMUST00000034611.14	-0.494581867	MGI:2143230	Phldb1
ENSMUST00000035985.7	-0.494581867	MGI:2444450	Fbxl18
ENSMUST00000035988.15	-0.494581867	MGI:2145316	Txndc5
ENSMUST00000036226.5	-0.494581867	MGI:2444737	Fem1c
ENSMUST00000036759.10	-0.494581867	MGI:106463	Washc2
ENSMUST00000036862.4	-0.494581867	MGI:2145130	Cog5
ENSMUST00000037119.3	-0.494581867	MGI:94901	Dlx1
ENSMUST00000041375.10	-0.494581867	MGI:2445031	Sik2
ENSMUST00000042456.3	-0.494581867	MGI:1349403	B3galt1
ENSMUST00000042767.8	-0.494581867	MGI:2679448	Slitrk5
ENSMUST00000046254.2	-0.494581867	MGI:1917780	Lrfn2
ENSMUST00000046835.13	-0.494581867	MGI:2444668	Fnip1
ENSMUST00000059914.12	-0.494581867	MGI:1913435	Virma

ENSMUST00000061578.8	-0.494581867	MGI:2443480	Setx
ENSMUST00000063551.6	-0.494581867	MGI:106334	Rgs7bp
ENSMUST00000076270.12	-0.494581867	MGI:1860236	Rabep1
ENSMUST00000076949.12	-0.494581867	MGI:1921504	Gpn1
ENSMUST00000077821.9	-0.494581867	MGI:107858	Arhgap39
ENSMUST00000080449.6	-0.494581867	MGI:96243	Hspa2
ENSMUST00000086325.12	-0.494581867	MGI:2442638	Flywch1
ENSMUST00000087031.6	-0.494581867	MGI:1913789	Xpo5
ENSMUST00000097644.8	-0.494581867	MGI:3036234	Hdac4
ENSMUST00000099384.3	-0.494581867	MGI:2145317	Brd9
ENSMUST00000099414.4	-0.494581867	MGI:4834573	Zfp955b
ENSMUST00000100125.11	-0.494581867	MGI:1916259	Thap7
ENSMUST00000102793.10	-0.494581867	MGI:2137022	Tm2d1
ENSMUST00000107749.3	-0.494581867	MGI:2386030	Gabbr2
ENSMUST00000112701.7	-0.494581867	MGI:2442792	Cdh7
ENSMUST00000115160.9	-0.494581867	MGI:1919899	Tmem209
ENSMUST00000119245.7	-0.494581867	MGI:1928469	Trip4
ENSMUST00000128616.5	-0.494581867	MGI:1922843	Malsu1
ENSMUST00000132527.8	-0.494581867	MGI:894686	Col9a3
ENSMUST00000153148.7	-0.494581867	MGI:1922909	Wdr54
ENSMUST00000162645.7	-0.494581867	MGI:3040693	Zmiz1
ENSMUST00000167715.7	-0.494581867	MGI:107157	Sin3a
ENSMUST00000171847.7	-0.494581867	MGI:1927073	Slc29a1
ENSMUST00000173246.7	-0.494581867	MGI:104813	Jarid2
ENSMUST00000174766.1	-0.494581867	MGI:2143628	L3mbtl3
ENSMUST00000178384.1	-0.494581867	MGI:1915555	Moap1
ENSMUST00000191268.6	-0.494581867	MGI:1922919	Pik3r4
ENSMUST00000194190.1	-0.494581867	MGI:1935212	Pcdhga1
ENSMUST00000222314.1	-0.494581867	MGI:107595	Sp4
ENSMUST00000237880.1	-0.494581867	MGI:1917675	Csnk1g3
ENSMUST00000003345.9	-0.494388187	MGI:103574	Amph
ENSMUST00000010899.13	-0.493872518	MGI:1351477	Cars
ENSMUST00000019975.13	-0.493809509	MGI:1890563	Wasf1
ENSMUST00000167199.2	-0.493231264	MGI:2137219	Mrpl16
ENSMUST00000036951.12	-0.491594883	MGI:1344408	Pebp1
ENSMUST00000010536.8	-0.491329406	MGI:1858260	Gosr1
ENSMUST00000027592.5	-0.491329406	MGI:1915062	Ubxn4
ENSMUST00000090568.6	-0.491329406	MGI:97816	Ptprz1
ENSMUST00000099547.3	-0.491329406	MGI:2145496	Fam8a1

ENSMUST00000170928.7	-0.491329406	MGI:1913463	Med7
ENSMUST00000021459.13	-0.490291285	MGI:1916865	Rab15
ENSMUST00000023507.12	-0.488874399	MGI:1861437	Gsk3b
ENSMUST00000034198.14	-0.487270186	MGI:95775	Gnao1
ENSMUST00000051477.12	-0.486083439	MGI:106211	Cdc42
ENSMUST00000034426.13	-0.485903875	MGI:1934754	Kars
ENSMUST00000003946.8	-0.484735254	MGI:1914869	Nob1
ENSMUST00000017354.12	-0.484735254	MGI:1344385	Med24
ENSMUST00000017637.12	-0.484735254	MGI:96439	Igfbp4
ENSMUST00000040314.11	-0.484735254	MGI:1924188	Rbm17
ENSMUST00000103015.3	-0.484735254	MGI:1914858	Narf
ENSMUST00000159086.9	-0.484735254	MGI:1921793	Zfp871
ENSMUST00000025505.6	-0.484317303	MGI:1914915	Dctn4
ENSMUST00000001834.3	-0.483357524	MGI:106379	Rtcb
ENSMUST00000109764.7	-0.483357524	MGI:97311	Nfix
ENSMUST00000034244.8	-0.481784181	MGI:1921416	Tmem38a
ENSMUST00000163153.8	-0.481784181	MGI:97887	Rdx
ENSMUST00000002413.14	-0.48130425	MGI:2384577	Tmem161a
ENSMUST00000003536.8	-0.48130425	MGI:1914474	Med29
ENSMUST00000025830.8	-0.48130425	MGI:1860297	Apba1
ENSMUST00000028350.8	-0.48130425	MGI:1914437	Zmynd19
ENSMUST00000031198.10	-0.48130425	MGI:1914516	Dipk1a
ENSMUST00000034228.15	-0.48130425	MGI:1349429	Arl2bp
ENSMUST00000038374.12	-0.48130425	MGI:97499	Pcca
ENSMUST00000040514.7	-0.48130425	MGI:109334	Irs2
ENSMUST00000042035.15	-0.48130425	MGI:1933736	Adgrb1
ENSMUST00000068916.15	-0.48130425	MGI:1919160	Plpp5
ENSMUST00000080368.12	-0.48130425	MGI:1354710	Atp8a2
ENSMUST00000101164.10	-0.48130425	MGI:1924819	Limch1
ENSMUST00000121326.7	-0.48130425	MGI:1916457	Srsf11
ENSMUST00000141755.7	-0.48130425	MGI:1914743	Mettl16
ENSMUST00000166280.7	-0.48130425	MGI:2146838	Dlk2
ENSMUST00000171691.8	-0.48130425	MGI:1922012	Mdga1
ENSMUST00000192069.5	-0.48130425	MGI:99516	Tnr
ENSMUST00000231360.1	-0.48130425	MGI:2443596	Senp5
ENSMUST00000026009.9	-0.479528377	MGI:1929699	Arl3
ENSMUST00000070658.15	-0.479528377	MGI:2447670	Mgrn1
ENSMUST00000030816.3	-0.478732501	MGI:1196227	Dffa
ENSMUST00000035058.9	-0.478732501	MGI:1352747	Cspg5

ENSMUST00000043962.8	-0.478732501	MGI:1917207	Cdc16
ENSMUST00000063886.3	-0.478732501	MGI:102806	Acvr2a
ENSMUST00000068282.6	-0.478732501	MGI:1929492	Atl2
ENSMUST00000103198.10	-0.478732501	MGI:1914384	Nop56
ENSMUST00000028005.2	-0.478106316	MGI:1913697	Mgst3
ENSMUST00000019382.16	-0.4769859	MGI:1915408	Tecr
ENSMUST00000017548.6	-0.475422837	MGI:98020	Rpl19
ENSMUST00000153500.7	-0.474255512	MGI:97744	Por
ENSMUST00000020223.7	-0.473244955	MGI:2444679	Tcp11l2
ENSMUST00000030457.11	-0.473244955	MGI:1355328	Nasp
ENSMUST00000033198.5	-0.473244955	MGI:102675	Crym
ENSMUST00000102993.9	-0.473244955	MGI:104632	Ube2h
ENSMUST00000165199.7	-0.473244955	MGI:88077	Arsa
ENSMUST00000022634.8	-0.471495847	MGI:1332659	Bnip3l
ENSMUST00000021807.12	-0.470272524	MGI:1926209	Dek
ENSMUST00000022450.5	-0.470272524	MGI:1921694	Tasor
ENSMUST00000031598.10	-0.470272524	MGI:1919240	Ddx54
ENSMUST00000033541.4	-0.470272524	MGI:1914641	Fundc2
ENSMUST00000036407.5	-0.470272524	MGI:2147545	Fam160b1
ENSMUST00000040971.13	-0.470272524	MGI:1100859	Capn5
ENSMUST00000045537.3	-0.470272524	MGI:88399	Chrm4
ENSMUST00000051310.12	-0.470272524	MGI:2150302	Ddhd1
ENSMUST00000053768.13	-0.470272524	MGI:2384963	Stxbp6
ENSMUST00000055195.10	-0.470272524	MGI:1861746	Lmbr1
ENSMUST00000067786.8	-0.470272524	MGI:97851	Slc20a2
ENSMUST00000067924.12	-0.470272524	MGI:2140839	Lrrc8c
ENSMUST00000068351.13	-0.470272524	MGI:2141485	Lrtm2
ENSMUST00000085701.6	-0.470272524	MGI:1917631	Tecpr1
ENSMUST00000089362.8	-0.470272524	MGI:1913565	Senp7
ENSMUST00000097888.9	-0.470272524	MGI:2446630	Ago1
ENSMUST00000100196.8	-0.470272524	MGI:2384774	Alg1
ENSMUST00000109552.2	-0.470272524	MGI:97860	Rasa1
ENSMUST00000115609.9	-0.470272524	MGI:88470	Comt
ENSMUST00000115642.7	-0.470272524	MGI:1333752	Hdac6
ENSMUST00000224726.2	-0.470272524	MGI:97520	Pcx
ENSMUST00000018992.3	-0.468208981	MGI:1914297	Rars
ENSMUST00000040907.7	-0.468208981	MGI:1347059	Decr2
ENSMUST00000070334.9	-0.468208981	MGI:1915651	G6pc3
ENSMUST00000081593.12	-0.467218263	MGI:97555	Pgk1

ENSMUST00000025649.9	-0.465351086	MGI:1202384	Ddb1
ENSMUST00000135454.7	-0.463571016	MGI:1921348	Czib
ENSMUST00000043964.12	-0.463378037	MGI:1328355	Wfs1
ENSMUST00000020263.13	-0.462678058	MGI:1926462	Hnrnph3
ENSMUST00000029444.12	-0.460961035	MGI:2137357	Trim33
ENSMUST00000032165.15	-0.460961035	MGI:1928760	Ruvbl1
ENSMUST00000037376.13	-0.460961035	MGI:1860418	Nagk
ENSMUST00000048898.16	-0.460961035	MGI:1891693	Mtmr7
ENSMUST00000057093.6	-0.460961035	MGI:1924536	Nkrf
ENSMUST00000059407.8	-0.460961035	MGI:2679447	Slitrk3
ENSMUST00000066936.8	-0.460961035	MGI:1915089	Gpsm1
ENSMUST00000074144.10	-0.460961035	MGI:91860	Dcaf8
ENSMUST00000089926.5	-0.460961035	MGI:1914782	Mfap1a
ENSMUST00000094215.9	-0.460961035	MGI:1891421	Mesd
ENSMUST00000095267.5	-0.460961035	MGI:96648	Jund
ENSMUST00000107425.7	-0.460961035	MGI:1915298	Aen
ENSMUST00000148721.7	-0.460961035	MGI:1916912	2310061I04Rik
ENSMUST00000166652.1	-0.460961035	MGI:2446107	Pde2a
ENSMUST00000021114.4	-0.459285648	MGI:95730	Galk1
ENSMUST00000027989.12	-0.459285648	MGI:1330808	Hsd17b7
ENSMUST00000030872.11	-0.459285648	MGI:1347044	Orc5
ENSMUST00000040484.5	-0.459285648	MGI:1915383	Gcsh
ENSMUST00000117989.1	-0.459285648	MGI:1933212	Ngrn
ENSMUST00000166032.7	-0.459285648	MGI:1919884	Tdrkh
ENSMUST00000019470.13	-0.458745527	MGI:1096366	Psme3
ENSMUST00000005711.5	-0.456099838	MGI:1916203	Chmp2a
ENSMUST00000029499.14	-0.456099838	MGI:2442535	Slc6a17
ENSMUST00000102655.9	-0.456099838	MGI:1201792	Pde1a
ENSMUST00000006478.9	-0.455314123	MGI:1915011	Tmem147
ENSMUST00000226208.2	-0.455314123	MGI:1096572	Zfand3
ENSMUST00000230614.1	-0.455314123	MGI:1925868	Acap2
ENSMUST00000033866.8	-0.45519284	MGI:1917410	Vps36
ENSMUST00000000642.10	-0.45299636	MGI:1315197	Hk2
ENSMUST00000001148.10	-0.45299636	MGI:1890470	Pcbp3
ENSMUST00000011733.8	-0.45299636	MGI:1934858	Fsd1
ENSMUST00000022507.12	-0.45299636	MGI:1913895	Pspc1
ENSMUST00000030795.9	-0.45299636	MGI:1351657	Abcf2
ENSMUST00000031984.8	-0.45299636	MGI:95453	Smarcad1
ENSMUST00000047954.14	-0.45299636	MGI:106039	Dtna

ENSMUST00000066272.5	-0.45299636	MGI:1346348	Taf7
ENSMUST00000108156.8	-0.45299636	MGI:1918742	Bbs7
ENSMUST00000177236.7	-0.45299636	MGI:1914400	Rnf141
ENSMUST00000000544.11	-0.451623169	MGI:1338944	Acvr1b
ENSMUST00000093040.12	-0.451623169	MGI:105071	Rab4b
ENSMUST00000043907.13	-0.451179417	MGI:1930188	Mrps34
ENSMUST00000109142.7	-0.45074239	MGI:1891925	Hnrnph1
ENSMUST00000033933.6	-0.44924167	MGI:1915137	Saraf
ENSMUST00000038234.12	-0.448184057	MGI:1932386	Ift122
ENSMUST00000189220.6	-0.448184057	MGI:1333754	Agfg1
ENSMUST00000031131.10	-0.44670019	MGI:103149	Uchl1
ENSMUST00000003360.9	-0.446105904	MGI:1336193	Car11
ENSMUST00000028392.7	-0.446105904	MGI:1914111	Dnajc10
ENSMUST00000052670.10	-0.446105904	MGI:3026939	Amer3
ENSMUST00000057801.7	-0.446105904	MGI:104743	Kcnj4
ENSMUST00000060226.10	-0.446105904	MGI:1929269	Tmed2
ENSMUST00000082254.7	-0.446105904	MGI:1923467	Jakmip2
ENSMUST00000117766.7	-0.446105904	MGI:2137202	Mrpl1
ENSMUST00000210005.1	-0.446105904	MGI:1919030	Isyna1
ENSMUST00000230490.1	-0.446105904	MGI:103307	Cacnb3
ENSMUST00000030814.10	-0.444604625	MGI:101765	Cdk5
ENSMUST00000046963.9	-0.4441913	MGI:1346869	Map2k4
ENSMUST00000035779.14	-0.443240623	MGI:1921455	Acsl3
ENSMUST00000048409.13	-0.442318698	MGI:3583900	Elmod1
ENSMUST00000029087.3	-0.441964813	MGI:1919325	Ogfr
ENSMUST00000102536.10	-0.44067995	MGI:1914275	Rpl11
ENSMUST00000007814.9	-0.440086006	MGI:1336214	Khsrp
ENSMUST00000030538.4	-0.440086006	MGI:1194508	Ddost
ENSMUST00000068697.10	-0.440086006	MGI:1921537	Kcmf1
ENSMUST00000108052.9	-0.440086006	MGI:1919151	Fam219a
ENSMUST00000111329.7	-0.440086006	MGI:2445003	Arhgap1
ENSMUST00000116514.3	-0.440086006	MGI:1891823	Wbp11
ENSMUST00000118886.8	-0.440086006	MGI:108104	Snrk
ENSMUST00000025250.13	-0.440054043	MGI:1919439	Bag6
ENSMUST00000031061.11	-0.43914388	MGI:1099786	Dhx15
ENSMUST00000021607.8	-0.438838249	MGI:1330838	Lgmn
ENSMUST00000023112.11	-0.438686963	MGI:1353418	Pmm1
ENSMUST00000059472.9	-0.438596675	MGI:2443731	Mat2a
ENSMUST00000060232.7	-0.43849393	MGI:1928750	Rab2a

ENSMUST00000078272.12	-0.436786484	MGI:88295	Cacna2d1
ENSMUST00000076325.11	-0.436492292	MGI:99532	Mef2a
ENSMUST00000088512.12	-0.436492292	MGI:97960	Rnps1
ENSMUST00000224188.1	-0.435755488	MGI:107592	Hmgcs1
ENSMUST00000023360.13	-0.434781442	MGI:2135951	Mpv17l
ENSMUST00000032443.13	-0.434781442	MGI:2687035	Far2
ENSMUST00000037843.6	-0.434781442	MGI:1916255	Ubald1
ENSMUST00000112304.9	-0.434781442	MGI:101841	Ppm1b
ENSMUST00000153609.7	-0.434781442	MGI:1916231	Snrpa1
ENSMUST00000061427.9	-0.43399524	MGI:3045353	Adamts3
ENSMUST00000025364.5	-0.431639593	MGI:1914430	Yipf5
ENSMUST00000072376.12	-0.431639593	MGI:1929668	Rnf14
ENSMUST00000005826.8	-0.431435102	MGI:88529	Cs
ENSMUST00000047935.7	-0.430528814	MGI:106393	Tspyl4
ENSMUST00000002080.11	-0.43007183	MGI:2443483	Pdzd4
ENSMUST00000038403.11	-0.43007183	MGI:96391	Ica1
ENSMUST00000086635.8	-0.43007183	MGI:1927542	Pitpnb
ENSMUST00000102962.9	-0.43007183	MGI:99605	Cntfr
ENSMUST00000102980.10	-0.43007183	MGI:1353494	Akr1b3
ENSMUST00000125304.7	-0.43007183	MGI:2443010	Lman2l
ENSMUST00000125366.7	-0.43007183	MGI:95886	H13
ENSMUST00000136983.7	-0.43007183	MGI:109529	Dpf2
ENSMUST00000163528.7	-0.43007183	MGI:1918928	Brox
ENSMUST00000163571.7	-0.43007183	MGI:894645	Pick1
ENSMUST00000103172.3	-0.427361525	MGI:1929270	Dstn
ENSMUST00000005714.13	-0.427307034	MGI:108278	Ube2m
ENSMUST00000033805.14	-0.427307034	MGI:1858898	Psmd10
ENSMUST00000028062.7	-0.426439088	MGI:98932	Vim
ENSMUST00000003710.9	-0.42586237	MGI:1098687	Aak1
ENSMUST00000025472.6	-0.42586237	MGI:3606062	Pcyox1l
ENSMUST00000030360.10	-0.42586237	MGI:1925059	Lrrc42
ENSMUST00000034437.7	-0.42586237	MGI:2442115	Wdr59
ENSMUST00000041685.6	-0.42586237	MGI:107755	Neurod2
ENSMUST00000053917.5	-0.42586237	MGI:1915224	Ccny
ENSMUST00000056521.11	-0.42586237	MGI:1914504	Bmerb1
ENSMUST00000093552.11	-0.42586237	MGI:1858259	Tomm40
ENSMUST00000162301.7	-0.42586237	MGI:1918995	Cul2
ENSMUST00000114551.9	-0.42531035	MGI:1347085	Cetn2
ENSMUST00000064667.8	-0.423866424	MGI:894315	Rap1b

ENSMUST00000028624.8	-0.42316862	MGI:1914342	Gatm
ENSMUST00000228009.1	-0.422267244	MGI:99670	Dmtn
ENSMUST00000022013.7	-0.422077392	MGI:99676	Adcy2
ENSMUST00000094028.9	-0.422077392	MGI:106227	Capza1
ENSMUST00000102875.10	-0.422077392	MGI:2183447	Ugp2
ENSMUST00000111915.7	-0.422077392	MGI:1921775	Fam234b
ENSMUST00000210135.1	-0.422077392	MGI:1098749	Erc2
ENSMUST00000227556.1	-0.422077392	MGI:2444173	Lmbrd2
ENSMUST00000067384.5	-0.421614101	MGI:107949	Rhob
ENSMUST00000170836.3	-0.420530208	MGI:104885	Psma2
ENSMUST00000037547.8	-0.419900153	MGI:2388733	Disp2
ENSMUST00000026879.7	-0.418655758	MGI:1338002	Gdap1
ENSMUST00000030453.4	-0.418655758	MGI:1914346	Mmachc
ENSMUST00000064503.12	-0.418655758	MGI:1918115	Lnpk
ENSMUST00000097495.4	-0.418655758	MGI:3639495	Dok6
ENSMUST00000110748.3	-0.418655758	MGI:104750	Nrcam
ENSMUST00000141116.1	-0.418655758	MGI:1346320	Taf10
ENSMUST00000166123.8	-0.418655758	MGI:95309	Eif5
ENSMUST00000055506.8	-0.415547538	MGI:107887	Gtf3c1
ENSMUST00000188347.6	-0.415547538	MGI:2138446	Atg9a
ENSMUST00000085248.11	-0.413800685	MGI:1096551	Morf4l1
ENSMUST00000210999.1	-0.413800685	MGI:1929264	Sae1
ENSMUST00000007042.5	-0.412711548	MGI:1345142	Ik
ENSMUST00000059680.6	-0.412711548	MGI:1913879	Golph3
ENSMUST00000066597.12	-0.412711548	MGI:2443079	Klhl26
ENSMUST00000110325.7	-0.412711548	MGI:1919727	Tmem87b
ENSMUST00000112532.7	-0.412711548	MGI:1098808	Pex5
ENSMUST00000147695.8	-0.412711548	MGI:1920209	Lonrf2
ENSMUST00000149441.7	-0.412711548	MGI:1915297	Mpnd
ENSMUST00000235966.1	-0.412351716	MGI:98107	Rps14
ENSMUST00000037401.9	-0.41011352	MGI:2444148	Phf20
ENSMUST00000075896.6	-0.41011352	MGI:1855672	Tsnax
ENSMUST00000077595.11	-0.41011352	MGI:1890212	Porcn
ENSMUST00000238823.1	-0.41011352	MGI:2442333	Celf5
ENSMUST00000049152.14	-0.409178499	MGI:1919232	Snx10
ENSMUST00000001809.14	-0.407724706	MGI:1349722	Pabpc1
ENSMUST00000025617.3	-0.407724706	MGI:1914688	Rfk
ENSMUST00000038521.13	-0.407724706	MGI:1913328	Tsen34
ENSMUST00000045376.10	-0.407724706	MGI:87930	Adk

ENSMUST00000049621.6	-0.407724706	MGI:104876	Hes5
ENSMUST00000020991.14	-0.407539878	MGI:1261827	Dnmt3a
ENSMUST00000103147.4	-0.406434094	MGI:1347014	Psmb3
ENSMUST00000033383.2	-0.405520809	MGI:2384312	Usp11
ENSMUST00000103123.9	-0.405520809	MGI:1917720	Rprd1b
ENSMUST00000035804.8	-0.405370574	MGI:105925	Cdo1
ENSMUST00000021375.11	-0.403786889	MGI:1349635	Sec23a
ENSMUST00000033093.9	-0.403481156	MGI:99702	Bax
ENSMUST00000037748.8	-0.403481156	MGI:1858195	Hnrnpu
ENSMUST00000020008.9	-0.401588044	MGI:2149946	Gopc
ENSMUST00000041401.10	-0.401588044	MGI:1921248	Herc3
ENSMUST00000079490.5	-0.401588044	MGI:1859565	Nap1l3
ENSMUST00000223690.1	-0.401588044	MGI:1855699	Vti1a
ENSMUST00000233645.1	-0.401588044	MGI:97837	Qk
ENSMUST00000025542.9	-0.401494112	MGI:2183441	Psat1
ENSMUST00000018470.9	-0.401004726	MGI:1891917	Ywhab
ENSMUST00000025707.8	-0.398182503	MGI:1891017	Zfpl1
ENSMUST00000027623.8	-0.398182503	MGI:109263	Tsn
ENSMUST00000102661.3	-0.398182503	MGI:106611	Rnf112
ENSMUST00000155282.8	-0.398182503	MGI:105976	Myo5a
ENSMUST00000032413.6	-0.398131695	MGI:1922570	Etnk1
ENSMUST00000065987.13	-0.398115003	MGI:2443582	Abat
ENSMUST00000018691.8	-0.396645382	MGI:1934234	Pip4k2b
ENSMUST00000103225.10	-0.396645382	MGI:1917841	5730455P16Rik
ENSMUST00000166126.6	-0.396645382	MGI:1338042	Pcdh10
ENSMUST00000023707.10	-0.396612324	MGI:98351	Sod1
ENSMUST00000028916.14	-0.395204819	MGI:1350925	Sec23b
ENSMUST00000026122.10	-0.393851991	MGI:97464	P4hb
ENSMUST00000107196.9	-0.393851991	MGI:1344351	Dlg2
ENSMUST00000076454.7	-0.39342013	MGI:1860276	Ubqln1
ENSMUST00000019109.7	-0.393163084	MGI:109194	Ywhah
ENSMUST00000004172.14	-0.391984839	MGI:109373	Hmox2
ENSMUST00000131475.1	-0.391379321	MGI:95700	Ggct
ENSMUST00000045602.8	-0.390732671	MGI:1915592	Ndufb10
ENSMUST00000107924.1	-0.389235565	MGI:1916477	Selenot
ENSMUST00000084526.11	-0.389175133	MGI:1333843	Slc31a1
ENSMUST00000094578.10	-0.389175133	MGI:1916412	Sec31a
ENSMUST00000106267.4	-0.388206079	MGI:1930705	Stx1b
ENSMUST00000029447.11	-0.388160416	MGI:1913891	Sike1

ENSMUST00000034048.12	-0.387197949	MGI:1914006	Cfap97
ENSMUST00000026120.7	-0.386790746	MGI:1930001	Bhlhe22
ENSMUST00000236889.1	-0.385414416	MGI:1914659	Kctd16
ENSMUST00000028398.13	-0.384947815	MGI:107412	Ube2e3
ENSMUST00000232139.1	-0.383797412	MGI:109240	Ube2l3
ENSMUST00000076155.5	-0.383167401	MGI:3648653	Eno1b
ENSMUST00000046945.12	-0.383044236	MGI:1261814	Palm
ENSMUST00000102573.7	-0.383044236	MGI:1931835	Trim44
ENSMUST00000084500.7	-0.382324653	MGI:97394	Oat
ENSMUST00000222404.1	-0.382324653	MGI:3582925	Tes3-ps
ENSMUST00000065917.15	-0.378696725	MGI:1855693	Nap1l1
ENSMUST00000045557.9	-0.377548475	MGI:1298205	Slc7a5
ENSMUST00000078139.12	-0.377548475	MGI:1914186	Asph
ENSMUST00000170715.7	-0.376572157	MGI:105110	Rps2
ENSMUST00000003971.9	-0.376110224	MGI:1330858	Lin7b
ENSMUST00000089230.6	-0.375234274	MGI:1919228	Ppp2r2a
ENSMUST00000020308.4	-0.373756656	MGI:1921997	Ddit4
ENSMUST00000076354.12	-0.366014176	MGI:1298407	Tspan7
ENSMUST00000004206.9	-0.364400061	MGI:1858258	Eif3g
ENSMUST00000169613.3	-0.359682826	MGI:95865	Gstp1
ENSMUST00000041606.13	-0.350495169	MGI:1916602	Necab1
ENSMUST00000139395.7	-0.350355356	MGI:1933548	Actl6b
ENSMUST00000007212.8	-0.350335422	MGI:1096584	Psmd2
ENSMUST00000049948.5	-0.350262417	MGI:1913764	Asrgl1
ENSMUST00000041736.10	-0.350242784	MGI:2385252	Hdac11
ENSMUST00000113607.9	-0.350232394	MGI:1858696	Copg1
ENSMUST00000090553.11	-0.350160432	MGI:102809	Sars
ENSMUST00000026667.14	-0.350132018	MGI:1923731	Eif4a3
ENSMUST00000026506.4	-0.350116804	MGI:109638	Clns1a
ENSMUST00000033184.5	-0.350100855	MGI:1336194	Tpp1
ENSMUST00000046518.11	-0.350100855	MGI:2446159	Itpk1
ENSMUST00000053015.6	-0.350100855	MGI:1345635	Pcbp1
ENSMUST00000131996.7	-0.350084114	MGI:1927469	Rgs17
ENSMUST00000029382.12	-0.350066523	MGI:1914988	Ppid
ENSMUST00000029876.1	-0.350048014	MGI:88248	Calb1
ENSMUST00000227691.1	-0.350007942	MGI:1859920	Ebag9
ENSMUST00000027475.14	-0.349986205	MGI:2138584	Gigyf2
ENSMUST00000044382.6	-0.349885388	MGI:2679294	Zc4h2
ENSMUST00000163785.1	-0.349885388	MGI:88396	Chrm1

ENSMUST00000080299.6	-0.349856031	MGI:1914307	Yaf2
ENSMUST00000109627.7	-0.349856031	MGI:3512628	Cbx6
ENSMUST00000131252.7	-0.349856031	MGI:1095403	Sf1
ENSMUST00000086471.11	-0.349791103	MGI:1919204	Suds3
ENSMUST00000236560.1	-0.349791103	MGI:6303291	Gm50388
ENSMUST00000022639.7	-0.349755077	MGI:97313	Nefl
ENSMUST00000026324.9	-0.349755077	MGI:1928939	Acot9
ENSMUST00000099735.5	-0.349755077	MGI:1914258	Yae1d1
ENSMUST00000172164.9	-0.349755077	MGI:2146512	Slc7a4
ENSMUST00000029666.13	-0.349716317	MGI:1330587	Papss1
ENSMUST00000076615.5	-0.349716317	MGI:2142523	Crtc1
ENSMUST00000038379.4	-0.349674498	MGI:1917826	Cpsf7
ENSMUST00000049411.11	-0.349674498	MGI:88042	Apex1
ENSMUST00000076670.2	-0.349674498	MGI:2445015	Plppr1
ENSMUST00000013807.7	-0.349629244	MGI:109583	Pten
ENSMUST00000022164.15	-0.349629244	MGI:1915808	Ankra2
ENSMUST00000033824.7	-0.349629244	MGI:96745	Lamp1
ENSMUST00000052556.4	-0.349629244	MGI:1917816	Abhd17b
ENSMUST00000055704.11	-0.349629244	MGI:95772	Gnai2
ENSMUST00000028238.14	-0.349580114	MGI:1915615	Rab14
ENSMUST00000053087.3	-0.349580114	MGI:2683536	Klhl23
ENSMUST00000062213.12	-0.349580114	MGI:2137679	Sfxn3
ENSMUST00000065957.6	-0.349580114	MGI:1926368	Syt5
ENSMUST00000172107.7	-0.349580114	MGI:1917606	St13
ENSMUST00000232437.1	-0.349580114	MGI:1345181	Pacsin1
ENSMUST00000009679.10	-0.349526586	MGI:1915147	Rnmt
ENSMUST00000109523.1	-0.349526586	MGI:2685537	Vstm2l
ENSMUST00000114839.7	-0.349526586	MGI:1344381	Dnajb6
ENSMUST00000118199.7	-0.349526586	MGI:1915148	Pef1
ENSMUST00000150268.7	-0.349526586	MGI:1858197	Plrg1
ENSMUST00000165123.7	-0.349526586	MGI:1934950	Csnk1a1
ENSMUST00000000260.12	-0.349468042	MGI:1913605	Gmpr
ENSMUST00000024748.13	-0.349468042	MGI:1860138	Gtpbp2
ENSMUST00000050328.14	-0.349468042	MGI:1349431	Eif2s3x
ENSMUST00000056034.12	-0.349468042	MGI:1913869	Atat1
ENSMUST00000060904.10	-0.349468042	MGI:1913354	Tceal3
ENSMUST00000063574.6	-0.349468042	MGI:1915577	Tsr3
ENSMUST00000064571.10	-0.349468042	MGI:1917542	Afap1
ENSMUST00000072113.5	-0.349468042	MGI:1922118	Tmem65

ENSMUST00000115645.9	-0.349468042	MGI:96269	Ranbp1
ENSMUST00000210640.1	-0.349468042	MGI:98003	Rpl18
ENSMUST00000023294.14	-0.349403744	MGI:1915958	Rabl2
ENSMUST00000023818.10	-0.349403744	MGI:1914738	Calcoco1
ENSMUST00000025408.9	-0.349403744	MGI:1916847	Afg3l2
ENSMUST00000028977.6	-0.349403744	MGI:107688	Kif3b
ENSMUST00000045004.10	-0.349403744	MGI:1917278	Dop1b
ENSMUST00000050516.13	-0.349403744	MGI:1916340	Ascc1
ENSMUST00000084770.4	-0.349403744	MGI:1330859	Fut9
ENSMUST00000161204.7	-0.349403744	MGI:102581	Rdh11
ENSMUST00000162364.7	-0.349403744	MGI:104899	Mob4
ENSMUST00000021689.13	-0.349332799	MGI:1194884	Evl
ENSMUST00000046770.9	-0.349332799	MGI:1913458	Nenf
ENSMUST00000067081.9	-0.349332799	MGI:88353	Cdk11b
ENSMUST00000084524.3	-0.349332799	MGI:1917302	Prpf4
ENSMUST00000103099.7	-0.349332799	MGI:108498	Nbr1
ENSMUST00000122965.7	-0.349332799	MGI:1925016	Elp4
ENSMUST00000153183.7	-0.349332799	MGI:1351652	Tbl2
ENSMUST00000009174.14	-0.34925412	MGI:1914716	Pdcl
ENSMUST00000038422.7	-0.34925412	MGI:2442463	Irf2bpl
ENSMUST00000049977.12	-0.34925412	MGI:1352748	Dpf1
ENSMUST00000056403.6	-0.34925412	MGI:2685307	H1f10
ENSMUST00000058981.2	-0.34925412	MGI:107633	Lxn
ENSMUST00000000080.7	-0.34916637	MGI:1346318	Klf6
ENSMUST00000007253.5	-0.34916637	MGI:97305	Neu1
ENSMUST00000026896.9	-0.34916637	MGI:106020	St8sia2
ENSMUST00000029336.5	-0.34916637	MGI:1919412	Dhx36
ENSMUST00000034983.6	-0.34916637	MGI:107788	Atp1b3
ENSMUST00000069106.4	-0.34916637	MGI:1341085	Epm2a
ENSMUST00000078944.12	-0.34916637	MGI:1918248	Phf6
ENSMUST00000096441.4	-0.34916637	MGI:1921772	Morc2a
ENSMUST00000114726.7	-0.34916637	MGI:1343044	Rbmx
ENSMUST00000151224.2	-0.34916637	MGI:1926106	Fam163b
ENSMUST00000051100.6	-0.34916637	NA	NA
ENSMUST00000000001.4	-0.349067883	MGI:95773	Gnai3
ENSMUST00000026937.11	-0.349067883	MGI:1913633	Iscu
ENSMUST00000032715.12	-0.349067883	MGI:1919224	Prmt3
ENSMUST00000049997.13	-0.349067883	MGI:2181363	Prokr2
ENSMUST00000058162.13	-0.349067883	MGI:96911	Mafg

ENSMUST00000060442.13	-0.349067883	MGI:1927851	Gpr85
ENSMUST00000061280.16	-0.349067883	MGI:2441773	Pcmtd1
ENSMUST00000062957.7	-0.349067883	MGI:2144724	Ttc7b
ENSMUST00000072451.10	-0.349067883	MGI:2444530	Slc9a7
ENSMUST00000121153.7	-0.349067883	MGI:1342291	Rlim
ENSMUST00000162517.7	-0.349067883	MGI:1920094	Kctd17
ENSMUST00000218004.1	-0.349067883	MGI:1920442	Xpot
ENSMUST00000008748.7	-0.34895656	MGI:2150152	Ubqln4
ENSMUST00000018727.3	-0.34895656	MGI:1351465	G3bp1
ENSMUST00000024885.9	-0.34895656	MGI:109386	Cebpz
ENSMUST00000026428.3	-0.34895656	MGI:1917789	Myl6b
ENSMUST00000034827.9	-0.34895656	MGI:1916119	Imp3
ENSMUST00000040008.3	-0.34895656	MGI:1914865	Ube2r2
ENSMUST00000055946.7	-0.34895656	MGI:2441697	Gpr158
ENSMUST00000064062.12	-0.34895656	MGI:1923864	Immt
ENSMUST00000102997.7	-0.34895656	MGI:1858199	Ccnc
ENSMUST00000111178.1	-0.34895656	MGI:2444851	Efr3b
ENSMUST00000115733.2	-0.34895656	MGI:2660877	Gucy1a2
ENSMUST00000169611.3	-0.34895656	MGI:97239	Mmut
ENSMUST00000179549.2	-0.34895656	MGI:2147607	Ccdc85b
ENSMUST00000217267.1	-0.34895656	MGI:2180854	Rfxap
ENSMUST00000002099.10	-0.348829718	MGI:1923818	Ift46
ENSMUST00000002885.7	-0.348829718	MGI:2145369	Epdr1
ENSMUST00000016553.4	-0.348829718	MGI:1914300	Nkap
ENSMUST00000016680.13	-0.348829718	MGI:1914487	Cul4a
ENSMUST00000019931.11	-0.348829718	MGI:2442989	Lrp11
ENSMUST00000022196.4	-0.348829718	MGI:1333879	Ap3b1
ENSMUST00000027123.14	-0.348829718	MGI:96242	Hspd1
ENSMUST00000029575.11	-0.348829718	MGI:1889574	Extl2
ENSMUST00000033727.13	-0.348829718	MGI:1933185	Ctps2
ENSMUST00000038166.8	-0.348829718	MGI:2147067	Dhx57
ENSMUST00000047057.8	-0.348829718	MGI:4820566	Gm17018
ENSMUST00000047521.6	-0.348829718	MGI:2139134	Cercam
ENSMUST00000048421.13	-0.348829718	MGI:2385896	Map11
ENSMUST00000049740.2	-0.348829718	MGI:1927290	Zbtb33
ENSMUST00000056508.11	-0.348829718	MGI:103555	Clcn3
ENSMUST00000076110.10	-0.348829718	MGI:1101759	Rnf2
ENSMUST00000095263.9	-0.348829718	MGI:106280	Yipf3
ENSMUST00000103081.9	-0.348829718	MGI:1098667	Adam11

ENSMUST00000109260.2	-0.348829718	MGI:2144243	Clint1
ENSMUST00000111325.4	-0.348829718	MGI:1916876	Sdr39u1
ENSMUST00000127698.7	-0.348829718	MGI:1339946	Tbpl1
ENSMUST00000167549.1	-0.348829718	MGI:1919196	Endod1
ENSMUST00000177633.7	-0.348829718	MGI:1918025	Zmynd8
ENSMUST00000017384.13	-0.348683868	MGI:2179723	Casc3
ENSMUST00000023099.7	-0.348683868	MGI:1915010	Slc38a2
ENSMUST00000028341.10	-0.348683868	MGI:2139135	Anapc2
ENSMUST00000028536.12	-0.348683868	MGI:1926020	Arl14ep
ENSMUST00000029199.10	-0.348683868	MGI:1195270	Zmat3
ENSMUST00000032963.9	-0.348683868	MGI:1919840	Ppme1
ENSMUST00000033875.9	-0.348683868	MGI:1891207	Plpbp
ENSMUST00000041374.7	-0.348683868	MGI:2444484	Manea
ENSMUST00000042750.2	-0.348683868	MGI:1914313	Tmem246
ENSMUST00000044113.11	-0.348683868	MGI:2446632	Ago2
ENSMUST00000086559.6	-0.348683868	MGI:2444823	Slc41a1
ENSMUST00000094434.12	-0.348683868	MGI:95589	Ftl1
ENSMUST00000105851.8	-0.348683868	MGI:1913443	Pithd1
ENSMUST00000113360.7	-0.348683868	MGI:107438	Cab39
ENSMUST00000131309.2	-0.348683868	MGI:108476	Fzd3
ENSMUST00000140901.7	-0.348683868	MGI:1926790	Fzr1
ENSMUST00000160597.7	-0.348683868	MGI:1921393	Opa1
ENSMUST00000170883.7	-0.348683868	MGI:99256	Hdlbp
ENSMUST00000202406.3	-0.348683868	MGI:102788	Rph3a
ENSMUST00000221368.1	-0.348683868	MGI:1917623	Gpatch2l
ENSMUST00000001202.14	-0.348514392	MGI:109589	Ocrl
ENSMUST00000015333.11	-0.348514392	MGI:2384865	Casd1
ENSMUST00000019945.14	-0.348514392	MGI:1929646	Pex3
ENSMUST00000026408.6	-0.348514392	MGI:1338027	Gdf11
ENSMUST00000028763.9	-0.348514392	MGI:104294	Tyro3
ENSMUST00000035350.11	-0.348514392	MGI:1923012	Aftph
ENSMUST00000035934.6	-0.348514392	MGI:2443972	Exoc3
ENSMUST00000042608.7	-0.348514392	MGI:87873	Acd
ENSMUST00000042614.12	-0.348514392	MGI:3647820	Hectd4
ENSMUST00000042661.7	-0.348514392	MGI:1925589	Ttyh3
ENSMUST00000048657.9	-0.348514392	MGI:1919746	Sec24c
ENSMUST00000061882.9	-0.348514392	MGI:2388651	Mcat
ENSMUST00000069250.13	-0.348514392	MGI:99204	Zfp57
ENSMUST00000090558.9	-0.348514392	MGI:1858235	Celsr2

ENSMUST00000110648.7	-0.348514392	MGI:1351320	Trp53bp1
ENSMUST00000121927.7	-0.348514392	MGI:1315213	Nrip1
ENSMUST00000130044.1	-0.348514392	MGI:1099446	Synpo
ENSMUST00000165978.2	-0.348514392	MGI:2144865	Tecpr2
ENSMUST00000198254.4	-0.348514392	MGI:1859730	Sh3glb1
ENSMUST00000201203.3	-0.348514392	MGI:1916046	Tmem214
ENSMUST00000215420.1	-0.348514392	MGI:1316650	Slc37a4
ENSMUST00000000641.14	-0.348315041	MGI:1340055	Sema4f
ENSMUST00000001412.16	-0.348315041	MGI:1920538	Vps50
ENSMUST00000006991.8	-0.348315041	MGI:1096392	Hcn1
ENSMUST00000016696.12	-0.348315041	MGI:106315	Foxred2
ENSMUST00000022224.15	-0.348315041	MGI:1914225	Trappc13
ENSMUST00000022819.12	-0.348315041	MGI:2443113	Jph4
ENSMUST00000023913.10	-0.348315041	MGI:2136882	Get1
ENSMUST00000026243.4	-0.348315041	MGI:1932139	Oga
ENSMUST00000028160.14	-0.348315041	MGI:1915913	Slc25a25
ENSMUST00000029773.12	-0.348315041	MGI:1919244	Cnn3
ENSMUST00000031695.14	-0.348315041	MGI:1920428	Wasl
ENSMUST00000032179.13	-0.348315041	MGI:1859555	Nup210
ENSMUST00000035991.7	-0.348315041	MGI:2179809	Bivm
ENSMUST00000045270.14	-0.348315041	MGI:1333833	Cbfa2t2
ENSMUST00000049822.9	-0.348315041	MGI:1923028	Them4
ENSMUST00000057717.7	-0.348315041	MGI:1890618	Zfp319
ENSMUST00000067567.4	-0.348315041	MGI:1347000	Lypla2
ENSMUST00000075448.12	-0.348315041	MGI:108056	Nfia
ENSMUST00000091259.8	-0.348315041	MGI:3040688	Slc7a14
ENSMUST00000093321.11	-0.348315041	MGI:103232	Grb10
ENSMUST00000099090.6	-0.348315041	MGI:1919283	Tsc22d2
ENSMUST00000103064.9	-0.348315041	MGI:1919045	Pitpnc1
ENSMUST00000103133.3	-0.348315041	MGI:1927347	Smarce1
ENSMUST00000106657.7	-0.348315041	MGI:2652838	Zfyve9
ENSMUST00000109553.9	-0.348315041	MGI:98663	Tef
ENSMUST00000110255.7	-0.348315041	MGI:1920175	Marchf1
ENSMUST00000111546.7	-0.348315041	MGI:1194500	Ablim1
ENSMUST00000112115.1	-0.348315041	MGI:106911	Hccs
ENSMUST00000120796.7	-0.348315041	MGI:3036273	Kcnt2
ENSMUST00000161263.7	-0.348315041	MGI:1922459	Sv2c
ENSMUST00000178687.1	-0.348315041	MGI:2136977	Tmem121b
ENSMUST00000179636.2	-0.348315041	MGI:2143484	Slc6a15

ENSMUST00000238290.1	-0.348315041	MGI:1920082	Crtac1
ENSMUST00000005841.15	-0.348077155	MGI:109447	Ctcf
ENSMUST00000006893.8	-0.348077155	MGI:3036268	D130043K22Rik
ENSMUST00000011450.7	-0.348077155	MGI:1917866	Sugp1
ENSMUST00000020490.12	-0.348077155	MGI:1924555	Wdr82
ENSMUST00000021898.5	-0.348077155	MGI:106179	Shc3
ENSMUST00000022358.8	-0.348077155	MGI:1919156	Zswim8
ENSMUST00000025393.13	-0.348077155	MGI:894293	Smad4
ENSMUST00000025486.8	-0.348077155	MGI:96795	Lmnb1
ENSMUST00000027797.8	-0.348077155	MGI:1914709	Nvl
ENSMUST00000030340.14	-0.348077155	MGI:98254	Scp2
ENSMUST00000032410.13	-0.348077155	MGI:1915724	Tada3
ENSMUST00000033992.8	-0.348077155	MGI:95804	Gsr
ENSMUST00000038627.8	-0.348077155	MGI:104854	Zfp91
ENSMUST00000043349.6	-0.348077155	MGI:95812	Grid1
ENSMUST00000056237.14	-0.348077155	MGI:2142651	Prmt9
ENSMUST00000058578.7	-0.348077155	MGI:1918054	Pgrmc2
ENSMUST00000068301.10	-0.348077155	MGI:2139793	Cept1
ENSMUST00000077477.11	-0.348077155	MGI:1860085	Stk19
ENSMUST00000102574.9	-0.348077155	MGI:895149	Acadvl
ENSMUST00000105502.7	-0.348077155	MGI:1890081	Foxo3
ENSMUST00000105778.7	-0.348077155	MGI:107628	Prdm2
ENSMUST00000106061.8	-0.348077155	MGI:1921898	S100pbp
ENSMUST00000113462.7	-0.348077155	MGI:1096362	Nrxn2
ENSMUST00000118831.7	-0.348077155	MGI:99665	Syt3
ENSMUST00000121390.7	-0.348077155	MGI:109482	Cacna1a
ENSMUST00000143400.7	-0.348077155	MGI:1925791	Asb8
ENSMUST00000151477.7	-0.348077155	MGI:3648294	Tnrc18
ENSMUST00000162751.7	-0.348077155	MGI:1352508	Stau2
ENSMUST00000165838.8	-0.348077155	MGI:1917333	Metrn
ENSMUST00000168381.7	-0.348077155	MGI:1916417	Ppp1r12b
ENSMUST00000170792.8	-0.348077155	MGI:1346863	Mapk10
ENSMUST00000183663.7	-0.348077155	MGI:96560	Il6st
ENSMUST00000193404.1	-0.348077155	MGI:1935227	Pcdhga10
ENSMUST00000229441.1	-0.348077155	MGI:3608416	Elfn2
ENSMUST00000003947.8	-0.347788363	MGI:103187	Nqo1
ENSMUST00000005057.6	-0.347788363	MGI:1354165	Thop1
ENSMUST00000018805.14	-0.347788363	MGI:1333873	Cog1
ENSMUST00000021824.7	-0.347788363	MGI:1918180	Nol8

ENSMUST00000022419.6	-0.347788363	MGI:2145814	Ppif
ENSMUST00000023562.8	-0.347788363	MGI:1913914	Tmem41a
ENSMUST00000023758.8	-0.347788363	MGI:1194915	Asic1
ENSMUST00000024575.7	-0.347788363	MGI:1342290	Rps6ka2
ENSMUST00000025910.12	-0.347788363	MGI:1096330	Bad
ENSMUST00000026671.12	-0.347788363	MGI:1921620	Rptor
ENSMUST00000028049.12	-0.347788363	MGI:1352507	Rabgap1l
ENSMUST00000031289.6	-0.347788363	MGI:1917368	Srrd
ENSMUST00000031740.15	-0.347788363	MGI:106477	Mepce
ENSMUST00000032071.12	-0.347788363	MGI:1919352	Dusp11
ENSMUST00000033086.7	-0.347788363	MGI:1916211	Phkg2
ENSMUST00000033965.13	-0.347788363	MGI:1345183	Tenm3
ENSMUST00000034720.11	-0.347788363	MGI:107282	Polr2m
ENSMUST00000034945.5	-0.347788363	MGI:1915500	Ciao2a
ENSMUST00000035661.6	-0.347788363	MGI:2153093	Cspg4
ENSMUST00000039750.6	-0.347788363	MGI:2180196	Lgi2
ENSMUST00000041733.8	-0.347788363	MGI:2443028	Taf2
ENSMUST00000044207.4	-0.347788363	MGI:1309453	Sart1
ENSMUST00000047247.11	-0.347788363	MGI:2442663	Hectd2
ENSMUST00000048377.10	-0.347788363	MGI:2138346	Suco
ENSMUST00000050211.6	-0.347788363	MGI:1913266	Tk2
ENSMUST00000053959.6	-0.347788363	MGI:1202397	Ints6
ENSMUST00000057272.14	-0.347788363	MGI:2444631	4932438A13Rik
ENSMUST00000060077.6	-0.347788363	MGI:104841	Cpox
ENSMUST00000060992.5	-0.347788363	MGI:1915835	Rtn4
ENSMUST00000065932.13	-0.347788363	MGI:1859616	Gripap1
ENSMUST00000066149.8	-0.347788363	MGI:1935221	Pcdhga8
ENSMUST00000067880.12	-0.347788363	MGI:109548	Adam10
ENSMUST00000072031.12	-0.347788363	MGI:1349438	Mprip
ENSMUST00000073957.7	-0.347788363	MGI:1340034	Sema3e
ENSMUST00000079121.3	-0.347788363	MGI:1914931	Mrpl18
ENSMUST00000084891.4	-0.347788363	MGI:1924399	Pacs2
ENSMUST00000087321.3	-0.347788363	MGI:1858229	Ppp1r3c
ENSMUST00000090680.10	-0.347788363	MGI:1858415	Ddx20
ENSMUST00000093450.5	-0.347788363	MGI:2687327	Ano8
ENSMUST00000098444.8	-0.347788363	MGI:1927223	Pard6a
ENSMUST00000098789.4	-0.347788363	MGI:2682314	Zc3h4
ENSMUST00000101191.9	-0.347788363	MGI:1919028	Klhl5
ENSMUST00000102729.9	-0.347788363	MGI:104583	Eps15

ENSMUST00000102799.9	-0.347788363	MGI:1100887	Elavl2
ENSMUST00000103111.8	-0.347788363	MGI:2444772	Zhx3
ENSMUST00000105646.2	-0.347788363	MGI:2685419	Ajap1
ENSMUST00000108022.7	-0.347788363	MGI:2444848	Ptrh2
ENSMUST00000109426.2	-0.347788363	MGI:1346016	Arhgef28
ENSMUST00000112819.8	-0.347788363	MGI:2147870	Lrch2
ENSMUST00000113952.9	-0.347788363	MGI:2155884	Wdr5
ENSMUST00000119142.7	-0.347788363	MGI:104771	Erbb4
ENSMUST00000125871.7	-0.347788363	MGI:2145310	Rnf44
ENSMUST00000129321.1	-0.347788363	MGI:1915359	Rnf227
ENSMUST00000130300.2	-0.347788363	MGI:2445053	Ccbe1
ENSMUST00000135431.7	-0.347788363	MGI:2442636	Lrrc4c
ENSMUST00000159405.2	-0.347788363	MGI:104692	Pcdh1
ENSMUST00000164099.2	-0.347788363	MGI:1921581	Snx19
ENSMUST00000168621.2	-0.347788363	MGI:104574	Ptprj
ENSMUST00000177978.2	-0.347788363	MGI:3714351	Fam177a2
ENSMUST00000191428.6	-0.347788363	MGI:109168	Fat1
ENSMUST00000194418.1	-0.347788363	MGI:1935216	Pcdhga4
ENSMUST00000208247.2	-0.347788363	MGI:105376	Adam9
ENSMUST00000210279.1	-0.347788363	MGI:2684420	Dcaf15
ENSMUST00000236152.1	-0.347788363	MGI:1345278	Slc29a2
ENSMUST00000237592.1	-0.347788363	MGI:4937091	Snhg4
ENSMUST00000237592.1	-0.347788363	MGI:4937091	Snhg4
ENSMUST00000237592.1	-0.347788363	MGI:4937091	Snhg4
ENSMUST00000237592.1	-0.347788363	MGI:4937091	Snhg4
ENSMUST00000071135.5	-0.337673562	MGI:107848	Tubb4a
ENSMUST00000019649.3	-0.33719953	MGI:98888	Ubb
ENSMUST00000020608.2	-0.336588812	MGI:1321159	Ppp2ca
ENSMUST00000069520.10	-0.331386464	MGI:98467	Syp
ENSMUST00000034097.7	-0.328775778	MGI:95792	Got2
ENSMUST00000084882.8	-0.327586524	MGI:1915587	Crip2
ENSMUST00000102645.3	-0.325784724	MGI:1914234	Med19
ENSMUST00000044911.9	-0.323463952	MGI:1891731	Stub1
ENSMUST00000072359.7	-0.323380804	MGI:99705	Tpm3-rs7
ENSMUST00000102683.10	-0.322410296	MGI:2443306	Tom1l2
ENSMUST00000002818.8	-0.321705403	MGI:1927550	Ykt6
ENSMUST00000008812.8	-0.321272078	MGI:98146	Rps18
ENSMUST00000005647.3	-0.321152163	MGI:1915599	Ndufs3
ENSMUST00000017458.10	-0.320038695	MGI:1858257	Mpp2

ENSMUST00000032078.8	-0.319626474	MGI:107184	Cct7
ENSMUST00000106008.2	-0.319382506	MGI:2387178	Gatd1
ENSMUST00000103124.10	-0.318697688	MGI:1261831	Hap1
ENSMUST00000069580.11	-0.317982325	MGI:1913760	Rnf181
ENSMUST00000109587.8	-0.317234328	MGI:2157953	Rbm39
ENSMUST00000109636.10	-0.317234328	MGI:1929472	Uqcc1
ENSMUST00000026196.13	-0.317176331	MGI:95791	Got1
ENSMUST00000109701.9	-0.31645141	MGI:97850	Raly
ENSMUST00000001569.14	-0.31643376	MGI:1100500	Flot1
ENSMUST00000085374.6	-0.31588355	MGI:1920211	Slc17a7
ENSMUST00000003461.14	-0.314770552	MGI:1098267	Ogdh
ENSMUST00000031779.16	-0.314770552	MGI:1097158	Calu
ENSMUST00000117831.7	-0.314770552	MGI:109622	Aip
ENSMUST00000165563.7	-0.313866838	MGI:2384909	Micu1
ENSMUST00000020285.9	-0.313172467	MGI:98230	Sar1a
ENSMUST00000172818.7	-0.313108211	MGI:2159342	Pja2
ENSMUST00000196745.4	-0.311499298	MGI:1330861	Dclk1
ENSMUST00000114840.1	-0.310335984	MGI:98747	Thy1
ENSMUST00000174064.8	-0.310181329	MGI:88057	Apoe
ENSMUST00000029082.8	-0.30974767	MGI:1347070	Psma7
ENSMUST00000201948.3	-0.30974767	MGI:1918771	Pds5a
ENSMUST00000103008.11	-0.308858861	MGI:1337026	Sdcbp
ENSMUST00000036734.5	-0.308570085	MGI:1914947	Gadd45gip1
ENSMUST00000038558.8	-0.308570085	MGI:2153049	Klf16
ENSMUST00000029708.7	-0.307322901	MGI:2180167	Naxe
ENSMUST00000051209.10	-0.307322901	MGI:104748	Peg3
ENSMUST00000063814.14	-0.307322901	MGI:1347054	Gnpda1
ENSMUST00000107366.1	-0.307322901	MGI:1917057	Trim32
ENSMUST00000111535.7	-0.307145737	MGI:2442933	Amn1
ENSMUST00000030125.4	-0.305999758	MGI:108047	Bag1
ENSMUST00000031613.10	-0.305999758	MGI:2385237	Aimp2
ENSMUST00000101087.9	-0.305999758	MGI:1333795	Srp72
ENSMUST00000103139.10	-0.305999758	MGI:98742	Thra
ENSMUST00000025804.6	-0.30575196	MGI:1923558	Rab1b
ENSMUST00000030935.9	-0.305496911	MGI:1913719	Prxl2b
ENSMUST00000080926.12	-0.305329547	MGI:95393	Eno1
ENSMUST00000122026.5	-0.304593498	MGI:1934604	Lias
ENSMUST00000029131.10	-0.303096036	MGI:1913385	Ggt7
ENSMUST00000096243.6	-0.303096036	MGI:1919977	B3gat3

ENSMUST00000027810.13	-0.301681356	MGI:95530	Fh1
ENSMUST00000099490.2	-0.301498201	MGI:1276545	Nsd1
ENSMUST00000025851.3	-0.300839749	MGI:1922471	Dpp3
ENSMUST00000020962.11	-0.299789548	MGI:2442310	Ubxn2a
ENSMUST00000221972.1	-0.299789548	MGI:2443413	Rps6kl1
ENSMUST00000059844.12	-0.297958136	MGI:1919279	Cnpy3
ENSMUST00000072287.11	-0.297958136	MGI:1334433	Pi4kb
ENSMUST00000084124.6	-0.297958136	MGI:97553	Pgd
ENSMUST00000032479.10	-0.295990238	MGI:2441908	Pianp
ENSMUST00000074208.5	-0.295990238	MGI:1913956	Ndufaf3
ENSMUST00000179001.7	-0.295990238	MGI:108563	Sirpa
ENSMUST00000001416.7	-0.295114605	MGI:108087	Hars
ENSMUST00000159616.1	-0.295114605	MGI:2389173	Lrrtm1
ENSMUST00000124444.1	-0.29481263	MGI:1929898	Cend1
ENSMUST00000022264.12	-0.293870006	MGI:97814	Ptprg
ENSMUST00000037947.14	-0.293870006	MGI:101769	Mcl1
ENSMUST00000045368.11	-0.293870006	MGI:2677850	Abitram
ENSMUST00000066888.9	-0.293870006	MGI:1923402	Utp18
ENSMUST00000112173.7	-0.293870006	MGI:1924558	Tspan9
ENSMUST00000123853.8	-0.293870006	MGI:2684060	Akap11
ENSMUST00000164465.2	-0.293870006	MGI:106586	Omg
ENSMUST00000227061.1	-0.293870006	MGI:1860443	Rabggta
ENSMUST00000023873.11	-0.291579037	MGI:1351645	Prmt5
ENSMUST00000024594.8	-0.291579037	MGI:1915512	Agpat4
ENSMUST00000061826.2	-0.291579037	MGI:1349405	B3galnt1
ENSMUST00000065887.13	-0.291579037	MGI:1919570	Kifbp
ENSMUST00000094467.5	-0.291579037	MGI:1202295	Entpd6
ENSMUST00000109135.8	-0.291579037	MGI:1353621	Zfp354c
ENSMUST00000180036.7	-0.291579037	MGI:109433	Oaz1
ENSMUST00000027370.12	-0.289234754	MGI:1930773	Pnkd
ENSMUST00000068856.4	-0.289095838	MGI:1913319	Snupn
ENSMUST00000094693.10	-0.289095838	MGI:98287	Srsf5
ENSMUST00000101070.4	-0.287973239	MGI:1915017	Jagn1
ENSMUST00000109420.9	-0.287906474	MGI:2385163	Gdap1l1
ENSMUST00000039784.11	-0.286395151	MGI:2148491	Acaa1a
ENSMUST00000067230.5	-0.286395151	MGI:98366	Sox4
ENSMUST00000082428.4	-0.286395151	MGI:108388	Sephs2
ENSMUST00000225355.1	-0.286395151	MGI:1921445	Elp3
ENSMUST00000047864.10	-0.28467423	MGI:95288	Eef2

ENSMUST00000032992.6	-0.284141111	MGI:1926966	Eif3c
ENSMUST00000112138.7	-0.283589642	MGI:107750	Dync1i2
ENSMUST00000130411.6	-0.283553719	MGI:1915246	Srsf6
ENSMUST00000023043.9	-0.28344709	MGI:103202	Adsl
ENSMUST00000025127.4	-0.28344709	MGI:106271	Mapre2
ENSMUST00000028162.4	-0.28344709	MGI:1917592	Ptges2
ENSMUST00000028377.13	-0.28344709	MGI:98248	Scn2a
ENSMUST00000031766.11	-0.28344709	MGI:1350929	Asns
ENSMUST00000076961.8	-0.28344709	MGI:1201692	Rabac1
ENSMUST00000113233.7	-0.28344709	MGI:1914440	Eif4e2
ENSMUST00000202214.3	-0.28344709	MGI:1196608	Slc4a1ap
ENSMUST00000106197.9	-0.280939675	MGI:2178103	Arhgdia
ENSMUST00000003527.9	-0.280216028	MGI:1202400	Supt5
ENSMUST00000047331.7	-0.280216028	MGI:2182619	Lgi3
ENSMUST00000052248.7	-0.280216028	MGI:1914410	Eef1g
ENSMUST00000110395.10	-0.280216028	MGI:96921	Max
ENSMUST00000171845.7	-0.280216028	MGI:88218	Commd3
ENSMUST00000036497.15	-0.279477481	MGI:97760	Prkar2b
ENSMUST00000031625.14	-0.278861743	MGI:1928896	Arpc1a
ENSMUST00000058159.5	-0.278553276	MGI:1917505	Cnrip1
ENSMUST00000022060.6	-0.27673638	MGI:109283	Pdcd6
ENSMUST00000021001.9	-0.276716903	MGI:105066	Rab10
ENSMUST00000015934.12	-0.276659152	MGI:98443	Surf1
ENSMUST00000034707.14	-0.276659152	MGI:88192	Smarca4
ENSMUST00000106439.1	-0.276659152	MGI:1926269	Mrpl38
ENSMUST00000117200.7	-0.276659152	MGI:1890110	Robo2
ENSMUST00000009727.11	-0.275910664	MGI:1328323	Syngr1
ENSMUST00000039694.12	-0.274784687	MGI:1923455	Stard3nl
ENSMUST00000043843.11	-0.272748921	MGI:109656	Lasp1
ENSMUST00000065970.5	-0.272748921	MGI:1917607	Kcnip1
ENSMUST00000102845.10	-0.272748921	MGI:1925544	Rps27a
ENSMUST00000001480.13	-0.272724549	MGI:1101358	Npepps
ENSMUST00000003964.16	-0.272724549	MGI:101805	Gys1
ENSMUST00000056601.10	-0.272724549	MGI:1915549	Vps53
ENSMUST00000057576.7	-0.272724549	MGI:2685013	Cog7
ENSMUST00000154032.1	-0.272724549	MGI:2446175	Spryd3
ENSMUST00000118209.7	-0.270604689	MGI:1100495	Atp5pb
ENSMUST00000025385.6	-0.27060347	MGI:105089	Hsd17b4
ENSMUST00000033018.14	-0.269857804	MGI:1914670	Far1

ENSMUST00000016452.10	-0.26834866	MGI:102959	Ube2a
ENSMUST00000027377.8	-0.26834866	MGI:96440	Igfbp5
ENSMUST00000035086.12	-0.26834866	MGI:1333753	Pdcd6ip
ENSMUST00000036700.6	-0.26834866	MGI:87934	Adra2a
ENSMUST00000037055.13	-0.26834866	MGI:1922022	Atp13a2
ENSMUST00000043169.13	-0.26834866	MGI:1915747	Arel1
ENSMUST00000048678.6	-0.26834866	MGI:1336155	Lss
ENSMUST00000071402.6	-0.26834866	MGI:2156528	Elovl6
ENSMUST00000077338.11	-0.26834866	MGI:1913599	Dmac2
ENSMUST00000086851.1	-0.26834866	MGI:1859852	Hes6
ENSMUST00000111230.7	-0.26834866	MGI:1312985	Tagln2
ENSMUST00000091609.10	-0.267642018	MGI:1921575	Cltb
ENSMUST00000056758.8	-0.267133196	MGI:107164	Ppp3ca
ENSMUST00000045993.14	-0.266734591	MGI:1349414	Cops4
ENSMUST00000019679.11	-0.265929784	MGI:1924063	Armc6
ENSMUST00000052919.7	-0.265929784	MGI:1913862	Ormdl3
ENSMUST00000057373.13	-0.265929784	MGI:99425	Rab11b
ENSMUST00000237431.1	-0.265929784	MGI:2448270	Rpl17
ENSMUST00000106257.9	-0.264373373	MGI:88262	Cap1
ENSMUST00000018572.10	-0.26345281	MGI:104729	Akap1
ENSMUST00000021530.7	-0.26345281	MGI:106918	Hif1a
ENSMUST00000022235.5	-0.26345281	MGI:96273	Htr1a
ENSMUST00000025166.13	-0.26345281	MGI:88355	Cdh2
ENSMUST00000027897.7	-0.26345281	MGI:1915889	Smyd2
ENSMUST00000085745.12	-0.26345281	MGI:1921932	Wdr35
ENSMUST00000145167.7	-0.26345281	MGI:107898	Selenoi
ENSMUST00000024978.6	-0.263383839	MGI:1930182	Nme3
ENSMUST00000110731.3	-0.263383839	MGI:1914163	Kdelr2
ENSMUST00000028214.14	-0.263360529	MGI:2385131	Sh3glb2
ENSMUST00000186213.6	-0.263337057	MGI:1095410	Tuba4a
ENSMUST00000084949.2	-0.261569789	MGI:1915720	Impad1
ENSMUST00000000727.3	-0.260680327	MGI:105938	Rab5b
ENSMUST00000025853.15	-0.260680327	MGI:1913806	Drap1
ENSMUST00000033809.3	-0.260680327	MGI:97775	Prps1
ENSMUST00000034131.9	-0.25938679	MGI:1890467	Vps35
ENSMUST00000021130.6	-0.257938437	MGI:1916785	Ten1
ENSMUST00000031736.15	-0.257938437	MGI:2443267	Agfg2
ENSMUST00000036805.6	-0.257938437	MGI:1925920	Plekhj1
ENSMUST00000044278.5	-0.257938437	MGI:1349471	St6galnac5

ENSMUST00000064759.6	-0.257938437	MGI:2443884	Strip1
ENSMUST00000064941.6	-0.257938437	MGI:2444210	Nr1d1
ENSMUST00000078528.6	-0.257938437	MGI:1194505	C1qbp
ENSMUST00000099858.3	-0.257938437	MGI:1270863	Prep
ENSMUST00000102528.10	-0.257938437	MGI:104872	Ppp1cc
ENSMUST00000151309.7	-0.257938437	MGI:2441837	Adgrb3
ENSMUST00000174462.7	-0.257938437	MGI:1924769	Zfp266
ENSMUST00000200154.4	-0.257938437	MGI:2139714	Adgrl2
ENSMUST00000028102.13	-0.257804147	MGI:1098269	Kif5c
ENSMUST00000228198.1	-0.257735883	MGI:107795	Hnrnpc
ENSMUST00000106251.9	-0.255655659	MGI:1353633	Fus
ENSMUST00000001975.5	-0.254738206	MGI:1914080	Nacc1
ENSMUST00000024702.4	-0.254738206	MGI:1923748	Paqr4
ENSMUST00000079970.5	-0.254738206	MGI:1913495	Hspbp1
ENSMUST00000098495.9	-0.254738206	MGI:109132	Snap91
ENSMUST00000119694.2	-0.25354346	MGI:1861434	Ctsf
ENSMUST00000080356.9	-0.253221919	MGI:1855690	Snrpa
ENSMUST00000067036.11	-0.252962457	MGI:88208	Bsg
ENSMUST00000066027.13	-0.252677927	MGI:1202877	Dgcr6
ENSMUST00000018653.7	-0.251680374	MGI:1920389	Cenpv
ENSMUST00000024708.5	-0.251680374	MGI:2151075	Tnfrsf21
ENSMUST00000026558.6	-0.251680374	MGI:2141866	Ric8a
ENSMUST00000028769.13	-0.251680374	MGI:97808	Ptpra
ENSMUST00000029938.9	-0.251680374	MGI:2385191	Gtf2b
ENSMUST00000030684.7	-0.251680374	MGI:2385207	Gnl2
ENSMUST00000031254.8	-0.251680374	MGI:2179430	Klhl8
ENSMUST00000033494.15	-0.251680374	MGI:1859615	Otud5
ENSMUST00000039476.14	-0.251680374	MGI:2441869	Arhgef11
ENSMUST00000039987.3	-0.251680374	MGI:2181659	Tox
ENSMUST00000044475.4	-0.251680374	MGI:1339639	Ogt
ENSMUST00000070172.5	-0.251680374	MGI:2444704	Snx32
ENSMUST00000072329.14	-0.251680374	MGI:2137586	Dtnbp1
ENSMUST00000080536.7	-0.251680374	MGI:1195458	Abce1
ENSMUST00000115511.8	-0.251680374	MGI:1923539	Phf14
ENSMUST00000174499.7	-0.251680374	MGI:1923020	Brsk2
ENSMUST00000185176.7	-0.251680374	MGI:2684762	Lzts1
ENSMUST00000194542.5	-0.251680374	MGI:1858732	Keap1
ENSMUST00000043814.4	-0.251463076	MGI:2143585	Fig4
ENSMUST00000053740.14	-0.251463076	MGI:1340045	Zfp207

ENSMUST00000075693.11	-0.251463076	MGI:1915532	Yipf1
ENSMUST00000163336.7	-0.251463076	MGI:1350932	Ncoa4
ENSMUST00000174252.7	-0.251463076	MGI:1289172	Scyl2
ENSMUST00000021610.6	-0.249067983	MGI:88394	Chga
ENSMUST00000046951.9	-0.249067983	MGI:1915333	Pak1ip1
ENSMUST00000088419.12	-0.248520521	MGI:2145597	Mbnl2
ENSMUST00000029610.8	-0.247956581	MGI:96009	Hadh
ENSMUST00000102695.3	-0.247956581	MGI:1917127	Nt5m
ENSMUST00000109249.8	-0.247956581	MGI:1919293	Sulf2
ENSMUST00000106677.7	-0.245340097	MGI:104878	Prkar1a
ENSMUST00000032931.8	-0.244517226	MGI:2448475	Fchsd2
ENSMUST00000035090.13	-0.244517226	MGI:1919429	Fbxl2
ENSMUST00000047485.14	-0.244517226	MGI:2685286	Rundc3b
ENSMUST00000077367.10	-0.244517226	MGI:95665	Gba
ENSMUST00000097811.9	-0.244517226	MGI:2152971	Rims1
ENSMUST00000103020.7	-0.244517226	MGI:1197518	Aatk
ENSMUST00000112810.7	-0.244517226	MGI:2444269	Gtdc1
ENSMUST00000112936.3	-0.244517226	MGI:2442789	Rc3h2
ENSMUST00000168787.7	-0.244517226	MGI:1916625	Btbd9
ENSMUST00000170580.2	-0.244517226	MGI:2687399	Kcnf1
ENSMUST00000180360.7	-0.244517226	MGI:2667252	Ehbp1
ENSMUST00000199834.4	-0.244517226	MGI:1921633	Ubap2l
ENSMUST00000106153.8	-0.244193297	MGI:2139150	Ssx2ip
ENSMUST00000188211.7	-0.244193297	MGI:106330	Larp4b
ENSMUST00000153700.8	-0.243972747	MGI:109492	Oaz2
ENSMUST00000007708.13	-0.242582923	MGI:1926334	Ppp2r1a
ENSMUST00000027863.12	-0.242078122	MGI:88108	Atp1b1
ENSMUST00000001845.12	-0.24205621	MGI:88266	Capns1
ENSMUST00000163242.2	-0.241903298	MGI:1859293	Atxn10
ENSMUST00000021412.8	-0.241653682	MGI:1347006	Psma6
ENSMUST00000057257.9	-0.241403341	MGI:1915057	Jkamp
ENSMUST00000082223.12	-0.241403341	MGI:102854	Rpl5
ENSMUST00000020484.7	-0.240143928	MGI:1354175	Txnrd1
ENSMUST00000044721.12	-0.239248956	MGI:103286	Atp6v0a1
ENSMUST00000110876.8	-0.238585002	MGI:103263	Mcf2l
ENSMUST00000005643.13	-0.236237225	MGI:1342295	Celf1
ENSMUST00000016144.11	-0.236237225	MGI:108412	Plpp1
ENSMUST00000021226.13	-0.236237225	MGI:1914934	Luc7l3
ENSMUST00000022225.11	-0.236237225	MGI:1933161	Trim23

ENSMUST00000025092.4	-0.236237225	MGI:1915277	Tmem178
ENSMUST00000027358.10	-0.236237225	MGI:1914071	Bcs1l
ENSMUST00000029084.8	-0.236237225	MGI:97386	Ntsr1
ENSMUST00000031538.8	-0.236237225	MGI:1919607	2210016L21Rik
ENSMUST00000032409.14	-0.236237225	MGI:1098535	Camk1
ENSMUST00000043680.8	-0.236237225	MGI:101834	Tubg1
ENSMUST00000046515.14	-0.236237225	MGI:2443191	Nceh1
ENSMUST00000050067.9	-0.236237225	MGI:1923858	Hectd3
ENSMUST00000058472.12	-0.236237225	MGI:1915916	Svop
ENSMUST00000068058.13	-0.236237225	MGI:1916977	Usp46
ENSMUST00000080919.11	-0.236237225	MGI:2442637	Thrap3
ENSMUST00000089236.10	-0.236237225	MGI:2444129	Pnma2
ENSMUST00000103066.9	-0.236237225	MGI:1921439	Phactr3
ENSMUST00000106157.7	-0.236237225	MGI:106441	Zranb1
ENSMUST00000204545.2	-0.236237225	MGI:1927340	Mpp6
ENSMUST00000236978.1	-0.236237225	MGI:1931787	Scyl1
ENSMUST00000052209.8	-0.235774545	MGI:99851	Cbfb
ENSMUST00000089027.2	-0.235774545	MGI:2139220	Tm9sf4
ENSMUST00000169687.7	-0.235617495	MGI:99778	Gpd2
ENSMUST00000021147.13	-0.235490817	MGI:1859270	Exoc7
ENSMUST00000003121.8	-0.23248865	MGI:96960	Rab8a
ENSMUST00000189373.7	-0.23248865	MGI:105371	Spock1
ENSMUST00000059667.8	-0.231353459	MGI:2157521	Hpcal4
ENSMUST00000009234.15	-0.231045631	MGI:1096368	Ap1b1
ENSMUST00000029178.6	-0.231045631	MGI:1913892	Ctnnbl1
ENSMUST00000030905.8	-0.231045631	MGI:1916241	Ssu72
ENSMUST00000087557.11	-0.231045631	MGI:1926264	Tspan6
ENSMUST00000019323.10	-0.230577089	MGI:97050	Mdh2
ENSMUST00000072298.12	-0.229927495	MGI:1352499	Ndrg3
ENSMUST00000024099.10	-0.229184931	MGI:87876	Ache
ENSMUST00000044106.5	-0.229184931	MGI:1351511	Psmd7
ENSMUST00000111220.7	-0.228886264	MGI:2137858	Cadm3
ENSMUST00000002733.6	-0.226556909	MGI:1923848	Gtf2f1
ENSMUST00000004379.7	-0.226556909	MGI:1315195	Emg1
ENSMUST00000008088.8	-0.226556909	MGI:1920282	Ttc9b
ENSMUST00000015901.10	-0.226556909	MGI:1914668	Ppil4
ENSMUST00000026050.7	-0.226556909	MGI:1342273	Gsto1
ENSMUST00000027780.5	-0.226556909	MGI:2181074	Acbd3
ENSMUST00000030702.13	-0.226556909	MGI:2140494	Ppp1r8

ENSMUST00000031262.8	-0.226556909	MGI:1919133	Coq2
ENSMUST00000034912.5	-0.226556909	MGI:99694	Rasgrf1
ENSMUST00000037850.6	-0.226556909	MGI:1915054	Snx2
ENSMUST00000043359.8	-0.226556909	MGI:1935129	Smarca5
ENSMUST00000048578.2	-0.226556909	MGI:1914077	Ttc1
ENSMUST00000055128.11	-0.226556909	MGI:2683537	Tapt1
ENSMUST00000056665.3	-0.226556909	MGI:2388648	Klhl11
ENSMUST00000058884.9	-0.226556909	MGI:1921358	Parn
ENSMUST00000065601.12	-0.226556909	MGI:1338871	Btrc
ENSMUST00000082414.1	-0.226556909	MGI:102498	mt-Nd4
ENSMUST00000084519.6	-0.226556909	MGI:1920230	Wdr11
ENSMUST00000090024.10	-0.226556909	MGI:101859	Ccser2
ENSMUST00000094452.3	-0.226556909	MGI:2445030	Wscd2
ENSMUST00000098350.9	-0.226556909	MGI:2135958	Scap
ENSMUST00000099151.5	-0.226556909	MGI:98364	Sox2
ENSMUST00000099689.4	-0.226556909	MGI:3652053	Gm13889
ENSMUST00000102866.9	-0.226556909	MGI:1860267	Set
ENSMUST00000105267.7	-0.226556909	MGI:1919318	Cnot2
ENSMUST00000114023.2	-0.226556909	MGI:1913859	Cryzl1
ENSMUST00000156390.7	-0.226556909	MGI:106594	Gk
ENSMUST00000169314.8	-0.226556909	MGI:2442264	Idi1
ENSMUST00000179865.7	-0.226556909	MGI:88261	Canx
ENSMUST00000186380.6	-0.226556909	MGI:108177	Dhx9
ENSMUST00000206129.2	-0.226556909	MGI:5625257	Gm42372
ENSMUST00000003961.15	-0.225910875	MGI:1924037	Ppfia3
ENSMUST00000036570.4	-0.225910875	MGI:1920243	Appl1
ENSMUST00000049064.3	-0.225910875	MGI:1921262	Rap2b
ENSMUST00000079987.12	-0.225910875	MGI:1923380	Las1l
ENSMUST00000096246.4	-0.225910875	MGI:1097667	Ganab
ENSMUST00000102800.7	-0.225910875	MGI:1913941	Gapvd1
ENSMUST00000182709.7	-0.225910875	MGI:1201691	Rnf4
ENSMUST00000033609.8	-0.225691249	MGI:1343054	Cstf2
ENSMUST00000112699.8	-0.225580615	MGI:1933391	Maged2
ENSMUST00000087435.6	-0.220315656	MGI:1095407	Bmpr2
ENSMUST00000113710.7	-0.220315656	MGI:1100521	Slc7a3
ENSMUST00000023599.12	-0.219239365	MGI:106906	Eif4a2
ENSMUST00000073339.6	-0.219012419	MGI:1858305	Pgrmc1
ENSMUST00000046404.7	-0.21865299	MGI:1913872	Ubr7
ENSMUST00000001757.8	-0.218064408	MGI:1913393	Eef1e1

ENSMUST00000070478.3	-0.217848955	MGI:1349163	Sdc3
ENSMUST00000025110.4	-0.217649506	MGI:101759	Syt4
ENSMUST00000034049.4	-0.217001731	MGI:1353495	Slc25a4
ENSMUST00000005015.9	-0.215087946	MGI:2137738	Prcc
ENSMUST00000006914.10	-0.215087946	MGI:1342057	B4galnt1
ENSMUST00000022169.9	-0.215087946	MGI:96074	Hexb
ENSMUST00000029053.7	-0.215087946	MGI:97805	Ptpn1
ENSMUST00000029784.9	-0.215087946	MGI:1926034	Celf3
ENSMUST00000030145.8	-0.215087946	MGI:1916220	Dcaf12
ENSMUST00000030784.13	-0.215087946	MGI:1336153	Prkag2
ENSMUST00000032326.10	-0.215087946	MGI:1915005	Ddx47
ENSMUST00000033473.11	-0.215087946	MGI:109178	Fgf13
ENSMUST00000041804.7	-0.215087946	MGI:3036247	Lmtk2
ENSMUST00000042485.10	-0.215087946	MGI:2143322	AW551984
ENSMUST00000044200.10	-0.215087946	MGI:107891	Nop2
ENSMUST00000046399.6	-0.215087946	MGI:1919131	Apmap
ENSMUST00000048068.14	-0.215087946	MGI:1913662	Arrdc4
ENSMUST00000050312.2	-0.215087946	MGI:1309464	Mapk8ip1
ENSMUST00000060389.9	-0.215087946	MGI:1914860	Rspry1
ENSMUST00000073192.13	-0.215087946	MGI:1914003	Erlec1
ENSMUST00000077741.11	-0.215087946	MGI:2443511	Slc9a6
ENSMUST00000080856.13	-0.215087946	MGI:2442377	Ipo11
ENSMUST00000088666.3	-0.215087946	MGI:1924232	Vxn
ENSMUST00000090791.7	-0.215087946	MGI:1922387	Rprd2
ENSMUST00000090858.9	-0.215087946	MGI:2445173	Ppig
ENSMUST00000094569.10	-0.215087946	MGI:104753	Nfasc
ENSMUST00000096240.2	-0.215087946	MGI:1346340	Mta2
ENSMUST00000102810.9	-0.215087946	MGI:2139309	Garnl3
ENSMUST00000103143.9	-0.215087946	MGI:1919444	Fbxl20
ENSMUST00000103179.9	-0.215087946	MGI:2180699	Mtmr4
ENSMUST00000105845.8	-0.215087946	MGI:99611	Ephb2
ENSMUST00000107558.8	-0.215087946	MGI:99533	Mef2d
ENSMUST00000117648.8	-0.215087946	MGI:1276539	Ttc3
ENSMUST00000126390.7	-0.215087946	MGI:1196373	Reps1
ENSMUST00000154111.7	-0.215087946	MGI:1330239	Dpm1
ENSMUST00000161592.7	-0.215087946	MGI:1913516	Eapp
ENSMUST00000184973.7	-0.215087946	MGI:1914714	Entpd4
ENSMUST00000197188.4	-0.215087946	MGI:1261856	Ankle2
ENSMUST00000001155.10	-0.214752778	MGI:88065	Araf

ENSMUST00000008893.8	-0.214195076	MGI:1345963	Coro1b
ENSMUST00000040576.9	-0.214195076	MGI:2443349	Parm1
ENSMUST00000057844.9	-0.214195076	MGI:1925771	B230219D22Rik
ENSMUST00000098312.3	-0.214195076	MGI:2142527	Exoc8
ENSMUST00000112691.8	-0.214195076	MGI:2448557	Gnl3l
ENSMUST00000174259.7	-0.214195076	MGI:1096367	Psme1
ENSMUST00000189936.6	-0.214195076	MGI:1914848	Uchl5
ENSMUST00000064054.13	-0.214008035	MGI:99667	Syt1
ENSMUST00000019065.9	-0.213890975	MGI:1922523	Pelp1
ENSMUST00000112308.8	-0.213890975	MGI:1919666	Lrpprc
ENSMUST00000022256.4	-0.213737681	MGI:1913663	Psmd6
ENSMUST00000072299.6	-0.213397471	MGI:1349453	Vsnl1
ENSMUST00000030636.10	-0.212150666	MGI:96739	Stmn1
ENSMUST00000023095.13	-0.209713051	MGI:1345148	Septin3
ENSMUST00000025000.3	-0.209446317	MGI:2445190	St6gal2
ENSMUST00000034947.6	-0.209446317	MGI:97750	Ppib
ENSMUST00000094154.5	-0.209446317	MGI:88283	Serpinh1
ENSMUST00000165559.2	-0.209103733	MGI:2685518	Ctif
ENSMUST00000030623.7	-0.20824274	MGI:1918764	Sfpq
ENSMUST00000055808.5	-0.208147603	MGI:108109	Ywhag
ENSMUST00000029013.9	-0.207718691	MGI:1913929	Rae1
ENSMUST00000074523.12	-0.20755035	MGI:1931224	Stmn4
ENSMUST00000003876.9	-0.207471436	MGI:1925906	Brd8
ENSMUST00000023100.7	-0.207471436	MGI:107585	Srebf2
ENSMUST00000026520.13	-0.207471436	MGI:2384993	Paip1
ENSMUST00000045905.13	-0.207471436	MGI:2385001	Fermt2
ENSMUST00000049095.5	-0.207471436	MGI:109609	Faah
ENSMUST00000053686.8	-0.207471436	MGI:1931744	Uck2
ENSMUST00000070039.13	-0.207471436	MGI:2675856	Fez2
ENSMUST00000086519.11	-0.207471436	MGI:1927636	Rplp0
ENSMUST00000095014.7	-0.207471436	MGI:2447427	Tgfbrap1
ENSMUST00000069107.13	-0.20723791	MGI:1346858	Mapk1
ENSMUST00000020286.6	-0.205667081	MGI:97831	Ppa1
ENSMUST00000027726.13	-0.204703082	MGI:1919267	Cyb5r1
ENSMUST00000160964.7	-0.204321221	MGI:2384590	Ndrg4
ENSMUST00000033715.4	-0.203555718	MGI:1099438	Nsdhl
ENSMUST00000101534.4	-0.203089305	MGI:97804	Ptn
ENSMUST00000008878.9	-0.201283652	MGI:1927596	Gprc5b
ENSMUST00000015138.12	-0.201283652	MGI:95317	Eln

ENSMUST00000017530.3	-0.201283652	MGI:1202880	Traf4
ENSMUST00000017692.14	-0.201283652	MGI:1261758	Suz12
ENSMUST00000018569.13	-0.201283652	MGI:1914737	Dhx40
ENSMUST00000020947.6	-0.201283652	MGI:1920402	Rdh14
ENSMUST00000022099.14	-0.201283652	MGI:2384812	Lpcat1
ENSMUST00000022322.16	-0.201283652	MGI:95753	Glud1
ENSMUST00000022377.10	-0.201283652	MGI:1917811	Txndc16
ENSMUST00000025253.11	-0.201283652	MGI:1915467	Prrc2a
ENSMUST00000027252.7	-0.201283652	MGI:2441772	Eif5b
ENSMUST00000028635.5	-0.201283652	MGI:1330276	Cops2
ENSMUST00000029769.13	-0.201283652	MGI:104995	Gclm
ENSMUST00000030365.5	-0.201283652	MGI:1926268	Mrpl37
ENSMUST00000031090.7	-0.201283652	MGI:1916941	Sel1l3
ENSMUST00000031492.14	-0.201283652	MGI:1924657	Rab35
ENSMUST00000031621.10	-0.201283652	MGI:2141070	Ccz1
ENSMUST00000032080.8	-0.201283652	MGI:1920577	Pradc1
ENSMUST00000035889.14	-0.201283652	MGI:104785	Myo6
ENSMUST00000036288.10	-0.201283652	MGI:2448514	R3hdm1
ENSMUST00000038252.3	-0.201283652	MGI:1349461	B3galt2
ENSMUST00000043624.8	-0.201283652	MGI:3029632	Med13
ENSMUST00000046999.11	-0.201283652	MGI:1916008	Abhd11
ENSMUST00000048685.12	-0.201283652	MGI:1915894	Abhd14a
ENSMUST00000059155.10	-0.201283652	MGI:1916289	Insig1
ENSMUST00000059642.16	-0.201283652	MGI:1888669	Psmd8
ENSMUST00000064740.8	-0.201283652	MGI:1927234	Suclg1
ENSMUST00000069443.13	-0.201283652	MGI:2145578	Slain1
ENSMUST00000075510.11	-0.201283652	MGI:2146808	Safb2
ENSMUST00000080721.5	-0.201283652	MGI:1926173	Chsy3
ENSMUST00000081728.6	-0.201283652	MGI:1195966	Ctnnd2
ENSMUST00000089925.9	-0.201283652	MGI:105060	Dgkg
ENSMUST00000090559.11	-0.201283652	MGI:1270859	Cry2
ENSMUST00000092629.3	-0.201283652	MGI:1914662	Soga3
ENSMUST00000099972.4	-0.201283652	MGI:96603	Itga4
ENSMUST00000108243.7	-0.201283652	MGI:1206581	Pik3ca
ENSMUST00000109097.8	-0.201283652	MGI:1924621	Sec24a
ENSMUST00000109543.8	-0.201283652	MGI:102889	Vcan
ENSMUST00000109579.8	-0.201283652	MGI:2384495	Mrtfa
ENSMUST00000109790.1	-0.201283652	MGI:2684063	Asxl1
ENSMUST00000112024.9	-0.201283652	MGI:1915674	Chn1

ENSMUST00000112471.8	-0.201283652	MGI:1917474	Map7d2
ENSMUST00000118788.7	-0.201283652	MGI:95745	Hagh
ENSMUST00000120595.7	-0.201283652	MGI:1919455	Eml2
ENSMUST00000125249.2	-0.201283652	MGI:3609243	Isoc2a
ENSMUST00000160456.7	-0.201283652	MGI:1917979	Nos1ap
ENSMUST00000162349.7	-0.201283652	MGI:108405	Apbb2
ENSMUST00000162372.7	-0.201283652	MGI:1923690	Slc30a9
ENSMUST00000164884.8	-0.201283652	MGI:1915773	Ciao2b
ENSMUST00000170202.8	-0.201283652	MGI:95871	Guk1
ENSMUST00000172272.7	-0.201283652	MGI:1920895	Ddx46
ENSMUST00000179543.7	-0.201283652	MGI:1931051	Noc2l
ENSMUST00000180376.7	-0.201283652	MGI:2447768	Fam193a
ENSMUST00000193777.5	-0.201283652	MGI:1298367	Pcdha6
ENSMUST00000193869.1	-0.201283652	MGI:1935214	Pcdhga2
ENSMUST00000209346.1	-0.201283652	MGI:2443755	Rasgef1b
ENSMUST00000215370.1	-0.201283652	MGI:2387648	Fam214a
ENSMUST00000236586.1	-0.201283652	MGI:95513	Fech
ENSMUST00000027247.10	-0.200050902	MGI:1916083	Pdcl3
ENSMUST00000036578.6	-0.200050902	MGI:2139371	Bahd1
ENSMUST00000066379.10	-0.200050902	MGI:1913325	Chchd3
ENSMUST00000076313.12	-0.200050902	MGI:1860512	Prpf40a
ENSMUST00000079659.11	-0.200050902	MGI:1915208	U2surp
ENSMUST00000100507.7	-0.200050902	MGI:106478	Eif3b
ENSMUST00000121623.7	-0.200050902	MGI:1922870	Kxd1
ENSMUST00000141158.7	-0.200050902	MGI:1929461	Adgrl1
ENSMUST00000001479.4	-0.199630109	MGI:107532	Kpnb1
ENSMUST00000034470.10	-0.199630109	MGI:1917656	Vps26b
ENSMUST00000044058.10	-0.199630109	MGI:1915600	Mul1
ENSMUST00000103061.2	-0.199630109	MGI:104837	Amz2
ENSMUST00000065118.6	-0.199417811	MGI:1924230	Ube2ql1
ENSMUST00000092892.9	-0.199417811	MGI:2144501	Ankrd13b
ENSMUST00000117160.1	-0.199417811	MGI:99551	Cdh13
ENSMUST00000216925.1	-0.199417811	MGI:1916709	Ubl7
ENSMUST00000025645.13	-0.199289816	MGI:2147810	Tmem132a
ENSMUST00000034230.6	-0.198869412	MGI:1097153	Cx3cl1
ENSMUST00000092096.13	-0.197022373	MGI:1928898	Usp14
ENSMUST00000236011.1	-0.197022373	MGI:1915229	Atad1
ENSMUST00000028059.8	-0.196049982	MGI:103040	Rsu1
ENSMUST00000183108.7	-0.195357223	MGI:2180003	Xpnpep1

ENSMUST00000022904.7	-0.195008774	MGI:1913585	Atp6v1c1
ENSMUST00000090971.10	-0.194457245	MGI:1096385	Bcan
ENSMUST00000046514.12	-0.194191742	MGI:97838	Eprs
ENSMUST00000165017.1	-0.194191742	MGI:1918019	Nolc1
ENSMUST00000032888.9	-0.19257367	MGI:1858943	Arl6ip1
ENSMUST00000040603.13	-0.191819089	MGI:1924809	Agl
ENSMUST00000041826.13	-0.191819089	MGI:1346341	Rnf13
ENSMUST00000047615.14	-0.191819089	MGI:1859183	Smarcal1
ENSMUST00000098817.3	-0.191819089	MGI:1261835	Vps37a
ENSMUST00000174302.7	-0.191819089	MGI:98512	Ubtf
ENSMUST00000177595.8	-0.191819089	MGI:1858901	Fut8
ENSMUST00000070395.8	-0.190820501	MGI:1916934	Aarsd1
ENSMUST00000106205.8	-0.190820501	MGI:104681	Hgs
ENSMUST00000162409.7	-0.18913554	MGI:1925642	Fnbp1l
ENSMUST00000005066.8	-0.188346986	MGI:1346866	Map2k1
ENSMUST00000047131.15	-0.188346986	MGI:1923001	Ipo4
ENSMUST00000019354.10	-0.187493654	MGI:894326	Atp6v1e1
ENSMUST00000027127.13	-0.186984308	MGI:1932339	Sf3b1
ENSMUST00000036153.11	-0.186858667	MGI:1915363	Cops8
ENSMUST00000056427.9	-0.186591408	MGI:107861	Tubb2a
ENSMUST00000009220.4	-0.18654463	MGI:1914428	Zmat5
ENSMUST00000000291.8	-0.184349006	MGI:109150	Mnt
ENSMUST00000002889.4	-0.184349006	MGI:1342286	Flii
ENSMUST00000006286.8	-0.184349006	MGI:1194899	Inpp5k
ENSMUST00000018143.15	-0.184349006	MGI:2385884	Ddx27
ENSMUST00000019660.10	-0.184349006	MGI:1921820	Zkscan1
ENSMUST00000021166.5	-0.184349006	MGI:2149481	Cygb
ENSMUST00000021271.13	-0.184349006	MGI:1098283	Per1
ENSMUST00000022245.9	-0.184349006	MGI:1926237	Mrps30
ENSMUST00000022458.10	-0.184349006	MGI:1206586	Bap1
ENSMUST00000022720.14	-0.184349006	MGI:1354702	Fbxl3
ENSMUST00000022828.8	-0.184349006	MGI:1934682	Emc9
ENSMUST00000022976.5	-0.184349006	MGI:2146110	Washc5
ENSMUST00000023334.14	-0.184349006	MGI:1930171	Nectin3
ENSMUST00000025089.8	-0.184349006	MGI:2154405	Map4k3
ENSMUST00000025241.6	-0.184349006	MGI:95414	Ercc3
ENSMUST00000026658.12	-0.184349006	MGI:2443265	Tnrc6c
ENSMUST00000026832.13	-0.184349006	MGI:1919356	Jmjd8
ENSMUST00000027512.12	-0.184349006	MGI:1924290	Atg16l1

ENSMUST00000028499.10	-0.184349006	MGI:96608	Itgav
ENSMUST00000028608.12	-0.184349006	MGI:2138939	Nat10
ENSMUST00000028668.7	-0.184349006	MGI:1925905	Eif3j1
ENSMUST00000028844.10	-0.184349006	MGI:1913802	Sppl2a
ENSMUST00000029386.13	-0.184349006	MGI:106100	Etfdh
ENSMUST00000029909.2	-0.184349006	MGI:101813	Coq3
ENSMUST00000031414.14	-0.184349006	MGI:1919649	Brap
ENSMUST00000037915.8	-0.184349006	MGI:1921276	Msl1
ENSMUST00000038185.9	-0.184349006	MGI:1922485	Exd2
ENSMUST00000039013.14	-0.184349006	MGI:1351617	Abca3
ENSMUST00000039733.9	-0.184349006	MGI:2146553	Osbpl11
ENSMUST00000039926.9	-0.184349006	MGI:106626	Dusp8
ENSMUST00000040500.8	-0.184349006	MGI:2442474	Dgkb
ENSMUST00000041231.13	-0.184349006	MGI:2143994	Psme4
ENSMUST00000041587.7	-0.184349006	MGI:2146207	Gga1
ENSMUST00000041638.7	-0.184349006	MGI:2138383	Gtf3c3
ENSMUST00000041965.4	-0.184349006	MGI:2136459	Cdc42bpb
ENSMUST00000042055.9	-0.184349006	MGI:1346006	Ptpa
ENSMUST00000042196.3	-0.184349006	MGI:2179729	Vwa1
ENSMUST00000043138.12	-0.184349006	MGI:2141867	Inpp5f
ENSMUST00000044507.11	-0.184349006	MGI:2158663	Inpp5j
ENSMUST00000047664.15	-0.184349006	MGI:2442507	Arhgef4
ENSMUST00000048976.7	-0.184349006	MGI:1926562	Gucy1a1
ENSMUST00000050772.9	-0.184349006	MGI:1926225	Slc22a17
ENSMUST00000051169.12	-0.184349006	MGI:1919831	Edrf1
ENSMUST00000051341.5	-0.184349006	MGI:2145901	Mfsd5
ENSMUST00000051377.14	-0.184349006	MGI:2443952	Dpy19l3
ENSMUST00000051950.13	-0.184349006	MGI:1277223	Atxn2
ENSMUST00000055792.7	-0.184349006	MGI:3583960	D030056L22Rik
ENSMUST00000058716.13	-0.184349006	MGI:1924303	Sun1
ENSMUST00000060435.6	-0.184349006	MGI:2151208	Selenon
ENSMUST00000062117.13	-0.184349006	MGI:97855	Rap2a
ENSMUST00000063857.10	-0.184349006	MGI:95760	Slc6a9
ENSMUST00000069453.8	-0.184349006	MGI:2679683	Paqr3
ENSMUST00000070064.10	-0.184349006	MGI:97566	Pgm3
ENSMUST00000072744.14	-0.184349006	MGI:2144805	Vipas39
ENSMUST00000074733.10	-0.184349006	MGI:1277214	Septin11
ENSMUST00000079362.12	-0.184349006	MGI:88039	Apc
ENSMUST00000079597.6	-0.184349006	MGI:1922802	Paqr9

ENSMUST00000084615.9	-0.184349006	MGI:1859165	Cacng3
ENSMUST00000085383.10	-0.184349006	MGI:2141980	Scaf1
ENSMUST00000085385.6	-0.184349006	MGI:1931050	Ralgapa1
ENSMUST00000087104.10	-0.184349006	MGI:1278336	Cdkl5
ENSMUST00000087659.10	-0.184349006	MGI:2685817	Hecw2
ENSMUST00000088615.10	-0.184349006	MGI:2442988	Arfgef1
ENSMUST00000089311.10	-0.184349006	MGI:2443011	Sun2
ENSMUST00000092618.8	-0.184349006	MGI:107789	Aup1
ENSMUST00000092834.11	-0.184349006	MGI:1354742	Synrg
ENSMUST00000092857.12	-0.184349006	MGI:1926078	Rhot1
ENSMUST00000093852.4	-0.184349006	MGI:103222	Zbtb16
ENSMUST00000096368.3	-0.184349006	MGI:1316727	Gspt2
ENSMUST00000098363.9	-0.184349006	MGI:2152211	Necab2
ENSMUST00000099513.7	-0.184349006	MGI:1298210	Hcn2
ENSMUST00000101122.2	-0.184349006	MGI:1202299	Gpr27
ENSMUST00000101509.8	-0.184349006	MGI:96417	Ids
ENSMUST00000101532.9	-0.184349006	MGI:2443430	Dgki
ENSMUST00000102628.10	-0.184349006	MGI:2387201	Yrdc
ENSMUST00000102776.4	-0.184349006	MGI:1891717	Rnf130
ENSMUST00000102905.7	-0.184349006	MGI:5141924	Pakap
ENSMUST00000102905.7	-0.184349006	MGI:5141924	Pakap
ENSMUST00000102905.7	-0.184349006	MGI:5141924	Pakap
ENSMUST00000102905.7	-0.184349006	MGI:5141924	Pakap
ENSMUST00000102905.7	-0.184349006	MGI:5141924	Pakap
ENSMUST00000102905.7	-0.184349006	MGI:5141924	Pakap
ENSMUST00000102905.7	-0.184349006	MGI:5141924	Pakap
ENSMUST00000102905.7	-0.184349006	MGI:5141924	Pakap
ENSMUST00000102905.7	-0.184349006	MGI:5141924	Pakap
ENSMUST00000105032.3	-0.184349006	MGI:3651622	Fam43b
ENSMUST00000105283.8	-0.184349006	MGI:96974	Kitl
ENSMUST00000107695.8	-0.184349006	MGI:1933163	Trim2
ENSMUST00000107734.9	-0.184349006	MGI:2182799	Kat7
ENSMUST00000108777.9	-0.184349006	MGI:2144404	Jmjd4
ENSMUST00000111238.7	-0.184349006	MGI:1921819	Ttc17
ENSMUST00000111557.7	-0.184349006	MGI:2444273	Dennd5b
ENSMUST00000111879.4	-0.184349006	MGI:3039580	Dclk3
ENSMUST00000113641.3	-0.184349006	MGI:1932051	Kat5
ENSMUST00000114656.7	-0.184349006	MGI:95564	Fmr1
ENSMUST00000115077.7	-0.184349006	MGI:1351667	Abcb8

ENSMUST00000117394.1	-0.184349006	MGI:1915919	Taok2
ENSMUST00000118317.7	-0.184349006	MGI:1314873	Hipk1
ENSMUST00000120705.2	-0.184349006	MGI:1915699	Tbc1d10b
ENSMUST00000122041.7	-0.184349006	MGI:99555	Pde4d
ENSMUST00000123285.1	-0.184349006	MGI:2443135	Nyap2
ENSMUST00000124973.8	-0.184349006	MGI:1922066	Tpr
ENSMUST00000126071.8	-0.184349006	MGI:1923827	Faf2
ENSMUST00000126879.7	-0.184349006	MGI:2176229	Zfp369
ENSMUST00000127188.2	-0.184349006	MGI:3648074	Tmem240
ENSMUST00000128119.1	-0.184349006	MGI:2446294	Megf8
ENSMUST00000129362.1	-0.184349006	MGI:1929878	Smoc1
ENSMUST00000130048.7	-0.184349006	MGI:106199	Vegfb
ENSMUST00000130916.7	-0.184349006	MGI:1891828	Becn1
ENSMUST00000133843.7	-0.184349006	MGI:6121585	Gm49369
ENSMUST00000148750.7	-0.184349006	MGI:1927555	Slc4a4
ENSMUST00000149359.1	-0.184349006	MGI:3050795	Mrtfb
ENSMUST00000153838.7	-0.184349006	MGI:1918177	Setd2
ENSMUST00000154584.8	-0.184349006	MGI:1913906	Eef1d
ENSMUST00000154617.7	-0.184349006	MGI:1270849	Rps6kb1
ENSMUST00000159917.7	-0.184349006	MGI:97502	Pcmt1
ENSMUST00000160844.9	-0.184349006	MGI:1096391	Nrxn1
ENSMUST00000168645.7	-0.184349006	MGI:2181434	Phc3
ENSMUST00000171474.1	-0.184349006	MGI:1917488	Rnf168
ENSMUST00000172306.2	-0.184349006	MGI:2387100	Dusp7
ENSMUST00000172435.7	-0.184349006	MGI:1917195	Fndc4
ENSMUST00000173107.7	-0.184349006	MGI:1315205	Slit2
ENSMUST00000177501.1	-0.184349006	MGI:1915068	Tmem70
ENSMUST00000181586.7	-0.184349006	MGI:88473	Cox4i1
ENSMUST00000183728.1	-0.184349006	MGI:3644991	Gm15013
ENSMUST00000188285.6	-0.184349006	MGI:99923	Kcnma1
ENSMUST00000188482.7	-0.184349006	MGI:97250	Myc
ENSMUST00000191275.6	-0.184349006	MGI:1890169	Erbin
ENSMUST00000192931.1	-0.184349006	MGI:1935169	Pcdhgb1
ENSMUST00000195112.1	-0.184349006	MGI:1935170	Pcdhgb2
ENSMUST00000204128.2	-0.184349006	MGI:1913581	Galntl6
ENSMUST00000204667.1	-0.184349006	MGI:1315214	Gpr162
ENSMUST00000209902.1	-0.184349006	MGI:1931221	Hdac9
ENSMUST00000211948.1	-0.184349006	MGI:2683541	Mast3
ENSMUST00000225661.2	-0.184349006	MGI:2443008	Trappc8

ENSMUST00000098402.4	-0.183182024	MGI:98347	Snrpn
ENSMUST00000233357.1	-0.183182024	MGI:6270609	Gm49909
ENSMUST00000114013.7	-0.183007177	MGI:97175	Map2
ENSMUST00000002839.8	-0.182635101	MGI:2388481	Ppp2r5d
ENSMUST00000016781.7	-0.182635101	MGI:1914292	Ift27
ENSMUST00000020283.4	-0.182635101	MGI:3037658	Macroh2a2
ENSMUST00000026760.2	-0.182635101	MGI:2177570	Tmem47
ENSMUST00000051301.5	-0.182635101	MGI:103079	Pura
ENSMUST00000064391.11	-0.182635101	MGI:1914121	Cpne8
ENSMUST00000069180.7	-0.182635101	MGI:1919168	Zcchc24
ENSMUST00000070748.9	-0.182635101	MGI:894670	Ldb2
ENSMUST00000071442.11	-0.182635101	MGI:1099802	Npdc1
ENSMUST00000102521.8	-0.182635101	MGI:3028623	Rap1gap2
ENSMUST00000103215.10	-0.182635101	MGI:1929258	Kcnip3
ENSMUST00000165418.8	-0.182635101	MGI:1332234	Zfp148
ENSMUST00000166487.9	-0.182635101	MGI:1915088	Dnajb11
ENSMUST00000030642.2	-0.18231658	MGI:1347045	Psmb2
ENSMUST00000027981.7	-0.182048466	MGI:1334459	Uap1
ENSMUST00000028610.9	-0.182048466	MGI:88271	Cat
ENSMUST00000031227.10	-0.182048466	MGI:1927246	Zfp326
ENSMUST00000045296.5	-0.182048466	MGI:108064	Siah1a
ENSMUST00000071585.9	-0.182048466	MGI:97440	Oprl1
ENSMUST00000123121.8	-0.182048466	MGI:1934928	Dusp15
ENSMUST00000233580.1	-0.182048466	MGI:2137520	Twsg1
ENSMUST00000010038.9	-0.181042453	MGI:1915076	Snap47
ENSMUST00000094361.10	-0.177520405	MGI:96250	Hsp90aa1
ENSMUST00000099356.9	-0.177520405	MGI:2145242	Arrdc3
ENSMUST00000034966.8	-0.177201173	MGI:1915141	Rpl4
ENSMUST00000034377.7	-0.176671291	MGI:2178076	Pla2g15
ENSMUST00000055676.3	-0.176671291	MGI:1096355	S1pr1
ENSMUST00000020999.6	-0.175241139	MGI:107979	Kif3c
ENSMUST00000038027.5	-0.175241139	MGI:95797	Gpi1
ENSMUST00000045743.12	-0.175241139	MGI:1336185	Prkab2
ENSMUST00000068725.9	-0.175241139	MGI:1914497	Mtarc2
ENSMUST00000070726.9	-0.175241139	MGI:2147279	Slc39a6
ENSMUST00000097590.4	-0.175241139	MGI:1913808	Lars
ENSMUST00000114630.2	-0.175241139	MGI:2448555	Tmem185a
ENSMUST00000030698.4	-0.174574539	MGI:1931027	Stx12
ENSMUST00000005954.8	-0.172323365	MGI:1927580	Bloc1s6

ENSMUST00000016569.10	-0.172323365	MGI:2140945	Pds5b
ENSMUST00000026436.9	-0.172323365	MGI:2137336	Baiap2
ENSMUST00000030986.14	-0.172323365	MGI:96829	Lrpap1
ENSMUST00000032114.7	-0.172323365	MGI:1929872	Mogs
ENSMUST00000054547.8	-0.172323365	MGI:99511	Ptpn11
ENSMUST00000063508.14	-0.172323365	MGI:95811	Gria4
ENSMUST00000066529.4	-0.172323365	MGI:97373	Npr3
ENSMUST00000076070.8	-0.172323365	MGI:1914447	Zcrb1
ENSMUST00000087211.8	-0.172323365	MGI:1289273	Ppp1r10
ENSMUST00000114002.8	-0.172323365	MGI:1338069	Itsn1
ENSMUST00000116456.9	-0.172323365	MGI:1335107	Cyth3
ENSMUST00000126008.7	-0.172323365	MGI:1915133	Uxs1
ENSMUST00000160726.3	-0.172323365	MGI:3034689	Fbll1
ENSMUST00000172142.7	-0.172323365	MGI:1928323	Nisch
ENSMUST00000103186.10	-0.171922188	MGI:2388633	Cltc
ENSMUST00000031697.8	-0.171275057	MGI:1349658	Cul1
ENSMUST00000034388.9	-0.171275057	MGI:1890520	Vps4a
ENSMUST00000109813.8	-0.170273816	MGI:88257	Camk2b
ENSMUST00000029423.8	-0.169182696	MGI:1194506	Serpini1
ENSMUST00000015622.7	-0.168801654	MGI:1860076	Rnf5
ENSMUST00000017975.6	-0.167900255	MGI:105926	Rab5a
ENSMUST00000004965.7	-0.167860668	MGI:1916192	Chmp2b
ENSMUST00000030140.2	-0.167860668	MGI:1914544	Elp1
ENSMUST00000115148.8	-0.167860668	MGI:1914953	Kirrel3
ENSMUST00000187541.6	-0.167860668	MGI:106217	Cacna1e
ENSMUST00000021345.13	-0.165883872	MGI:1933277	Gtf2a1
ENSMUST00000029845.14	-0.165883872	MGI:1916469	Ddah1
ENSMUST00000108530.1	-0.165533145	MGI:104900	Nup88
ENSMUST00000081542.5	-0.164104249	MGI:102663	Kcnd2
ENSMUST00000129339.7	-0.162440488	MGI:1929520	Eef1b2
ENSMUST00000018905.11	-0.160662251	MGI:1346040	Mpdu1
ENSMUST00000023132.4	-0.160662251	MGI:97523	Pde1b
ENSMUST00000025506.6	-0.160662251	MGI:1914060	Rbm22
ENSMUST00000030536.12	-0.160662251	MGI:1916193	Pink1
ENSMUST00000033544.13	-0.160662251	MGI:2389572	Brcc3
ENSMUST00000043526.14	-0.160662251	MGI:1921506	Cyld
ENSMUST00000044767.9	-0.160662251	MGI:106593	Neurod6
ENSMUST00000047614.3	-0.160662251	MGI:1298370	Pcdha12
ENSMUST00000069221.11	-0.160662251	MGI:1891690	Syncrip

ENSMUST00000079833.5	-0.160662251	MGI:96239	Hsf2
ENSMUST00000107094.1	-0.160662251	MGI:96646	Jun
ENSMUST00000193603.5	-0.160662251	MGI:2179435	Nlgn1
ENSMUST00000027174.9	-0.15983138	MGI:1933184	Nop58
ENSMUST00000047036.9	-0.15983138	MGI:1917583	Cd3eap
ENSMUST00000162812.7	-0.15983138	MGI:1336192	Pitpnm2
ENSMUST00000019577.9	-0.159411192	MGI:1926252	Gipc1
ENSMUST00000022766.7	-0.159411192	MGI:1915389	Tox4
ENSMUST00000036470.13	-0.159411192	MGI:2685477	Ccdc32
ENSMUST00000028426.8	-0.159157539	MGI:2442564	Cers6
ENSMUST00000047408.5	-0.159157539	MGI:2448730	Atcay
ENSMUST00000007980.6	-0.158987789	MGI:1924384	Hnrnpa0
ENSMUST00000065938.14	-0.158987789	MGI:1933158	Impa1
ENSMUST00000051064.8	-0.158600054	MGI:1914556	Zc2hc1a
ENSMUST00000011896.7	-0.15476755	MGI:97552	Pgam1
ENSMUST00000172387.7	-0.154649804	MGI:2137218	Mrpl13
ENSMUST00000022925.9	-0.153941104	MGI:1915385	Eif3h
ENSMUST00000110375.8	-0.153941104	MGI:2139090	Stard7
ENSMUST00000029803.11	-0.152870115	MGI:95305	Eif4e
ENSMUST00000103188.9	-0.152870115	MGI:1914576	1700037H04Rik
ENSMUST00000112707.2	-0.152870115	MGI:2141353	Lrrc8b
ENSMUST00000115578.9	-0.152870115	MGI:109353	Ufd1
ENSMUST00000028252.13	-0.152172957	MGI:1355324	Grb14
ENSMUST00000103242.4	-0.152172957	MGI:1916321	Tmem97
ENSMUST00000018430.6	-0.151293955	MGI:104880	Psmb6
ENSMUST00000075181.10	-0.151063641	MGI:2442213	Plekha1
ENSMUST00000116234.8	-0.151063641	MGI:99436	Arl1
ENSMUST00000140991.1	-0.151063641	MGI:1927479	Sap30bp
ENSMUST00000024739.13	-0.150949004	MGI:96247	Hsp90ab1
ENSMUST00000022197.14	-0.149466905	MGI:1349480	Scamp1
ENSMUST00000025642.13	-0.147840443	MGI:106247	Prpf19
ENSMUST00000000985.6	-0.147367882	MGI:1916339	Oxa1l
ENSMUST00000021424.4	-0.147367882	MGI:108074	Sptlc2
ENSMUST00000023071.7	-0.147367882	MGI:1915903	Samm50
ENSMUST00000032422.5	-0.147367882	MGI:2152539	Creld1
ENSMUST00000044384.4	-0.147367882	MGI:1919785	Aldh1b1
ENSMUST00000047799.12	-0.147367882	MGI:2143169	Acad11
ENSMUST00000059849.14	-0.147367882	MGI:1931035	Nelfb
ENSMUST00000060474.13	-0.147367882	MGI:1888939	Septin6

ENSMUST00000067491.13	-0.147367882	MGI:1350922	Cadps
ENSMUST00000080164.11	-0.147367882	MGI:1914149	Fip1l1
ENSMUST00000080371.7	-0.147367882	MGI:2384818	Mtss1
ENSMUST00000084885.11	-0.147367882	MGI:104889	Ubp1
ENSMUST00000110064.7	-0.147367882	MGI:1920149	Macrod2
ENSMUST00000112121.5	-0.147367882	MGI:1924015	Mlec
ENSMUST00000130491.2	-0.147367882	MGI:104982	Cebpg
ENSMUST00000151189.1	-0.147367882	MGI:1913926	Tmed7
ENSMUST00000156876.7	-0.147367882	MGI:1922925	Mfsd6
ENSMUST00000161074.7	-0.147367882	MGI:1914097	Hint3
ENSMUST00000197992.1	-0.147367882	MGI:1925076	Lhfpl3
ENSMUST00000008573.8	-0.14586527	MGI:1915393	Herpud2
ENSMUST00000057486.8	-0.14586527	MGI:1916089	Ankrd46
ENSMUST00000061143.14	-0.14586527	MGI:2384297	Map7d1
ENSMUST00000027494.10	-0.145356184	MGI:1913635	Ppp1r7
ENSMUST00000027777.11	-0.145356184	MGI:1340806	Parp1
ENSMUST00000000137.7	-0.14504763	MGI:1913963	Actr2
ENSMUST00000022960.3	-0.143098506	MGI:99257	Eif3e
ENSMUST00000035043.11	-0.142605568	MGI:1921375	Armc8
ENSMUST00000046094.5	-0.142605568	MGI:103016	Ppp1ca
ENSMUST00000049040.13	-0.142605568	MGI:1924059	Bri3bp
ENSMUST00000023116.6	-0.141507714	MGI:87880	Aco2
ENSMUST00000021170.8	-0.141450483	MGI:1914872	Mxra7
ENSMUST00000033577.10	-0.141450483	MGI:1914933	Pbdc1
ENSMUST00000043374.6	-0.141450483	MGI:1923392	Ppp1r14c
ENSMUST00000102830.9	-0.141450483	MGI:1343489	Mpdz
ENSMUST00000028607.12	-0.141370952	MGI:1858234	Caprin1
ENSMUST00000113820.8	-0.141344286	MGI:1923959	Arpc2
ENSMUST00000106112.1	-0.140803704	MGI:109326	Bnip3
ENSMUST00000027974.6	-0.138742498	MGI:1926157	Atf6
ENSMUST00000002400.6	-0.138345432	MGI:1915131	Mdp1
ENSMUST00000023677.9	-0.138345432	MGI:106341	Atp5o
ENSMUST00000023677.9	-0.138345432	MGI:106341	Atp5o
ENSMUST00000023677.9	-0.138345432	MGI:106341	Atp5o
ENSMUST00000023677.9	-0.138345432	MGI:106341	Atp5o
ENSMUST00000026572.10	-0.138345432	MGI:96224	Hras
ENSMUST00000029387.14	-0.138345432	MGI:1098684	Eif2a
ENSMUST00000041591.15	-0.138345432	MGI:1321390	Enpp2
ENSMUST00000137826.7	-0.138345432	MGI:1861305	Fntb

ENSMUST00000169536.7	-0.138345432	MGI:1919602	Zfyve27
ENSMUST00000080035.10	-0.137988202	MGI:2177151	Cd99l2
ENSMUST00000046587.7	-0.136567194	MGI:1928948	Scamp5
ENSMUST00000025836.5	-0.133490975	MGI:2137215	Mrpl11
ENSMUST00000061242.7	-0.132537326	MGI:99431	Arf1
ENSMUST00000001256.10	-0.132070583	MGI:1202889	Sema6b
ENSMUST00000018577.7	-0.132070583	MGI:1916229	Nol11
ENSMUST00000030665.6	-0.132070583	MGI:106014	Nudc
ENSMUST00000031411.14	-0.132070583	MGI:99600	Aldh2
ENSMUST00000034281.12	-0.132070583	MGI:2443793	6430548M08Rik
ENSMUST00000035721.13	-0.132070583	MGI:1914479	Prpf18
ENSMUST00000040718.5	-0.132070583	MGI:2655574	Ostm1
ENSMUST00000067663.13	-0.132070583	MGI:1098754	Psmc3
ENSMUST00000072055.12	-0.132070583	MGI:107760	Chka
ENSMUST00000081170.8	-0.132070583	MGI:1346523	Sgcb
ENSMUST00000097625.9	-0.132070583	MGI:97475	Pam
ENSMUST00000103013.9	-0.132070583	MGI:1919686	Tbcd
ENSMUST00000105362.7	-0.132070583	MGI:1917498	Dazap1
ENSMUST00000115443.7	-0.132070583	MGI:96969	Met
ENSMUST00000125541.1	-0.132070583	MGI:1916789	Trnp1
ENSMUST00000166237.7	-0.132070583	MGI:107560	Sema4a
ENSMUST00000166897.2	-0.132070583	MGI:106295	Tomm70a
ENSMUST00000179804.7	-0.132070583	MGI:1929288	Cldn12
ENSMUST00000217262.1	-0.132070583	MGI:1914224	Rnf187
ENSMUST00000239008.1	-0.132070583	MGI:3647581	Tmem178b
ENSMUST00000085519.12	-0.131817631	MGI:108447	Anp32a
ENSMUST00000026494.13	-0.130863186	MGI:2444521	Rnf165
ENSMUST00000028599.7	-0.130863186	MGI:1351825	Cstf3
ENSMUST00000036541.7	-0.130863186	MGI:1922673	Arl5a
ENSMUST00000037900.8	-0.130863186	MGI:2142747	Cpne7
ENSMUST00000109485.8	-0.130863186	MGI:2444531	Ralgapb
ENSMUST00000114034.8	-0.130863186	MGI:2442689	Lrrc49
ENSMUST00000028617.6	-0.130251525	MGI:1888993	Api5
ENSMUST00000032065.14	-0.130251525	MGI:1914131	Pcyox1
ENSMUST00000064656.7	-0.130251525	MGI:2143676	Zfp365
ENSMUST00000106252.8	-0.130251525	MGI:96799	Mycl
ENSMUST00000047632.13	-0.129634462	MGI:98447	Surf6
ENSMUST00000039913.8	-0.129457156	MGI:2442752	Tigar
ENSMUST00000019268.10	-0.129323882	MGI:1917188	Scrn1

ENSMUST00000009774.10	-0.129136874	MGI:1321161	Ppp2cb
ENSMUST00000099173.10	-0.129136874	MGI:1914454	Eif2s2
ENSMUST00000034630.14	-0.128761361	MGI:2670976	Fez1
ENSMUST00000203566.2	-0.126457093	MGI:1355332	Myadm
ENSMUST00000027684.10	-0.125757553	MGI:1915974	Arl8a
ENSMUST00000122375.7	-0.125287742	MGI:1918396	Golga7b
ENSMUST00000025181.17	-0.124502052	MGI:95904	H2-K1
ENSMUST00000088940.5	-0.123843977	MGI:1924356	Tmem181a
ENSMUST00000163123.2	-0.123587112	MGI:107956	Slc8a1
ENSMUST00000021466.9	-0.123178668	MGI:1921241	Atl1
ENSMUST00000068700.6	-0.122917227	MGI:1930140	Wdr6
ENSMUST00000002518.8	-0.122110274	MGI:95806	Tle5
ENSMUST00000033001.5	-0.12151506	MGI:1915050	Dgat2
ENSMUST00000043864.8	-0.12151506	MGI:2138811	Cdc123
ENSMUST00000115351.9	-0.12151506	MGI:97844	Rab3d
ENSMUST00000002790.13	-0.120490015	MGI:1339951	Cse1l
ENSMUST00000023460.6	-0.12014518	MGI:1915342	Ncbp2
ENSMUST00000031627.8	-0.119389411	MGI:2448536	Pdap1
ENSMUST00000023104.6	-0.119145442	MGI:1277218	Rpap3
ENSMUST00000053713.4	-0.119145442	MGI:2442159	Irf2bp1
ENSMUST00000146100.7	-0.119145442	MGI:1921265	Fcho1
ENSMUST00000154553.1	-0.119145442	MGI:1918689	Sft2d1
ENSMUST00000019854.12	-0.117741419	MGI:1914957	Mrpl24
ENSMUST00000026480.12	-0.117741419	MGI:1919604	Ttc4
ENSMUST00000102769.10	-0.117741419	MGI:1923786	Mmadhc
ENSMUST00000032949.13	-0.115802816	MGI:1345961	Coro1a
ENSMUST00000004560.11	-0.115498021	MGI:108093	Bid
ENSMUST00000070736.11	-0.115498021	MGI:108449	Adcyap1r1
ENSMUST00000006851.14	-0.114279742	MGI:1916482	Qrich1
ENSMUST00000008991.7	-0.114279742	MGI:1313261	Sptbn2
ENSMUST00000022701.6	-0.114279742	MGI:97874	Rb1
ENSMUST00000023687.8	-0.114279742	MGI:107654	Ifngr2
ENSMUST00000025590.10	-0.114279742	MGI:97447	Osbp
ENSMUST00000027040.12	-0.114279742	MGI:1341850	Rb1cc1
ENSMUST00000032899.11	-0.114279742	MGI:1929274	1110004F10Rik
ENSMUST00000034702.5	-0.114279742	MGI:1917332	Lysmd2
ENSMUST00000036540.11	-0.114279742	MGI:1920000	Fam117b
ENSMUST00000037488.7	-0.114279742	MGI:1918006	Dock4
ENSMUST00000038364.14	-0.114279742	MGI:2442179	Fstl5

ENSMUST00000046371.12	-0.114279742	MGI:2384575	Plppr2
ENSMUST00000052838.10	-0.114279742	MGI:2443157	Mib1
ENSMUST00000060945.11	-0.114279742	MGI:2136171	Aff4
ENSMUST00000061222.8	-0.114279742	MGI:2685141	Kbtbd7
ENSMUST00000069965.8	-0.114279742	MGI:97517	Cdk17
ENSMUST00000073899.5	-0.114279742	MGI:1859545	Syt7
ENSMUST00000076124.6	-0.114279742	MGI:1916205	Srrm4
ENSMUST00000087374.9	-0.114279742	MGI:1345162	Adam23
ENSMUST00000088452.10	-0.114279742	MGI:2145895	Slc38a1
ENSMUST00000093362.11	-0.114279742	MGI:1919296	Urgcp
ENSMUST00000106557.7	-0.114279742	MGI:2141942	Ccp110
ENSMUST00000121805.8	-0.114279742	MGI:1349762	Dpysl3
ENSMUST00000144211.1	-0.114279742	MGI:1920309	Srrm3
ENSMUST00000166873.8	-0.114279742	MGI:107436	Cdh10
ENSMUST00000189661.6	-0.114279742	MGI:104665	Soat1
ENSMUST00000207477.1	-0.114279742	MGI:2685951	Myo16
ENSMUST00000100244.9	-0.11389344	MGI:1860437	Olfm1
ENSMUST00000113364.9	-0.11389344	MGI:2442849	Pcdh11x
ENSMUST00000066257.5	-0.112324665	MGI:893579	Khdrbs1
ENSMUST00000027185.10	-0.112056176	MGI:2144047	Stradb
ENSMUST00000047652.5	-0.112056176	MGI:1196325	Tspan14
ENSMUST00000070323.11	-0.112056176	MGI:1919253	Synpr
ENSMUST00000102889.9	-0.112056176	MGI:2140220	Ecpas
ENSMUST00000035155.7	-0.111707974	MGI:107283	Rab6b
ENSMUST00000025774.10	-0.10820625	MGI:2441856	Sf3b2
ENSMUST00000042990.6	-0.10820625	MGI:1914120	Serbp1
ENSMUST00000127206.7	-0.10820625	MGI:1098271	Rgs2
ENSMUST00000003912.6	-0.107578225	MGI:88252	Calr
ENSMUST00000022341.6	-0.107578225	MGI:1915295	Rtraf
ENSMUST00000127477.7	-0.107578225	MGI:1913296	Ndufb5
ENSMUST00000001319.14	-0.10610553	MGI:105097	Efnb2
ENSMUST00000001620.12	-0.10610553	MGI:104860	Fxr1
ENSMUST00000022954.6	-0.10610553	MGI:1313312	Khdrbs3
ENSMUST00000025215.9	-0.10610553	MGI:1932040	Sil1
ENSMUST00000027384.5	-0.10610553	MGI:1351352	Atic
ENSMUST00000033934.4	-0.10610553	MGI:1913153	Mrps31
ENSMUST00000036977.8	-0.10610553	MGI:2685015	Mtg1
ENSMUST00000065308.12	-0.10610553	MGI:1859169	Azin1
ENSMUST00000095224.10	-0.10610553	MGI:2146974	Safb

ENSMUST00000103212.9	-0.10610553	MGI:1927086	Ube4b
ENSMUST00000113822.2	-0.10610553	MGI:2385612	Lrfn4
ENSMUST00000161356.7	-0.10610553	MGI:103022	Reln
ENSMUST00000034699.7	-0.105737898	MGI:103032	Scg3
ENSMUST00000220041.1	-0.105737898	MGI:1858210	Stx7
ENSMUST00000000356.9	-0.104853405	MGI:1344344	Dazap2
ENSMUST00000028727.10	-0.10460379	MGI:98331	Snap25
ENSMUST00000169094.7	-0.10456309	MGI:2442040	G3bp2
ENSMUST00000021347.11	-0.104493167	MGI:1329016	Sel1l
ENSMUST00000091288.12	-0.103787354	MGI:97769	Prnp
ENSMUST00000044277.9	-0.102421585	MGI:1922858	Chmp4b
ENSMUST00000005548.7	-0.102238569	MGI:96163	Hmox1
ENSMUST00000050000.15	-0.102148208	MGI:107363	Stxbp1
ENSMUST00000116444.9	-0.101920691	MGI:1336200	Hpca
ENSMUST00000043951.9	-0.101781172	MGI:1917446	Actr1b
ENSMUST00000057598.6	-0.101781172	MGI:1920102	Mblac2
ENSMUST00000063091.12	-0.101781172	MGI:95602	Fyn
ENSMUST00000074616.6	-0.101781172	MGI:2145245	Srek1
ENSMUST00000102724.4	-0.101781172	MGI:109419	Faf1
ENSMUST00000037324.11	-0.09922276	MGI:103575	Skp1a
ENSMUST00000026987.11	-0.099105276	MGI:107862	Nop16
ENSMUST00000145910.8	-0.099105276	MGI:1333757	Strn
ENSMUST00000101045.9	-0.098068801	MGI:105368	Atp2b2
ENSMUST00000113278.8	-0.098068801	MGI:87977	Ak1
ENSMUST00000025421.8	-0.097286137	MGI:1919374	Seh1l
ENSMUST00000031862.13	-0.097286137	MGI:108515	Cbx3
ENSMUST00000057742.14	-0.097286137	MGI:1921270	Cpne4
ENSMUST00000000579.2	-0.093329814	MGI:98371	Sox9
ENSMUST00000020801.13	-0.093329814	MGI:1921383	Smg8
ENSMUST00000022310.6	-0.093329814	MGI:1913276	Ngly1
ENSMUST00000022855.11	-0.093329814	MGI:1915082	Brix1
ENSMUST00000023467.8	-0.093329814	MGI:1339984	Pak2
ENSMUST00000024958.8	-0.093329814	MGI:2442952	Caskin1
ENSMUST00000027287.10	-0.093329814	MGI:1931123	Inpp4a
ENSMUST00000029676.11	-0.093329814	MGI:1333882	Adam15
ENSMUST00000030032.12	-0.093329814	MGI:1926810	Tmeff1
ENSMUST00000030169.14	-0.093329814	MGI:1913842	Stoml2
ENSMUST00000030763.12	-0.093329814	MGI:894318	Cdk14
ENSMUST00000037977.14	-0.093329814	MGI:1196419	Ccpg1

ENSMUST00000038431.7	-0.093329814	MGI:1343087	Pdk2
ENSMUST00000041047.3	-0.093329814	MGI:2387123	Lnpep
ENSMUST00000041055.8	-0.093329814	MGI:1923809	Atg2b
ENSMUST00000045807.13	-0.093329814	MGI:2144566	Tsr1
ENSMUST00000048043.11	-0.093329814	MGI:2444283	Coro2b
ENSMUST00000048545.9	-0.093329814	MGI:2442985	Arglu1
ENSMUST00000054883.3	-0.093329814	MGI:102564	Pou3f3
ENSMUST00000059271.12	-0.093329814	MGI:2386964	St7l
ENSMUST00000062821.12	-0.093329814	MGI:2155091	Emid1
ENSMUST00000082408.1	-0.093329814	MGI:99927	mt-Atp6
ENSMUST00000085546.12	-0.093329814	MGI:96113	Hmgb1
ENSMUST00000088716.11	-0.093329814	MGI:1203524	Smarcc1
ENSMUST00000089017.11	-0.093329814	MGI:1196463	Fndc3a
ENSMUST00000101018.10	-0.093329814	MGI:2145043	Cep170b
ENSMUST00000102518.9	-0.093329814	MGI:1101357	Ece1
ENSMUST00000102754.10	-0.093329814	MGI:109281	Pkp4
ENSMUST00000113026.1	-0.093329814	MGI:1914139	Rnf128
ENSMUST00000113337.9	-0.093329814	MGI:1861099	Ubr2
ENSMUST00000115050.9	-0.093329814	MGI:1915416	Spire1
ENSMUST00000115072.7	-0.093329814	MGI:1098583	Stag2
ENSMUST00000117654.2	-0.093329814	MGI:1100877	Tbrg1
ENSMUST00000136381.7	-0.093329814	MGI:2443155	Sidt1
ENSMUST00000138667.1	-0.093329814	MGI:5313163	Gm20716
ENSMUST00000145614.1	-0.093329814	MGI:1333830	Mrps26
ENSMUST00000150042.7	-0.093329814	MGI:1289321	Mrpl48
ENSMUST00000200137.4	-0.093329814	MGI:2676665	Lrrc7
ENSMUST00000209117.1	-0.093329814	MGI:1929510	Zfand6
ENSMUST00000122941.7	-0.093042956	MGI:1341890	Zfr
ENSMUST00000006692.5	-0.091977386	MGI:2179327	Mvd
ENSMUST00000021296.6	-0.091505655	MGI:1923797	Tmem101
ENSMUST00000025515.6	-0.091505655	MGI:2442418	Tmx3
ENSMUST00000027877.6	-0.091505655	MGI:107566	Kifap3
ENSMUST00000038388.6	-0.091505655	MGI:2441787	Washc4
ENSMUST00000054251.12	-0.091505655	MGI:1920393	Wdr37
ENSMUST00000098782.3	-0.091505655	MGI:2685357	Layn
ENSMUST00000102487.3	-0.091505655	MGI:1098672	Szrd1
ENSMUST00000111064.2	-0.091505655	MGI:108018	Ntsr2
ENSMUST00000144331.7	-0.091505655	MGI:105084	Satb1
ENSMUST00000003513.10	-0.090579366	MGI:1913644	Nosip

ENSMUST00000017552.12	-0.090579366	MGI:102522	Cacnb1
ENSMUST00000030315.12	-0.090579366	MGI:1931749	Pum1
ENSMUST00000032066.12	-0.090579366	MGI:98724	Tgfa
ENSMUST00000034738.13	-0.090579366	MGI:2681840	Rsl24d1
ENSMUST00000052761.8	-0.090579366	MGI:1099463	Idh3g
ENSMUST00000059595.10	-0.090579366	MGI:97595	Prkca
ENSMUST00000144668.7	-0.090579366	MGI:1914672	Dhdds
ENSMUST00000028335.12	-0.09001896	MGI:95819	Grin1
ENSMUST00000032457.16	-0.09001896	MGI:1921494	Atg7
ENSMUST00000002737.6	-0.089643405	MGI:1913650	Alkbh7
ENSMUST00000025955.7	-0.089643405	MGI:95301	Eif3a
ENSMUST00000071329.7	-0.089643405	MGI:107701	Bckdha
ENSMUST00000106333.7	-0.089643405	MGI:2385295	Sez6l2
ENSMUST00000035033.6	-0.08937419	MGI:1354962	Copb2
ENSMUST00000100729.8	-0.08937419	MGI:1914401	Psmd9
ENSMUST00000114340.8	-0.08917175	MGI:87918	Add1
ENSMUST00000115816.2	-0.089013983	MGI:1932407	Celf4
ENSMUST00000000896.10	-0.08888757	MGI:1891701	Pxmp4
ENSMUST00000042664.9	-0.08888757	MGI:1203732	Slc16a2
ENSMUST00000180095.3	-0.088113023	MGI:1353554	Mlf2
ENSMUST00000026704.13	-0.08621086	MGI:107183	Cct8
ENSMUST00000039659.8	-0.085697102	MGI:88284	Cbr1
ENSMUST00000015435.10	-0.085439576	MGI:99846	Gdi1
ENSMUST00000023750.8	-0.085107834	MGI:1919643	Faim2
ENSMUST00000097739.4	-0.084664395	MGI:2443342	Pgap1
ENSMUST00000007012.5	-0.08267197	MGI:98352	Sod2
ENSMUST00000072580.11	-0.082427857	MGI:3039582	Lmtk3
ENSMUST00000027502.15	-0.08152411	MGI:1913865	Atg4b
ENSMUST00000065358.8	-0.08152411	MGI:1913449	Commd4
ENSMUST00000230931.1	-0.08152411	MGI:1914161	Nudt16l1
ENSMUST00000198199.4	-0.080504723	MGI:99458	Mef2c
ENSMUST00000062991.8	-0.080249971	MGI:1334416	Grpel2
ENSMUST00000076349.11	-0.080249971	MGI:95810	Gria3
ENSMUST00000006027.6	-0.079265387	MGI:1270152	Reep5
ENSMUST00000021243.15	-0.078319049	MGI:1343133	Slc35b1
ENSMUST00000023069.8	-0.078319049	MGI:1920475	Fam118a
ENSMUST00000026625.6	-0.078319049	MGI:1916884	Clybl
ENSMUST00000043498.8	-0.078319049	MGI:1343091	Hdac3
ENSMUST00000045450.6	-0.078319049	MGI:1924315	Ints7

ENSMUST00000045898.3	-0.078319049	MGI:2147987	Pcyt1b
ENSMUST00000056820.12	-0.078319049	MGI:1334255	Cyth2
ENSMUST00000102814.4	-0.078319049	MGI:98159	Rps6
ENSMUST00000117798.7	-0.078319049	MGI:2143792	Zfr2
ENSMUST00000159879.1	-0.078319049	MGI:106652	Ro60
ENSMUST00000209469.1	-0.075139246	MGI:101757	Cfl1
ENSMUST00000031383.13	-0.075133662	MGI:1333112	Ran
ENSMUST00000091201.6	-0.075115894	MGI:2442308	Arl15
ENSMUST00000034392.12	-0.075101118	MGI:1913414	Nip7
ENSMUST00000000365.2	-0.075047179	MGI:1916245	Mcts1
ENSMUST00000002198.3	-0.075047179	MGI:1914715	Sf3a1
ENSMUST00000017365.14	-0.075047179	MGI:98858	Psmd3
ENSMUST00000021443.6	-0.075047179	MGI:1342005	Mthfd1
ENSMUST00000032476.10	-0.075047179	MGI:95757	Slc2a3
ENSMUST00000049074.12	-0.075047179	MGI:102695	Ptprf
ENSMUST00000093138.12	-0.075047179	MGI:1924045	Tbc1d9b
ENSMUST00000097588.8	-0.075047179	MGI:109605	Srgap2
ENSMUST00000102973.3	-0.075047179	MGI:87879	Aco1
ENSMUST00000102989.9	-0.075047179	MGI:1202298	Nmt2
ENSMUST00000206832.1	-0.075047179	MGI:2443517	Hnrnpul1
ENSMUST00000110890.7	-0.07469167	MGI:97759	Prkar1b
ENSMUST00000047357.9	-0.072882102	MGI:1861601	Cpsf2
ENSMUST00000067744.7	-0.072882102	MGI:2146370	Cggbp1
ENSMUST00000020717.11	-0.071305908	MGI:99434	Arf5
ENSMUST00000079746.9	-0.070236313	MGI:1201685	Ctbp1
ENSMUST00000047281.9	-0.070074349	MGI:1922833	Trir
ENSMUST00000081982.11	-0.069718118	MGI:2139080	Dzank1
ENSMUST00000115524.7	-0.069718118	MGI:1915291	Mid1ip1
ENSMUST00000006462.13	-0.068367334	MGI:107809	Aamp
ENSMUST00000071026.9	-0.068367334	MGI:106248	Eif5a
ENSMUST00000002280.10	-0.068294443	MGI:1919247	Smg9
ENSMUST00000003436.11	-0.068294443	MGI:106388	Abhd17a
ENSMUST00000004036.5	-0.068294443	MGI:109196	Efnb3
ENSMUST00000018803.11	-0.068294443	MGI:2144151	Pnpo
ENSMUST00000020270.5	-0.068294443	MGI:2182303	Ddx50
ENSMUST00000020681.9	-0.068294443	MGI:2385598	Slu7
ENSMUST00000021085.10	-0.068294443	MGI:3046173	Nup85
ENSMUST00000023165.8	-0.068294443	MGI:1098280	Crebbp
ENSMUST00000025503.9	-0.068294443	MGI:1913557	Isoc1

ENSMUST00000026135.14	-0.068294443	MGI:1916188	Aspscr1
ENSMUST00000026994.13	-0.068294443	MGI:894682	Unc5a
ENSMUST00000028841.13	-0.068294443	MGI:1934029	Usp8
ENSMUST00000032754.8	-0.068294443	MGI:107559	Sema4b
ENSMUST00000033006.13	-0.068294443	MGI:1914961	Nsmce1
ENSMUST00000033828.6	-0.068294443	MGI:95660	Gas6
ENSMUST00000034121.10	-0.068294443	MGI:107286	Man2b1
ENSMUST00000035242.8	-0.068294443	MGI:105065	Rab24
ENSMUST00000038890.5	-0.068294443	MGI:2142979	Dennd4a
ENSMUST00000040017.7	-0.068294443	MGI:2182066	Mios
ENSMUST00000043775.8	-0.068294443	MGI:1923356	Kdm3b
ENSMUST00000046687.15	-0.068294443	MGI:2385287	Spon1
ENSMUST00000055935.10	-0.068294443	MGI:1935205	Pcdhgc5
ENSMUST00000057293.7	-0.068294443	MGI:2679002	Prr12
ENSMUST00000061047.6	-0.068294443	MGI:2138327	Phlpp1
ENSMUST00000064454.11	-0.068294443	MGI:2444248	Gcn1
ENSMUST00000074875.10	-0.068294443	MGI:2385848	Bcas3
ENSMUST00000076734.7	-0.068294443	MGI:1925212	Scaf8
ENSMUST00000077626.12	-0.068294443	MGI:3039607	Pigu
ENSMUST00000092794.11	-0.068294443	MGI:1923876	Msi2
ENSMUST00000093772.3	-0.068294443	MGI:2670992	Zfp651
ENSMUST00000094340.3	-0.068294443	MGI:2181178	Mkrn3
ENSMUST00000095220.3	-0.068294443	MGI:1915878	Fbxw9
ENSMUST00000095852.4	-0.068294443	MGI:95813	Grid2
ENSMUST00000109445.8	-0.068294443	MGI:1321152	Ptprt
ENSMUST00000110516.2	-0.068294443	MGI:2444115	Hecw1
ENSMUST00000110865.1	-0.068294443	MGI:2442201	Adap1
ENSMUST00000112606.7	-0.068294443	MGI:2442409	Dpp10
ENSMUST00000112736.7	-0.068294443	MGI:1100499	Vps4b
ENSMUST00000113504.9	-0.068294443	MGI:1316736	Men1
ENSMUST00000114355.1	-0.068294443	MGI:1916096	Rnf208
ENSMUST00000117549.7	-0.068294443	MGI:2154580	Ube4a
ENSMUST00000118592.7	-0.068294443	MGI:2445022	Ccdc186
ENSMUST00000119261.7	-0.068294443	MGI:2446249	Edc4
ENSMUST00000165665.8	-0.068294443	MGI:1916882	Arhgef12
ENSMUST00000182006.3	-0.068294443	MGI:5439400	Iqschfp
ENSMUST00000185072.7	-0.068294443	MGI:5435040	Entpd4b
ENSMUST00000191234.6	-0.068294443	MGI:1923447	Nkain2
ENSMUST00000200758.3	-0.068294443	MGI:105098	Ube3a

ENSMUST00000217854.1	-0.068294443	MGI:2443834	Ppfia2
ENSMUST00000224047.1	-0.068294443	MGI:1333811	Mbd1
ENSMUST00000238937.1	-0.068294443	MGI:1923019	Plppr5
ENSMUST00000022429.8	-0.067903174	MGI:99433	Arf4
ENSMUST00000043520.4	-0.065180293	MGI:1914072	Fbxo25
ENSMUST00000142456.1	-0.065180293	MGI:6303253	Gm50364
ENSMUST00000005798.8	-0.064842233	MGI:1919433	Snx6
ENSMUST00000030586.14	-0.064842233	MGI:1913514	Ccdc28b
ENSMUST00000031167.5	-0.064842233	MGI:2388280	Polr2b
ENSMUST00000033839.8	-0.064842233	MGI:1913673	Coprs
ENSMUST00000035227.7	-0.064842233	MGI:1913507	Nicn1
ENSMUST00000036211.7	-0.064842233	MGI:101909	Gpr12
ENSMUST00000040434.8	-0.064842233	MGI:1919976	Tbcc
ENSMUST00000042844.6	-0.064842233	MGI:104591	Nbl1
ENSMUST00000049149.14	-0.064842233	MGI:96828	Lrp1
ENSMUST00000055262.12	-0.064842233	MGI:1855688	Vti1b
ENSMUST00000059045.7	-0.064842233	MGI:1923576	Exosc4
ENSMUST00000068825.7	-0.064842233	MGI:1889001	Nub1
ENSMUST00000069652.7	-0.064842233	MGI:1916043	Rab3gap2
ENSMUST00000105097.2	-0.064842233	MGI:1914513	Zswim6
ENSMUST00000119068.7	-0.064842233	MGI:1920152	Spock3
ENSMUST00000139167.2	-0.064842233	MGI:1923776	Gatc
ENSMUST00000144950.7	-0.064842233	MGI:1914285	Dnajb4
ENSMUST00000193254.5	-0.064842233	MGI:88041	Apeh
ENSMUST00000040766.8	-0.063665054	MGI:1915831	Tmed10
ENSMUST00000167861.7	-0.063333099	MGI:1914366	Cuedc2
ENSMUST00000103045.3	-0.063111264	MGI:1277137	Stmn3
ENSMUST00000018431.12	-0.063071377	MGI:107380	Spag7
ENSMUST00000057525.13	-0.063071377	MGI:1860040	Trim3
ENSMUST00000077755.10	-0.061827361	MGI:1915021	Arpc5
ENSMUST00000160507.7	-0.059699685	MGI:1306775	Sucla2
ENSMUST00000081769.12	-0.059268925	MGI:1329025	Edil3
ENSMUST00000085192.6	-0.059173736	MGI:1915077	Aldh6a1
ENSMUST00000024005.7	-0.058735585	MGI:98289	Scg5
ENSMUST00000035034.9	-0.058330097	MGI:1928137	Mrps22
ENSMUST00000089140.12	-0.058330097	MGI:3042273	Nsfl1c
ENSMUST00000089378.4	-0.058330097	MGI:1919282	Pdxp
ENSMUST00000114513.8	-0.058330097	MGI:95752	Gls
ENSMUST00000031160.15	-0.057583074	MGI:1914304	Paics

ENSMUST00000015800.15	-0.05729881	MGI:105384	Hspa8
ENSMUST00000018156.11	-0.056349759	MGI:2180784	Rac3
ENSMUST00000023762.12	-0.056349759	MGI:1919199	Cers5
ENSMUST00000024620.7	-0.056349759	MGI:1914295	Riok2
ENSMUST00000025305.15	-0.056349759	MGI:1914223	Mrps18b
ENSMUST00000039303.6	-0.056349759	MGI:104963	Npy1r
ENSMUST00000041905.7	-0.056349759	MGI:6121524	Gm49336
ENSMUST00000043464.13	-0.056349759	MGI:1913765	Cul7
ENSMUST00000044509.6	-0.056349759	MGI:106244	Tspyl2
ENSMUST00000044851.7	-0.056349759	MGI:1935229	Pcdhga12
ENSMUST00000060531.15	-0.056349759	MGI:1922368	Lrrc8d
ENSMUST00000065904.4	-0.056349759	MGI:1354960	Hs6st3
ENSMUST00000076532.13	-0.056349759	MGI:103123	Serpinb6a
ENSMUST00000084953.12	-0.056349759	MGI:1341865	Mark3
ENSMUST00000164843.7	-0.056349759	MGI:88263	Capn1
ENSMUST00000209885.1	-0.056349759	MGI:101934	Tfdp1
ENSMUST00000220377.1	-0.056349759	MGI:101857	Usp15
ENSMUST00000102928.4	-0.055708796	MGI:700006	Dbnl
ENSMUST00000128646.7	-0.055708796	MGI:2659021	Phactr1
ENSMUST00000233514.2	-0.055708796	MGI:1924140	Memo1
ENSMUST00000046983.9	-0.053032097	MGI:98325	Smpd1
ENSMUST00000021148.12	-0.052954474	MGI:1914378	Ube2g1
ENSMUST00000168846.2	-0.052954474	MGI:108411	Prkag1
ENSMUST00000067664.9	-0.052878271	MGI:894689	Ywhae
ENSMUST00000100497.10	-0.051114204	MGI:87904	Actb
ENSMUST00000023686.14	-0.050712491	MGI:1925225	Tmem50b
ENSMUST00000111416.6	-0.050496786	MGI:1196370	Ildr2
ENSMUST00000146623.7	-0.050496786	MGI:1289257	Gpd1l
ENSMUST00000195612.1	-0.050089023	MGI:1353609	Zbtb18
ENSMUST00000024873.6	-0.049957725	MGI:1915114	Yipf4
ENSMUST00000030738.7	-0.049957725	MGI:1914455	Utp11
ENSMUST00000033999.7	-0.049957725	MGI:893597	Frg1
ENSMUST00000034303.2	-0.049957725	MGI:1915783	Mphosph6
ENSMUST00000066039.7	-0.049957725	MGI:1932622	Cstf2t
ENSMUST00000115995.3	-0.049957725	MGI:1928740	Fads3
ENSMUST00000165316.7	-0.049957725	MGI:2145219	Iars
ENSMUST00000105367.7	-0.049497569	MGI:1913293	Atp5d
ENSMUST00000000175.5	-0.04849507	MGI:1914175	Sdhd
ENSMUST00000021332.9	-0.04849507	MGI:1353460	Fkbp3

ENSMUST00000022494.9	-0.045976681	MGI:1915427	Ebpl
ENSMUST00000022867.4	-0.045976681	MGI:1890494	Laptm4b
ENSMUST00000038107.8	-0.045976681	MGI:1306784	Cited2
ENSMUST00000041139.8	-0.045976681	MGI:2442982	Rab8b
ENSMUST00000116468.1	-0.045976681	MGI:1922589	Mphosph8
ENSMUST00000163765.2	-0.045976681	MGI:2181182	Nup155
ENSMUST00000021197.9	-0.045952291	MGI:1345186	Blmh
ENSMUST00000030896.14	-0.045952291	MGI:1915058	Tprgl
ENSMUST00000170953.2	-0.045931195	MGI:1915302	Rps13
ENSMUST00000008297.4	-0.044327573	MGI:2178323	Clstn3
ENSMUST00000025679.10	-0.043258965	MGI:2147616	Otub1
ENSMUST00000114059.9	-0.043258965	MGI:104807	Pls3
ENSMUST00000006496.14	-0.042984745	MGI:1924096	Rps9
ENSMUST00000020522.8	-0.041285575	MGI:97547	Pfkl
ENSMUST00000028123.3	-0.041285575	MGI:95634	Gad2
ENSMUST00000061925.4	-0.041285575	MGI:2442605	Plcxd3
ENSMUST00000113482.7	-0.041285575	MGI:2443699	Fubp3
ENSMUST00000022977.13	-0.040894692	MGI:109296	Sqle
ENSMUST00000003720.4	-0.039787556	MGI:1921364	Crot
ENSMUST00000105675.7	-0.039655866	MGI:2135637	Park7
ENSMUST00000177005.7	-0.038611604	MGI:1913318	Glmp
ENSMUST00000164756.3	-0.038525215	MGI:1889651	Eid1
ENSMUST00000002844.13	-0.037844295	MGI:1351622	Mrpl2
ENSMUST00000016323.10	-0.037844295	MGI:2388073	Camk1g
ENSMUST00000023113.6	-0.037844295	MGI:1926179	Polr3h
ENSMUST00000025462.6	-0.037844295	MGI:1915981	Rbfa
ENSMUST00000025520.9	-0.037844295	MGI:2147363	Slc6a7
ENSMUST00000025827.9	-0.037844295	MGI:1336159	Minpp1
ENSMUST00000026670.4	-0.037844295	MGI:107811	Nptx1
ENSMUST00000026827.15	-0.037844295	MGI:1915597	Mettl26
ENSMUST00000027856.12	-0.037844295	MGI:1921356	Dcaf6
ENSMUST00000028829.12	-0.037844295	MGI:2150016	Spred1
ENSMUST00000029574.12	-0.037844295	MGI:98926	Vcam1
ENSMUST00000030010.3	-0.037844295	MGI:99607	Abca1
ENSMUST00000030138.8	-0.037844295	MGI:2140151	Nol6
ENSMUST00000030879.11	-0.037844295	MGI:1347049	Clcn6
ENSMUST00000031617.12	-0.037844295	MGI:108057	Rpl6
ENSMUST00000033575.6	-0.037844295	MGI:2148316	Magee2
ENSMUST00000033929.5	-0.037844295	MGI:1341087	Tnks

ENSMUST00000034204.10	-0.037844295	MGI:1915469	Nudt21
ENSMUST00000034243.6	-0.037844295	MGI:109320	Mmp15
ENSMUST00000035645.11	-0.037844295	MGI:2140371	Rusc2
ENSMUST00000035973.4	-0.037844295	MGI:1914705	Klhl13
ENSMUST00000037557.8	-0.037844295	MGI:1914324	Mon2
ENSMUST00000038369.10	-0.037844295	MGI:1919185	Cipc
ENSMUST00000038551.7	-0.037844295	MGI:1921075	Ppp1r21
ENSMUST00000039259.6	-0.037844295	MGI:3580016	Agap2
ENSMUST00000039674.12	-0.037844295	MGI:2445415	Pknox2
ENSMUST00000040021.11	-0.037844295	MGI:2144837	Ptpn23
ENSMUST00000040787.12	-0.037844295	MGI:2139746	Ankrd13c
ENSMUST00000041369.7	-0.037844295	MGI:2385459	Socs5
ENSMUST00000046709.8	-0.037844295	MGI:1890948	Supt16
ENSMUST00000049020.8	-0.037844295	MGI:2667176	Irgq
ENSMUST00000050248.8	-0.037844295	MGI:2443876	Zbtb11
ENSMUST00000054014.8	-0.037844295	MGI:1914290	Ddx17
ENSMUST00000057783.5	-0.037844295	MGI:1920260	Gpr22
ENSMUST00000058326.5	-0.037844295	MGI:1354373	Sall2
ENSMUST00000059250.7	-0.037844295	MGI:1196337	Brms1l
ENSMUST00000059498.11	-0.037844295	MGI:1914217	Edem3
ENSMUST00000060396.6	-0.037844295	MGI:1914703	Slc25a46
ENSMUST00000063042.10	-0.037844295	MGI:88151	Glb1
ENSMUST00000064371.13	-0.037844295	MGI:1923484	Magi3
ENSMUST00000066439.7	-0.037844295	MGI:1351611	Exoc6
ENSMUST00000066743.10	-0.037844295	MGI:2448562	Adnp2
ENSMUST00000076463.11	-0.037844295	MGI:1915776	Gpr155
ENSMUST00000077282.6	-0.037844295	MGI:2147749	Atrnl1
ENSMUST00000085585.11	-0.037844295	MGI:1914320	Lsm14a
ENSMUST00000087556.6	-0.037844295	MGI:2385088	Smc5
ENSMUST00000095027.8	-0.037844295	MGI:106927	Aff3
ENSMUST00000095074.3	-0.037844295	MGI:1919154	Paqr7
ENSMUST00000096495.10	-0.037844295	MGI:1349442	Med14
ENSMUST00000100784.8	-0.037844295	MGI:103309	Flot2
ENSMUST00000102715.3	-0.037844295	MGI:1858416	Stk39
ENSMUST00000103109.3	-0.037844295	MGI:1858201	Cntnap1
ENSMUST00000106017.7	-0.037844295	MGI:2451244	Adgrb2
ENSMUST00000106930.7	-0.037844295	MGI:1919935	Dnajc6
ENSMUST00000107153.2	-0.037844295	MGI:2682305	Rsf1
ENSMUST00000107894.7	-0.037844295	MGI:1889276	Dgke

ENSMUST00000109554.2	-0.037844295	MGI:1328310	Zc3h7b
ENSMUST00000109860.7	-0.037844295	MGI:1915769	Eml1
ENSMUST00000111030.5	-0.037844295	MGI:108360	Enah
ENSMUST00000111605.8	-0.037844295	MGI:2446193	Tnks1bp1
ENSMUST00000113493.7	-0.037844295	MGI:2445020	Exoc1
ENSMUST00000118316.7	-0.037844295	MGI:2687207	N4bp2l2
ENSMUST00000121465.2	-0.037844295	MGI:1914098	Fuca2
ENSMUST00000163139.7	-0.037844295	MGI:107685	Plxna1
ENSMUST00000172809.1	-0.037844295	MGI:2384801	Gps1
ENSMUST00000174552.7	-0.037844295	MGI:1920179	Dip2c
ENSMUST00000201185.3	-0.037844295	MGI:95851	Gsn
ENSMUST00000214828.1	-0.037844295	MGI:2384573	Ttc13
ENSMUST00000022927.10	-0.037663936	MGI:108016	Rad21
ENSMUST00000025208.6	-0.037663936	MGI:1923844	Dnajc18
ENSMUST00000033913.10	-0.035198435	MGI:1343154	Dctn6
ENSMUST00000000188.11	-0.034921239	MGI:88314	Ccnd2
ENSMUST00000014640.8	-0.034921239	MGI:2145661	Ankrd28
ENSMUST00000020217.6	-0.034921239	MGI:1918457	Nudt4
ENSMUST00000027975.7	-0.034921239	MGI:891978	Phyh
ENSMUST00000031597.6	-0.034921239	MGI:1919022	Plbd2
ENSMUST00000032172.13	-0.034921239	MGI:1913348	Chchd6
ENSMUST00000034878.11	-0.034921239	MGI:106402	Tmem30a
ENSMUST00000034946.14	-0.034921239	MGI:1928395	Snx1
ENSMUST00000035237.11	-0.034921239	MGI:98905	Usp4
ENSMUST00000035612.6	-0.034921239	MGI:2444228	Ccar2
ENSMUST00000040455.4	-0.034921239	MGI:2442345	Hif1an
ENSMUST00000057438.6	-0.034921239	MGI:1917925	Vcpip1
ENSMUST00000068519.6	-0.034921239	MGI:2444661	Susd6
ENSMUST00000076219.5	-0.034921239	MGI:106253	Pum3
ENSMUST00000084731.4	-0.034921239	MGI:2152414	Ipo7
ENSMUST00000093923.8	-0.034921239	MGI:102685	Cdc27
ENSMUST00000097275.8	-0.034921239	MGI:97599	Prkce
ENSMUST00000098534.8	-0.034921239	MGI:1916996	Znhit6
ENSMUST00000102636.3	-0.034921239	MGI:1915300	Akirin1
ENSMUST00000102869.7	-0.034921239	MGI:2144013	Xpo1
ENSMUST00000103092.8	-0.034921239	MGI:97748	Ctsa
ENSMUST00000165007.8	-0.034921239	MGI:2651568	Klhdc3
ENSMUST00000234107.1	-0.034921239	MGI:1929211	Rnf138
ENSMUST00000036493.7	-0.033960719	MGI:88105	Atp1a1

ENSMUST00000029881.9	-0.033431882	MGI:1276107	Mmp16
ENSMUST00000030675.7	-0.033431882	MGI:1913657	Mrps15
ENSMUST00000061789.13	-0.033431882	MGI:2443880	Nyap1
ENSMUST00000068664.6	-0.033431882	MGI:1097159	Neo1
ENSMUST00000074541.5	-0.033431882	MGI:2141450	Jazf1
ENSMUST00000098143.10	-0.033431882	MGI:1921414	Nfx1
ENSMUST00000147535.7	-0.033111749	MGI:96759	Ldha
ENSMUST00000025053.9	-0.032529158	MGI:1927238	Mllt1
ENSMUST00000027498.13	-0.032529158	MGI:1891699	Stk25
ENSMUST00000040010.9	-0.032529158	MGI:1099460	Rbm3
ENSMUST00000087195.8	-0.032529158	MGI:1916377	Ociad2
ENSMUST00000109787.7	-0.032529158	MGI:106374	Zmiz2
ENSMUST00000111234.9	-0.032529158	MGI:1352474	Tnfrsf19
ENSMUST00000215836.1	-0.032529158	MGI:106387	Arfgef3
ENSMUST00000015891.5	-0.031923498	MGI:891965	Vps45
ENSMUST00000160884.8	-0.031923498	MGI:109239	Kcnab2
ENSMUST00000025474.13	-0.031845448	MGI:104561	Napg
ENSMUST00000007251.13	-0.031488985	MGI:99476	Abhd16a
ENSMUST00000051484.4	-0.031488985	MGI:1922875	Mageh1
ENSMUST00000102944.10	-0.031488985	MGI:99946	Creb3
ENSMUST00000106370.9	-0.030535419	MGI:1921569	Mettl23
ENSMUST00000197748.4	-0.030395719	MGI:2159711	Usp33
ENSMUST00000106650.8	-0.025249889	MGI:1917158	Rab3b
ENSMUST00000103118.3	-0.024689961	MGI:1343101	Kat2a
ENSMUST00000033156.4	-0.023533258	MGI:1891689	Dctn5
ENSMUST00000166411.7	-0.023423841	MGI:98423	Ssb
ENSMUST00000233741.1	-0.022811105	MGI:1913846	Gtf3a
ENSMUST00000021523.6	-0.02204231	MGI:106207	Mnat1
ENSMUST00000046212.1	-0.02204231	MGI:106013	Slc16a1
ENSMUST00000047404.6	-0.02204231	MGI:2135610	Dync1li1
ENSMUST00000080001.8	-0.02204231	MGI:1913405	Ufc1
ENSMUST00000159916.4	-0.02204231	MGI:1916267	Prrt2
ENSMUST00000169825.7	-0.02129575	MGI:105980	Cntn1
ENSMUST00000237341.1	-0.021064967	MGI:2385079	Ndufs8
ENSMUST00000025684.3	-0.021049135	MGI:1341878	Ehd1
ENSMUST00000044369.12	-0.021049135	MGI:1914864	Atp6v1h
ENSMUST00000077938.9	-0.021049135	MGI:1919877	Haghl
ENSMUST00000046206.4	-0.020263123	MGI:2385066	Rprd1a
ENSMUST00000003319.5	-0.019716564	MGI:1351656	Abcf3

ENSMUST00000025695.9	-0.019716564	MGI:2388480	Ppp2r5b
ENSMUST00000031423.9	-0.019716564	MGI:88110	Atp2a2
ENSMUST00000034529.13	-0.019716564	MGI:1922967	Cul5
ENSMUST00000035608.9	-0.019716564	MGI:1355331	Olig2
ENSMUST00000067120.13	-0.019716564	MGI:87888	Chrna4
ENSMUST00000070112.5	-0.019716564	MGI:1915625	Ndufa8
ENSMUST00000086046.9	-0.019716564	MGI:1278343	Nipsnap2
ENSMUST00000102927.9	-0.019716564	MGI:1338068	Pdpk1
ENSMUST00000174697.1	-0.019716564	MGI:2444389	Tmem229b
ENSMUST00000023291.5	-0.017834773	MGI:1926555	Mapk8ip2
ENSMUST00000026479.10	-0.017834773	MGI:107733	Dctn2
ENSMUST00000036509.13	-0.017834773	MGI:1920995	Ubac1
ENSMUST00000057551.13	-0.017834773	MGI:108402	Slbp
ENSMUST00000065534.9	-0.017834773	MGI:1346331	Def8
ENSMUST00000031565.14	-0.017031882	MGI:1352745	Fscn1
ENSMUST00000020496.13	-0.014976084	MGI:891999	Adarb1
ENSMUST00000037942.10	-0.014976084	MGI:1918142	Ttll7
ENSMUST00000039571.13	-0.014976084	MGI:1913671	2410004B18Rik
ENSMUST00000042148.5	-0.014976084	MGI:1913480	Mrps28
ENSMUST00000047226.9	-0.014976084	MGI:1921392	Lonp1
ENSMUST00000092623.4	-0.014976084	MGI:1920030	Rspo3
ENSMUST00000171737.2	-0.014976084	MGI:97402	Odc1
ENSMUST00000037615.6	-0.012384385	MGI:2441982	Aldh5a1
ENSMUST00000150576.7	-0.012384385	MGI:99687	Rpl29
ENSMUST00000006035.12	-0.012167678	MGI:1913616	Ergic3
ENSMUST00000151287.7	-0.012167678	MGI:98535	Tcp1
ENSMUST00000024727.9	-0.011508712	MGI:1918952	Cdc5l
ENSMUST00000004868.5	-0.0101131	MGI:1916686	Mtfp1
ENSMUST00000013737.12	-0.0101131	MGI:2385112	Ndufs2
ENSMUST00000021692.8	-0.0101131	MGI:99150	Yy1
ENSMUST00000025786.8	-0.0101131	MGI:1277113	Pacs1
ENSMUST00000026126.9	-0.0101131	MGI:1917164	Ints4
ENSMUST00000027139.14	-0.0101131	MGI:1920924	Wdr75
ENSMUST00000027517.13	-0.0101131	MGI:2138334	Dgkd
ENSMUST00000027521.14	-0.0101131	MGI:2653690	Agap1
ENSMUST00000027979.13	-0.0101131	MGI:1341908	Uhmk1
ENSMUST00000031977.11	-0.0101131	MGI:1917173	Agk
ENSMUST00000034079.13	-0.0101131	MGI:2444491	Heatr3
ENSMUST00000037814.7	-0.0101131	MGI:2447164	Cmtm5

ENSMUST00000038498.9	-0.0101131	MGI:1914634	Bag4
ENSMUST00000047629.6	-0.0101131	MGI:1096573	Utp4
ENSMUST00000049474.8	-0.0101131	MGI:1923041	Fbxw8
ENSMUST00000053218.6	-0.0101131	MGI:1920347	Dact2
ENSMUST00000064285.14	-0.0101131	MGI:2179717	Glcci1
ENSMUST00000066497.11	-0.0101131	MGI:1929704	Zfp24
ENSMUST00000090046.11	-0.0101131	MGI:2441844	Tmem87a
ENSMUST00000094917.9	-0.0101131	MGI:2138365	Tmem237
ENSMUST00000102748.10	-0.0101131	MGI:1931053	Marchf7
ENSMUST00000103101.10	-0.0101131	MGI:1926178	Pigt
ENSMUST00000113239.9	-0.0101131	MGI:1913277	Crbn
ENSMUST00000192509.5	-0.0101131	MGI:1349394	Map4k4
ENSMUST00000200607.4	-0.0101131	MGI:1344349	Rnf216
ENSMUST00000208222.1	-0.0101131	MGI:1922997	Sesn3
ENSMUST00000232732.1	-0.0101131	MGI:96957	Mea1
ENSMUST00000034567.3	-0.009900215	MGI:2385311	Dlat
ENSMUST00000022062.7	-0.008868854	MGI:1914195	Sdha
ENSMUST00000047973.3	-0.007410404	MGI:1922004	Dhcr24
ENSMUST00000223160.1	-0.007410404	MGI:2137354	Trim9
ENSMUST00000006828.8	-0.007239093	MGI:88046	Aplp1
ENSMUST00000023040.8	-0.006785697	MGI:1342248	Slc25a17
ENSMUST00000031190.4	-0.006533156	MGI:1100515	Dr1
ENSMUST00000040275.8	-0.006533156	MGI:1924427	Sobp
ENSMUST00000041901.6	-0.006533156	MGI:1929293	Cib2
ENSMUST00000033008.9	-0.006234104	MGI:1347005	Psma1
ENSMUST00000229641.1	-0.005317769	MGI:98857	Tsta3
ENSMUST00000071718.11	-0.005207019	MGI:894320	Prdx6
ENSMUST00000004057.8	-0.002567296	MGI:1917436	Fam162a
ENSMUST00000015049.4	-0.002138956	MGI:1351618	Dnajb9
ENSMUST00000028917.6	-0.002138956	MGI:1913294	Dtd1
ENSMUST00000022704.8	-0.00193205	MGI:1309517	Itm2b
ENSMUST00000002445.9	-0.001710799	MGI:1919060	Ranbp3
ENSMUST00000031334.14	-0.001710799	MGI:2384802	Eif2b1
ENSMUST00000055117.8	-0.001710799	MGI:2385045	Lemd2
ENSMUST00000064637.10	-0.001710799	MGI:109483	Rnf103
ENSMUST00000071926.4	-0.001710799	MGI:1917328	Nol7
ENSMUST00000079213.5	-0.001710799	MGI:1916962	Prpf38a
ENSMUST00000114976.8	-0.001710799	MGI:1914228	Luc7l
ENSMUST00000000275.8	0	MGI:95749	Glra3

ENSMUST00000000384.7	0	MGI:1336209	Trappc10
ENSMUST00000000901.12	0	MGI:1888986	Dlg3
ENSMUST00000001415.8	0	MGI:108404	Apbb3
ENSMUST00000002825.5	0	MGI:1353499	Baz1b
ENSMUST00000002837.10	0	MGI:1921586	Tmed5
ENSMUST00000003434.13	0	MGI:1933831	Btbd2
ENSMUST00000003843.15	0	MGI:104677	Man1a
ENSMUST00000005616.15	0	MGI:108022	Pkn1
ENSMUST00000010421.6	0	MGI:1919753	2610507B11Rik
ENSMUST00000011302.8	0	MGI:1919558	Brf1
ENSMUST00000012580.12	0	MGI:2153839	Hps3
ENSMUST00000012679.14	0	MGI:1196412	Tnpo3
ENSMUST00000013130.14	0	MGI:2151064	Strn3
ENSMUST00000013931.11	0	MGI:2148922	Ehmt2
ENSMUST00000015449.5	0	MGI:1917347	Sash1
ENSMUST00000015605.14	0	MGI:105121	Atf6b
ENSMUST00000016771.12	0	MGI:107717	Myh9
ENSMUST00000016907.7	0	MGI:1890616	Scube1
ENSMUST00000017783.12	0	MGI:2442920	Rab11fip4
ENSMUST00000018304.6	0	MGI:1100846	Med1
ENSMUST00000018716.9	0	MGI:1925496	Phf23
ENSMUST00000018909.3	0	MGI:1346074	Fxr2
ENSMUST00000019220.15	0	MGI:2142346	Strn4
ENSMUST00000019791.13	0	MGI:1203727	Sema6a
ENSMUST00000020112.6	0	MGI:2442888	Uhrf1bp1l
ENSMUST00000020159.14	0	MGI:1917458	Med23
ENSMUST00000020251.9	0	MGI:3643902	Gnptab
ENSMUST00000020329.12	0	MGI:95294	Egfr
ENSMUST00000020339.9	0	MGI:1913937	Tbc1d15
ENSMUST00000020420.8	0	MGI:107734	Ap3d1
ENSMUST00000020463.13	0	MGI:1926081	Ncln
ENSMUST00000020513.9	0	MGI:2442119	Papolg
ENSMUST00000020552.6	0	MGI:106618	Tpgs1
ENSMUST00000020756.8	0	MGI:1918951	Pnpt1
ENSMUST00000020904.7	0	MGI:107926	Rock2
ENSMUST00000020909.3	0	MGI:108017	Laptm4a
ENSMUST00000021209.7	0	MGI:1100497	Doc2b
ENSMUST00000021335.6	0	MGI:1924233	Scfd1
ENSMUST00000021900.13	0	MGI:109244	Sema4d

ENSMUST00000022153.7	0	MGI:1916177	Ptcd2
ENSMUST00000022666.8	0	MGI:2136335	Klhl1
ENSMUST00000022707.6	0	MGI:1194894	Gpc5
ENSMUST00000022793.14	0	MGI:1891824	Acin1
ENSMUST00000022890.9	0	MGI:1353623	Rnf19a
ENSMUST00000022947.6	0	MGI:109613	Matn2
ENSMUST00000023211.15	0	MGI:1913331	Sharpin
ENSMUST00000023561.7	0	MGI:1923076	Senp2
ENSMUST00000023689.10	0	MGI:107658	Ifnar1
ENSMUST00000024860.8	0	MGI:1928900	Ehd3
ENSMUST00000025186.15	0	MGI:95909	Slc39a7
ENSMUST00000025377.13	0	MGI:1920180	Ppp2r2b
ENSMUST00000025729.11	0	MGI:1921743	Tnks2
ENSMUST00000025993.9	0	MGI:1315203	Slit1
ENSMUST00000026220.6	0	MGI:1353437	Scd3
ENSMUST00000026886.7	0	MGI:1925751	Itih5
ENSMUST00000026897.13	0	MGI:1351867	Slco3a1
ENSMUST00000026972.7	0	MGI:2136853	Fam20c
ENSMUST00000027580.10	0	MGI:1921400	Slc35f5
ENSMUST00000027725.10	0	MGI:2385619	Klhl12
ENSMUST00000027752.14	0	MGI:99914	Lamc1
ENSMUST00000028667.9	0	MGI:1278339	Dgkz
ENSMUST00000028767.8	0	MGI:1309480	Rtf1
ENSMUST00000028944.3	0	MGI:1915988	Acss1
ENSMUST00000029141.5	0	MGI:1341867	Mmp24
ENSMUST00000029480.8	0	MGI:1914171	Prpf38b
ENSMUST00000029667.12	0	MGI:1924937	Kcnq5
ENSMUST00000029740.13	0	MGI:1915095	Rnf115
ENSMUST00000029891.11	0	MGI:1919348	Tmem68
ENSMUST00000030471.8	0	MGI:2441984	Lrrc41
ENSMUST00000030769.5	0	MGI:109555	Psmc2
ENSMUST00000030884.9	0	MGI:2442230	Mfn2
ENSMUST00000030917.5	0	MGI:98310	Ski
ENSMUST00000030922.14	0	MGI:97602	Prkcz
ENSMUST00000031519.13	0	MGI:1352750	Cabp1
ENSMUST00000031554.8	0	MGI:1921794	Tmem168
ENSMUST00000031859.13	0	MGI:109275	Trim24
ENSMUST00000032105.10	0	MGI:107935	Lrig1
ENSMUST00000032151.2	0	MGI:1914142	Eif4e3

ENSMUST00000032719.14	0	MGI:2183703	Nav3
ENSMUST00000032892.6	0	MGI:2451073	Xylt1
ENSMUST00000032978.7	0	MGI:1201407	Sh2b1
ENSMUST00000033058.13	0	MGI:1921831	Sbf2
ENSMUST00000033158.5	0	MGI:107301	Ubfd1
ENSMUST00000033163.7	0	MGI:1914862	Mettl9
ENSMUST00000033341.11	0	MGI:2651573	Tub
ENSMUST00000034074.7	0	MGI:2136825	N4bp1
ENSMUST00000034085.7	0	MGI:1349766	Brd7
ENSMUST00000034141.15	0	MGI:1914137	Lonp2
ENSMUST00000034225.6	0	MGI:2183572	Cntnap4
ENSMUST00000034296.14	0	MGI:1098772	Pik3r2
ENSMUST00000034411.9	0	MGI:2182585	Med17
ENSMUST00000034453.5	0	MGI:87902	Acta1
ENSMUST00000034469.6	0	MGI:1932286	Egln1
ENSMUST00000034510.8	0	MGI:1926483	Nectin1
ENSMUST00000034592.9	0	MGI:2150309	Dscaml1
ENSMUST00000034960.13	0	MGI:1921638	Dpp8
ENSMUST00000035010.9	0	MGI:1915542	Stt3b
ENSMUST00000035177.14	0	MGI:2137204	Mrpl3
ENSMUST00000035214.10	0	MGI:1351633	Ip6k1
ENSMUST00000035462.6	0	MGI:2442948	Dsel
ENSMUST00000036113.3	0	MGI:2652878	Tbc1d16
ENSMUST00000036161.11	0	MGI:2448506	Pi4ka
ENSMUST00000037012.2	0	MGI:96661	Kcna4
ENSMUST00000037472.12	0	MGI:2142195	Leng8
ENSMUST00000037649.5	0	MGI:2445001	Rab3gap1
ENSMUST00000037788.5	0	MGI:2444430	Pomt2
ENSMUST00000038116.12	0	MGI:1914357	Ankrd12
ENSMUST00000038237.7	0	MGI:1351333	Thoc5
ENSMUST00000038574.6	0	MGI:2145374	Dhx29
ENSMUST00000038696.11	0	MGI:2387581	Ppp1r9b
ENSMUST00000038794.5	0	MGI:2443967	Dpp9
ENSMUST00000039061.14	0	MGI:2444585	Trappc11
ENSMUST00000040280.13	0	MGI:1914222	Slc25a23
ENSMUST00000040700.8	0	MGI:106321	Dock9
ENSMUST00000040992.7	0	MGI:2387995	Nek9
ENSMUST00000041415.4	0	MGI:1341723	Kcnh3
ENSMUST00000041683.8	0	MGI:2144157	Usp22

ENSMUST00000041723.14	0	MGI:2445110	Zdhhc17
ENSMUST00000041956.13	0	MGI:1918084	Spag9
ENSMUST00000042057.11	0	MGI:1890222	Midn
ENSMUST00000042365.8	0	MGI:1916812	Cdk13
ENSMUST00000042700.11	0	MGI:95864	Gstp2
ENSMUST00000043200.7	0	MGI:1917030	Smap2
ENSMUST00000043368.11	0	MGI:2445126	Tut4
ENSMUST00000043531.9	0	MGI:1922937	Ripor1
ENSMUST00000043599.6	0	MGI:1920336	Rps6ka5
ENSMUST00000043725.8	0	MGI:3643623	Cntnap5a
ENSMUST00000043938.7	0	MGI:1352756	Plcl2
ENSMUST00000044455.7	0	MGI:2137896	Zfp451
ENSMUST00000044532.10	0	MGI:2429763	Dock3
ENSMUST00000044616.9	0	MGI:1919906	Ints8
ENSMUST00000044624.7	0	MGI:3521816	Kcnk9
ENSMUST00000044858.15	0	MGI:98215	Rxrb
ENSMUST00000045540.3	0	MGI:2651588	Socs7
ENSMUST00000045713.3	0	MGI:3603030	Nacad
ENSMUST00000045847.14	0	MGI:109637	Erf
ENSMUST00000045903.7	0	MGI:1914345	Trak1
ENSMUST00000046521.13	0	MGI:1924828	Bcl9
ENSMUST00000046548.13	0	MGI:1891468	Lgr4
ENSMUST00000047037.14	0	MGI:2442413	Thoc2
ENSMUST00000047086.9	0	MGI:1919765	Wdr43
ENSMUST00000047232.13	0	MGI:2443065	Agps
ENSMUST00000047903.9	0	MGI:1924348	Colgalt1
ENSMUST00000048002.6	0	MGI:2652891	B4galnt4
ENSMUST00000048138.6	0	MGI:109581	S100a13
ENSMUST00000048418.13	0	MGI:2444611	Ipo8
ENSMUST00000048519.16	0	MGI:2661416	Snx13
ENSMUST00000048706.9	0	MGI:1354944	Orc3
ENSMUST00000048896.7	0	MGI:104648	Fbrs
ENSMUST00000048966.6	0	MGI:108399	Tcf20
ENSMUST00000049146.11	0	MGI:2686228	Ephx4
ENSMUST00000049206.5	0	MGI:1918954	Arhgef3
ENSMUST00000049956.4	0	MGI:3605040	Lrrc24
ENSMUST00000051139.12	0	MGI:2444993	Rsbn1
ENSMUST00000052179.7	0	MGI:2136758	Pcdhb20
ENSMUST00000052245.8	0	MGI:1921094	Ankrd45

ENSMUST00000052249.6	0	MGI:1919064	Mrps27
ENSMUST00000052550.12	0	MGI:2151136	Lrp1b
ENSMUST00000053491.8	0	MGI:101896	Pou3f1
ENSMUST00000053699.12	0	MGI:1917604	Secisbp2l
ENSMUST00000053764.6	0	MGI:1890077	Foxo1
ENSMUST00000054245.7	0	MGI:2685490	Tmem132e
ENSMUST00000055931.4	0	MGI:1922469	Dusp18
ENSMUST00000056919.8	0	MGI:88547	Csnk2a2
ENSMUST00000057028.14	0	MGI:1916947	Camsap3
ENSMUST00000057416.7	0	MGI:2443862	Tmem251
ENSMUST00000057795.11	0	MGI:1913489	Rsrc2
ENSMUST00000058150.7	0	MGI:96816	Lor
ENSMUST00000058470.15	0	MGI:98086	Polr2a
ENSMUST00000058856.8	0	MGI:2670997	Scd4
ENSMUST00000058860.13	0	MGI:99961	Usf2
ENSMUST00000060043.12	0	MGI:2442092	Wnk1
ENSMUST00000061772.10	0	MGI:2443205	Rbm15
ENSMUST00000062528.8	0	MGI:1346069	Cdh20
ENSMUST00000063761.7	0	MGI:2446526	Cpt1c
ENSMUST00000064091.11	0	MGI:1099792	Ptpn4
ENSMUST00000064174.11	0	MGI:2443629	Cemip
ENSMUST00000064444.7	0	MGI:2684896	Maneal
ENSMUST00000064795.5	0	MGI:95295	Egr1
ENSMUST00000065263.11	0	MGI:2384298	Sbno1
ENSMUST00000065330.7	0	MGI:1098670	Clk3
ENSMUST00000065630.7	0	MGI:1924841	Ddx10
ENSMUST00000065858.2	0	MGI:2444609	Nlgn3
ENSMUST00000066058.7	0	MGI:1347004	Mapkbp1
ENSMUST00000066958.10	0	MGI:1341873	Gfra4
ENSMUST00000067219.4	0	MGI:1890446	Dach2
ENSMUST00000068681.11	0	MGI:1858414	Ngef
ENSMUST00000073441.12	0	MGI:2682334	Smg7
ENSMUST00000073572.10	0	MGI:103025	Mtx1
ENSMUST00000074729.5	0	MGI:2444188	Dgkh
ENSMUST00000074840.11	0	MGI:1355326	Preb
ENSMUST00000076698.12	0	MGI:108081	Sypl
ENSMUST00000076810.11	0	MGI:2685385	Kalrn
ENSMUST00000077273.8	0	MGI:894663	Ext1
ENSMUST00000077972.10	0	MGI:2139014	Osbpl6

ENSMUST00000078308.12	0	MGI:2443881	Rasal2
ENSMUST00000079176.13	0	MGI:1351469	Plekhb1
ENSMUST00000079300.12	0	MGI:1918053	Arhgef26
ENSMUST00000079817.7	0	MGI:2385007	Stk24
ENSMUST00000080030.13	0	MGI:1316728	Gspt1
ENSMUST00000080036.2	0	MGI:96067	Htt
ENSMUST00000080123.1	0	MGI:1931052	Aldoart2
ENSMUST00000080437.12	0	MGI:1201681	Dennd5a
ENSMUST00000081635.12	0	MGI:1927450	St7
ENSMUST00000082034.12	0	MGI:1914321	Rps6ka6
ENSMUST00000082392.1	0	MGI:101787	mt-Nd1
ENSMUST00000082396.1	0	MGI:102500	mt-Nd2
ENSMUST00000082402.1	0	MGI:102504	mt-Co1
ENSMUST00000082405.1	0	MGI:102503	mt-Co2
ENSMUST00000082409.1	0	MGI:102502	mt-Co3
ENSMUST00000082418.1	0	MGI:102496	mt-Nd5
ENSMUST00000082419.1	0	MGI:102495	mt-Nd6
ENSMUST00000082421.1	0	MGI:102501	mt-Cytb
ENSMUST00000084299.5	0	MGI:1889364	Akirin2
ENSMUST00000084894.14	0	MGI:5804952	Gm45837
ENSMUST00000085678.7	0	MGI:2444629	Kctd3
ENSMUST00000086395.6	0	MGI:2686146	Gpr25
ENSMUST00000087200.3	0	MGI:95764	Gnl1
ENSMUST00000087867.5	0	MGI:2685620	Uprt
ENSMUST00000088174.3	0	MGI:1354958	Hs6st1
ENSMUST00000088516.9	0	MGI:1354953	Magi2
ENSMUST00000088737.10	0	MGI:87940	Grk2
ENSMUST00000088786.10	0	MGI:98419	Sri
ENSMUST00000089645.12	0	MGI:1928676	Htra2
ENSMUST00000090247.6	0	MGI:1927230	Trio
ENSMUST00000091039.4	0	MGI:1922008	Ralgps1
ENSMUST00000091497.10	0	MGI:1919850	Frmd4a
ENSMUST00000091694.9	0	MGI:2444879	Ripor2
ENSMUST00000093393.4	0	MGI:1918560	Tbc1d9
ENSMUST00000093468.11	0	MGI:1918215	Psd3
ENSMUST00000093820.9	0	MGI:2442675	Rfx7
ENSMUST00000094933.4	0	MGI:1919936	Usp24
ENSMUST00000095310.2	0	MGI:2443807	Osbpl8
ENSMUST00000096275.4	0	MGI:3528396	Iqsec2

ENSMUST00000096987.6	0	MGI:1195461	Septin5
ENSMUST00000097561.8	0	MGI:88549	Csrp1
ENSMUST00000097650.9	0	MGI:1342770	Lrrfip1
ENSMUST00000098098.8	0	MGI:1920719	Rnf38
ENSMUST00000099337.4	0	MGI:1890127	Plxnc1
ENSMUST00000099931.10	0	MGI:2155278	Sesn1
ENSMUST00000100309.2	0	MGI:2146159	Alg10b
ENSMUST00000100327.9	0	MGI:1921463	Rbm26
ENSMUST00000100337.9	0	MGI:1353586	Lmo7
ENSMUST00000101118.3	0	MGI:1929059	Rybp
ENSMUST00000101141.8	0	MGI:107194	Shroom2
ENSMUST00000101638.3	0	MGI:1197517	Limk2
ENSMUST00000102592.9	0	MGI:98759	Tjp1
ENSMUST00000102637.7	0	MGI:88016	Ampd2
ENSMUST00000102689.9	0	MGI:1277166	Sp3
ENSMUST00000102694.3	0	MGI:1277114	Ptgfrn
ENSMUST00000102838.9	0	MGI:98388	Sptbn1
ENSMUST00000102872.10	0	MGI:104580	Rapgef1
ENSMUST00000105584.9	0	MGI:2153589	Acap3
ENSMUST00000105902.7	0	MGI:2671987	Shank2
ENSMUST00000106108.8	0	MGI:1915035	Zmym4
ENSMUST00000106226.8	0	MGI:107913	Tial1
ENSMUST00000106550.10	0	MGI:1913606	Knop1
ENSMUST00000106600.8	0	MGI:107427	Elavl4
ENSMUST00000106625.9	0	MGI:1923930	5330417C22Rik
ENSMUST00000106939.8	0	MGI:1346023	Tlk2
ENSMUST00000107170.2	0	MGI:1934229	Setdb1
ENSMUST00000107359.8	0	MGI:1918264	Megf9
ENSMUST00000107478.8	0	MGI:3588195	Adamts17
ENSMUST00000107479.2	0	MGI:3611446	Rapgefl1
ENSMUST00000107865.8	0	MGI:2441680	Tmem8b
ENSMUST00000108153.8	0	MGI:1329041	Rngtt
ENSMUST00000108285.8	0	MGI:1915349	Fam92a
ENSMUST00000108588.8	0	MGI:1277959	Dlg4
ENSMUST00000108661.7	0	MGI:1344395	Chd3
ENSMUST00000108682.8	0	MGI:1202388	Gas7
ENSMUST00000109158.8	0	MGI:2443183	Mia3
ENSMUST00000109645.8	0	MGI:2384826	Vstm2a
ENSMUST00000109691.3	0	MGI:2444365	Rapgef5

ENSMUST00000109923.8	0	MGI:1931838	Dbn1
ENSMUST00000110097.8	0	MGI:1922857	Wnk2
ENSMUST00000110746.1	0	MGI:5663654	Gm43517
ENSMUST00000111091.7	0	MGI:1917353	Znhit1
ENSMUST00000111346.5	0	MGI:2443235	Rimbp2
ENSMUST00000111690.7	0	MGI:105100	Ctnnd1
ENSMUST00000112096.8	0	MGI:1859162	Rnf10
ENSMUST00000112098.10	0	MGI:1923998	Pbrm1
ENSMUST00000112498.2	0	MGI:1354756	Crim1
ENSMUST00000112512.7	0	MGI:96958	Golga3
ENSMUST00000112588.8	0	MGI:99781	Kdm5c
ENSMUST00000113533.2	0	MGI:1913656	Sac3d1
ENSMUST00000113878.7	0	MGI:2442233	Arhgef9
ENSMUST00000114112.3	0	MGI:1339963	St3gal5
ENSMUST00000114167.8	0	MGI:3036242	Camsap1
ENSMUST00000114881.8	0	MGI:1914347	Rps10
ENSMUST00000115366.2	0	MGI:101924	Slc12a2
ENSMUST00000115797.8	0	MGI:1926129	Arid1b
ENSMUST00000116238.8	0	MGI:96103	Hk1
ENSMUST00000116388.3	0	MGI:106340	Rcor1
ENSMUST00000116574.9	0	MGI:1861755	Nsmf
ENSMUST00000117085.1	0	MGI:1917428	Abhd17c
ENSMUST00000117245.1	0	MGI:2138319	Trp53bp2
ENSMUST00000117661.8	0	MGI:2151156	Stim2
ENSMUST00000118444.2	0	MGI:3584516	Lrp3
ENSMUST00000119339.7	0	MGI:2443713	Ythdc1
ENSMUST00000120194.1	0	MGI:106351	Fam104a
ENSMUST00000121212.8	0	MGI:1098669	Clk2
ENSMUST00000122103.8	0	MGI:2444431	Rab11fip3
ENSMUST00000128187.7	0	MGI:1355299	Hnrnpdl
ENSMUST00000129241.2	0	MGI:98386	Sptan1
ENSMUST00000129423.7	0	MGI:1889007	Ttyh1
ENSMUST00000130379.8	0	MGI:2147134	Fbxo11
ENSMUST00000130451.1	0	MGI:1914647	Erp29
ENSMUST00000131070.1	0	MGI:96412	Ide
ENSMUST00000138410.7	0	MGI:1921303	Grip1
ENSMUST00000139517.8	0	MGI:1916329	Sgsm3
ENSMUST00000139666.7	0	MGI:1314653	Afdn
ENSMUST00000149154.7	0	MGI:2685563	Pcdh19

ENSMUST00000154078.2	0	MGI:2685104	Akap5
ENSMUST00000155487.7	0	MGI:1913881	Mfsd14b
ENSMUST00000159038.7	0	MGI:2442786	Mfsd4a
ENSMUST00000159081.7	0	MGI:109474	Commd1
ENSMUST00000159124.7	0	MGI:96567	Impdh1
ENSMUST00000159295.7	0	MGI:106341	Atp5o
ENSMUST00000159295.7	0	MGI:106341	Atp5o
ENSMUST00000159295.7	0	MGI:106341	Atp5o
ENSMUST00000159295.7	0	MGI:106341	Atp5o
ENSMUST00000159952.7	0	MGI:5547772	Gm28036
ENSMUST00000160378.2	0	MGI:109182	Acat3
ENSMUST00000162144.1	0	MGI:1918161	Phyhipl
ENSMUST00000163065.7	0	MGI:102810	Lmo3
ENSMUST00000163370.7	0	MGI:103306	Tiam1
ENSMUST00000164107.2	0	MGI:88141	Bcr
ENSMUST00000164950.10	0	MGI:107238	Tmem191c
ENSMUST00000165937.7	0	MGI:3611233	Tox2
ENSMUST00000166429.8	0	MGI:2443679	Sipa1l1
ENSMUST00000167926.7	0	MGI:1917275	Acot7
ENSMUST00000168076.1	0	MGI:2147269	Slc39a3
ENSMUST00000168386.8	0	MGI:3605626	Prr36
ENSMUST00000168579.7	0	MGI:1933438	Slc16a3
ENSMUST00000169035.7	0	MGI:2442640	Cpeb2
ENSMUST00000169256.4	0	MGI:2669829	D630045J12Rik
ENSMUST00000169423.8	0	MGI:2140230	Camta1
ENSMUST00000169863.8	0	MGI:2444268	Fam169a
ENSMUST00000173875.8	0	MGI:1344414	Sra1
ENSMUST00000174050.7	0	MGI:109547	Dnm2
ENSMUST00000174641.7	0	MGI:2450166	Arhgap32
ENSMUST00000175903.8	0	MGI:103264	Arhgef2
ENSMUST00000176233.1	0	MGI:5313142	Gm20695
ENSMUST00000178174.2	0	MGI:101895	Pou3f2
ENSMUST00000179234.1	0	MGI:2447811	Aldoart1
ENSMUST00000179393.7	0	MGI:2179326	Oxr1
ENSMUST00000180037.7	0	MGI:1276108	Birc6
ENSMUST00000182884.7	0	MGI:88026	Ank3
ENSMUST00000183156.7	0	MGI:1924781	Anks1b
ENSMUST00000183850.7	0	MGI:2153182	Kcnn2
ENSMUST00000183984.7	0	MGI:1338822	Celf2

ENSMUST00000184183.1	0	MGI:4937914	Gm17087
ENSMUST00000186207.6	0	MGI:3039600	Fbxl19
ENSMUST00000186375.7	0	MGI:1922013	Naa60
ENSMUST00000186548.6	0	MGI:2446229	Tet3
ENSMUST00000189137.6	0	MGI:109528	Trpc1
ENSMUST00000190285.1	0	MGI:103013	Cacna1c
ENSMUST00000191138.6	0	MGI:1338076	Hivep2
ENSMUST00000191677.5	0	MGI:109252	Sema4c
ENSMUST00000191758.5	0	MGI:2443399	Tmem131l
ENSMUST00000192093.5	0	MGI:1916672	Pex5l
ENSMUST00000194563.5	0	MGI:1927110	Tmem131
ENSMUST00000195708.1	0	MGI:2659071	Rapgef2
ENSMUST00000195823.1	0	MGI:1935218	Pcdhga6
ENSMUST00000198499.4	0	MGI:1923811	Snx7
ENSMUST00000203482.1	0	MGI:1917977	Rasgef1a
ENSMUST00000208538.1	0	MGI:2673998	Arhgap33
ENSMUST00000212889.1	0	MGI:1347098	Slc27a1
ENSMUST00000213990.1	0	MGI:101848	Gnb5
ENSMUST00000217647.1	0	MGI:1925976	Scaper
ENSMUST00000218823.1	0	MGI:95815	Grik2
ENSMUST00000219443.1	0	MGI:1332637	Arhgap5
ENSMUST00000220302.1	0	MGI:104296	Nova2
ENSMUST00000220816.1	0	MGI:1261845	Esyt2
ENSMUST00000221240.1	0	MGI:1919508	Kcnk10
ENSMUST00000224905.1	0	MGI:1921086	Slc35b2
ENSMUST00000226414.1	0	MGI:1918040	Ubr5
ENSMUST00000227496.1	0	MGI:109503	Cdh12
ENSMUST00000229741.1	0	MGI:1926224	Rbfox1
ENSMUST00000232239.1	0	MGI:1890564	Rcan1
ENSMUST00000233173.1	0	MGI:1346065	Dlgap1
ENSMUST00000233197.1	0	MGI:1914239	Kctd20
ENSMUST00000234081.1	0	MGI:105094	Adcyap1
ENSMUST00000236045.1	0	MGI:1914049	Ube2w
ENSMUST00000238331.1	0	MGI:1350923	Sh3d19
ENSMUST00000238821.1	0	MGI:1196356	Iqsec1
ENSMUST00000238951.1	0	MGI:2449923	Egfl7
ENSMUST00000239069.1	0	MGI:109155	Kcnab1
ENSMUST00000000153.8	0	MGI:95767	Gna12
ENSMUST00000000304.13	0	MGI:1916942	Hddc2

ENSMUST00000001347.6	0	MGI:1338755	Rnd2
ENSMUST00000001920.12	0	MGI:1919598	Aif1l
ENSMUST00000002100.7	0	MGI:1918937	Tmem25
ENSMUST00000002127.13	0	MGI:1328357	Unc119
ENSMUST00000002152.12	0	MGI:2181667	Bbc3
ENSMUST00000002397.6	0	MGI:1917903	Gmpr2
ENSMUST00000002532.8	0	MGI:1915153	Rgs19
ENSMUST00000002625.14	0	MGI:98904	Uck1
ENSMUST00000003152.13	0	MGI:1341870	Stk11
ENSMUST00000004326.3	0	MGI:107683	Plxna3
ENSMUST00000004381.13	0	MGI:1315211	Lpcat3
ENSMUST00000004681.13	0	MGI:1354723	Pnpla6
ENSMUST00000004684.12	0	MGI:2142567	Arhgef18
ENSMUST00000004684.12	0	MGI:2142567	Arhgef18
ENSMUST00000004684.12	0	MGI:2142567	Arhgef18
ENSMUST00000004684.12	0	MGI:2142567	Arhgef18
ENSMUST00000004729.4	0	MGI:106098	Etfb
ENSMUST00000004968.10	0	MGI:1347008	Plod3
ENSMUST00000005815.6	0	MGI:96677	Kit
ENSMUST00000006217.9	0	MGI:1343161	Snf8
ENSMUST00000006611.8	0	MGI:102690	Srm
ENSMUST00000006632.7	0	MGI:1917855	Zdhhc24
ENSMUST00000007255.12	0	MGI:1859016	Ddah2
ENSMUST00000008004.9	0	MGI:2136689	Ddx49
ENSMUST00000010795.4	0	MGI:3605455	Acaa1b
ENSMUST00000012028.13	0	MGI:1929253	Gltp
ENSMUST00000013667.2	0	MGI:1924210	Bcas1
ENSMUST00000013773.11	0	MGI:1916969	Cad
ENSMUST00000014981.7	0	MGI:1921606	4931428F04Rik
ENSMUST00000015017.7	0	MGI:98444	Surf2
ENSMUST00000015456.9	0	MGI:107776	Gadd45b
ENSMUST00000015894.11	0	MGI:2385110	Aph1a
ENSMUST00000016072.11	0	MGI:1932395	Rrbp1
ENSMUST00000016088.8	0	MGI:1933384	Castor2
ENSMUST00000016631.13	0	MGI:1914783	Ppfibp1
ENSMUST00000017622.11	0	MGI:1923264	Zc3h18
ENSMUST00000018337.8	0	MGI:2384876	Cdc73
ENSMUST00000018993.6	0	MGI:2388637	Wwc1
ENSMUST00000019962.14	0	MGI:1859568	Cd164

ENSMUST00000020004.7	0	MGI:1913653	Asf1a
ENSMUST00000020171.11	0	MGI:95537	Ccn2
ENSMUST00000020248.15	0	MGI:1914532	Washc3
ENSMUST00000020403.6	0	MGI:1202907	Csrp2
ENSMUST00000020461.14	0	MGI:109591	Nfic
ENSMUST00000020546.2	0	MGI:1316731	Stc2
ENSMUST00000020719.6	0	MGI:1915112	2310033P09Rik
ENSMUST00000020957.12	0	MGI:2144929	Adi1
ENSMUST00000020971.13	0	MGI:1344401	Rnf144a
ENSMUST00000021077.3	0	MGI:1349482	Slc9a3r1
ENSMUST00000021135.4	0	MGI:1914124	Ncbp3
ENSMUST00000021620.12	0	MGI:1915399	Otub2
ENSMUST00000021669.14	0	MGI:1920986	Fcf1
ENSMUST00000021920.7	0	MGI:1099431	Sptlc1
ENSMUST00000021937.11	0	MGI:1349417	Zfp346
ENSMUST00000021942.7	0	MGI:1913744	Prelid1
ENSMUST00000022499.12	0	MGI:1914403	Rnaseh2b
ENSMUST00000022665.3	0	MGI:2180557	Rhobtb2
ENSMUST00000022693.8	0	MGI:88176	Bmp1
ENSMUST00000022865.16	0	MGI:1914404	Mtdh
ENSMUST00000023007.6	0	MGI:1341110	Adcy8
ENSMUST00000023213.7	0	MGI:1930628	Hgh1
ENSMUST00000024260.13	0	MGI:1890471	Pcbp4
ENSMUST00000024572.9	0	MGI:3630308	Rsph3b
ENSMUST00000024839.5	0	MGI:104754	Sik1
ENSMUST00000024954.10	0	MGI:109169	Epas1
ENSMUST00000024983.11	0	MGI:2146906	Ift140
ENSMUST00000025003.9	0	MGI:98370	Sox8
ENSMUST00000025081.12	0	MGI:1344313	Zeb1
ENSMUST00000025363.6	0	MGI:96070	Hbegf
ENSMUST00000025420.13	0	MGI:97806	Ptpn2
ENSMUST00000025835.5	0	MGI:1098296	Cpt1a
ENSMUST00000025924.3	0	MGI:2147611	Spindoc
ENSMUST00000026256.8	0	MGI:1915681	Fbxl15
ENSMUST00000026357.11	0	MGI:1891497	Jph3
ENSMUST00000026448.9	0	MGI:1919135	Faap100
ENSMUST00000026470.5	0	MGI:1277989	Shmt2
ENSMUST00000026828.6	0	MGI:1915491	Mcrip2
ENSMUST00000027279.11	0	MGI:1923258	Nabp1

ENSMUST00000027303.13	0	MGI:106572	Imp4
ENSMUST00000027396.14	0	MGI:1921354	Abcb6
ENSMUST00000027743.12	0	MGI:1926235	Stx6
ENSMUST00000028209.14	0	MGI:1914093	Dolpp1
ENSMUST00000028288.4	0	MGI:97363	Notch1
ENSMUST00000028522.9	0	MGI:96605	Itga6
ENSMUST00000028600.13	0	MGI:1314882	Hipk3
ENSMUST00000028728.5	0	MGI:1277977	Ubr1
ENSMUST00000028995.4	0	MGI:1914267	Fam210b
ENSMUST00000029441.3	0	MGI:1859546	Syt10
ENSMUST00000029673.9	0	MGI:106644	Efna3
ENSMUST00000029850.14	0	MGI:88527	Cryz
ENSMUST00000030025.9	0	MGI:1352457	Nr4a3
ENSMUST00000030090.3	0	MGI:96853	Alad
ENSMUST00000030202.13	0	MGI:1917770	Glipr2
ENSMUST00000030556.7	0	MGI:104673	Ptpn12
ENSMUST00000030669.7	0	MGI:102462	Slc9a1
ENSMUST00000030739.10	0	MGI:95401	Epb41
ENSMUST00000030775.11	0	MGI:3036258	Chd5
ENSMUST00000030901.8	0	MGI:1919207	Ints11
ENSMUST00000030903.11	0	MGI:1919214	Atad3a
ENSMUST00000031345.14	0	MGI:1915348	Rchy1
ENSMUST00000031399.12	0	MGI:97788	Psph
ENSMUST00000031490.10	0	MGI:1270126	Ulk1
ENSMUST00000031588.11	0	MGI:2140991	Usp30
ENSMUST00000032192.8	0	MGI:96623	Itpr1
ENSMUST00000032462.8	0	MGI:109125	Timp4
ENSMUST00000032566.2	0	MGI:1914619	Qpctl
ENSMUST00000032710.6	0	MGI:2156052	Slc17a6
ENSMUST00000033160.14	0	MGI:1921355	Gga2
ENSMUST00000033173.14	0	MGI:1349452	Polr3e
ENSMUST00000033380.6	0	MGI:97516	Cdk16
ENSMUST00000033930.4	0	MGI:2442191	Dusp4
ENSMUST00000033938.6	0	MGI:97740	Polb
ENSMUST00000034000.14	0	MGI:1277124	Asah1
ENSMUST00000034136.11	0	MGI:1915391	Gpt2
ENSMUST00000034249.7	0	MGI:107428	Cfap20
ENSMUST00000034522.7	0	MGI:1918816	Clmp
ENSMUST00000034697.7	0	MGI:1915932	Slc44a2

ENSMUST00000034749.15	0	MGI:1924136	Fam81a
ENSMUST00000034889.9	0	MGI:1298209	Hcn4
ENSMUST00000035007.9	0	MGI:2447165	Cmtm6
ENSMUST00000035099.8	0	MGI:1921748	Gorasp1
ENSMUST00000035201.12	0	MGI:1338037	Rbm6
ENSMUST00000035346.13	0	MGI:1918765	Nol4l
ENSMUST00000035484.10	0	MGI:2448759	Cdv3
ENSMUST00000035604.12	0	MGI:2449311	Gemin5
ENSMUST00000035776.9	0	MGI:1923173	Dnttip2
ENSMUST00000035812.13	0	MGI:1916987	Ttl
ENSMUST00000036649.7	0	MGI:104842	Coil
ENSMUST00000036880.7	0	MGI:2448549	Cdk10
ENSMUST00000037097.8	0	MGI:1925847	Fhod3
ENSMUST00000037246.6	0	MGI:1333783	Ccs
ENSMUST00000037418.6	0	MGI:1923480	Tmed8
ENSMUST00000037534.7	0	MGI:1917760	Rnf167
ENSMUST00000037708.9	0	MGI:2652846	Asic4
ENSMUST00000038332.8	0	MGI:2385277	Ctu1
ENSMUST00000038600.3	0	MGI:2153089	Mrps2
ENSMUST00000038709.13	0	MGI:2384959	Cybc1
ENSMUST00000038753.5	0	MGI:2442062	Sh3pxd2b
ENSMUST00000039071.2	0	MGI:2157946	Cacng5
ENSMUST00000039331.8	0	MGI:2681842	Igsf21
ENSMUST00000039388.2	0	MGI:1933155	Arl4d
ENSMUST00000039818.9	0	MGI:2443883	Aldh4a1
ENSMUST00000039840.14	0	MGI:2445171	Enpp6
ENSMUST00000040340.15	0	MGI:3505790	Fcho2
ENSMUST00000040616.8	0	MGI:2442265	Kctd7
ENSMUST00000040647.10	0	MGI:95515	Fgf1
ENSMUST00000040676.10	0	MGI:2444209	Ankrd54
ENSMUST00000040776.5	0	MGI:2443939	Cenpt
ENSMUST00000040967.8	0	MGI:1916724	Vps37b
ENSMUST00000041053.10	0	MGI:1098623	Acaa2
ENSMUST00000041407.6	0	MGI:1913292	Sostdc1
ENSMUST00000041627.13	0	MGI:2443847	Sdk2
ENSMUST00000041776.11	0	MGI:108408	Rgs8
ENSMUST00000042412.4	0	MGI:1341800	Hey1
ENSMUST00000042603.13	0	MGI:96570	Inhba
ENSMUST00000043214.7	0	MGI:97846	Rac2

ENSMUST00000043338.9	0	MGI:1917362	Sft2d2
ENSMUST00000043493.6	0	MGI:2385865	Ice1
ENSMUST00000043521.4	0	MGI:2447876	Sec22a
ENSMUST00000043975.10	0	MGI:1922910	Lin37
ENSMUST00000044081.8	0	MGI:2682295	Tent4a
ENSMUST00000044484.12	0	MGI:1095419	Kdm6a
ENSMUST00000044734.2	0	MGI:2674366	Rims4
ENSMUST00000044844.8	0	MGI:3604804	Mfsd12
ENSMUST00000045110.13	0	MGI:1925064	Dstyk
ENSMUST00000045454.8	0	MGI:1916334	Fam207a
ENSMUST00000045487.3	0	MGI:1916831	Rhou
ENSMUST00000045521.8	0	MGI:2672905	Dtx4
ENSMUST00000045693.7	0	MGI:108048	Smyd5
ENSMUST00000045970.7	0	MGI:1194891	Gpc1
ENSMUST00000046073.15	0	MGI:1354737	Kdm2b
ENSMUST00000046157.9	0	MGI:1919257	Fndc3b
ENSMUST00000046174.7	0	MGI:106925	Cldn11
ENSMUST00000046223.13	0	MGI:2386846	Abca8a
ENSMUST00000046633.9	0	MGI:2146232	AW549877
ENSMUST00000046941.7	0	MGI:1916117	Rnf122
ENSMUST00000047034.8	0	MGI:2147036	Ttbk1
ENSMUST00000047085.15	0	MGI:2449973	Tbc1d17
ENSMUST00000047203.8	0	MGI:1917544	Rnf126
ENSMUST00000047815.12	0	MGI:1924281	Slc9a8
ENSMUST00000047923.11	0	MGI:1916858	Sec24d
ENSMUST00000048982.10	0	MGI:1916034	Prickle1
ENSMUST00000049138.7	0	MGI:1924042	2410131K14Rik
ENSMUST00000049156.6	0	MGI:1921597	Meak7
ENSMUST00000050625.14	0	MGI:107555	Sema5b
ENSMUST00000051053.4	0	MGI:2444752	Ubtd2
ENSMUST00000052168.5	0	MGI:1918448	Otud1
ENSMUST00000052183.6	0	MGI:2156003	Snip1
ENSMUST00000052315.12	0	MGI:3846135	Gm44502
ENSMUST00000052346.9	0	MGI:102682	Llgl1
ENSMUST00000052691.8	0	MGI:1915162	1600012H06Rik
ENSMUST00000052712.5	0	MGI:2151797	Tgs1
ENSMUST00000053445.15	0	MGI:1923734	Kndc1
ENSMUST00000054230.11	0	MGI:1859609	Sfmbt1
ENSMUST00000054343.14	0	MGI:1914855	Akt1s1

ENSMUST00000054697.6	0	MGI:1925179	Yipf6
ENSMUST00000055241.12	0	MGI:1270153	Zfp106
ENSMUST00000057324.3	0	MGI:3603594	Flrt2
ENSMUST00000057631.11	0	MGI:2144695	Sgsm2
ENSMUST00000057944.11	0	MGI:109522	Ugt8a
ENSMUST00000058137.8	0	MGI:2442633	Rabl6
ENSMUST00000058444.9	0	MGI:2687042	Ppp1r37
ENSMUST00000059057.13	0	MGI:1924483	Fam118b
ENSMUST00000060061.10	0	MGI:1916161	Pygo2
ENSMUST00000060173.8	0	MGI:2443872	Samd10
ENSMUST00000060834.11	0	MGI:2142037	Alkbh6
ENSMUST00000061169.6	0	MGI:3617843	Gal3st3
ENSMUST00000061545.6	0	MGI:2387350	C1ql3
ENSMUST00000061833.5	0	MGI:2685030	Tlcd5
ENSMUST00000061891.10	0	MGI:1920997	Shld1
ENSMUST00000062831.15	0	MGI:95413	Ercc2
ENSMUST00000063117.9	0	MGI:1341859	Gstz1
ENSMUST00000063470.10	0	MGI:109559	Ptprr
ENSMUST00000064016.5	0	MGI:3584514	Gpr17
ENSMUST00000064190.12	0	MGI:103291	Rai1
ENSMUST00000064405.7	0	MGI:99612	Epha3
ENSMUST00000064473.12	0	MGI:2151118	Adarb2
ENSMUST00000064536.12	0	MGI:1096335	Adam17
ENSMUST00000066060.10	0	MGI:1929913	Bcl11b
ENSMUST00000066279.10	0	MGI:2138297	Sh3bp4
ENSMUST00000066345.14	0	MGI:1921624	Clec16a
ENSMUST00000067215.8	0	MGI:1913501	Arfgap3
ENSMUST00000067458.6	0	MGI:107556	Sema5a
ENSMUST00000070642.3	0	MGI:88373	Cebpb
ENSMUST00000070801.10	0	MGI:1914824	Alg13
ENSMUST00000070923.2	0	MGI:1925301	Them6
ENSMUST00000071263.6	0	MGI:1913364	Dnajc30
ENSMUST00000071792.6	0	MGI:2152337	1110038F14Rik
ENSMUST00000071858.4	0	MGI:1855689	Hpcal1
ENSMUST00000072093.12	0	MGI:2154238	Plxnb1
ENSMUST00000072383.13	0	MGI:1916017	Washc1
ENSMUST00000072910.5	0	MGI:3036284	Chadl
ENSMUST00000073570.11	0	MGI:1915641	Zfp414
ENSMUST00000073705.11	0	MGI:2384939	Fam222b

ENSMUST00000074002.11	0	MGI:1891295	Ube3b
ENSMUST00000074552.11	0	MGI:1289164	Ncaph2
ENSMUST00000074991.9	0	MGI:2445179	Tmem184b
ENSMUST00000075610.12	0	MGI:2685945	Pstk
ENSMUST00000075770.12	0	MGI:2685177	Megf10
ENSMUST00000076813.7	0	MGI:1914982	Iah1
ENSMUST00000076957.6	0	MGI:1338012	Zdhhc8
ENSMUST00000077078.11	0	MGI:1922078	Rnf185
ENSMUST00000077220.13	0	MGI:1306825	Gtpbp6
ENSMUST00000077548.11	0	MGI:1933137	Cttnbp2nl
ENSMUST00000078357.4	0	MGI:1098726	Emp2
ENSMUST00000078626.7	0	MGI:1341834	Trank1
ENSMUST00000078880.5	0	MGI:1913923	Sorcs3
ENSMUST00000079080.12	0	MGI:1346347	Mapk7
ENSMUST00000079618.10	0	MGI:109243	St8sia5
ENSMUST00000079724.8	0	MGI:1346319	Hax1
ENSMUST00000080553.8	0	MGI:1858496	Deaf1
ENSMUST00000081333.10	0	MGI:1096879	Fxn
ENSMUST00000081688.12	0	MGI:2152936	Srgap1
ENSMUST00000081872.12	0	MGI:2443902	Nell1
ENSMUST00000082104.6	0	MGI:2137383	Csmd1
ENSMUST00000084027.12	0	MGI:95522	Fgfr1
ENSMUST00000084129.8	0	MGI:1919140	Mad2l2
ENSMUST00000085840.10	0	MGI:2155779	Ttbk2
ENSMUST00000086083.10	0	MGI:1337080	Ncor2
ENSMUST00000086465.5	0	MGI:99401	Adora1
ENSMUST00000086829.10	0	MGI:1101760	Bicd1
ENSMUST00000086843.10	0	MGI:1929735	Asb1
ENSMUST00000087908.9	0	MGI:1923396	Stx16
ENSMUST00000089497.6	0	MGI:1923310	Isy1
ENSMUST00000090103.10	0	MGI:109620	Arvcf
ENSMUST00000092498.11	0	MGI:1261415	Sgpl1
ENSMUST00000094053.6	0	MGI:2385292	Tnrc6a
ENSMUST00000094077.4	0	MGI:2448492	Kdm6b
ENSMUST00000096250.4	0	MGI:1924294	Arid2
ENSMUST00000096299.8	0	MGI:96281	Htr2c
ENSMUST00000097646.4	0	MGI:1915296	2700062C07Rik
ENSMUST00000099482.4	0	MGI:1347078	Grk6
ENSMUST00000099683.1	0	MGI:3641657	Gm10800

ENSMUST00000100789.10	0	MGI:1915896	Nadk2
ENSMUST00000102578.10	0	MGI:1915670	Ankrd13a
ENSMUST00000102581.10	0	MGI:2141207	Kctd10
ENSMUST00000102585.1	0	MGI:109167	Fgf11
ENSMUST00000102604.10	0	MGI:87978	Ak2
ENSMUST00000102626.9	0	MGI:97807	Ptpn5
ENSMUST00000102787.9	0	MGI:2442794	Dennd1a
ENSMUST00000103232.1	0	MGI:1924284	2510039O18Rik
ENSMUST00000105384.4	0	MGI:1351911	Ilvbl
ENSMUST00000105397.9	0	MGI:1915134	Cfap410
ENSMUST00000105987.8	0	MGI:1321151	Ptpru
ENSMUST00000105993.3	0	MGI:1914399	Nkain1
ENSMUST00000106236.8	0	MGI:1095412	Unc5c
ENSMUST00000106354.8	0	MGI:1858222	Septin9
ENSMUST00000106486.7	0	MGI:894676	Mast2
ENSMUST00000107032.2	0	MGI:2673002	Arhgef17
ENSMUST00000107165.7	0	MGI:2447063	Tenm4
ENSMUST00000107259.3	0	MGI:5504144	Gm27029
ENSMUST00000107737.10	0	MGI:1861380	Sphk2
ENSMUST00000107818.8	0	MGI:1918702	Ankrd40
ENSMUST00000107938.7	0	MGI:3613677	Shank1
ENSMUST00000107974.2	0	MGI:2686277	Iglon5
ENSMUST00000109309.8	0	MGI:1930016	Shank3
ENSMUST00000109605.4	0	MGI:88096	Atf4
ENSMUST00000109935.7	0	MGI:3702158	Syndig1
ENSMUST00000110181.7	0	MGI:2445356	Smox
ENSMUST00000110208.7	0	MGI:1916846	Ap5s1
ENSMUST00000110276.7	0	MGI:1924408	Coq6
ENSMUST00000111171.5	0	MGI:2137624	Pom121
ENSMUST00000111263.8	0	MGI:2135272	Vangl2
ENSMUST00000111999.7	0	MGI:1336167	Prkab1
ENSMUST00000112456.8	0	MGI:1889583	Sh3kbp1
ENSMUST00000112529.7	0	MGI:109490	Sms
ENSMUST00000112992.8	0	MGI:1916851	Dab2ip
ENSMUST00000113334.7	0	MGI:1920234	Ciz1
ENSMUST00000113495.8	0	MGI:3039562	Taf9b
ENSMUST00000113585.8	0	MGI:1346042	Mgll
ENSMUST00000113654.7	0	MGI:2652847	Lrrc8a
ENSMUST00000113763.7	0	MGI:1098824	Odf2

ENSMUST00000113975.2	0	MGI:1858226	Slc5a3
ENSMUST00000114195.7	0	MGI:1933820	Jam2
ENSMUST00000114777.9	0	MGI:1096566	Pias2
ENSMUST00000114943.10	0	MGI:94869	Dcc
ENSMUST00000115576.2	0	MGI:1913893	Lix1
ENSMUST00000116359.2	0	MGI:1916214	Ctc1
ENSMUST00000116429.8	0	MGI:1309465	Slc12a4
ENSMUST00000117236.7	0	MGI:90168	Dcaf11
ENSMUST00000117363.8	0	MGI:107236	Lsg1
ENSMUST00000118360.7	0	MGI:1928743	Kcnd3
ENSMUST00000119033.7	0	MGI:1917820	Tent4b
ENSMUST00000119567.7	0	MGI:1917682	Rufy2
ENSMUST00000122328.7	0	MGI:1916925	Pxdn
ENSMUST00000123791.7	0	MGI:1925230	Sbf1
ENSMUST00000132151.7	0	MGI:2442443	Fsd1l
ENSMUST00000132676.7	0	MGI:1921701	Pgs1
ENSMUST00000133925.7	0	MGI:1195972	Terf2
ENSMUST00000136026.7	0	MGI:1919096	Syce2
ENSMUST00000138880.8	0	MGI:2146285	Nol12
ENSMUST00000139454.2	0	MGI:5547771	Gm28035
ENSMUST00000142742.8	0	MGI:97360	Nos1
ENSMUST00000143829.4	0	MGI:1923241	Slain2
ENSMUST00000147639.7	0	MGI:2652840	Vgll4
ENSMUST00000147835.3	0	MGI:104605	Rasl2-9
ENSMUST00000150627.8	0	MGI:1914240	Tmem134
ENSMUST00000153128.1	0	MGI:2151060	Cnnm4
ENSMUST00000153360.7	0	MGI:2443014	Ino80c
ENSMUST00000153424.7	0	MGI:107432	Drp2
ENSMUST00000155998.1	0	MGI:1337008	Ankfy1
ENSMUST00000156440.7	0	MGI:1330262	Zpr1
ENSMUST00000159109.1	0	MGI:2674092	Zfp609
ENSMUST00000160616.7	0	MGI:1919410	Tmem163
ENSMUST00000160853.7	0	MGI:1916127	Maf1
ENSMUST00000161639.7	0	MGI:2444783	Gfm2
ENSMUST00000161779.7	0	MGI:1314872	Hipk2
ENSMUST00000164848.2	0	MGI:2685758	Siah3
ENSMUST00000165666.8	0	MGI:2442934	Minar2
ENSMUST00000165952.8	0	MGI:1333883	Lats1
ENSMUST00000167042.7	0	MGI:6121629	Gm49396

ENSMUST00000167588.8	0	MGI:3045323	Trim67
ENSMUST00000168338.1	0	MGI:2443487	Trmt61a
ENSMUST00000168515.7	0	MGI:1915757	Ppfia4
ENSMUST00000169212.8	0	MGI:95524	Fgfr3
ENSMUST00000170141.2	0	MGI:1929285	Orc6
ENSMUST00000171143.1	0	MGI:3036259	Fam102b
ENSMUST00000171300.7	0	MGI:1915152	Sumf2
ENSMUST00000171696.7	0	MGI:1923330	Ttpal
ENSMUST00000172314.8	0	MGI:894659	Hbp1
ENSMUST00000172856.7	0	MGI:2177308	Znrf1
ENSMUST00000177374.7	0	MGI:1342335	Asap1
ENSMUST00000178473.7	0	MGI:1914763	Faap20
ENSMUST00000179721.7	0	MGI:2444928	Phlpp2
ENSMUST00000179802.1	0	MGI:2142888	Cmtm4
ENSMUST00000180359.7	0	MGI:1915938	Abhd4
ENSMUST00000189400.6	0	MGI:1921479	Paqr8
ENSMUST00000189588.6	0	MGI:1932411	Tmem108
ENSMUST00000191497.1	0	MGI:1859645	Usp27x
ENSMUST00000197216.2	0	MGI:5662654	Gm42517
ENSMUST00000207023.1	0	MGI:98847	Kdm3a
ENSMUST00000212987.1	0	MGI:2676970	Sipa1l2
ENSMUST00000213524.2	0	MGI:1858236	Celsr3
ENSMUST00000214117.1	0	MGI:107936	Il18
ENSMUST00000214357.1	0	MGI:2685354	Igsf9b
ENSMUST00000215396.1	0	MGI:88537	Csk
ENSMUST00000215474.1	0	MGI:104803	Siae
ENSMUST00000216172.1	0	MGI:2444847	Ncoa7
ENSMUST00000218802.1	0	MGI:6121566	Gm49358
ENSMUST00000222485.1	0	MGI:1916867	Pitrm1
ENSMUST00000222544.1	0	MGI:97904	Trim27
ENSMUST00000223982.1	0	MGI:102694	Ptprm
ENSMUST00000225141.1	0	MGI:1926233	Fibp
ENSMUST00000227275.1	0	MGI:1913520	Retreg1
ENSMUST00000229744.1	0	MGI:1858219	Rcan2
ENSMUST00000231700.1	0	MGI:2685625	Zdhhc23
ENSMUST00000234473.1	0	MGI:107387	Aqp4
ENSMUST00000236953.1	0	MGI:1919451	Otud6b
ENSMUST00000238856.1	0	MGI:1858308	Tnk2
ENSMUST00000239081.1	0	MGI:2135937	Sbk1

ENSMUST00000239111.1	0	MGI:2443012	Tns3
ENSMUST00000239113.1	0	MGI:1347344	Gla
ENSMUST00000001672.11	0	MGI:1316717	Ifrd1
ENSMUST00000020408.15	0	MGI:96952	Mdm2
ENSMUST00000020687.14	0	MGI:1353578	Pttg1
ENSMUST00000023344.9	0	MGI:1921352	Slc35a5
ENSMUST00000025997.6	0	MGI:1923729	Smndc1
ENSMUST00000027499.12	0	MGI:1858494	Bok
ENSMUST00000027802.8	0	MGI:1277956	Pycr2
ENSMUST00000030080.6	0	MGI:2443882	Snx30
ENSMUST00000030201.13	0	MGI:1354951	Gne
ENSMUST00000032768.14	0	MGI:1352452	Nr2f2
ENSMUST00000033509.14	0	MGI:107822	Ebp
ENSMUST00000034413.7	0	MGI:1916387	Vstm5
ENSMUST00000034801.10	0	MGI:88137	Bckdhb
ENSMUST00000036156.5	0	MGI:2385205	Ipo13
ENSMUST00000036665.9	0	MGI:1914792	Cog6
ENSMUST00000041042.12	0	MGI:2144164	Tbc1d10a
ENSMUST00000043767.8	0	MGI:1914281	Upf3a
ENSMUST00000045078.12	0	MGI:1923488	Grhpr
ENSMUST00000050668.3	0	MGI:2445100	Zfp770
ENSMUST00000052932.9	0	MGI:2443226	Pde12
ENSMUST00000056085.5	0	MGI:1919082	Csl
ENSMUST00000065539.5	0	MGI:1344365	Dand5
ENSMUST00000073456.8	0	MGI:1913883	Nsa2
ENSMUST00000075994.10	0	MGI:1933157	Pdzrn3
ENSMUST00000081574.7	0	MGI:104966	Syt17
ENSMUST00000084497.11	0	MGI:1926116	Abraxas2
ENSMUST00000089052.10	0	MGI:1922462	Rnf121
ENSMUST00000091628.10	0	MGI:104783	Atxn1
ENSMUST00000106609.7	0	MGI:2385186	Clcc1
ENSMUST00000112610.1	0	MGI:96904	M6pr
ENSMUST00000113242.4	0	MGI:1351631	Sh2d3c
ENSMUST00000113896.7	0	MGI:1915566	Apoo
ENSMUST00000119570.7	0	MGI:1890149	Tpm3
ENSMUST00000128302.7	0	MGI:1916976	Smyd3
ENSMUST00000145112.8	0	MGI:96270	Trmt2a
ENSMUST00000153031.1	0	MGI:2444637	Sh3rf3
ENSMUST00000159292.7	0	MGI:1913496	Osgep

ENSMUST00000167316.7	0	MGI:1919861	Zfp655
ENSMUST00000170705.7	0	MGI:1915403	Gtf2e2
ENSMUST00000176978.7	0	MGI:1933131	Kcnip4
ENSMUST00000177594.7	0	MGI:2445165	Elmod2
ENSMUST00000181904.2	0	MGI:2442112	Cxxc4
ENSMUST00000209467.1	0	MGI:5804828	Gm45713
ENSMUST00000216692.1	0	MGI:6121526	Gm49337
ENSMUST00000218362.1	0	MGI:1916730	Ttc9
ENSMUST00000223428.1	0	MGI:3649931	Rps18-ps5
ENSMUST00000225200.1	0	MGI:1306780	Egr3
ENSMUST00000000388.14	0	MGI:2384924	Ccm2
ENSMUST00000001081.9	0	MGI:1913339	Rmnd5b
ENSMUST00000001520.12	0	MGI:1928277	Afg3l1
ENSMUST00000002350.10	0	MGI:1914813	Ciao3
ENSMUST00000002699.6	0	MGI:1928488	Akap8
ENSMUST00000003238.13	0	MGI:1926805	Foxj2
ENSMUST00000003438.10	0	MGI:3050117	Mob3a
ENSMUST00000003645.8	0	MGI:95412	Ercc1
ENSMUST00000003907.13	0	MGI:104541	Gcdh
ENSMUST00000004327.10	0	MGI:105979	G6pdx
ENSMUST00000009707.13	0	MGI:1353596	Tor2a
ENSMUST00000010189.2	0	MGI:1930765	Tmem115
ENSMUST00000015858.11	0	MGI:1924143	Cers2
ENSMUST00000017153.3	0	MGI:1349164	Sdc4
ENSMUST00000018353.13	0	MGI:1929004	Stk4
ENSMUST00000018821.8	0	MGI:1196386	Slc25a39
ENSMUST00000018841.2	0	MGI:1929608	Aatf
ENSMUST00000019439.8	0	MGI:1915616	Tmem129
ENSMUST00000020643.3	0	MGI:2429762	Rufy1
ENSMUST00000020647.9	0	MGI:1915317	Mrnip
ENSMUST00000021039.11	0	MGI:109152	Lig3
ENSMUST00000021045.12	0	MGI:1100867	Asic2
ENSMUST00000021168.13	0	MGI:2448493	Wscd1
ENSMUST00000021681.3	0	MGI:2442543	Vash1
ENSMUST00000021714.8	0	MGI:1915770	Zfyve21
ENSMUST00000023087.12	0	MGI:1100520	Twf1
ENSMUST00000023432.9	0	MGI:1261838	Nit2
ENSMUST00000024981.8	0	MGI:1196260	Jpt2
ENSMUST00000025823.5	0	MGI:1336895	Rce1

ENSMUST00000026737.11	0	MGI:1915044	Shisa5
ENSMUST00000026917.9	0	MGI:106206	Nrp1
ENSMUST00000027867.6	0	MGI:1922145	Ccdc181
ENSMUST00000027940.5	0	MGI:1914200	Pacc1
ENSMUST00000028257.2	0	MGI:1918521	Gca
ENSMUST00000029459.9	0	MGI:1338001	Gdap2
ENSMUST00000029686.3	0	MGI:1298211	Hcn3
ENSMUST00000030206.9	0	MGI:1916338	Snapc3
ENSMUST00000030760.14	0	MGI:1913397	Necap2
ENSMUST00000030791.11	0	MGI:1914243	Smarcd3
ENSMUST00000030895.11	0	MGI:1891749	Wrap73
ENSMUST00000031037.13	0	MGI:1345280	Slc30a3
ENSMUST00000031251.15	0	MGI:2149821	Hsd17b11
ENSMUST00000031914.5	0	MGI:2146066	Ccdc184
ENSMUST00000032559.16	0	MGI:107612	Rtn2
ENSMUST00000032815.10	0	MGI:104752	Nfkbib
ENSMUST00000032841.6	0	MGI:1914558	Mrpl46
ENSMUST00000032844.6	0	MGI:1913521	Tmem126a
ENSMUST00000032879.14	0	MGI:1923235	Rab30
ENSMUST00000032936.7	0	MGI:1891763	Ppp4c
ENSMUST00000033030.13	0	MGI:1931144	Parva
ENSMUST00000033133.11	0	MGI:1915115	Rgs10
ENSMUST00000033513.9	0	MGI:1859648	Ftsj1
ENSMUST00000034277.13	0	MGI:1343095	Emc8
ENSMUST00000034385.11	0	MGI:109599	Has3
ENSMUST00000034465.8	0	MGI:1913773	2810004N23Rik
ENSMUST00000034552.7	0	MGI:103224	Fdx1
ENSMUST00000034949.9	0	MGI:2660884	Csnk1g1
ENSMUST00000036111.9	0	MGI:2385255	Mrps35
ENSMUST00000036631.13	0	MGI:1914209	Dusp26
ENSMUST00000036744.7	0	MGI:1913954	Rbm4b
ENSMUST00000036796.7	0	MGI:2443199	Fstl4
ENSMUST00000038096.7	0	MGI:2679256	Fn3krp
ENSMUST00000038149.12	0	MGI:1341793	Pbx2
ENSMUST00000039286.4	0	MGI:1277186	Atg5
ENSMUST00000039763.13	0	MGI:2384905	Ginm1
ENSMUST00000040307.5	0	MGI:2446144	Marchf9
ENSMUST00000040961.2	0	MGI:2136401	Pabpc5
ENSMUST00000041010.14	0	MGI:1921443	Ccdc9

ENSMUST00000041190.16	0	MGI:1858420	Mcrs1
ENSMUST00000042390.4	0	MGI:2684954	Man1b1
ENSMUST00000042732.5	0	MGI:2447775	Fbxo45
ENSMUST00000042818.10	0	MGI:1355297	Pim3
ENSMUST00000042889.11	0	MGI:1920145	Setd5
ENSMUST00000044252.6	0	MGI:1347072	Nubp2
ENSMUST00000044332.15	0	MGI:2386052	Cerk
ENSMUST00000046425.15	0	MGI:105968	Txlna
ENSMUST00000046663.7	0	MGI:1920629	Dcbld2
ENSMUST00000047721.9	0	MGI:2384313	Rrp9
ENSMUST00000048044.11	0	MGI:2442186	Trub2
ENSMUST00000048263.13	0	MGI:2675859	Wapl
ENSMUST00000048305.9	0	MGI:1915984	Ppp4r3a
ENSMUST00000048731.5	0	MGI:1917670	Arpin
ENSMUST00000048790.6	0	MGI:2685214	Prrg3
ENSMUST00000048923.6	0	MGI:2142186	Spred3
ENSMUST00000049022.14	0	MGI:1196617	Ninj1
ENSMUST00000050735.11	0	MGI:1347071	Zfp260
ENSMUST00000051129.9	0	MGI:2143657	Lrrc75b
ENSMUST00000051186.8	0	MGI:2145955	Prkaa1
ENSMUST00000051888.3	0	MGI:1919173	Borcs6
ENSMUST00000053744.8	0	MGI:1919202	Riox1
ENSMUST00000053981.5	0	MGI:1915868	1110012L19Rik
ENSMUST00000054387.7	0	MGI:1330805	Rab33b
ENSMUST00000055168.4	0	MGI:96654	Kcna1
ENSMUST00000055688.9	0	MGI:2446217	Phf13
ENSMUST00000056176.7	0	MGI:102718	Vav2
ENSMUST00000056907.6	0	MGI:2444720	Smcr8
ENSMUST00000057792.8	0	MGI:106687	Pon2
ENSMUST00000058659.8	0	MGI:98852	Tst
ENSMUST00000060433.9	0	MGI:1861712	Tssc4
ENSMUST00000060798.5	0	MGI:2147162	Unc119b
ENSMUST00000061390.8	0	MGI:2447586	Fkrp
ENSMUST00000061995.9	0	MGI:1913951	Spryd4
ENSMUST00000062694.15	0	MGI:1346865	Mapk14
ENSMUST00000062755.9	0	MGI:1915024	Borcs5
ENSMUST00000062855.14	0	MGI:1917677	Mier2
ENSMUST00000063976.8	0	MGI:2686271	Opa3
ENSMUST00000064595.14	0	MGI:2685438	Asap2

ENSMUST00000066052.11	0	MGI:1920462	3110082I17Rik
ENSMUST00000066610.7	0	MGI:1923497	Inka2
ENSMUST00000066632.13	0	MGI:1196310	Angel2
ENSMUST00000066675.9	0	MGI:1923616	Mtif3
ENSMUST00000066984.13	0	MGI:1345669	Gtf2h2
ENSMUST00000068044.13	0	MGI:2384851	Slc39a14
ENSMUST00000069476.4	0	MGI:2675858	Rtl6
ENSMUST00000069772.15	0	MGI:1917459	Tmem143
ENSMUST00000070070.7	0	MGI:1927638	Dnaja4
ENSMUST00000070552.13	0	MGI:1921559	Osbp2
ENSMUST00000071539.9	0	MGI:1917056	Slc39a11
ENSMUST00000072866.11	0	MGI:2149543	Ubap1
ENSMUST00000076169.3	0	MGI:2686040	Mtx3
ENSMUST00000081790.14	0	MGI:1926082	Cacul1
ENSMUST00000087215.6	0	MGI:1928902	Cnot9
ENSMUST00000088217.11	0	MGI:1336172	Tbl1x
ENSMUST00000089494.5	0	MGI:2446510	Il17d
ENSMUST00000089688.5	0	MGI:101900	Mmp14
ENSMUST00000090473.6	0	MGI:1927653	Gpr88
ENSMUST00000090522.4	0	MGI:1918313	Zfp597
ENSMUST00000093298.11	0	MGI:2150019	Spred2
ENSMUST00000099373.11	0	MGI:2151054	Cnnm2
ENSMUST00000099955.3	0	MGI:106595	Fam89b
ENSMUST00000101165.8	0	MGI:1919363	Adck1
ENSMUST00000102762.9	0	MGI:1913736	Acot11
ENSMUST00000107724.8	0	MGI:1343085	Spop
ENSMUST00000108277.2	0	MGI:104961	Tnfaip1
ENSMUST00000108872.8	0	MGI:2445299	Slc36a1
ENSMUST00000109214.7	0	MGI:1933159	Rnf114
ENSMUST00000109352.7	0	MGI:1913710	Sys1
ENSMUST00000110896.1	0	MGI:97527	Pdgfa
ENSMUST00000111118.7	0	MGI:2444263	Tcp11l1
ENSMUST00000111737.2	0	MGI:97245	Mxi1
ENSMUST00000113262.1	0	MGI:1921373	Foxp4
ENSMUST00000113682.8	0	MGI:1929864	Myg1
ENSMUST00000114126.8	0	MGI:1918366	Stx18
ENSMUST00000114988.7	0	MGI:2146854	Fam234a
ENSMUST00000116605.7	0	MGI:1860374	Mad2l1
ENSMUST00000121534.7	0	MGI:1349388	Cops7b

ENSMUST00000130320.7	0	MGI:1917004	Fbxo7
ENSMUST00000131384.2	0	MGI:2141989	Grwd1
ENSMUST00000143916.7	0	MGI:1923433	Celf6
ENSMUST00000147545.7	0	MGI:1923801	Ccdc6
ENSMUST00000150639.1	0	MGI:1891740	Dact1
ENSMUST00000152080.7	0	MGI:2142403	Slc35e1
ENSMUST00000160300.1	0	MGI:107492	Nxph1
ENSMUST00000160959.7	0	MGI:1098806	Commd2
ENSMUST00000164163.7	0	MGI:104295	Sla
ENSMUST00000164273.8	0	MGI:1860055	Panx1
ENSMUST00000164589.8	0	MGI:1921083	Fam98c
ENSMUST00000165630.2	0	MGI:1338026	Tom1
ENSMUST00000166040.8	0	MGI:1860075	Ppt2
ENSMUST00000167023.7	0	MGI:1915980	Dus1l
ENSMUST00000168003.8	0	MGI:2176159	Cyp26b1
ENSMUST00000170759.2	0	MGI:1858435	Gpatch11
ENSMUST00000176565.7	0	MGI:1914004	Foxp1
ENSMUST00000176652.7	0	MGI:3042141	Traf7
ENSMUST00000177054.7	0	MGI:1858178	Usp2
ENSMUST00000179556.1	0	MGI:2442951	Zfp574
ENSMUST00000180252.2	0	MGI:2685169	Tmem151b
ENSMUST00000193286.5	0	MGI:1925517	Klhdc8b
ENSMUST00000210282.1	0	MGI:2685003	Zbtb45
ENSMUST00000218766.1	0	MGI:1921642	Specc1l
ENSMUST00000219109.1	0	MGI:105933	Rab3ip
ENSMUST00000228977.1	0	MGI:3818630	Sco2
ENSMUST00000235900.1	0	MGI:1913275	Rcl1
ENSMUST00000019723.7	0.002032924	MGI:2156020	Mydgf
ENSMUST00000021450.5	0.002032924	MGI:2135760	Sgpp1
ENSMUST00000022909.9	0.002032924	MGI:2684929	Dcaf13
ENSMUST00000028648.2	0.002032924	MGI:1933945	Syt13
ENSMUST00000040184.3	0.002032924	MGI:109524	Trpc5
ENSMUST00000076807.6	0.002032924	MGI:1935201	Pcdhgc3
ENSMUST00000094993.2	0.004151506	MGI:2180122	Klhl9
ENSMUST00000174510.7	0.004151506	MGI:1343188	Ube2g2
ENSMUST00000014750.14	0.005770531	MGI:1915113	Slc25a11
ENSMUST00000021667.6	0.005770531	MGI:1921566	Isca2
ENSMUST00000028225.11	0.005770531	MGI:1914248	Psmd5
ENSMUST00000033025.6	0.005770531	MGI:1353593	Lcmt1

ENSMUST00000052120.13	0.005770531	MGI:2443143	Wdr3
ENSMUST00000061419.8	0.005770531	MGI:3641855	Gm9833
ENSMUST00000061568.8	0.005770531	MGI:2442595	Slc36a4
ENSMUST00000100802.10	0.005770531	MGI:1915814	Nufip2
ENSMUST00000102515.9	0.005770531	MGI:97594	Prkacb
ENSMUST00000118193.7	0.005770531	MGI:1914765	Ttc33
ENSMUST00000127756.7	0.005770531	MGI:99675	Adcy3
ENSMUST00000185362.6	0.005770531	MGI:1916617	Glrx2
ENSMUST00000029002.8	0.006924846	MGI:98241	Stmn2
ENSMUST00000062678.10	0.007165319	MGI:1203500	Rrp1
ENSMUST00000026433.8	0.007749398	MGI:1915344	Smarcc2
ENSMUST00000020630.7	0.00876035	MGI:1342292	Hspa4
ENSMUST00000196456.4	0.009353115	MGI:1913129	Rbm8a
ENSMUST00000088169.6	0.009686012	MGI:1339970	Rtn3
ENSMUST00000034432.6	0.00975695	MGI:1344403	Cfdp1
ENSMUST00000029125.9	0.010263394	MGI:1921502	Armc1
ENSMUST00000120539.7	0.013042531	MGI:109360	Lmo4
ENSMUST00000103053.9	0.013726094	MGI:1915372	Nkain4
ENSMUST00000237301.1	0.013726094	MGI:1351615	Add3
ENSMUST00000108849.7	0.014157375	MGI:1924068	Mfap3
ENSMUST00000026221.6	0.014725856	MGI:98240	Scd2
ENSMUST00000053748.15	0.014836496	MGI:103009	Epb41l2
ENSMUST00000113147.7	0.014861506	MGI:1917418	Gprasp1
ENSMUST00000027478.6	0.015170848	MGI:1914523	Ndufa10
ENSMUST00000030014.8	0.016063227	MGI:1891840	Ncbp1
ENSMUST00000031426.13	0.016063227	MGI:1098597	Ift81
ENSMUST00000039064.7	0.016063227	MGI:3645930	Fam124a
ENSMUST00000041830.9	0.016063227	MGI:1913867	Ntmt1
ENSMUST00000043604.5	0.016063227	MGI:95766	Gna11
ENSMUST00000045702.5	0.016063227	MGI:2443286	Slc2a6
ENSMUST00000089473.4	0.016063227	MGI:1923215	Zdhhc20
ENSMUST00000092688.11	0.016063227	MGI:1915356	Nt5c3b
ENSMUST00000100527.12	0.016063227	MGI:1915209	Puf60
ENSMUST00000149248.8	0.016063227	MGI:2385906	Spg7
ENSMUST00000091403.5	0.016635553	MGI:88075	Arsb
ENSMUST00000172298.7	0.018544348	MGI:1858202	Rab11a
ENSMUST00000235547.1	0.018875873	MGI:95742	Glo1
ENSMUST00000017851.3	0.018947353	MGI:1349457	Serinc3
ENSMUST00000020365.14	0.018947353	MGI:1915364	Pwwp3a

ENSMUST00000025276.14	0.018947353	MGI:1916528	Rmc1
ENSMUST00000025357.8	0.018947353	MGI:1337062	Ap3s1
ENSMUST00000027952.11	0.018947353	MGI:107684	Plxna2
ENSMUST00000028781.8	0.018947353	MGI:1341628	Atrn
ENSMUST00000029194.11	0.018947353	MGI:106203	Skil
ENSMUST00000029269.11	0.018947353	MGI:1355319	Exosc9
ENSMUST00000030030.14	0.018947353	MGI:1344413	Tex10
ENSMUST00000031250.13	0.018947353	MGI:1921417	Nudt9
ENSMUST00000031351.10	0.018947353	MGI:1929500	Arl6ip4
ENSMUST00000031606.9	0.018947353	MGI:1330842	Rasal1
ENSMUST00000033431.13	0.018947353	MGI:1330823	Slc25a14
ENSMUST00000037285.9	0.018947353	MGI:1927140	Git1
ENSMUST00000041048.5	0.018947353	MGI:2443195	Orai2
ENSMUST00000061156.9	0.018947353	MGI:1918007	Hacd2
ENSMUST00000071361.12	0.018947353	MGI:1916463	Zfp428
ENSMUST00000079754.10	0.018947353	MGI:2443075	Cpeb3
ENSMUST00000088357.11	0.018947353	MGI:104725	Atn1
ENSMUST00000090413.5	0.018947353	MGI:1919230	Utp3
ENSMUST00000092123.10	0.018947353	MGI:1278321	Epc2
ENSMUST00000093458.10	0.018947353	MGI:2678085	Sugp2
ENSMUST00000102608.9	0.018947353	MGI:2140475	AU040320
ENSMUST00000106058.7	0.018947353	MGI:1858211	Zranb2
ENSMUST00000107609.3	0.018947353	MGI:2445107	Tmem245
ENSMUST00000109288.8	0.018947353	MGI:109188	Kif21a
ENSMUST00000109556.8	0.018947353	MGI:2442978	Ogfod1
ENSMUST00000115151.4	0.018947353	MGI:2146388	Ubxn7
ENSMUST00000119945.7	0.018947353	MGI:1928139	Mrps10
ENSMUST00000141112.1	0.018947353	MGI:2140466	AU022252
ENSMUST00000159348.2	0.018947353	MGI:1919667	2700081O15Rik
ENSMUST00000159692.7	0.018947353	MGI:106250	Ermp1
ENSMUST00000160144.8	0.018947353	MGI:1915281	Rnf146
ENSMUST00000168461.7	0.018947353	MGI:1922484	Rnf19b
ENSMUST00000180572.1	0.018947353	MGI:87961	Agrn
ENSMUST00000001186.10	0.019892974	MGI:1913868	Snrnp27
ENSMUST00000049941.11	0.019892974	MGI:1918882	Scn3b
ENSMUST00000040167.10	0.020343982	MGI:1913667	Mat2b
ENSMUST00000026723.8	0.020814402	MGI:96217	Hprt
ENSMUST00000102611.9	0.022327362	MGI:1930780	Myh10
ENSMUST00000034607.9	0.023275334	MGI:2387591	Arcn1

ENSMUST00000102507.9	0.023275334	MGI:104652	Capzb
ENSMUST00000176540.7	0.023275334	MGI:97495	Pbx1
ENSMUST00000210477.1	0.023275334	MGI:1924880	Prdm8
ENSMUST00000037315.12	0.024747041	MGI:1914344	Abhd2
ENSMUST00000195826.5	0.024747041	MGI:1306801	Pcdh9
ENSMUST00000027438.7	0.024999482	MGI:97286	Ncl
ENSMUST00000052798.13	0.025488466	MGI:1929282	Ptges3
ENSMUST00000042834.3	0.027980312	MGI:1913944	Uqcrfs1
ENSMUST00000110636.8	0.028347625	MGI:1913755	Zmynd11
ENSMUST00000030284.9	0.028609908	MGI:1352759	Rnf11
ENSMUST00000037127.14	0.028609908	MGI:2153887	Eri3
ENSMUST00000089299.5	0.028609908	MGI:3845555	Npcd
ENSMUST00000109349.8	0.028609908	MGI:106562	Dbndd2
ENSMUST00000146905.1	0.028609908	MGI:99437	Arl4a
ENSMUST00000200157.4	0.028609908	MGI:1914536	Ift22
ENSMUST00000003038.11	0.029265313	MGI:101920	Ap2a2
ENSMUST00000020057.15	0.029265313	MGI:2135609	Lin7a
ENSMUST00000020543.12	0.029265313	MGI:1914829	Cpeb4
ENSMUST00000021913.15	0.029265313	MGI:1338011	Auh
ENSMUST00000026826.13	0.029265313	MGI:2183454	Rab40c
ENSMUST00000035154.3	0.029265313	MGI:1913452	1110059G10Rik
ENSMUST00000061179.11	0.029265313	MGI:2385139	Rabgap1
ENSMUST00000062904.10	0.029265313	MGI:2443386	Dnajc11
ENSMUST00000098190.9	0.029265313	MGI:1098604	Rragd
ENSMUST00000161600.7	0.029265313	MGI:1098784	Fam126b
ENSMUST00000169707.7	0.029265313	MGI:1328363	Ccnt1
ENSMUST00000170795.2	0.029265313	MGI:1930089	Mcm3ap
ENSMUST00000055770.3	0.030568173	MGI:1931523	H1f1
ENSMUST00000091458.12	0.031226667	MGI:1352451	Nr2f1
ENSMUST00000160953.7	0.031814226	MGI:1924879	Sphkap
ENSMUST00000021091.14	0.032588729	MGI:109520	Pafah1b1
ENSMUST00000082152.4	0.032639566	MGI:2444266	Ube2o
ENSMUST00000040234.8	0.032715704	MGI:2141599	Tsen2
ENSMUST00000073878.11	0.033573827	MGI:1298378	Dhcr7
ENSMUST00000074628.12	0.033738064	MGI:104709	Wbp2
ENSMUST00000054220.9	0.033883643	MGI:1924058	Rpl18a
ENSMUST00000078222.8	0.033883643	MGI:99441	Ckmt1
ENSMUST00000019226.13	0.034491746	MGI:1915517	Slc25a22
ENSMUST00000171796.7	0.03506066	MGI:108391	Kif1a

ENSMUST00000020553.4	0.035755834	MGI:1915294	Chac2
ENSMUST00000030332.6	0.035755834	MGI:1914555	Gpx7
ENSMUST00000033941.6	0.035755834	MGI:97610	Plat
ENSMUST00000034396.13	0.035755834	MGI:1924366	Mtmr2
ENSMUST00000057676.6	0.035755834	MGI:1914635	Ubald2
ENSMUST00000072080.9	0.035755834	MGI:1914394	Lrrc40
ENSMUST00000103152.10	0.035755834	MGI:1933126	Cdk5rap3
ENSMUST00000108127.3	0.035755834	MGI:1920455	C9orf72
ENSMUST00000170998.8	0.035755834	MGI:106921	Scn2b
ENSMUST00000216286.1	0.035755834	MGI:1921763	Neto2
ENSMUST00000022614.6	0.035973445	MGI:1914429	Ccdc25
ENSMUST00000023486.14	0.035973445	MGI:98822	Tfrc
ENSMUST00000111159.1	0.035973445	MGI:1345147	Akt3
ENSMUST00000072634.14	0.036057407	MGI:88047	Aplp2
ENSMUST00000101102.1	0.036148251	MGI:2663511	Reps2
ENSMUST00000022573.16	0.038097231	MGI:95421	Esd
ENSMUST00000229854.1	0.038467365	MGI:108202	Pcbp2
ENSMUST00000001242.8	0.038621292	MGI:1351861	Gatd3a
ENSMUST00000037813.4	0.038621292	MGI:95780	Gnaz
ENSMUST00000102525.10	0.03958846	MGI:1928375	Arpc3
ENSMUST00000211214.1	0.03958846	MGI:1342299	Ruvbl2
ENSMUST00000106348.7	0.039938503	MGI:87994	Aldoa
ENSMUST00000106348.7	0.039938503	MGI:87994	Aldoa
ENSMUST00000106348.7	0.039938503	MGI:87994	Aldoa
ENSMUST00000106348.7	0.039938503	MGI:87994	Aldoa
ENSMUST00000001485.9	0.040215434	MGI:1333801	Mrpl10
ENSMUST00000066791.6	0.040215434	MGI:2144891	Tmem179
ENSMUST00000093169.2	0.040215434	MGI:3650635	Gm12166
ENSMUST00000112507.3	0.040215434	MGI:1919972	Fam98a
ENSMUST00000136008.7	0.040215434	MGI:1914706	Ergic2
ENSMUST00000169020.7	0.040215434	MGI:104967	Glg1
ENSMUST00000037380.14	0.042664367	MGI:1330848	Atp8a1
ENSMUST00000020446.10	0.043468399	MGI:1915462	Tmbim4
ENSMUST00000025904.11	0.043468399	MGI:1859821	Prdx5
ENSMUST00000031841.8	0.043468399	MGI:1933972	Tra2a
ENSMUST00000107843.10	0.043468399	MGI:107846	Prmt1
ENSMUST00000047889.12	0.043777469	MGI:88109	Atp1b2
ENSMUST00000001566.9	0.044427596	MGI:107812	Tubb5
ENSMUST00000002855.13	0.045606025	MGI:1915387	Kdelr1

ENSMUST00000022148.6	0.045946096	MGI:1925288	Mccc2
ENSMUST00000100330.9	0.045946096	MGI:2444121	Tanc2
ENSMUST00000127405.1	0.045946096	MGI:1098547	Nhp2
ENSMUST00000055990.7	0.046064074	MGI:1096317	Eef1a2
ENSMUST00000103075.10	0.047842388	MGI:104560	Nsf
ENSMUST00000104983.1	0.047896169	MGI:3612447	Rbm8a2
ENSMUST00000034138.6	0.048770869	MGI:1931882	Dnaja2
ENSMUST00000073868.8	0.048770869	MGI:106095	Naca
ENSMUST00000090569.9	0.049599959	MGI:1347009	Psma5
ENSMUST00000160027.7	0.049599959	MGI:104883	Psma3
ENSMUST00000221220.1	0.050344807	MGI:2673872	Syt16
ENSMUST00000222517.1	0.050344807	MGI:88564	Ctsl
ENSMUST00000028087.5	0.050458748	MGI:1915107	Ppp6c
ENSMUST00000184744.1	0.050458748	MGI:2145823	Kctd12
ENSMUST00000021425.7	0.050769235	MGI:2387603	Ahsa1
ENSMUST00000233710.1	0.052784156	MGI:6270466	Gm49804
ENSMUST00000102593.10	0.052969939	MGI:1860763	Eif3i
ENSMUST00000000476.14	0.053463689	MGI:97530	Pdgfra
ENSMUST00000001712.7	0.053463689	MGI:1298375	Cabin1
ENSMUST00000003906.12	0.053463689	MGI:1913840	Farsa
ENSMUST00000016294.7	0.053463689	MGI:1345185	Tenm1
ENSMUST00000019405.3	0.053463689	MGI:2443304	Map1s
ENSMUST00000021832.6	0.053463689	MGI:1926153	Wrnip1
ENSMUST00000023776.12	0.053463689	MGI:1928745	Slc4a8
ENSMUST00000025930.9	0.053463689	MGI:1339795	Smc3
ENSMUST00000026699.14	0.053463689	MGI:1351638	Mkln1
ENSMUST00000029355.8	0.053463689	MGI:2139740	Ppm1l
ENSMUST00000029911.11	0.053463689	MGI:1913875	Pnisr
ENSMUST00000029925.9	0.053463689	MGI:1915743	Ndufaf4
ENSMUST00000040202.14	0.053463689	MGI:2446242	Atxn2l
ENSMUST00000041589.5	0.053463689	MGI:1349721	Tob1
ENSMUST00000042288.7	0.053463689	MGI:2145525	Asb13
ENSMUST00000042868.5	0.053463689	MGI:88258	Camk4
ENSMUST00000043082.15	0.053463689	MGI:1914702	Pnpla8
ENSMUST00000045840.4	0.053463689	MGI:2441758	Gpr26
ENSMUST00000046603.14	0.053463689	MGI:2442153	Gak
ENSMUST00000046839.9	0.053463689	MGI:107757	Gfer
ENSMUST00000048155.15	0.053463689	MGI:1914289	Rbm25
ENSMUST00000051400.7	0.053463689	MGI:1932403	Cyp4x1

ENSMUST00000054960.7	0.053463689	MGI:2443921	Irf2bp2
ENSMUST00000057228.1	0.053463689	MGI:2136744	Pcdhb9
ENSMUST00000060067.11	0.053463689	MGI:1933786	Dnaja3
ENSMUST00000061331.13	0.053463689	MGI:1919942	Hnrnpll
ENSMUST00000071921.12	0.053463689	MGI:1344415	Dmtf1
ENSMUST00000074240.3	0.053463689	MGI:1927576	Dipk1b
ENSMUST00000075387.10	0.053463689	MGI:1935121	Sez6l
ENSMUST00000081946.4	0.053463689	MGI:1913775	Timm50
ENSMUST00000084474.5	0.053463689	MGI:2442466	Frmd3
ENSMUST00000086023.11	0.053463689	MGI:2137594	Galnt17
ENSMUST00000094639.9	0.053463689	MGI:1306776	Map1a
ENSMUST00000099329.4	0.053463689	MGI:1934835	Ube2n
ENSMUST00000113560.7	0.053463689	MGI:109606	Fnbp1
ENSMUST00000115804.8	0.053463689	MGI:1920909	Pdxdc1
ENSMUST00000140076.1	0.053463689	MGI:2387203	Ppat
ENSMUST00000168423.8	0.053463689	MGI:108051	Smad2
ENSMUST00000211887.1	0.053463689	MGI:2442402	Cnot1
ENSMUST00000211896.1	0.053463689	MGI:3646208	Btbd8
ENSMUST00000221877.1	0.053463689	MGI:1289332	Eipr1
ENSMUST00000225980.1	0.053463689	MGI:1354757	Atp9b
ENSMUST00000235957.1	0.053463689	MGI:109162	Gpam
ENSMUST00000022803.5	0.053581613	MGI:1194513	Psmb5
ENSMUST00000080882.10	0.053595849	MGI:88107	Atp1a3
ENSMUST00000030660.8	0.055333806	MGI:1351486	Trappc3
ENSMUST00000075491.13	0.055365265	MGI:1341070	Fkbp8
ENSMUST00000065865.9	0.055423761	MGI:1913481	Thoc7
ENSMUST00000025319.6	0.056096884	MGI:1914926	Rpp21
ENSMUST00000027795.13	0.056096884	MGI:1920228	Cnih3
ENSMUST00000028081.12	0.056096884	MGI:1914698	Plxdc2
ENSMUST00000029644.15	0.056096884	MGI:1922026	Ppa2
ENSMUST00000046283.15	0.056096884	MGI:1309463	Hspa13
ENSMUST00000047835.7	0.056096884	MGI:1919443	Scaf11
ENSMUST00000064701.7	0.056096884	MGI:2384987	B4galt7
ENSMUST00000088345.11	0.056096884	MGI:1353598	Mapk8ip3
ENSMUST00000108336.7	0.056096884	MGI:99207	Zfp60
ENSMUST00000119878.7	0.056096884	MGI:1330299	Dyrk1a
ENSMUST00000153627.7	0.056096884	MGI:1920036	Tdp1
ENSMUST00000167020.7	0.056096884	MGI:2387357	Wac
ENSMUST00000169464.8	0.056096884	MGI:2138865	Dlgap4

ENSMUST00000211283.1	0.056992135	MGI:1915816	Caly
ENSMUST00000022871.6	0.057697563	MGI:1349165	Sdc2
ENSMUST00000052138.10	0.057697563	MGI:1929871	Terf2ip
ENSMUST00000109418.1	0.057697563	MGI:2444508	Fitm2
ENSMUST00000009699.15	0.058773477	MGI:1328368	Cdk9
ENSMUST00000016105.8	0.058773477	MGI:87948	Adss
ENSMUST00000027495.14	0.058773477	MGI:97298	Septin2
ENSMUST00000027495.14	0.058773477	MGI:97298	Septin2
ENSMUST00000027495.14	0.058773477	MGI:97298	Septin2
ENSMUST00000027495.14	0.058773477	MGI:97298	Septin2
ENSMUST00000028205.9	0.058773477	MGI:2385132	BC005624
ENSMUST00000030028.4	0.058773477	MGI:1923549	Erp44
ENSMUST00000037912.11	0.058773477	MGI:2679255	Ssh2
ENSMUST00000112091.8	0.058773477	MGI:1890695	Rab9
ENSMUST00000132374.8	0.058773477	MGI:1916971	Nkiras1
ENSMUST00000190647.2	0.058773477	MGI:3044626	Begain
ENSMUST00000220578.1	0.058773477	MGI:1346087	Srp54a
ENSMUST00000238036.1	0.058773477	MGI:1289301	Ubxn1
ENSMUST00000022212.8	0.059203921	MGI:1099790	Plk2
ENSMUST00000067630.12	0.05954634	MGI:1914421	Dram2
ENSMUST00000068505.9	0.05954634	MGI:88264	Capn2
ENSMUST00000079703.10	0.05954634	MGI:1915751	Nsmce2
ENSMUST00000018449.10	0.060128385	MGI:2179381	Prpf8
ENSMUST00000023918.12	0.060128385	MGI:2152389	Ivns1abp
ENSMUST00000006625.7	0.060582517	MGI:1929092	Rbm14
ENSMUST00000025811.5	0.060582517	MGI:1915340	Yif1a
ENSMUST00000030568.13	0.060582517	MGI:107557	Sema3c
ENSMUST00000032508.10	0.060582517	MGI:95543	Fkbp4
ENSMUST00000043884.5	0.060946726	MGI:106036	Lrrn3
ENSMUST00000092325.10	0.060946726	MGI:2388640	Plppr3
ENSMUST00000120722.1	0.061245315	MGI:1914278	2610002M06Rik
ENSMUST00000023612.16	0.061494554	MGI:95456	Ets2
ENSMUST00000021447.8	0.061705742	MGI:1349473	Ppp2r5e
ENSMUST00000014546.14	0.061886975	MGI:106581	Tsg101
ENSMUST00000038128.14	0.061886975	MGI:1915789	Tmem109
ENSMUST00000166658.7	0.062508491	MGI:1928753	Pfdn5
ENSMUST00000090002.9	0.062872123	MGI:104819	Hnrnpa2b1
ENSMUST00000004072.9	0.06668146	MGI:1350927	Rpl8
ENSMUST00000041096.3	0.066753305	MGI:1353431	Pcsk1n

ENSMUST00000024932.11	0.069045748	MGI:88116	Atp6v0c
ENSMUST00000007482.7	0.069421966	MGI:108180	Mrpl49
ENSMUST00000037687.7	0.071518992	MGI:1914814	Tmem35a
ENSMUST00000211649.1	0.071782746	MGI:107996	Slc8a2
ENSMUST00000033236.8	0.072286687	MGI:2444479	Thumpd1
ENSMUST00000028083.5	0.072395271	MGI:107637	Psmb7
ENSMUST00000045562.5	0.073104871	MGI:1920112	Cox15
ENSMUST00000051589.8	0.074108821	MGI:1914868	Aasdhppt
ENSMUST00000140438.1	0.074108821	MGI:1928140	Mrps25
ENSMUST00000202281.3	0.074108821	MGI:1913299	Rogdi
ENSMUST00000215619.1	0.074108821	MGI:105387	Cdkn2d
ENSMUST00000016033.8	0.075369903	MGI:96836	Lta4h
ENSMUST00000038942.9	0.077001412	MGI:2441670	Pbxip1
ENSMUST00000080208.6	0.077001412	MGI:103008	Epb41l3
ENSMUST00000172951.1	0.077001412	MGI:1351344	Grm7
ENSMUST00000201452.3	0.078349065	MGI:105053	Hsph1
ENSMUST00000007236.4	0.078982932	MGI:1341881	Syngr3
ENSMUST00000002683.2	0.079194661	MGI:1196455	Ccdc97
ENSMUST00000009003.8	0.079194661	MGI:1927243	Rala
ENSMUST00000013693.10	0.079194661	MGI:1343485	Commd8
ENSMUST00000027067.14	0.079194661	MGI:95856	Gsta3
ENSMUST00000029135.14	0.079194661	MGI:1890410	Acss2
ENSMUST00000096753.4	0.079194661	MGI:1915943	Hnrnpul2
ENSMUST00000033157.9	0.08219401	MGI:1917566	Ndufab1
ENSMUST00000034524.4	0.082231979	MGI:1888981	Rexo2
ENSMUST00000020022.7	0.082300102	MGI:1931437	Smpdl3a
ENSMUST00000021082.6	0.082300102	MGI:1354954	Nt5c
ENSMUST00000026607.14	0.082300102	MGI:892979	Chm
ENSMUST00000029147.15	0.082300102	MGI:1316706	Nfs1
ENSMUST00000037280.4	0.082300102	MGI:2443626	Vps18
ENSMUST00000042824.12	0.082300102	MGI:2384589	Herc1
ENSMUST00000043160.12	0.082300102	MGI:1276102	Aqr
ENSMUST00000063669.7	0.082300102	MGI:2141813	Dhx32
ENSMUST00000070345.4	0.082300102	MGI:107622	Usp39
ENSMUST00000071891.11	0.082300102	MGI:1890496	Elac2
ENSMUST00000124341.7	0.082300102	MGI:109350	Slc4a3
ENSMUST00000022529.7	0.08325625	MGI:105992	Tkt
ENSMUST00000034623.7	0.085751482	MGI:1926211	Trappc4
ENSMUST00000058437.13	0.08600857	MGI:98073	Rpl7

ENSMUST00000122010.7	0.08600857	MGI:1929711	Anapc7
ENSMUST00000024763.9	0.087036244	MGI:1915815	Mrps18a
ENSMUST00000025314.6	0.087036244	MGI:1914089	Dele1
ENSMUST00000029445.12	0.087036244	MGI:97376	Nras
ENSMUST00000035027.12	0.087036244	MGI:1929897	Clstn2
ENSMUST00000035597.9	0.087036244	MGI:1920468	Sppl2b
ENSMUST00000037726.13	0.087036244	MGI:1921050	Tmtc4
ENSMUST00000040962.5	0.087036244	MGI:94203	Nudt19
ENSMUST00000042163.14	0.087036244	MGI:2442563	Naa25
ENSMUST00000061611.14	0.087036244	MGI:2443419	Rps6kc1
ENSMUST00000072079.8	0.087036244	MGI:1929721	Rrs1
ENSMUST00000113520.7	0.087036244	MGI:103178	Vegfa
ENSMUST00000114160.1	0.087036244	MGI:1351626	Fam50a
ENSMUST00000118919.8	0.087036244	MGI:1925658	Fam131a
ENSMUST00000128166.7	0.087036244	MGI:1923452	Zdhhc2
ENSMUST00000130481.1	0.087036244	MGI:3647874	Plcxd2
ENSMUST00000162175.8	0.087036244	MGI:2145645	Exoc5
ENSMUST00000166768.2	0.087036244	MGI:1926029	Spata2l
ENSMUST00000228284.1	0.087036244	MGI:1918724	Ppp6r2
ENSMUST00000110894.8	0.087607992	MGI:104890	Tpt1
ENSMUST00000112932.1	0.088562014	MGI:2442998	Zbtb6
ENSMUST00000008517.12	0.089001432	MGI:1916238	Prpf31
ENSMUST00000016081.12	0.089210123	MGI:1349392	Macroh2a1
ENSMUST00000049972.5	0.091025186	MGI:103033	Scg2
ENSMUST00000135192.7	0.091025186	MGI:1334462	Copa
ENSMUST00000110690.8	0.09175375	MGI:1914291	Oxct1
ENSMUST00000048393.7	0.091794871	MGI:1915368	Atg101
ENSMUST00000058814.6	0.091794871	MGI:2442454	Rab9b
ENSMUST00000068056.11	0.091794871	MGI:99240	Ddx39b
ENSMUST00000018568.3	0.093409536	MGI:1342307	Drg2
ENSMUST00000027464.8	0.093409536	MGI:1916413	Mrpl44
ENSMUST00000044299.2	0.093409536	MGI:98327	Sstr1
ENSMUST00000021471.12	0.093484737	MGI:1919986	Tmx1
ENSMUST00000025394.13	0.094711735	MGI:1913536	Sec11c
ENSMUST00000004281.9	0.095144914	MGI:1330301	Dyrk2
ENSMUST00000004294.11	0.095144914	MGI:109187	Kifc2
ENSMUST00000004920.3	0.095144914	MGI:1352758	Ulk2
ENSMUST00000005017.14	0.095144914	MGI:1194494	Hdgf
ENSMUST00000020564.6	0.095144914	MGI:106180	Shc2

ENSMUST00000022480.7	0.095144914	MGI:3616088	Ogdhl
ENSMUST00000024805.14	0.095144914	MGI:2385908	Cpne5
ENSMUST00000025109.7	0.095144914	MGI:1919782	Sap130
ENSMUST00000028200.8	0.095144914	MGI:1353568	Tor1a
ENSMUST00000030925.2	0.095144914	MGI:95622	Gabrd
ENSMUST00000032895.14	0.095144914	MGI:1858179	Nucb2
ENSMUST00000033532.6	0.095144914	MGI:1202294	Aff2
ENSMUST00000034763.9	0.095144914	MGI:2157166	Rp9
ENSMUST00000034905.8	0.095144914	MGI:104990	Gclc
ENSMUST00000036904.6	0.095144914	MGI:1923091	Rnf139
ENSMUST00000039327.10	0.095144914	MGI:2677061	Dagla
ENSMUST00000043551.10	0.095144914	MGI:1918047	Ankib1
ENSMUST00000048248.8	0.095144914	MGI:2685946	Brsk1
ENSMUST00000054234.9	0.095144914	MGI:1891638	Nek6
ENSMUST00000064404.7	0.095144914	MGI:1353653	Glrx3
ENSMUST00000067545.7	0.095144914	MGI:2684937	Lclat1
ENSMUST00000070673.8	0.095144914	MGI:1914603	Rab31
ENSMUST00000071325.8	0.095144914	MGI:97306	Nf1
ENSMUST00000074053.5	0.095144914	MGI:3704317	Sap18b
ENSMUST00000077115.12	0.095144914	MGI:1097152	Ptpro
ENSMUST00000085913.10	0.095144914	MGI:88106	Atp1a2
ENSMUST00000091920.5	0.095144914	MGI:2147194	Rbm27
ENSMUST00000092170.6	0.095144914	MGI:1914476	Tmem19
ENSMUST00000093943.9	0.095144914	MGI:105369	Cbx1
ENSMUST00000098346.4	0.095144914	MGI:2150656	Man2a2
ENSMUST00000100024.2	0.095144914	MGI:109183	Fgf12
ENSMUST00000100203.9	0.095144914	MGI:2145977	Dip2b
ENSMUST00000103197.4	0.095144914	MGI:1921285	Nol9
ENSMUST00000107283.7	0.095144914	MGI:1923992	Snx27
ENSMUST00000108249.8	0.095144914	MGI:99260	Prkci
ENSMUST00000111108.9	0.095144914	MGI:109284	Psen2
ENSMUST00000115118.7	0.095144914	MGI:1919834	Cul4b
ENSMUST00000116563.7	0.095144914	MGI:107953	Klc2
ENSMUST00000119944.7	0.095144914	MGI:3580376	Lemd3
ENSMUST00000143278.7	0.095144914	MGI:1923406	Fam131b
ENSMUST00000151390.7	0.095144914	MGI:108247	Tdg
ENSMUST00000159584.2	0.095144914	MGI:1920464	Nwd2
ENSMUST00000173078.7	0.095144914	MGI:1098801	Otud4
ENSMUST00000182078.8	0.095144914	MGI:88025	Ank2

ENSMUST00000219263.1	0.095144914	MGI:1309528	Ppp1r12a
ENSMUST00000226810.1	0.095144914	MGI:1922667	Rspo2
ENSMUST00000005493.13	0.096019926	MGI:99917	Slc1a3
ENSMUST00000034859.14	0.096019926	MGI:1926014	Fbxo22
ENSMUST00000037115.8	0.096019926	MGI:1917040	Med30
ENSMUST00000040881.13	0.096019926	MGI:1924029	Cluap1
ENSMUST00000070330.13	0.096019926	MGI:1341299	Dnm3
ENSMUST00000114200.9	0.096019926	MGI:1925573	Fam219b
ENSMUST00000030207.14	0.096260288	MGI:2142116	Psip1
ENSMUST00000034883.6	0.096260288	MGI:1916356	Stoml1
ENSMUST00000166855.2	0.096260288	MGI:2180756	Mchr1
ENSMUST00000191601.6	0.098063747	MGI:107765	Apbb1
ENSMUST00000079949.12	0.098855676	MGI:99960	Ewsr1
ENSMUST00000190827.6	0.099729136	MGI:1343180	Vgf
ENSMUST00000046288.15	0.100552352	MGI:1890894	Ndufv3
ENSMUST00000020343.8	0.101776786	MGI:894308	Rab21
ENSMUST00000031034.11	0.101776786	MGI:2183436	Nrbp1
ENSMUST00000031739.5	0.101776786	MGI:1922853	Ppp1r35
ENSMUST00000045846.11	0.101776786	MGI:2137681	Sfxn5
ENSMUST00000056732.3	0.101776786	MGI:3694697	Mfap1b
ENSMUST00000077693.9	0.101776786	MGI:95300	Eif2b4
ENSMUST00000099525.4	0.101776786	MGI:2683212	Ranbp6
ENSMUST00000166750.8	0.101776786	MGI:1921690	Cmip
ENSMUST00000024762.2	0.104041557	MGI:1922814	Rsph9
ENSMUST00000046533.8	0.104041557	MGI:3487246	Prr7
ENSMUST00000026387.10	0.105184377	MGI:1913961	Sbds
ENSMUST00000169681.2	0.105184377	MGI:95304	Eif4b
ENSMUST00000021802.15	0.105873445	MGI:1914502	Cap2
ENSMUST00000005406.11	0.106681691	MGI:88059	App
ENSMUST00000058438.8	0.108965898	MGI:1919083	Dcaf7
ENSMUST00000119430.2	0.109165479	MGI:1914880	Samd8
ENSMUST00000102598.3	0.109547894	MGI:1194912	Rbbp4
ENSMUST00000020109.4	0.110083023	MGI:1914269	Actr6
ENSMUST00000022378.8	0.110083023	MGI:1354385	Ero1l
ENSMUST00000032738.6	0.110083023	MGI:99779	Chrna7
ENSMUST00000048774.12	0.110083023	MGI:1858683	Copg2
ENSMUST00000067101.9	0.110083023	MGI:104742	Kcnj3
ENSMUST00000068911.12	0.110083023	MGI:95624	Gabrg3
ENSMUST00000088373.10	0.110083023	MGI:2152938	Srgap3

ENSMUST00000102483.4	0.110083023	MGI:3040672	Rpl23a
ENSMUST00000108297.2	0.110083023	MGI:2685870	Pdp1
ENSMUST00000154330.1	0.110083023	MGI:1928142	Mrps24
ENSMUST00000169861.8	0.110083023	MGI:1927197	Shoc2
ENSMUST00000182660.7	0.110083023	MGI:1913754	Prrc2c
ENSMUST00000220509.1	0.110083023	MGI:1349475	Ppp2r5c
ENSMUST00000014913.10	0.110732809	MGI:104884	Psmb1
ENSMUST00000006764.8	0.110917849	MGI:88061	Aprt
ENSMUST00000028981.8	0.11205375	MGI:891995	Mapre1
ENSMUST00000078084.6	0.112198851	MGI:106498	Rsrp1
ENSMUST00000014990.12	0.114347356	MGI:1915221	Tppp3
ENSMUST00000020741.11	0.114347356	MGI:1343297	Drg1
ENSMUST00000030942.12	0.114347356	MGI:2137221	Mrpl20
ENSMUST00000072726.6	0.114347356	MGI:1860197	Wdr7
ENSMUST00000006235.8	0.114641334	MGI:88561	Ctsb
ENSMUST00000068259.9	0.114728152	MGI:1924038	Klhdc10
ENSMUST00000023749.14	0.115930755	MGI:99682	Tmbim6
ENSMUST00000032373.11	0.116049678	MGI:96763	Ldhb
ENSMUST00000187839.7	0.116515814	MGI:109153	Ktn1
ENSMUST00000000925.9	0.116823684	MGI:1328366	Smarcb1
ENSMUST00000007747.9	0.116823684	MGI:2147092	Dus3l
ENSMUST00000019614.12	0.116823684	MGI:1914689	Xab2
ENSMUST00000021368.9	0.116823684	MGI:1918305	Nemf
ENSMUST00000045312.5	0.116823684	MGI:1344345	Smc1a
ENSMUST00000052566.7	0.116823684	MGI:2144113	Tmem199
ENSMUST00000059042.13	0.116823684	MGI:1202722	Gtf2i
ENSMUST00000103208.1	0.116823684	MGI:1196277	Tmem201
ENSMUST00000179163.2	0.117606118	MGI:1929514	Mlst8
ENSMUST00000110082.10	0.119452332	MGI:88251	Calm1
ENSMUST00000054908.9	0.119963459	MGI:1915205	Sugt1
ENSMUST00000163230.7	0.119963459	MGI:1196990	Cd200
ENSMUST00000030513.12	0.120661792	MGI:1917152	Mrto4
ENSMUST00000087600.9	0.120661792	MGI:95678	Gda
ENSMUST00000053413.11	0.122237729	MGI:101764	Cdk5r1
ENSMUST00000019937.4	0.123113995	MGI:2155302	Sec63
ENSMUST00000021239.6	0.123113995	MGI:2138133	Lrrc59
ENSMUST00000025193.13	0.123113995	MGI:99495	Brd2
ENSMUST00000033775.8	0.123113995	MGI:105941	Mpp1
ENSMUST00000033899.13	0.123113995	MGI:88455	Col4a2

ENSMUST00000036299.13	0.123113995	MGI:2135957	Camta2
ENSMUST00000045396.8	0.123113995	MGI:1918867	Armh3
ENSMUST00000046011.11	0.123113995	MGI:2684913	Nol10
ENSMUST00000054145.7	0.123113995	MGI:2444785	Dcaf5
ENSMUST00000066208.12	0.123113995	MGI:108186	Aldh7a1
ENSMUST00000067414.12	0.123113995	MGI:2183683	Nav1
ENSMUST00000072735.8	0.123113995	MGI:2384888	Fam173a
ENSMUST00000075845.10	0.123113995	MGI:1929494	Arhgap35
ENSMUST00000088652.5	0.123113995	MGI:1919709	Htatsf1
ENSMUST00000099194.3	0.123113995	MGI:2139328	Tspyl3
ENSMUST00000105561.8	0.123113995	MGI:1351338	Grm1
ENSMUST00000108593.7	0.123113995	MGI:1914431	Ctdnep1
ENSMUST00000134077.1	0.123113995	MGI:5141986	Gm20521
ENSMUST00000152796.7	0.123113995	MGI:2444233	Ythdf2
ENSMUST00000156859.2	0.123113995	MGI:2135593	Hadha
ENSMUST00000239079.1	0.123113995	MGI:1919375	Amer2
ENSMUST00000142247.7	0.123113995	NA	NA
ENSMUST00000021381.5	0.123139996	MGI:1100514	Pnn
ENSMUST00000114276.2	0.123139996	MGI:1349765	Rcn2
ENSMUST00000115559.9	0.123139996	MGI:107743	Dync1i1
ENSMUST00000037918.11	0.123145796	MGI:1915128	Tmem33
ENSMUST00000165649.3	0.125484391	MGI:1913342	Ghitm
ENSMUST00000062264.7	0.125967802	MGI:1934811	Nucks1
ENSMUST00000166497.8	0.126151367	MGI:1913332	Abhd6
ENSMUST00000002172.13	0.126885279	MGI:87882	Acp2
ENSMUST00000021203.6	0.126885279	MGI:1929742	Timm22
ENSMUST00000026274.13	0.126885279	MGI:1934860	Lztfl1
ENSMUST00000035871.14	0.126885279	MGI:1916720	Tmem127
ENSMUST00000038407.5	0.126885279	MGI:1914807	Larp6
ENSMUST00000050785.13	0.126885279	MGI:96757	Lcn2
ENSMUST00000078717.6	0.126885279	MGI:2443542	Lrrc58
ENSMUST00000171975.7	0.126885279	MGI:1344363	Arih1
ENSMUST00000134214.2	0.128004826	MGI:6155101	Gm49450
ENSMUST00000001452.13	0.128798354	MGI:104708	Cct3
ENSMUST00000034929.6	0.128798354	MGI:1933395	Lactb
ENSMUST00000091967.12	0.128798354	MGI:108092	Bin1
ENSMUST00000030914.3	0.129070862	MGI:1915080	Rer1
ENSMUST00000057829.3	0.129955131	MGI:107329	Mrpl50
ENSMUST00000148665.7	0.129955131	MGI:104912	Sf3a2

ENSMUST00000111336.9	0.135710782	MGI:1913302	Sdhc
ENSMUST00000017610.9	0.138554865	MGI:98753	Timp2
ENSMUST00000027587.14	0.138554865	MGI:1920199	Ccnt2
ENSMUST00000033634.4	0.138554865	MGI:1354713	Acsl4
ENSMUST00000173804.7	0.138554865	MGI:2442071	Gprasp2
ENSMUST00000031072.13	0.139678937	MGI:1098673	Anapc4
ENSMUST00000041023.13	0.139678937	MGI:1918944	Ipo9
ENSMUST00000168115.7	0.139678937	MGI:2384838	Prpsap2
ENSMUST00000228307.1	0.139678937	MGI:107433	Cdh9
ENSMUST00000002572.5	0.141711956	MGI:1890662	Slc9a3r2
ENSMUST00000006854.12	0.141711956	MGI:1918722	Usp19
ENSMUST00000021133.15	0.141711956	MGI:1917447	Srp68
ENSMUST00000028004.10	0.141711956	MGI:1861622	Aldh9a1
ENSMUST00000032183.5	0.141711956	MGI:1921372	Tmem43
ENSMUST00000036952.4	0.141711956	MGI:2654705	9530068E07Rik
ENSMUST00000038361.10	0.141711956	MGI:894701	Mgat5
ENSMUST00000040481.3	0.141711956	MGI:2679005	Slc38a7
ENSMUST00000102919.3	0.141711956	MGI:99606	Abca2
ENSMUST00000105907.8	0.141711956	MGI:1098568	Tmem222
ENSMUST00000192314.1	0.141711956	MGI:1922434	Camsap2
ENSMUST00000208253.1	0.141711956	MGI:1922863	Med25
ENSMUST00000225805.1	0.141711956	MGI:1919094	Nupl1
ENSMUST00000227729.1	0.141711956	MGI:2674071	Morn2
ENSMUST00000228050.1	0.141711956	MGI:1921409	Acbd5
ENSMUST00000023210.7	0.142482649	MGI:1913695	Cyc1
ENSMUST00000044564.14	0.143170945	MGI:2443432	Foxj3
ENSMUST00000007216.8	0.146089205	MGI:1298405	Ap2m1
ENSMUST00000000314.12	0.146504712	MGI:99218	Cdh4
ENSMUST00000003599.8	0.146504712	MGI:1935197	Pcdhgb6
ENSMUST00000004076.4	0.146504712	MGI:1351340	Grm3
ENSMUST00000008094.9	0.146504712	MGI:1918946	Abhd8
ENSMUST00000008445.6	0.146504712	MGI:1891839	Phax
ENSMUST00000015511.14	0.146504712	MGI:2154244	Plxnd1
ENSMUST00000020182.15	0.146504712	MGI:1321392	Pex7
ENSMUST00000020846.7	0.146504712	MGI:107606	Srebf1
ENSMUST00000021803.9	0.146504712	MGI:2385621	Nup153
ENSMUST00000023393.14	0.146504712	MGI:1921435	Gbe1
ENSMUST00000024829.7	0.146504712	MGI:107704	Abcg1
ENSMUST00000025279.5	0.146504712	MGI:1097712	Npc1

ENSMUST00000025338.15	0.146504712	MGI:1860139	Gabbr1
ENSMUST00000026252.13	0.146504712	MGI:894762	Ldb1
ENSMUST00000026292.14	0.146504712	MGI:1926884	Huwe1
ENSMUST00000027888.12	0.146504712	MGI:87860	Abl2
ENSMUST00000027961.11	0.146504712	MGI:1354164	Hspa14
ENSMUST00000029964.11	0.146504712	MGI:95276	Epha7
ENSMUST00000031091.12	0.146504712	MGI:1261849	D5Ertd579e
ENSMUST00000031446.6	0.146504712	MGI:3609245	Tmem132b
ENSMUST00000032629.15	0.146504712	MGI:1338801	Cyfip1
ENSMUST00000034369.9	0.146504712	MGI:1096380	Psmb10
ENSMUST00000034644.9	0.146504712	MGI:1918982	Vps11
ENSMUST00000036267.7	0.146504712	MGI:1891160	Chst2
ENSMUST00000037609.7	0.146504712	MGI:1196297	Hgsnat
ENSMUST00000038488.16	0.146504712	MGI:2446134	Sidt2
ENSMUST00000039152.13	0.146504712	MGI:1306823	Dhx8
ENSMUST00000040025.13	0.146504712	MGI:1917799	Tln2
ENSMUST00000041097.12	0.146504712	MGI:1289252	Ppp2r2d
ENSMUST00000041500.7	0.146504712	MGI:104971	Srxn1
ENSMUST00000045682.6	0.146504712	MGI:1925037	Gramd1b
ENSMUST00000045705.13	0.146504712	MGI:2443564	Ambra1
ENSMUST00000046316.10	0.146504712	MGI:2443718	Lrig2
ENSMUST00000046755.13	0.146504712	MGI:105056	Cobl
ENSMUST00000047243.11	0.146504712	MGI:109338	Rap1gap
ENSMUST00000047273.2	0.146504712	MGI:1919186	Rpusd1
ENSMUST00000048260.14	0.146504712	MGI:1917611	Lman1
ENSMUST00000053306.7	0.146504712	MGI:109147	Oxtr
ENSMUST00000053871.4	0.146504712	MGI:2444926	Ckap4
ENSMUST00000054598.11	0.146504712	MGI:2385758	Ablim2
ENSMUST00000055352.7	0.146504712	MGI:1914794	Fam120b
ENSMUST00000055485.11	0.146504712	MGI:3040677	Lsm14b
ENSMUST00000056355.8	0.146504712	MGI:2447776	Nat8l
ENSMUST00000057015.7	0.146504712	MGI:2443878	Slc4a7
ENSMUST00000057195.16	0.146504712	MGI:1351500	Nup62
ENSMUST00000060579.9	0.146504712	MGI:2384966	Mgat2
ENSMUST00000060837.9	0.146504712	MGI:1098586	Rab11fip5
ENSMUST00000066041.11	0.146504712	MGI:3605641	Shisa7
ENSMUST00000066760.7	0.146504712	MGI:2158736	Senp3
ENSMUST00000074352.10	0.146504712	MGI:1927551	Osbpl1a
ENSMUST00000075068.13	0.146504712	MGI:97542	Pepd

ENSMUST00000075117.9	0.146504712	MGI:1933108	Zxdc
ENSMUST00000075666.7	0.146504712	MGI:107995	Upf1
ENSMUST00000076226.12	0.146504712	MGI:103234	Herc2
ENSMUST00000077569.10	0.146504712	MGI:1935127	Smarca1
ENSMUST00000078482.12	0.146504712	MGI:2685011	Dchs1
ENSMUST00000080812.13	0.146504712	MGI:1916023	Zc3hc1
ENSMUST00000081121.3	0.146504712	MGI:2137513	Slc24a3
ENSMUST00000100539.9	0.146504712	MGI:1926079	Tsc22d4
ENSMUST00000103176.9	0.146504712	MGI:2679684	Mib2
ENSMUST00000105307.7	0.146504712	MGI:1921257	Btbd11
ENSMUST00000105327.9	0.146504712	MGI:1298224	Pip5k1c
ENSMUST00000105473.2	0.146504712	MGI:2139810	Slc35f1
ENSMUST00000105916.7	0.146504712	MGI:2444218	Ahdc1
ENSMUST00000106653.3	0.146504712	MGI:1922246	Usp47
ENSMUST00000108789.8	0.146504712	MGI:2685478	Zfp512b
ENSMUST00000109583.8	0.146504712	MGI:1926021	Mctp1
ENSMUST00000109699.10	0.146504712	MGI:107252	Nsun2
ENSMUST00000110778.1	0.146504712	MGI:1923831	Wipi2
ENSMUST00000112046.1	0.146504712	MGI:3029290	Zfp281
ENSMUST00000113264.8	0.146504712	MGI:1095737	Cntn4
ENSMUST00000119481.1	0.146504712	MGI:2144814	Lrfn5
ENSMUST00000119581.6	0.146504712	MGI:2685683	Thsd7a
ENSMUST00000121205.7	0.146504712	MGI:99259	Matk
ENSMUST00000121285.7	0.146504712	MGI:1888520	Brd4
ENSMUST00000126865.7	0.146504712	MGI:2446296	Sik3
ENSMUST00000133298.7	0.146504712	MGI:2443181	Dlgap2
ENSMUST00000140012.7	0.146504712	MGI:2429955	Pgbd5
ENSMUST00000149749.2	0.146504712	MGI:1918767	Vps35l
ENSMUST00000161515.7	0.146504712	MGI:106580	Cnot11
ENSMUST00000162671.7	0.146504712	MGI:2183747	Fgd4
ENSMUST00000163643.2	0.146504712	MGI:104582	Eps15l1
ENSMUST00000169061.7	0.146504712	MGI:1261425	Drosha
ENSMUST00000172132.8	0.146504712	MGI:98797	Tpi1
ENSMUST00000176438.8	0.146504712	MGI:1344407	Zeb2
ENSMUST00000179474.8	0.146504712	MGI:1921599	Fam160a2
ENSMUST00000182222.7	0.146504712	MGI:1932376	Cacng8
ENSMUST00000183703.7	0.146504712	MGI:2151013	Erc1
ENSMUST00000184945.7	0.146504712	MGI:2183691	Nav2
ENSMUST00000185333.1	0.146504712	MGI:96663	Kcna6

ENSMUST00000191602.1	0.146504712	MGI:2685842	Ajm1
ENSMUST00000194598.5	0.146504712	MGI:3039785	Syngap1
ENSMUST00000197928.4	0.146504712	MGI:1920081	Dhx30
ENSMUST00000210369.1	0.146504712	MGI:1924150	Ssbp4
ENSMUST00000235343.1	0.146504712	MGI:1933754	Nedd4l
ENSMUST00000000129.13	0.146504712	MGI:105917	Fer
ENSMUST00000002473.9	0.146504712	MGI:1915501	Babam1
ENSMUST00000002533.14	0.146504712	MGI:1929866	Rgs20
ENSMUST00000019441.8	0.146504712	MGI:1915092	Nop9
ENSMUST00000028170.14	0.146504712	MGI:107485	Ralgds
ENSMUST00000034234.14	0.146504712	MGI:1915164	Coq9
ENSMUST00000047144.12	0.146504712	MGI:2444899	5031439G07Rik
ENSMUST00000049932.11	0.146504712	MGI:88494	Creb1
ENSMUST00000057110.10	0.146504712	MGI:3704486	Eif3j2
ENSMUST00000065574.8	0.146504712	MGI:1915884	Tm2d3
ENSMUST00000078694.12	0.146504712	MGI:94860	Ppp1r1b
ENSMUST00000102514.3	0.146504712	MGI:2661375	Rtn4rl1
ENSMUST00000110531.8	0.146504712	MGI:3613669	Shf
ENSMUST00000113707.8	0.146504712	MGI:98809	Tpm1
ENSMUST00000164375.3	0.146504712	MGI:103072	Zyx
ENSMUST00000217537.1	0.146504712	MGI:1914621	Gtf3c6
ENSMUST00000020145.11	0.146504712	MGI:1340062	Sgk1
ENSMUST00000020188.12	0.146504712	MGI:107655	Ifngr1
ENSMUST00000021413.8	0.146504712	MGI:104741	Nfkbia
ENSMUST00000023365.12	0.146504712	MGI:1914368	Bfar
ENSMUST00000028300.5	0.146504712	MGI:1915241	Nacc2
ENSMUST00000029562.4	0.146504712	MGI:87891	Chrnb2
ENSMUST00000031273.8	0.146504712	MGI:1921846	Cds1
ENSMUST00000032926.11	0.146504712	MGI:1915992	Tmem219
ENSMUST00000033545.5	0.146504712	MGI:1915040	Rab39b
ENSMUST00000033671.12	0.146504712	MGI:104557	Rps6ka3
ENSMUST00000035497.4	0.146504712	MGI:1916308	Oscp1
ENSMUST00000037383.12	0.146504712	MGI:1916082	Ldah
ENSMUST00000040972.3	0.146504712	MGI:2145443	Utp15
ENSMUST00000042121.10	0.146504712	MGI:95921	H2-DMa
ENSMUST00000043336.10	0.146504712	MGI:106018	St8sia4
ENSMUST00000049470.10	0.146504712	MGI:1914729	Tmem183a
ENSMUST00000062289.10	0.146504712	MGI:2444572	Bend6
ENSMUST00000068025.12	0.146504712	MGI:2143315	Klhl18

ENSMUST00000072695.12	0.146504712	MGI:104636	Tle1
ENSMUST00000073787.6	0.146504712	MGI:107796	Akr7a5
ENSMUST00000088922.4	0.146504712	MGI:1915955	Gtf2f2
ENSMUST00000098367.4	0.146504712	MGI:1928485	Mlycd
ENSMUST00000115659.5	0.146504712	MGI:2447322	Pcdha9
ENSMUST00000117399.1	0.146504712	MGI:97167	Msn
ENSMUST00000132032.7	0.146504712	MGI:1298230	Cnot7
ENSMUST00000154256.2	0.146504712	MGI:2662729	Zfp703
ENSMUST00000166968.8	0.146504712	MGI:1097165	Nrg3
ENSMUST00000168292.1	0.146504712	MGI:5313030	Gm20721
ENSMUST00000168828.2	0.146504712	MGI:1352495	Zfp385a
ENSMUST00000176155.1	0.146504712	MGI:1919435	Dbndd1
ENSMUST00000188842.6	0.146504712	MGI:99666	Syt2
ENSMUST00000210682.1	0.146504712	MGI:109404	Nup98
ENSMUST00000218184.1	0.146504712	MGI:1918814	Izumo4
ENSMUST00000229028.1	0.146504712	MGI:98304	St3gal1
ENSMUST00000025411.8	0.146657476	MGI:2442865	Prelid3a
ENSMUST00000028905.9	0.146657476	MGI:97512	Pcsk2
ENSMUST00000035157.9	0.146657476	MGI:102964	Srprb
ENSMUST00000089726.9	0.146657476	MGI:2181743	D430041D05Rik
ENSMUST00000067284.9	0.14833267	MGI:1859328	Cpsf3
ENSMUST00000045593.11	0.149746005	MGI:2442032	Daglb
ENSMUST00000060490.10	0.149746005	MGI:1347345	Homer1
ENSMUST00000166247.7	0.149746005	MGI:1916348	Tmem176b
ENSMUST00000035371.8	0.150503808	MGI:1927139	Sv2a
ENSMUST00000044857.3	0.150503808	MGI:1913932	Trappc5
ENSMUST00000090150.10	0.150503808	MGI:1196326	Ncald
ENSMUST00000032143.7	0.151242217	MGI:98084	Rpn1
ENSMUST00000114897.8	0.151997873	MGI:1261437	Atp5c1
ENSMUST00000058793.13	0.152650375	MGI:1921076	Poldip3
ENSMUST00000015467.8	0.1529402	MGI:1353474	Slc39a1
ENSMUST00000001806.9	0.153493975	MGI:1918993	Coasy
ENSMUST00000019231.11	0.153493975	MGI:109629	Atp6ap1
ENSMUST00000045286.8	0.153493975	MGI:1915187	Tmem59l
ENSMUST00000092430.10	0.153493975	MGI:107810	Tfam
ENSMUST00000143987.8	0.153817507	MGI:1920150	Ndufv2
ENSMUST00000008350.15	0.154015838	MGI:1914510	Cers4
ENSMUST00000024897.9	0.154015838	MGI:1353561	Vapa
ENSMUST00000033241.5	0.154015838	MGI:1923679	Lhpp

ENSMUST00000020820.1	0.154973749	MGI:1333794	Mrpl22
ENSMUST00000030036.5	0.154973749	MGI:1928478	Brinp1
ENSMUST00000032500.8	0.154973749	MGI:3043083	Prmt8
ENSMUST00000041769.7	0.154973749	MGI:107738	Dync1li2
ENSMUST00000050561.12	0.154973749	MGI:1921799	Mau2
ENSMUST00000054514.5	0.154973749	MGI:2669796	Rtn4rl2
ENSMUST00000066905.8	0.154973749	MGI:1098266	Chl1
ENSMUST00000113476.7	0.154973749	MGI:1333849	Rasgrp2
ENSMUST00000152367.7	0.154973749	MGI:104592	Myef2
ENSMUST00000002121.4	0.157874366	MGI:107726	Supt6
ENSMUST00000006424.7	0.157874366	MGI:1915723	Mob1b
ENSMUST00000016023.8	0.157874366	MGI:2442958	Fam184b
ENSMUST00000021187.11	0.157874366	MGI:1917701	Dhrs13
ENSMUST00000043757.14	0.157874366	MGI:1351658	Abcf1
ENSMUST00000050646.12	0.157874366	MGI:1920045	Ttc19
ENSMUST00000100347.10	0.158682708	MGI:97180	Mapt
ENSMUST00000025957.8	0.159339681	MGI:1915144	Fam45a
ENSMUST00000033506.12	0.159339681	MGI:1914661	Wdr13
ENSMUST00000031726.14	0.159834924	MGI:95784	Gnb2
ENSMUST00000020238.13	0.160223731	MGI:98817	Hsp90b1
ENSMUST00000044385.13	0.164395469	MGI:2444615	Sgtb
ENSMUST00000061755.8	0.164574964	MGI:96748	Lamp2
ENSMUST00000102960.10	0.164574964	MGI:1329014	Stam
ENSMUST00000160307.8	0.164574964	MGI:1916264	Tnik
ENSMUST00000014684.5	0.164908221	MGI:98884	U2af1
ENSMUST00000028386.11	0.164908221	MGI:1355333	Nckap1
ENSMUST00000032995.14	0.164908221	MGI:99473	Arrb1
ENSMUST00000042701.12	0.164908221	MGI:104991	Mxd4
ENSMUST00000063838.10	0.164908221	MGI:1923520	Fam49b
ENSMUST00000098112.8	0.164908221	MGI:1930018	Dnajb5
ENSMUST00000105525.11	0.164908221	MGI:87971	Ahi1
ENSMUST00000107384.9	0.164908221	MGI:96414	Idh2
ENSMUST00000001780.9	0.165740856	MGI:87986	Akt1
ENSMUST00000010241.13	0.165740856	MGI:1858330	Nxf1
ENSMUST00000012152.12	0.165740856	MGI:892866	Dgcr2
ENSMUST00000019333.9	0.165740856	MGI:1921565	Rnf145
ENSMUST00000024599.13	0.165740856	MGI:96435	Igf2r
ENSMUST00000026439.13	0.165740856	MGI:1917167	Nabp2
ENSMUST00000033783.1	0.165740856	MGI:1923939	Tceal6

ENSMUST00000037763.10	0.165740856	MGI:2448561	Ythdc2
ENSMUST00000044795.7	0.165740856	MGI:2442620	Nup133
ENSMUST00000049057.4	0.165740856	MGI:2448496	Fam171a2
ENSMUST00000053681.5	0.165740856	MGI:2442704	Frrs1l
ENSMUST00000054636.6	0.165740856	MGI:1917619	Bag5
ENSMUST00000061174.6	0.165740856	MGI:2136419	Sarm1
ENSMUST00000074225.10	0.165740856	MGI:1334445	Cpne6
ENSMUST00000081551.13	0.165740856	MGI:1196624	Tcea1
ENSMUST00000090057.5	0.165740856	MGI:2442392	Sybu
ENSMUST00000105897.9	0.165740856	MGI:1935147	Arid1a
ENSMUST00000107152.8	0.165740856	MGI:1333784	Hdac5
ENSMUST00000107270.8	0.165740856	MGI:2442117	Pogz
ENSMUST00000111466.2	0.165740856	MGI:1914695	C1qtnf4
ENSMUST00000114444.8	0.165740856	MGI:1923552	Pcnp
ENSMUST00000117944.1	0.165740856	MGI:1201606	Hs3st1
ENSMUST00000129411.6	0.165740856	MGI:5753810	Gm45234
ENSMUST00000130216.2	0.165740856	MGI:98285	Srsf3
ENSMUST00000155551.7	0.165740856	MGI:2140179	Dcaf10
ENSMUST00000100484.5	0.16620713	MGI:1933181	Eif3d
ENSMUST00000026624.10	0.167150032	MGI:1915309	Tm9sf2
ENSMUST00000025014.9	0.167188016	MGI:1915861	Mrpl28
ENSMUST00000021362.4	0.167298132	MGI:1916804	Klhdc2
ENSMUST00000095797.5	0.168213511	MGI:109242	Spin1
ENSMUST00000058845.8	0.168511748	MGI:1917600	Basp1
ENSMUST00000150350.8	0.16867265	MGI:1351455	Rpl13a
ENSMUST00000170217.7	0.16932876	MGI:1346035	Farsb
ENSMUST00000022268.9	0.170193666	MGI:1915513	Pdhb
ENSMUST00000032272.12	0.170193666	MGI:93830	Adipor2
ENSMUST00000131728.3	0.170193666	MGI:894684	Pa2g4
ENSMUST00000031038.10	0.170928525	MGI:1915345	Ociad1
ENSMUST00000007131.15	0.170935542	MGI:103251	Acly
ENSMUST00000020488.8	0.171197507	MGI:106381	D10Wsu102e
ENSMUST00000037785.13	0.172628176	MGI:97282	Ncam2
ENSMUST00000072954.7	0.172628176	MGI:1919085	Lancl2
ENSMUST00000072961.5	0.172628176	MGI:1929215	Vps41
ENSMUST00000098048.5	0.172628176	MGI:1923686	Tufm
ENSMUST00000102687.3	0.172628176	MGI:1913483	Dmap1
ENSMUST00000166664.1	0.172628176	MGI:5141963	Gm20498
ENSMUST00000167004.2	0.172628176	MGI:2142716	Gpat4

ENSMUST00000216344.1	0.172628176	MGI:107877	Prkcsh
ENSMUST00000002043.9	0.172701578	MGI:1914413	Ccdc47
ENSMUST00000006181.6	0.174165802	MGI:104563	Napa
ENSMUST00000028768.1	0.174972963	MGI:1916952	Ndufaf1
ENSMUST00000033662.8	0.174972963	MGI:97532	Pdha1
ENSMUST00000065167.8	0.174972963	MGI:87941	Grk3
ENSMUST00000108345.8	0.174972963	MGI:1918850	Ythdf3
ENSMUST00000181173.1	0.174972963	MGI:3782011	Gm3839
ENSMUST00000050027.8	0.175803402	MGI:109185	Ccn3
ENSMUST00000099224.9	0.176154769	MGI:88543	Csnk2a1
ENSMUST00000132846.1	0.177469966	MGI:97749	Ppia
ENSMUST00000021963.4	0.178054367	MGI:104728	Caml
ENSMUST00000026093.8	0.178054367	MGI:1933765	Btbd1
ENSMUST00000030243.7	0.178054367	MGI:1336173	Prkaa2
ENSMUST00000034464.7	0.178054367	MGI:1916801	2310022B05Rik
ENSMUST00000036999.9	0.178054367	MGI:1313136	Clip2
ENSMUST00000039608.8	0.178054367	MGI:1918480	Dym
ENSMUST00000043059.8	0.178054367	MGI:1306826	Sema7a
ENSMUST00000077853.4	0.178054367	MGI:109584	Prpf4b
ENSMUST00000092295.9	0.178054367	MGI:1333812	Mbd3
ENSMUST00000100206.3	0.178054367	MGI:2443114	Larp4
ENSMUST00000106259.8	0.178054367	MGI:95609	Gaa
ENSMUST00000114157.2	0.178054367	MGI:1100492	Nrp2
ENSMUST00000179343.2	0.178054367	MGI:1338779	Purb
ENSMUST00000024706.11	0.178138568	MGI:1351327	Pla2g7
ENSMUST00000032220.14	0.178799908	MGI:1349400	Cops7a
ENSMUST00000030455.14	0.179087905	MGI:1929955	Akr1a1
ENSMUST00000021049.8	0.179399726	MGI:105047	Psmc5
ENSMUST00000094179.10	0.182668029	MGI:95808	Gria1
ENSMUST00000135941.7	0.183096892	MGI:1276111	Pfdn2
ENSMUST00000021522.4	0.183846754	MGI:1920296	Glrx5
ENSMUST00000030412.10	0.183846754	MGI:1298204	Ppt1
ENSMUST00000075280.11	0.183846754	MGI:1913833	Exosc1
ENSMUST00000079791.10	0.183846754	MGI:88557	Pcyt1a
ENSMUST00000166984.7	0.183846754	MGI:1196294	Fubp1
ENSMUST00000100162.4	0.184888875	MGI:1929063	Copz1
ENSMUST00000006137.8	0.185811246	MGI:1915265	Trap1
ENSMUST00000021479.5	0.186612594	MGI:1891654	Actr10
ENSMUST00000029719.13	0.186612594	MGI:1918012	Dclk2

ENSMUST00000049460.10	0.186612594	MGI:95832	Grn
ENSMUST00000088429.7	0.186612594	MGI:1347353	Atp2b3
ENSMUST00000102488.7	0.186612594	MGI:2667185	Myo18a
ENSMUST00000114871.1	0.186612594	MGI:1354959	Hs6st2
ENSMUST00000143219.7	0.186612594	MGI:99887	Pitpna
ENSMUST00000162031.7	0.186612594	MGI:2138153	Txndc9
ENSMUST00000192168.1	0.186612594	MGI:1298371	Pcdha5
ENSMUST00000205658.1	0.186612594	MGI:2387643	B9d2
ENSMUST00000227843.1	0.186612594	MGI:1914679	Nudcd1
ENSMUST00000022849.6	0.186799935	MGI:106314	Tars
ENSMUST00000055966.12	0.188050593	MGI:95615	Gabra3
ENSMUST00000017430.11	0.189172155	MGI:1914451	Glod4
ENSMUST00000022147.14	0.191072101	MGI:109257	Smn1
ENSMUST00000023502.5	0.191072101	MGI:1916400	Snx4
ENSMUST00000102880.4	0.191072101	MGI:107172	Ppp3r1
ENSMUST00000005067.5	0.191698329	MGI:1098703	Sgta
ENSMUST00000028295.8	0.192906232	MGI:106559	Dnlz
ENSMUST00000032825.13	0.192906232	MGI:102768	Mfge8
ENSMUST00000064234.6	0.192906232	MGI:98931	Ezr
ENSMUST00000097886.3	0.192906232	MGI:3609248	5730409E04Rik
ENSMUST00000099466.9	0.192906232	MGI:700014	Sorbs1
ENSMUST00000031420.10	0.194653243	MGI:1289326	Gpn3
ENSMUST00000045986.7	0.194653243	MGI:103078	Spr
ENSMUST00000025918.8	0.194655866	MGI:109130	Stip1
ENSMUST00000027467.10	0.194997208	MGI:101780	Serpine2
ENSMUST00000193560.5	0.197118709	MGI:1923750	Ip6k2
ENSMUST00000023554.8	0.197729145	MGI:2387188	Slc49a4
ENSMUST00000042561.13	0.197729145	MGI:2442746	Slc43a2
ENSMUST00000063577.9	0.197729145	MGI:1927231	Zmym3
ENSMUST00000075954.8	0.197729145	MGI:109577	Taf1b
ENSMUST00000097694.10	0.197729145	MGI:2388278	Retreg2
ENSMUST00000100399.10	0.197729145	MGI:95606	Xrcc6
ENSMUST00000023572.14	0.198774164	MGI:1201679	Cxadr
ENSMUST00000033804.4	0.199572155	MGI:1914245	Zcchc18
ENSMUST00000000348.14	0.199602556	MGI:1913618	Rtca
ENSMUST00000165790.8	0.199602556	MGI:1337995	Klhl22
ENSMUST00000170303.1	0.199965802	MGI:5141855	Gm20390
ENSMUST00000001419.9	0.201542946	MGI:1913742	Zmat2
ENSMUST00000028139.10	0.201542946	MGI:1916225	Med27

ENSMUST00000029297.5	0.201542946	MGI:1347355	Slc7a11
ENSMUST00000053020.7	0.201542946	MGI:3643092	Neurl1b
ENSMUST00000061279.9	0.201542946	MGI:1935228	Pcdhga11
ENSMUST00000030841.9	0.201639851	MGI:1196453	Klhl7
ENSMUST00000066601.12	0.201639851	MGI:108030	Hyou1
ENSMUST00000031402.11	0.201710702	MGI:107943	Cct6a
ENSMUST00000034822.11	0.20295812	MGI:2385656	Acsbg1
ENSMUST00000045633.5	0.204634361	MGI:106181	Mybbp1a
ENSMUST00000046735.10	0.204634361	MGI:1096570	Tex264
ENSMUST00000055758.15	0.204634361	MGI:1861100	Ubr3
ENSMUST00000093157.12	0.205070225	MGI:101919	Ap1g1
ENSMUST00000071230.7	0.206527809	MGI:95299	Eif2s1
ENSMUST00000107851.9	0.207041051	MGI:894297	Clta
ENSMUST00000061508.7	0.207190882	MGI:2153740	Zfp358
ENSMUST00000075316.9	0.207190882	MGI:95809	Gria2
ENSMUST00000003521.9	0.208018621	MGI:1351329	Rps11
ENSMUST00000044681.6	0.208095738	MGI:1929501	Arl6ip5
ENSMUST00000167962.1	0.208308229	MGI:894284	Rab12
ENSMUST00000005830.14	0.209340285	MGI:1915433	Bcas2
ENSMUST00000032425.6	0.209340285	MGI:1913337	Emc3
ENSMUST00000053705.7	0.209340285	MGI:1919680	B4gat1
ENSMUST00000117786.7	0.210139255	MGI:1351660	Csnk1e
ENSMUST00000172450.2	0.211172648	MGI:108415	Pafah1b2
ENSMUST00000082090.14	0.212753299	MGI:1100869	Ap3b2
ENSMUST00000164181.1	0.213438934	MGI:109318	Myl6
ENSMUST00000138703.7	0.214130773	MGI:1891833	Pfkp
ENSMUST00000069747.5	0.215341879	MGI:1920274	Emc7
ENSMUST00000039720.10	0.216415041	MGI:3038613	Rragb
ENSMUST00000042726.13	0.216415041	MGI:1915285	Rbm42
ENSMUST00000004683.12	0.216903047	MGI:1890498	Mcoln1
ENSMUST00000006838.15	0.216903047	MGI:1915851	Qars
ENSMUST00000018466.3	0.216903047	MGI:1914395	Tomm34
ENSMUST00000019135.13	0.216903047	MGI:2384159	Gga3
ENSMUST00000020349.6	0.216903047	MGI:1346052	Apc2
ENSMUST00000026013.5	0.216903047	MGI:96915	Maoa
ENSMUST00000027271.8	0.216903047	MGI:104848	Inpp1
ENSMUST00000028554.3	0.216903047	MGI:2138993	Lpcat4
ENSMUST00000030143.12	0.216903047	MGI:1916176	Ubap2
ENSMUST00000030691.16	0.216903047	MGI:1349390	Pclo

ENSMUST00000033257.14	0.216903047	MGI:1919346	Eef1akmt2
ENSMUST00000034017.8	0.216903047	MGI:1924363	Klhl2
ENSMUST00000034328.12	0.216903047	MGI:1918135	Ints10
ENSMUST00000037739.7	0.216903047	MGI:1353651	Gnl3
ENSMUST00000041391.4	0.216903047	MGI:1920978	Psd
ENSMUST00000054591.9	0.216903047	MGI:2139347	Thnsl1
ENSMUST00000066778.5	0.216903047	MGI:1934031	Pi4k2a
ENSMUST00000070735.9	0.216903047	MGI:95623	Gabrg2
ENSMUST00000082088.9	0.216903047	MGI:3045303	Mamld1
ENSMUST00000090938.10	0.216903047	MGI:1929538	Dap3
ENSMUST00000091252.4	0.216903047	MGI:2139207	Sec16a
ENSMUST00000099678.4	0.216903047	MGI:1341907	Fjx1
ENSMUST00000100322.3	0.216903047	MGI:2679446	Slitrk1
ENSMUST00000101059.3	0.216903047	MGI:2444810	Prrt3
ENSMUST00000109221.8	0.216903047	MGI:1927169	B4galt5
ENSMUST00000109399.8	0.216903047	MGI:2681523	Tnpo1
ENSMUST00000109424.3	0.216903047	MGI:98962	Wnt7b
ENSMUST00000109670.7	0.216903047	MGI:1929915	Ncoa6
ENSMUST00000109962.3	0.216903047	MGI:105372	Sstr4
ENSMUST00000110030.9	0.216903047	MGI:1916428	Snx5
ENSMUST00000112683.8	0.216903047	MGI:1916749	Tsr2
ENSMUST00000113409.7	0.216903047	MGI:3045342	Zfp711
ENSMUST00000114472.7	0.216903047	MGI:1347357	Pnck
ENSMUST00000139560.7	0.216903047	MGI:1920037	Ndc1
ENSMUST00000159855.7	0.216903047	MGI:2179432	Mycbp2
ENSMUST00000166241.1	0.216903047	MGI:2679449	Slitrk2
ENSMUST00000170578.2	0.216903047	MGI:1916049	Rgmb
ENSMUST00000053811.9	0.218389616	MGI:1926170	Dlst
ENSMUST00000005234.12	0.219637503	MGI:1337100	Wdr1
ENSMUST00000005749.5	0.220263339	MGI:109345	Ctr9
ENSMUST00000016901.4	0.220263339	MGI:3039573	Ttll12
ENSMUST00000017576.10	0.220263339	MGI:106368	Rbfox3
ENSMUST00000021670.14	0.220263339	MGI:1926195	Ylpm1
ENSMUST00000027929.9	0.220263339	MGI:2664902	Mark1
ENSMUST00000032570.13	0.220263339	MGI:94907	Dmwd
ENSMUST00000039173.12	0.220263339	MGI:1922832	Relch
ENSMUST00000046765.9	0.220263339	MGI:109322	Kcnk1
ENSMUST00000059354.14	0.220263339	MGI:1195268	Sigmar1
ENSMUST00000064110.13	0.220263339	MGI:109446	Doc2a

ENSMUST00000067925.7	0.220263339	MGI:96157	Hmgb2
ENSMUST00000069637.14	0.220263339	MGI:1890393	Zfp277
ENSMUST00000085724.4	0.220263339	MGI:2138351	Susd4
ENSMUST00000085912.9	0.220263339	MGI:2154090	Igsf8
ENSMUST00000091554.5	0.220263339	MGI:3588199	Cntnap3
ENSMUST00000105372.7	0.220263339	MGI:104767	Gpx4
ENSMUST00000108594.7	0.220263339	MGI:1859017	Elp5
ENSMUST00000109462.7	0.220263339	MGI:97615	Plcg1
ENSMUST00000109726.7	0.220263339	MGI:2150037	Mta1
ENSMUST00000114321.1	0.220263339	MGI:2384811	Kbtbd2
ENSMUST00000149964.8	0.220263339	MGI:1309503	Kcnq2
ENSMUST00000150068.1	0.220263339	MGI:5141983	Gm20518
ENSMUST00000166791.7	0.220263339	MGI:1917483	Cd2bp2
ENSMUST00000099396.2	0.221480177	MGI:3513266	Nt5dc3
ENSMUST00000036380.13	0.221622496	MGI:1890510	Atp6v0b
ENSMUST00000005487.11	0.222529666	MGI:1929468	Txn2
ENSMUST00000027114.5	0.222529666	MGI:1915365	Maip1
ENSMUST00000030628.14	0.222529666	MGI:1913396	Maco1
ENSMUST00000031434.7	0.222529666	MGI:2153340	Rnf34
ENSMUST00000039442.11	0.222529666	MGI:1914039	Alg14
ENSMUST00000042942.9	0.222529666	MGI:2450915	Sec23ip
ENSMUST00000063663.5	0.222529666	MGI:2389490	B3gat2
ENSMUST00000099761.9	0.222529666	MGI:1261760	Lsamp
ENSMUST00000103027.9	0.222529666	MGI:3606200	Mgat5b
ENSMUST00000106052.1	0.222529666	MGI:3039601	Zfp941
ENSMUST00000109532.8	0.222529666	MGI:1918925	0610010F05Rik
ENSMUST00000114792.7	0.222529666	MGI:104670	Fkbp5
ENSMUST00000117551.3	0.222529666	MGI:1197013	Rasa3
ENSMUST00000234266.1	0.222863284	MGI:1889642	Elp2
ENSMUST00000110093.8	0.223130968	MGI:97843	Rab3a
ENSMUST00000042012.6	0.224161465	MGI:1289341	Sf3b3
ENSMUST00000062181.8	0.224161465	MGI:1347092	Zfp146
ENSMUST00000068110.9	0.224161465	MGI:1860487	Pcdh7
ENSMUST00000076364.5	0.224161465	MGI:3704336	Rpl10-ps3
ENSMUST00000079439.9	0.224161465	MGI:2443589	Tmem91
ENSMUST00000103221.9	0.224161465	MGI:1928394	Mtor
ENSMUST00000159899.7	0.224161465	MGI:1913863	Pigk
ENSMUST00000186465.6	0.224161465	MGI:1309481	Trip12
ENSMUST00000033313.2	0.224552323	MGI:1917745	Atp6ap2

ENSMUST00000023357.13	0.225392497	MGI:1913336	Fopnl
ENSMUST00000024802.9	0.225392497	MGI:1916066	Ppil1
ENSMUST00000025864.10	0.225392497	MGI:1915459	Rnaseh2c
ENSMUST00000030882.11	0.225392497	MGI:1920328	Pmpcb
ENSMUST00000172361.7	0.225392497	MGI:101947	Hnrnpd
ENSMUST00000002133.8	0.226354255	MGI:108019	Sdf2
ENSMUST00000019920.12	0.226354255	MGI:2443223	Clvs2
ENSMUST00000025019.8	0.226354255	MGI:108430	Arhgdig
ENSMUST00000043237.13	0.226354255	MGI:1915978	Trp53inp2
ENSMUST00000055438.4	0.226354255	MGI:2444096	Ppm1e
ENSMUST00000074233.11	0.226354255	MGI:2661187	Synm
ENSMUST00000110142.7	0.226354255	MGI:104898	Gpcpd1
ENSMUST00000012259.8	0.22712638	MGI:2137379	Med15
ENSMUST00000026665.7	0.22712638	MGI:1195985	Cbx4
ENSMUST00000028517.12	0.22712638	MGI:1914309	Ola1
ENSMUST00000169390.7	0.22712638	MGI:1915120	Enoph1
ENSMUST00000219037.1	0.22712638	MGI:1928477	Cnpy2
ENSMUST00000195015.5	0.227759926	MGI:1921173	Tmco1
ENSMUST00000075641.9	0.228289128	MGI:106184	Npm1
ENSMUST00000213683.1	0.228289128	MGI:1925112	Thyn1
ENSMUST00000020081.10	0.228768239	MGI:1289227	Zwint
ENSMUST00000098574.8	0.229338672	MGI:2385189	Rap1gds1
ENSMUST00000022204.15	0.230799317	MGI:108390	Kif2a
ENSMUST00000025961.6	0.230952271	MGI:88034	Prdx3
ENSMUST00000166468.1	0.230952271	MGI:103310	Ptprk
ENSMUST00000073791.9	0.231220167	MGI:1918929	Atp5h
ENSMUST00000019514.9	0.231381291	MGI:103249	Calm3
ENSMUST00000003117.14	0.231447075	MGI:102776	Ap1m1
ENSMUST00000022380.8	0.231886841	MGI:1914339	Psmc6
ENSMUST00000064762.5	0.233220393	MGI:1306778	Map1b
ENSMUST00000076694.12	0.235477772	MGI:1353498	Slc25a3
ENSMUST00000163507.7	0.239192295	MGI:97548	Pfkm
ENSMUST00000097014.6	0.240041539	MGI:98869	Tuba1a
ENSMUST00000115817.2	0.24006362	MGI:1913898	Tpgs2
ENSMUST00000000619.7	0.240503433	MGI:104571	Clcn4
ENSMUST00000045441.7	0.240503433	MGI:97828	Pygb
ENSMUST00000023694.10	0.24195722	MGI:1915251	Cfap298
ENSMUST00000168264.1	0.242332146	MGI:1914666	Armcx2
ENSMUST00000066587.11	0.242837421	MGI:1330812	Acox1

ENSMUST00000117611.7	0.243859782	MGI:1333782	Pld3
ENSMUST00000019939.11	0.244280152	MGI:1860188	Snx3
ENSMUST00000102590.10	0.244869815	MGI:1100836	Kpna6
ENSMUST00000004173.11	0.244921987	MGI:1913876	Cdip1
ENSMUST00000135884.7	0.245156163	MGI:97355	Nme1
ENSMUST00000023312.13	0.24565099	MGI:1313266	Alcam
ENSMUST00000043211.6	0.24648621	MGI:2684847	Coq10a
ENSMUST00000025713.11	0.247452686	MGI:1920416	Tm7sf2
ENSMUST00000043716.8	0.247452686	MGI:1914486	Cinp
ENSMUST00000097911.8	0.247452686	MGI:2446210	Kdm4a
ENSMUST00000020537.8	0.248531873	MGI:1202070	Nsg2
ENSMUST00000030192.4	0.248583999	MGI:1916167	Hint2
ENSMUST00000075895.8	0.248583999	MGI:1334458	Pex19
ENSMUST00000004494.15	0.24992625	MGI:107158	Sin3b
ENSMUST00000055294.3	0.24992625	MGI:1344367	Grem2
ENSMUST00000058994.5	0.24992625	MGI:2443503	Tram1l1
ENSMUST00000212824.1	0.24992625	MGI:1919055	Nup93
ENSMUST00000034203.16	0.251544501	MGI:2142808	Cog4
ENSMUST00000098845.9	0.251544501	MGI:1333763	Epn1
ENSMUST00000111080.7	0.251822003	MGI:1098244	Ap1s1
ENSMUST00000079644.12	0.253356489	MGI:99146	Ybx1
ENSMUST00000080537.13	0.253356489	MGI:97845	Rac1
ENSMUST00000051839.8	0.253533636	MGI:1920177	Hepacam
ENSMUST00000084238.4	0.253533636	MGI:3527792	Zdhhc18
ENSMUST00000091299.7	0.253533636	MGI:102956	Cdk7
ENSMUST00000111961.7	0.253533636	MGI:1917171	Hnrnpa3
ENSMUST00000017572.13	0.254338454	MGI:1916327	Psmd11
ENSMUST00000022293.13	0.2553821	MGI:106915	Vdac2
ENSMUST00000008826.13	0.256037666	MGI:105943	Rpl10
ENSMUST00000038608.13	0.256037666	MGI:1924832	Mboat7
ENSMUST00000060481.8	0.256037666	MGI:2444462	Dcaf12l1
ENSMUST00000077340.13	0.256037666	MGI:2384902	Rnpep
ENSMUST00000093995.9	0.256037666	MGI:104745	Sez6
ENSMUST00000204059.2	0.256037666	MGI:87919	Add2
ENSMUST00000057934.9	0.256812083	MGI:1914105	Tcf25
ENSMUST00000002808.6	0.257831161	MGI:1344375	Prkra
ENSMUST00000048967.8	0.258590989	MGI:101932	Cpe
ENSMUST00000102746.10	0.259279564	MGI:1858313	Uba2
ENSMUST00000001950.11	0.259286241	MGI:1891808	Tollip

ENSMUST00000010248.3	0.259286241	MGI:1914086	Tmem223
ENSMUST00000022718.10	0.259286241	MGI:102720	Ednrb
ENSMUST00000031032.10	0.259286241	MGI:106065	Ppm1g
ENSMUST00000054351.5	0.259286241	MGI:1915419	Ndnf
ENSMUST00000068892.14	0.259286241	MGI:1344416	Ash2l
ENSMUST00000072837.6	0.259286241	MGI:1347488	Foxk1
ENSMUST00000079423.6	0.259286241	MGI:1916202	Fam57b
ENSMUST00000125172.7	0.259286241	MGI:97572	Phb
ENSMUST00000001825.8	0.260287647	MGI:1914167	Chordc1
ENSMUST00000009789.14	0.260920125	MGI:97463	P4ha1
ENSMUST00000074694.6	0.260920125	MGI:95771	Gnai1
ENSMUST00000001079.14	0.263669439	MGI:2139764	Sec24b
ENSMUST00000027285.12	0.263669439	MGI:1914637	Unc50
ENSMUST00000037182.13	0.263669439	MGI:2443554	Hook3
ENSMUST00000041640.4	0.263669439	MGI:2144755	Ankmy2
ENSMUST00000044783.13	0.263669439	MGI:2384784	Eif4g1
ENSMUST00000045218.8	0.263669439	MGI:1277958	Pcm1
ENSMUST00000046389.4	0.263669439	MGI:2387367	Rbm45
ENSMUST00000046575.16	0.263669439	MGI:1933946	Ptov1
ENSMUST00000056433.6	0.263669439	MGI:2139054	Gpr107
ENSMUST00000057442.7	0.263669439	MGI:1915453	Diras2
ENSMUST00000098548.7	0.263669439	MGI:98247	Scn1b
ENSMUST00000106343.2	0.263669439	MGI:2141881	Ino80e
ENSMUST00000120613.8	0.263669439	MGI:107689	Kif3a
ENSMUST00000193734.5	0.263669439	MGI:2441730	Tbl1xr1
ENSMUST00000200169.5	0.263669439	MGI:1915022	Chd8
ENSMUST00000206720.1	0.263669439	MGI:88279	Cbl
ENSMUST00000034866.8	0.264937487	MGI:106092	Etfa
ENSMUST00000043313.14	0.264937487	MGI:2444155	Nmnat2
ENSMUST00000014065.15	0.265678368	MGI:1923936	Clip3
ENSMUST00000015829.14	0.266848453	MGI:1914135	Acadsb
ENSMUST00000028251.9	0.267435746	MGI:1915139	Rbm18
ENSMUST00000040440.6	0.267561104	MGI:103250	Calm2
ENSMUST00000021684.5	0.268577371	MGI:1341877	Cyp46a1
ENSMUST00000080723.10	0.268577371	MGI:1289308	Tax1bp1
ENSMUST00000000449.8	0.269907498	MGI:1914277	Mkrn2
ENSMUST00000020634.13	0.269907498	MGI:1346862	Mapk9
ENSMUST00000027706.3	0.269907498	MGI:106037	Lrrn2
ENSMUST00000028880.9	0.269907498	MGI:108392	Slc20a1

ENSMUST00000031388.12	0.269907498	MGI:1924823	Vps33a
ENSMUST00000033283.9	0.269907498	MGI:98180	Rrm1
ENSMUST00000034703.14	0.269907498	MGI:1913208	Carm1
ENSMUST00000035166.11	0.269907498	MGI:1913913	Uba5
ENSMUST00000042147.5	0.269907498	MGI:2140843	Noc4l
ENSMUST00000048116.14	0.269907498	MGI:88117	Slc7a1
ENSMUST00000049506.7	0.269907498	MGI:98885	Zrsr1
ENSMUST00000052204.5	0.269907498	MGI:2442058	Nipa1
ENSMUST00000059571.6	0.269907498	MGI:2136757	Pcdhb19
ENSMUST00000075269.9	0.269907498	MGI:2444672	Madd
ENSMUST00000076264.7	0.269907498	MGI:1917345	Zfp512
ENSMUST00000079496.8	0.269907498	MGI:1925141	Ube2s
ENSMUST00000114641.7	0.269907498	MGI:1914047	Cntnap2
ENSMUST00000122912.7	0.269907498	MGI:1925021	Csrnp3
ENSMUST00000144326.3	0.269907498	MGI:1928741	Ranbp9
ENSMUST00000171751.9	0.269907498	MGI:1933973	Rbfox2
ENSMUST00000239099.1	0.269907498	MGI:1915293	Eef1akmt1
ENSMUST00000009256.3	0.270375845	MGI:2136959	Bcl2l13
ENSMUST00000033450.2	0.270375845	MGI:104902	Gpc4
ENSMUST00000044009.13	0.270375845	MGI:2442190	Camk1d
ENSMUST00000040270.5	0.270499565	MGI:1858964	Actr1a
ENSMUST00000028619.4	0.272106119	MGI:1926967	Hsd17b12
ENSMUST00000028921.5	0.272649363	MGI:894687	Xrn2
ENSMUST00000103199.8	0.272649363	MGI:98342	Snrpb
ENSMUST00000071134.3	0.272979081	MGI:107813	Tubb3
ENSMUST00000053880.12	0.273886375	MGI:95821	Grin2b
ENSMUST00000061875.7	0.273886375	MGI:1289282	Zfp622
ENSMUST00000209440.1	0.273886375	MGI:1933134	Tusc3
ENSMUST00000156898.4	0.274902912	MGI:1915339	Arpc4
ENSMUST00000031029.14	0.275069739	MGI:2387801	Snx17
ENSMUST00000027626.12	0.275637828	MGI:1915199	Nifk
ENSMUST00000045855.8	0.275637828	MGI:1333880	Hebp1
ENSMUST00000068242.8	0.276191014	MGI:1918325	Rimklb
ENSMUST00000072727.6	0.277662449	MGI:1316687	Nap1l4
ENSMUST00000006911.11	0.27781521	MGI:88357	Cdk4
ENSMUST00000034796.13	0.278151196	MGI:1933331	Elovl4
ENSMUST00000071852.9	0.278151196	MGI:1914197	Commd7
ENSMUST00000079601.12	0.278151196	MGI:1096867	Etv5
ENSMUST00000080511.2	0.279306815	MGI:1861461	H1f5

ENSMUST00000000201.6	0.27949392	MGI:2444306	Nalcn
ENSMUST00000014499.9	0.27949392	MGI:103097	Anapc1
ENSMUST00000021048.6	0.27949392	MGI:1860295	Ftsj3
ENSMUST00000022567.8	0.27949392	MGI:1338890	Cacna2d3
ENSMUST00000023849.14	0.27949392	MGI:109382	Noct
ENSMUST00000025178.16	0.27949392	MGI:1330304	Vps52
ENSMUST00000025846.15	0.27949392	MGI:1921807	Ppp6r3
ENSMUST00000026360.8	0.27949392	MGI:1338035	Itgb8
ENSMUST00000027760.13	0.27949392	MGI:107484	Rgl1
ENSMUST00000029303.12	0.27949392	MGI:1922088	Naa15
ENSMUST00000030757.9	0.27949392	MGI:1924992	Fbxo42
ENSMUST00000035116.11	0.27949392	MGI:1914154	Pccb
ENSMUST00000035208.13	0.27949392	MGI:1277955	Bsn
ENSMUST00000036972.13	0.27949392	MGI:1914669	Armh4
ENSMUST00000038091.7	0.27949392	MGI:2384788	Sde2
ENSMUST00000043707.6	0.27949392	MGI:1915612	Rhbdd2
ENSMUST00000045942.8	0.27949392	MGI:95387	Emx1
ENSMUST00000046386.4	0.27949392	MGI:2159407	Zcchc14
ENSMUST00000049424.10	0.27949392	MGI:2147427	Wdr74
ENSMUST00000055993.12	0.27949392	MGI:2443887	Ric3
ENSMUST00000056635.12	0.27949392	MGI:1924705	Rlf
ENSMUST00000058914.9	0.27949392	MGI:1095409	Tuba1c
ENSMUST00000064477.13	0.27949392	MGI:107231	Dlg1
ENSMUST00000065080.9	0.27949392	MGI:1919014	C2cd2l
ENSMUST00000065297.5	0.27949392	MGI:3609241	Lonrf1
ENSMUST00000065977.10	0.27949392	MGI:1201386	Nrd1
ENSMUST00000066432.11	0.27949392	MGI:98249	Scn3a
ENSMUST00000068714.6	0.27949392	MGI:98354	Sos1
ENSMUST00000071370.6	0.27949392	MGI:2684924	Pcdh17
ENSMUST00000071500.12	0.27949392	MGI:94921	Dpp6
ENSMUST00000072312.11	0.27949392	MGI:1919857	Usp13
ENSMUST00000089217.10	0.27949392	MGI:98890	Uba1
ENSMUST00000089317.11	0.27949392	MGI:1203522	Magi1
ENSMUST00000091014.9	0.27949392	MGI:2442208	Map9
ENSMUST00000094463.4	0.27949392	MGI:1261813	Mn1
ENSMUST00000095228.3	0.27949392	MGI:2142433	Samd1
ENSMUST00000100013.8	0.27949392	MGI:2685387	Atp13a3
ENSMUST00000105840.7	0.27949392	MGI:2158502	Usp48
ENSMUST00000106908.8	0.27949392	MGI:99557	Pde4b

ENSMUST00000107975.7	0.27949392	MGI:2140712	Phf24
ENSMUST00000114020.9	0.27949392	MGI:2684957	Rexo4
ENSMUST00000118009.1	0.27949392	MGI:2682306	Naf1
ENSMUST00000118927.7	0.27949392	MGI:1919227	Zdhhc13
ENSMUST00000160882.7	0.27949392	MGI:104634	Tle3
ENSMUST00000161137.7	0.27949392	MGI:1928396	Pdcd10
ENSMUST00000161203.7	0.27949392	MGI:2444451	Rnf214
ENSMUST00000161804.7	0.27949392	MGI:1919847	Auts2
ENSMUST00000163347.7	0.27949392	MGI:2444817	C530008M17Rik
ENSMUST00000166232.3	0.27949392	MGI:108091	Bcar1
ENSMUST00000167182.7	0.27949392	MGI:2138741	Hnrnpf
ENSMUST00000180430.1	0.27949392	MGI:3610315	Ksr2
ENSMUST00000181969.7	0.27949392	MGI:1923523	Ndfip2
ENSMUST00000200393.4	0.27949392	MGI:1915760	Ints1
ENSMUST00000211140.1	0.27949392	MGI:97813	Ptpre
ENSMUST00000233427.1	0.27949392	MGI:1343877	Rpl10a
ENSMUST00000235822.1	0.27949392	MGI:3036282	Zfp532
ENSMUST00000002487.14	0.27949392	MGI:88190	Braf
ENSMUST00000004910.11	0.27949392	MGI:2145118	Eif2b2
ENSMUST00000022585.4	0.27949392	MGI:1914717	Gpalpp1
ENSMUST00000026225.14	0.27949392	MGI:1347047	Sema4g
ENSMUST00000030399.6	0.27949392	MGI:1858751	Rragc
ENSMUST00000030446.14	0.27949392	MGI:98916	Urod
ENSMUST00000032141.13	0.27949392	MGI:1914053	Hmces
ENSMUST00000032309.12	0.27949392	MGI:2137670	Ybx3
ENSMUST00000036992.8	0.27949392	MGI:102812	Lmo1
ENSMUST00000045748.6	0.27949392	MGI:2384308	Pdk3
ENSMUST00000052509.5	0.27949392	MGI:2442050	Zfp771
ENSMUST00000075686.6	0.27949392	MGI:2685083	Ado
ENSMUST00000103007.3	0.27949392	MGI:1930957	Nudt11
ENSMUST00000103035.9	0.27949392	MGI:1917632	Kctd2
ENSMUST00000109533.7	0.27949392	MGI:98397	Src
ENSMUST00000114913.2	0.27949392	MGI:99673	Adcy5
ENSMUST00000115662.8	0.27949392	MGI:2681880	Pcdha2
ENSMUST00000146150.2	0.27949392	MGI:1917963	Gpr137c
ENSMUST00000147147.7	0.27949392	MGI:1924933	Ehmt1
ENSMUST00000162460.7	0.27949392	MGI:97316	Nfya
ENSMUST00000210139.1	0.27949392	MGI:1916095	Pih1d1
ENSMUST00000211933.1	0.27949392	MGI:1914917	Alkbh8

ENSMUST00000000500.7	0.27949392	MGI:97528	Pdgfb
ENSMUST00000019803.8	0.27949392	MGI:1919904	Ccdc12
ENSMUST00000020550.12	0.27949392	MGI:102657	Cdc34
ENSMUST00000021173.13	0.27949392	MGI:1917150	Mfsd11
ENSMUST00000021797.8	0.27949392	MGI:1914296	Tbc1d7
ENSMUST00000025418.3	0.27949392	MGI:1922901	Psmg2
ENSMUST00000027186.11	0.27949392	MGI:1918077	Trak2
ENSMUST00000028835.12	0.27949392	MGI:1913836	Crls1
ENSMUST00000031094.14	0.27949392	MGI:1098708	Tbc1d14
ENSMUST00000033591.5	0.27949392	MGI:107706	Itm2a
ENSMUST00000036647.12	0.27949392	MGI:1196405	Ctdspl2
ENSMUST00000049031.5	0.27949392	MGI:2443086	Mindy2
ENSMUST00000050120.3	0.27949392	MGI:1914766	Kctd4
ENSMUST00000052011.14	0.27949392	MGI:104633	Tle4
ENSMUST00000057072.5	0.27949392	MGI:1336888	Prdx6b
ENSMUST00000059589.5	0.27949392	MGI:2136886	Rtn4r
ENSMUST00000059955.14	0.27949392	MGI:1917370	Yars2
ENSMUST00000059975.7	0.27949392	MGI:1914948	Fam174a
ENSMUST00000085177.4	0.27949392	MGI:1925103	Msl2
ENSMUST00000086423.5	0.27949392	MGI:3704480	Gm10184
ENSMUST00000087026.12	0.27949392	MGI:103288	Polr1c
ENSMUST00000091852.4	0.27949392	MGI:2652843	Mex3c
ENSMUST00000113460.7	0.27949392	MGI:1352466	Nr2c2
ENSMUST00000122358.7	0.27949392	MGI:1921390	Tm9sf1
ENSMUST00000159551.7	0.27949392	MGI:1926395	Wtap
ENSMUST00000060714.9	0.281182466	MGI:1860283	Ubqln2
ENSMUST00000014321.4	0.283442704	MGI:1914760	Tvp23b
ENSMUST00000032824.9	0.283442704	MGI:1346093	Psmc4
ENSMUST00000033282.4	0.283442704	MGI:1913415	Bccip
ENSMUST00000072965.4	0.283442704	MGI:1927664	Sirt2
ENSMUST00000087258.9	0.283442704	MGI:1928994	Tro
ENSMUST00000103241.7	0.283442704	MGI:2387661	Sema6d
ENSMUST00000047510.9	0.284584112	MGI:1347343	Usp5
ENSMUST00000073605.14	0.284584112	MGI:95640	Gapdh
ENSMUST00000111665.7	0.285126851	MGI:1914208	Tmx2
ENSMUST00000032477.5	0.285444067	MGI:1914852	Necap1
ENSMUST00000080281.13	0.287507047	MGI:1913730	Rpl15
ENSMUST00000033074.7	0.288219214	MGI:106442	Vkorc1
ENSMUST00000002091.5	0.28869242	MGI:1350933	Bcap31

ENSMUST00000127095.7	0.2888217	MGI:1346030	Ly6h
ENSMUST00000045054.10	0.28976861	MGI:1914665	Tcaf1
ENSMUST00000022616.13	0.289829985	MGI:88423	Clu
ENSMUST00000020702.10	0.290182418	MGI:96438	Igfbp3
ENSMUST00000029476.8	0.290182418	MGI:1338759	Sec22b
ENSMUST00000031355.9	0.290182418	MGI:1929095	Uso1
ENSMUST00000034843.8	0.290182418	MGI:1928268	Ireb2
ENSMUST00000047028.8	0.290182418	MGI:1916114	Lgalsl
ENSMUST00000064139.7	0.290182418	MGI:1915166	Plpp3
ENSMUST00000168574.8	0.290182418	MGI:2138391	Pid1
ENSMUST00000222098.1	0.290182418	MGI:1916487	Gtpbp4
ENSMUST00000167866.1	0.291160654	MGI:1915084	Idh3a
ENSMUST00000002403.9	0.291711339	MGI:1196314	Dhrs1
ENSMUST00000026495.14	0.292983492	MGI:88115	Atp5a1
ENSMUST00000034541.11	0.293839422	MGI:1914648	Srpr
ENSMUST00000030128.5	0.29525799	MGI:1924209	Chmp5
ENSMUST00000022135.14	0.295641613	MGI:5510732	Ak6
ENSMUST00000156400.8	0.295686114	MGI:3833940	Gm16286
ENSMUST00000019917.5	0.297545097	MGI:1913771	Rwdd1
ENSMUST00000000206.3	0.299059237	MGI:1919264	Btbd17
ENSMUST00000010807.5	0.299059237	MGI:1923517	Fads1
ENSMUST00000014642.9	0.299059237	MGI:2444029	Ankrd52
ENSMUST00000016897.11	0.299059237	MGI:2443047	Ttll1
ENSMUST00000025979.12	0.299059237	MGI:1888908	Aldh18a1
ENSMUST00000038677.4	0.299059237	MGI:2147437	Rrp12
ENSMUST00000045994.7	0.299059237	MGI:1098653	Rbm34
ENSMUST00000054815.14	0.299059237	MGI:1336199	Ppp1r13b
ENSMUST00000068023.7	0.299059237	MGI:2449088	Cadm4
ENSMUST00000073447.7	0.299059237	MGI:1935215	Pcdhga3
ENSMUST00000127430.1	0.299059237	MGI:1921605	Smchd1
ENSMUST00000032994.14	0.299870973	MGI:1920908	Spns1
ENSMUST00000049453.8	0.299870973	MGI:2140998	Ube3c
ENSMUST00000058615.9	0.299870973	MGI:1914185	Cir1
ENSMUST00000081291.12	0.299870973	MGI:1859320	Cyhr1
ENSMUST00000026891.4	0.300251049	MGI:1913696	Exosc7
ENSMUST00000065515.13	0.300471462	MGI:1338041	Tfg
ENSMUST00000032946.9	0.300615356	MGI:894313	Rab6a
ENSMUST00000226459.1	0.300615356	MGI:1913398	Dnajc15
ENSMUST00000114729.7	0.304600372	MGI:1929772	Dpysl5

ENSMUST00000026839.4	0.305548148	MGI:97776	Prps2
ENSMUST00000060455.14	0.305548148	MGI:109595	Ccndbp1
ENSMUST00000233692.1	0.305548148	MGI:2684934	Lrrc73
ENSMUST00000045628.14	0.305716372	MGI:1924814	R3hdm4
ENSMUST00000023133.7	0.306705613	MGI:1889595	Ppp1r1a
ENSMUST00000069926.13	0.307259715	MGI:2442509	Slitrk4
ENSMUST00000110057.2	0.307259715	MGI:1918686	Flrt3
ENSMUST00000161286.7	0.307259715	MGI:106016	Tra2b
ENSMUST00000029800.8	0.307744563	MGI:1928096	Tspan5
ENSMUST00000031119.5	0.307744563	MGI:103156	Gabrg1
ENSMUST00000033121.6	0.308849153	MGI:2385850	Nomo1
ENSMUST00000031249.7	0.309285912	MGI:108110	Sparcl1
ENSMUST00000102888.9	0.310275644	MGI:88256	Camk2a
ENSMUST00000003310.6	0.311281462	MGI:894323	Ranbp2
ENSMUST00000005705.7	0.311281462	MGI:109274	Trim28
ENSMUST00000022543.9	0.311281462	MGI:1915764	Micu2
ENSMUST00000023812.9	0.311281462	MGI:1346881	Map3k12
ENSMUST00000043961.11	0.311281462	MGI:2386607	Abca5
ENSMUST00000046875.13	0.311281462	MGI:2443162	Uggt1
ENSMUST00000113523.8	0.311281462	MGI:2387609	Tmem63b
ENSMUST00000115527.7	0.311281462	MGI:1915402	Fam133b
ENSMUST00000163558.2	0.311281462	MGI:1922847	Ndufaf2
ENSMUST00000077577.7	0.311768962	MGI:107804	Tuba1b
ENSMUST00000087315.13	0.311768962	MGI:90675	Vars
ENSMUST00000096261.4	0.311768962	MGI:1914960	Polr2g
ENSMUST00000027846.7	0.311915684	MGI:1196415	Tada1
ENSMUST00000234711.1	0.311915684	MGI:2136782	BC004004
ENSMUST00000094807.5	0.313484634	MGI:3645856	Pnmal2
ENSMUST00000022057.8	0.313879725	MGI:1920198	Tppp
ENSMUST00000008477.12	0.314621391	MGI:104805	Snrpb2
ENSMUST00000028278.13	0.316521942	MGI:1913284	Psmd14
ENSMUST00000239083.1	0.316588425	MGI:1933169	Sacm1l
ENSMUST00000160132.7	0.318022276	MGI:109207	Eif4g2
ENSMUST00000118163.7	0.318026921	MGI:2444630	Dmxl2
ENSMUST00000030622.2	0.319178264	MGI:1915842	Syf2
ENSMUST00000019734.10	0.31969974	MGI:103253	Cyb561
ENSMUST00000043627.7	0.320783797	MGI:1926273	Mrpl12
ENSMUST00000085077.4	0.32185909	MGI:107188	Arnt2
ENSMUST00000015877.13	0.3232088	MGI:106222	Capza2

ENSMUST00000075774.4	0.323812387	MGI:1920960	Tubb2b
ENSMUST00000067538.5	0.327705665	MGI:97815	Ptprs
ENSMUST00000004200.8	0.327767153	MGI:1913320	Cwc15
ENSMUST00000049937.12	0.327850335	MGI:1913761	Chtop
ENSMUST00000113097.7	0.327850335	MGI:1927167	Morf4l2
ENSMUST00000166170.8	0.327850335	MGI:1858510	Nell2
ENSMUST00000072697.12	0.328152751	MGI:87867	Acadm
ENSMUST00000020822.11	0.328473958	MGI:1916375	Cnot8
ENSMUST00000026129.15	0.328473958	MGI:1915921	Pcyt2
ENSMUST00000032093.11	0.328473958	MGI:1925144	Prickle2
ENSMUST00000069168.12	0.328473958	MGI:1913948	Cpsf6
ENSMUST00000112868.7	0.328473958	MGI:1339656	Pak3
ENSMUST00000137573.1	0.328473958	MGI:3584369	Zfp866
ENSMUST00000218879.1	0.328473958	MGI:3714359	Srp54c
ENSMUST00000006750.7	0.329180213	MGI:1858752	Rundc3a
ENSMUST00000015501.10	0.329180213	MGI:1346017	Clpx
ENSMUST00000018478.10	0.329180213	MGI:105051	Ksr1
ENSMUST00000022734.8	0.329180213	MGI:107373	Dnajc3
ENSMUST00000032924.5	0.329180213	MGI:1923739	Kctd13
ENSMUST00000033961.6	0.329180213	MGI:1916992	Tm2d2
ENSMUST00000037117.5	0.329180213	MGI:2138937	Gtf3c4
ENSMUST00000041240.3	0.329180213	MGI:1924802	Shisa4
ENSMUST00000042345.7	0.329180213	MGI:88274	Ctnna1
ENSMUST00000044083.8	0.329180213	MGI:1916885	Dapk1
ENSMUST00000044954.6	0.329180213	MGI:1345281	Slc30a1
ENSMUST00000046168.11	0.329180213	MGI:106316	Mpped1
ENSMUST00000051822.12	0.329180213	MGI:1917493	Wdr61
ENSMUST00000054920.4	0.329180213	MGI:2140300	Myorg
ENSMUST00000056512.13	0.329180213	MGI:2445123	Wdr41
ENSMUST00000061837.10	0.329180213	MGI:1921092	Neurl4
ENSMUST00000072572.12	0.329180213	MGI:2142632	Alg11
ENSMUST00000078324.6	0.329180213	MGI:103301	Cacnb4
ENSMUST00000082209.12	0.329180213	MGI:103169	Scn8a
ENSMUST00000102698.9	0.329180213	MGI:1917723	Rapgef4
ENSMUST00000113966.7	0.329180213	MGI:2687319	Il1rapl1
ENSMUST00000123491.7	0.329180213	MGI:1921462	Dnal1
ENSMUST00000127664.1	0.329180213	MGI:5141853	Galnt2l
ENSMUST00000143517.7	0.329180213	MGI:1346867	Map2k2
ENSMUST00000169266.7	0.329180213	MGI:1918972	Cic

ENSMUST00000170139.7	0.329180213	MGI:1914448	Spats2l
ENSMUST00000197284.4	0.329180213	MGI:95614	Gabra2
ENSMUST00000047672.8	0.333266226	MGI:107186	Cct2
ENSMUST00000021083.6	0.333960411	MGI:1096361	Jpt1
ENSMUST00000061796.7	0.334720835	MGI:2155249	Gpr6
ENSMUST00000000895.12	0.336199179	MGI:1861721	Necab3
ENSMUST00000027050.9	0.33645534	MGI:1349415	Cops5
ENSMUST00000102696.4	0.336973008	MGI:98166	Rps8
ENSMUST00000205002.2	0.339009104	MGI:1913358	Ndufa9
ENSMUST00000001592.14	0.339247196	MGI:96650	Jup
ENSMUST00000022916.12	0.339247196	MGI:2443132	Lrp12
ENSMUST00000023525.8	0.339247196	MGI:1921447	Gtf2e1
ENSMUST00000040729.8	0.339247196	MGI:1347048	Clcn7
ENSMUST00000092404.12	0.339247196	MGI:98284	Srsf2
ENSMUST00000217570.1	0.339247196	MGI:1919391	Adpgk
ENSMUST00000032168.6	0.340069169	MGI:1858417	Sec61a1
ENSMUST00000084097.11	0.342130289	MGI:1913327	Aurkaip1
ENSMUST00000021306.13	0.342679574	MGI:1336880	Eftud2
ENSMUST00000135573.7	0.342679574	MGI:99523	Prdx1
ENSMUST00000156816.6	0.342679574	MGI:1351639	Mrpl15
ENSMUST00000009344.15	0.344487149	MGI:2429950	Xpo6
ENSMUST00000028623.12	0.344487149	MGI:108050	Ext2
ENSMUST00000028758.7	0.344487149	MGI:1333822	Itpka
ENSMUST00000029663.10	0.344487149	MGI:102774	Aimp1
ENSMUST00000038671.9	0.344487149	MGI:2384899	Kri1
ENSMUST00000038695.5	0.344487149	MGI:96659	Kcna2
ENSMUST00000041555.9	0.344487149	MGI:1919793	Mvb12b
ENSMUST00000042021.4	0.344487149	MGI:2442458	Tspyl5
ENSMUST00000050872.14	0.344487149	MGI:103188	Nfib
ENSMUST00000058679.6	0.344487149	MGI:2442842	Mtmr9
ENSMUST00000059699.8	0.344487149	MGI:2685084	C2cd4c
ENSMUST00000075175.11	0.344487149	MGI:1913712	Rex1bd
ENSMUST00000102564.10	0.344487149	MGI:99474	Arrb2
ENSMUST00000114292.7	0.346372376	MGI:2442722	Cadm2
ENSMUST00000103220.3	0.346630967	MGI:2444401	Snrnp200
ENSMUST00000105364.7	0.347519081	MGI:1922656	Ndufs7
ENSMUST00000167493.8	0.347519081	MGI:1195456	Rnh1
ENSMUST00000029446.12	0.348004835	MGI:92356	Csde1
ENSMUST00000021313.8	0.352007497	MGI:1915337	Dcakd

ENSMUST00000069538.13	0.352007497	MGI:2443264	9330182L06Rik
ENSMUST00000022344.3	0.353313852	MGI:1917851	Ecd
ENSMUST00000027173.14	0.353313852	MGI:1927241	Wdr12
ENSMUST00000027434.14	0.353313852	MGI:1926045	Armc9
ENSMUST00000063562.8	0.353313852	MGI:1917728	Mipep
ENSMUST00000077408.7	0.353313852	MGI:2678952	Bloc1s3
ENSMUST00000162610.7	0.353313852	MGI:1913503	Aig1
ENSMUST00000191899.5	0.353313852	MGI:101864	Dag1
ENSMUST00000031009.7	0.355203835	MGI:109149	Nsg1
ENSMUST00000001507.4	0.355838199	MGI:106040	Cyp51
ENSMUST00000081893.6	0.356097359	MGI:98460	Syn1
ENSMUST00000119953.1	0.35712577	MGI:1913659	Rsl1d1
ENSMUST00000067826.14	0.359056657	MGI:2151172	Mta3
ENSMUST00000102632.6	0.359056657	MGI:1338015	Sort1
ENSMUST00000171342.2	0.359056657	MGI:1914838	Rnf41
ENSMUST00000003561.9	0.359313079	MGI:1860417	Phyhip
ENSMUST00000093360.11	0.359313079	MGI:2384849	Tnpo2
ENSMUST00000067476.8	0.36062877	MGI:1923937	Spcs3
ENSMUST00000033683.7	0.361925996	MGI:98158	Rps4x
ENSMUST00000085651.11	0.362195068	MGI:108077	Nptn
ENSMUST00000013845.12	0.363091436	MGI:1858317	Timm23
ENSMUST00000044923.14	0.363571338	MGI:1351337	Ddx24
ENSMUST00000107364.7	0.363571338	MGI:105306	Rab5c
ENSMUST00000023363.7	0.364045709	MGI:1925255	Rrn3
ENSMUST00000030164.7	0.364668009	MGI:99919	Vcp
ENSMUST00000035700.13	0.366570507	MGI:2384296	Camkv
ENSMUST00000026262.7	0.367056842	MGI:96073	Hexa
ENSMUST00000028603.9	0.367056842	MGI:1929084	Fbxo3
ENSMUST00000031309.15	0.367056842	MGI:2144041	Wsb2
ENSMUST00000033578.6	0.367056842	MGI:2148149	Magee1
ENSMUST00000039554.6	0.367056842	MGI:1914176	Trmt6
ENSMUST00000041806.12	0.367056842	MGI:1202717	Psen1
ENSMUST00000045866.8	0.367056842	MGI:1860494	Ddx21
ENSMUST00000050149.11	0.367056842	MGI:2444947	Mical2
ENSMUST00000052457.14	0.367056842	MGI:3039591	Mtss2
ENSMUST00000065625.11	0.367056842	MGI:1916185	Trmt1l
ENSMUST00000072896.12	0.367056842	MGI:1914461	Armc10
ENSMUST00000074669.9	0.367056842	MGI:1330294	Hnrnpab
ENSMUST00000075164.10	0.367056842	MGI:109234	Kif21b

ENSMUST00000107596.8	0.367056842	MGI:1933179	Srcin1
ENSMUST00000109236.8	0.367056842	MGI:1338864	Stau1
ENSMUST00000140299.2	0.367056842	MGI:2384919	Rxylt1
ENSMUST00000152227.7	0.367056842	MGI:106313	Desi1
ENSMUST00000168630.3	0.367056842	MGI:2389364	Unc5d
ENSMUST00000172785.7	0.367056842	MGI:95896	H2-D1
ENSMUST00000178202.7	0.367056842	MGI:1333766	Epn2
ENSMUST00000179669.2	0.367056842	MGI:1347464	Foxg1
ENSMUST00000202261.4	0.367056842	MGI:2136841	Morc3
ENSMUST00000204751.2	0.367056842	MGI:1203484	Zfp638
ENSMUST00000222167.1	0.367056842	MGI:2444706	Mdga2
ENSMUST00000022176.14	0.369830165	MGI:96159	Hmgcr
ENSMUST00000107305.7	0.369951162	MGI:1352760	Hdgfl3
ENSMUST00000078673.13	0.37021692	MGI:2444518	Samd12
ENSMUST00000021207.6	0.370379119	MGI:1923816	Rflnb
ENSMUST00000018875.12	0.371206273	MGI:1919020	Ap2b1
ENSMUST00000023426.11	0.371699927	MGI:2447860	Cldnd1
ENSMUST00000065694.7	0.371732885	MGI:2442555	Dis3l2
ENSMUST00000070117.7	0.371732885	MGI:1926143	Cnot10
ENSMUST00000090178.9	0.371732885	MGI:1917854	Dnajb14
ENSMUST00000125009.8	0.371732885	MGI:1351342	Grm5
ENSMUST00000182056.7	0.371732885	MGI:2685783	Baiap3
ENSMUST00000205568.1	0.371732885	MGI:1338823	Maz
ENSMUST00000000451.13	0.374572149	MGI:97847	Raf1
ENSMUST00000021314.7	0.374572149	MGI:102579	Nmt1
ENSMUST00000037489.14	0.374572149	MGI:1932075	Agpat1
ENSMUST00000118535.7	0.374572149	MGI:105069	Rab4a
ENSMUST00000159027.7	0.374572149	MGI:107163	Ppp3cb
ENSMUST00000237004.1	0.374572149	MGI:1860078	Txnl1
ENSMUST00000100026.9	0.376479223	MGI:1914751	Ccdc50
ENSMUST00000022108.8	0.377848457	MGI:1337006	Hapln1
ENSMUST00000002412.8	0.378879259	MGI:104694	Ncan
ENSMUST00000088244.5	0.380586792	MGI:2661120	Actr3b
ENSMUST00000081104.9	0.382269909	MGI:1343131	Timm17a
ENSMUST00000165335.7	0.385781655	MGI:95781	Gnb1
ENSMUST00000114548.7	0.387177871	MGI:1889272	Cadm1
ENSMUST00000164233.7	0.387330149	MGI:1270129	Dnaja1
ENSMUST00000091089.11	0.388310326	MGI:107384	Dnm1
ENSMUST00000027339.13	0.389420955	MGI:2138261	Smap1

ENSMUST00000021428.8	0.389729955	MGI:1913604	Snw1
ENSMUST00000029047.11	0.389729955	MGI:1921968	Snx16
ENSMUST00000020107.7	0.390104293	MGI:104653	Atp2b1
ENSMUST00000023854.9	0.390567146	MGI:1298387	Fhl1
ENSMUST00000036854.3	0.391154115	MGI:106504	Efhd2
ENSMUST00000020317.7	0.391922857	MGI:1913499	Pno1
ENSMUST00000020576.7	0.391922857	MGI:102890	Ccng1
ENSMUST00000051711.15	0.391922857	MGI:99638	Mark2
ENSMUST00000053986.8	0.391922857	MGI:3609246	Lingo3
ENSMUST00000034140.8	0.392973274	MGI:106419	Itfg1
ENSMUST00000044158.12	0.392973274	MGI:2154441	Nop53
ENSMUST00000047898.13	0.392973274	MGI:1354736	Kdm2a
ENSMUST00000101801.6	0.392973274	MGI:95994	Selenos
ENSMUST00000114144.8	0.392973274	MGI:2444277	Islr2
ENSMUST00000120447.7	0.392973274	MGI:1201607	Blzf1
ENSMUST00000014339.14	0.39449491	MGI:1928373	Dnajc7
ENSMUST00000029875.3	0.39449491	MGI:1919769	Pip4p2
ENSMUST00000047616.9	0.39449491	MGI:1858910	Jmjd6
ENSMUST00000058943.7	0.39449491	MGI:3617846	Ankrd34a
ENSMUST00000073364.5	0.39449491	MGI:2387687	Fam120c
ENSMUST00000075812.10	0.39449491	MGI:1276574	Nsd2
ENSMUST00000009036.10	0.395824709	MGI:106922	Vdac3
ENSMUST00000003183.11	0.396896534	MGI:102666	Ppp5c
ENSMUST00000018792.11	0.396896534	MGI:1927168	Dusp14
ENSMUST00000022102.8	0.396896534	MGI:2442892	Clptm1l
ENSMUST00000028951.13	0.396896534	MGI:2139270	Snph
ENSMUST00000064099.7	0.396896534	MGI:2442163	Ppp6r1
ENSMUST00000074950.10	0.396896534	MGI:1201779	Hnrnph2
ENSMUST00000106830.8	0.396896534	MGI:108554	Dab1
ENSMUST00000114449.6	0.396896534	MGI:1891748	Maea
ENSMUST00000126610.1	0.396896534	MGI:98002	Rpl12
ENSMUST00000134159.2	0.396896534	MGI:1919955	Zcchc17
ENSMUST00000178422.8	0.396896534	MGI:1203520	Dapk3
ENSMUST00000111632.4	0.39702426	MGI:1352498	Ndrg2
ENSMUST00000037941.9	0.397549931	MGI:1096398	Cd81
ENSMUST00000032898.8	0.399747553	MGI:1917822	Ipo5
ENSMUST00000102774.10	0.400930974	MGI:107931	Sqstm1
ENSMUST00000006853.10	0.401252053	MGI:1921693	P4htm
ENSMUST00000019615.10	0.401252053	MGI:109531	Cdc37

ENSMUST00000020316.3	0.401252053	MGI:1929658	Tbk1
ENSMUST00000020851.14	0.401252053	MGI:1917052	Cox11
ENSMUST00000024761.12	0.401252053	MGI:1354704	Fbxl17
ENSMUST00000025696.4	0.401252053	MGI:1860835	Ak3
ENSMUST00000028815.14	0.401252053	MGI:1859682	Slc23a2
ENSMUST00000029786.13	0.401252053	MGI:2137211	Mrpl9
ENSMUST00000031288.13	0.401252053	MGI:1930075	Tfip11
ENSMUST00000034739.11	0.401252053	MGI:1934919	Rnf111
ENSMUST00000034775.9	0.401252053	MGI:1335087	Fem1b
ENSMUST00000036719.11	0.401252053	MGI:3040696	Prex1
ENSMUST00000037270.4	0.401252053	MGI:1277178	Fam91a1
ENSMUST00000041099.4	0.401252053	MGI:1339708	Neurod1
ENSMUST00000049239.7	0.401252053	MGI:1925503	Map4k5
ENSMUST00000051207.1	0.401252053	MGI:2144150	Slc35e4
ENSMUST00000057557.13	0.401252053	MGI:1920977	Mcmbp
ENSMUST00000060805.6	0.401252053	MGI:2446163	Fam120a
ENSMUST00000060989.8	0.401252053	MGI:1202296	Sorl1
ENSMUST00000061262.10	0.401252053	MGI:2442488	Podxl2
ENSMUST00000062684.8	0.401252053	MGI:2140359	Tmem64
ENSMUST00000068581.8	0.401252053	MGI:95713	Gja1
ENSMUST00000099326.9	0.401252053	MGI:109137	Rasgrf2
ENSMUST00000106542.8	0.401252053	MGI:2445087	Hid1
ENSMUST00000117301.7	0.401252053	MGI:99216	Ddr1
ENSMUST00000129820.7	0.401252053	MGI:1919540	Lsm11
ENSMUST00000139129.8	0.401252053	MGI:98283	Srsf1
ENSMUST00000146590.7	0.401252053	MGI:1931881	Dnajb12
ENSMUST00000215073.1	0.401252053	MGI:1919334	Pigx
ENSMUST00000219915.1	0.401252053	MGI:1891704	Hbs1l
ENSMUST00000002710.9	0.401252053	MGI:1915329	Pdcd2l
ENSMUST00000015124.14	0.401252053	MGI:1913887	Tsen15
ENSMUST00000022550.7	0.401252053	MGI:1860765	Extl3
ENSMUST00000023221.12	0.401252053	MGI:1202392	Gpaa1
ENSMUST00000029090.8	0.401252053	MGI:1923675	Gid8
ENSMUST00000029699.12	0.401252053	MGI:96794	Lmna
ENSMUST00000030181.11	0.401252053	MGI:1913423	Ccdc107
ENSMUST00000031017.10	0.401252053	MGI:102858	Fosl2
ENSMUST00000032748.14	0.401252053	MGI:2142246	Unc45a
ENSMUST00000034264.10	0.401252053	MGI:1913421	Pgls
ENSMUST00000035667.8	0.401252053	MGI:1914775	Trim62

ENSMUST00000040531.8	0.401252053	MGI:2448542	Samd4b
ENSMUST00000092937.12	0.401252053	MGI:1891766	Camkk1
ENSMUST00000171561.7	0.401252053	MGI:1919449	Mms19
ENSMUST00000006774.10	0.401252053	MGI:1277216	Gtf2h1
ENSMUST00000009120.7	0.401252053	MGI:95610	Gabpa
ENSMUST00000011055.6	0.401252053	MGI:1926788	Apip
ENSMUST00000015481.5	0.401252053	MGI:1261433	Endog
ENSMUST00000025101.9	0.401252053	MGI:1913996	Dync2li1
ENSMUST00000028584.7	0.401252053	MGI:1923751	Commd9
ENSMUST00000029759.15	0.401252053	MGI:2442926	Mettl14
ENSMUST00000033950.6	0.401252053	MGI:1923847	Gins4
ENSMUST00000034920.10	0.401252053	MGI:1346345	Map2k5
ENSMUST00000048677.8	0.401252053	MGI:2681867	Tbc1d22b
ENSMUST00000066264.12	0.401252053	MGI:1858208	Ech1
ENSMUST00000078525.6	0.401252053	MGI:2443860	Rnf150
ENSMUST00000130911.7	0.401252053	MGI:97317	Nfyb
ENSMUST00000151288.7	0.401252053	MGI:2146236	Slc45a4
ENSMUST00000164787.7	0.401252053	MGI:1344366	Cdh18
ENSMUST00000171292.7	0.401252053	MGI:1925505	Ralgps2
ENSMUST00000172332.3	0.401252053	MGI:1919373	Ccdc71l
ENSMUST00000221919.1	0.401252053	MGI:3026685	Zfyve1
ENSMUST00000138532.7	0.40128392	MGI:1921376	Syvn1
ENSMUST00000028098.10	0.40321298	MGI:1347043	Orc4
ENSMUST00000049911.15	0.40321298	MGI:107365	Ube2i
ENSMUST00000006367.7	0.404731656	MGI:1929076	Htra1
ENSMUST00000193547.5	0.405004758	MGI:97281	Ncam1
ENSMUST00000021785.7	0.405707354	MGI:1913732	Exoc2
ENSMUST00000023343.3	0.405707354	MGI:1915091	Atg3
ENSMUST00000032124.8	0.405707354	MGI:1926274	Mrpl19
ENSMUST00000135088.8	0.405851507	MGI:891963	Ywhaq
ENSMUST00000070524.4	0.40668601	MGI:105080	Tgoln1
ENSMUST00000074015.10	0.40668601	MGI:2444846	Negr1
ENSMUST00000195717.5	0.407115321	MGI:1918348	Cep170
ENSMUST00000033342.6	0.408556339	MGI:1913335	Eif3f
ENSMUST00000102817.4	0.409537899	MGI:95639	Gap43
ENSMUST00000224625.1	0.411287754	MGI:102706	Fdft1
ENSMUST00000004786.9	0.413798128	MGI:1913670	Polr2e
ENSMUST00000032185.8	0.413798128	MGI:98488	Slc6a6
ENSMUST00000050214.8	0.413798128	MGI:1860606	Akap8l

ENSMUST00000080065.2	0.413798128	MGI:1347347	Slc27a4
ENSMUST00000081650.14	0.413798128	MGI:1351605	Rpl3
ENSMUST00000088896.9	0.413798128	MGI:2442368	Tmcc1
ENSMUST00000032440.5	0.414192821	MGI:99832	Sec13
ENSMUST00000066140.12	0.414192821	MGI:1935203	Pcdhgc4
ENSMUST00000155737.7	0.414192821	MGI:104581	Gnb4
ENSMUST00000084935.10	0.414448016	MGI:1918224	Pgm2l1
ENSMUST00000219317.1	0.416142815	MGI:99529	Cd63
ENSMUST00000004507.10	0.42101672	MGI:1277172	Ddx56
ENSMUST00000030427.5	0.42101672	MGI:1351315	Eloa
ENSMUST00000036691.13	0.42101672	MGI:1923304	Prrc2b
ENSMUST00000047309.5	0.42101672	MGI:3039561	Nat14
ENSMUST00000063854.6	0.42101672	MGI:3027896	Ppp4r2
ENSMUST00000055655.8	0.421233914	MGI:95485	Fasn
ENSMUST00000112856.2	0.423806198	MGI:1277171	Dcx
ENSMUST00000086216.8	0.42423254	MGI:1929722	Anapc5
ENSMUST00000053183.11	0.426141125	MGI:99432	Arf3
ENSMUST00000036274.7	0.428086982	MGI:1913874	Spcs2
ENSMUST00000006128.6	0.430242857	MGI:104559	Rcn1
ENSMUST00000018645.12	0.430376641	MGI:1349717	Ncor1
ENSMUST00000034879.4	0.430376641	MGI:1914117	Hmg20a
ENSMUST00000036439.5	0.430376641	MGI:107435	Cdh6
ENSMUST00000038570.8	0.430376641	MGI:1278344	Nipsnap1
ENSMUST00000091903.4	0.430376641	MGI:1344391	Sh3bp5
ENSMUST00000113792.1	0.431337658	MGI:1101765	Pja1
ENSMUST00000234810.1	0.431419205	MGI:102790	Rab18
ENSMUST00000020755.11	0.433346855	MGI:2144474	Ppp4r3b
ENSMUST00000024816.12	0.433346855	MGI:1921407	Cmtr1
ENSMUST00000026267.15	0.433346855	MGI:1914537	Parp6
ENSMUST00000033333.12	0.433346855	MGI:1915254	Tmem9b
ENSMUST00000034713.8	0.433346855	MGI:96765	Ldlr
ENSMUST00000035325.14	0.433346855	MGI:1330818	Qsox1
ENSMUST00000039333.9	0.433346855	MGI:2442188	Pdpr
ENSMUST00000041282.12	0.433346855	MGI:2153072	Trim37
ENSMUST00000049896.12	0.433346855	MGI:1859610	Gpkow
ENSMUST00000058902.5	0.433346855	MGI:2442895	Eml6
ENSMUST00000069399.6	0.433346855	MGI:1922151	Kbtbd11
ENSMUST00000071564.13	0.433346855	MGI:1354699	Fbh1
ENSMUST00000106113.1	0.433346855	MGI:1916087	Foxk2

ENSMUST00000109283.1	0.433346855	MGI:2146030	Slc2a13
ENSMUST00000114985.9	0.433346855	MGI:98506	Tcf4
ENSMUST00000115812.9	0.433346855	MGI:2445019	Pik3c3
ENSMUST00000209579.1	0.433346855	MGI:1100517	Clpb
ENSMUST00000029405.7	0.43344814	MGI:2448526	Gmps
ENSMUST00000209099.1	0.43344814	MGI:98886	U2af2
ENSMUST00000045068.9	0.435836028	MGI:2384571	Cplx3
ENSMUST00000062677.11	0.435836028	MGI:2144726	Tmem11
ENSMUST00000098950.5	0.435836028	MGI:1100851	Elavl1
ENSMUST00000107157.8	0.435836028	MGI:1923626	Slc24a2
ENSMUST00000040538.9	0.438421186	MGI:1924486	Sccpdh
ENSMUST00000030212.14	0.439412665	MGI:700009	Sh3gl2
ENSMUST00000151937.7	0.440675833	MGI:1926080	Slc25a12
ENSMUST00000005256.13	0.440920601	MGI:1341799	Ndrg1
ENSMUST00000019199.13	0.440920601	MGI:99907	Plod1
ENSMUST00000098924.8	0.440920601	MGI:1889575	Adar
ENSMUST00000176381.7	0.440920601	MGI:1928483	Stx5a
ENSMUST00000021536.8	0.441303625	MGI:1921084	Atp6v1d
ENSMUST00000173867.7	0.442374529	MGI:104689	Cct4
ENSMUST00000179238.7	0.442437152	MGI:97783	Psap
ENSMUST00000098651.5	0.444770216	MGI:1913125	Pias1
ENSMUST00000107107.8	0.444770216	MGI:104810	Plaa
ENSMUST00000112862.6	0.444770216	MGI:1921442	Arpc5l
ENSMUST00000178328.7	0.444770216	MGI:1351614	Gyg
ENSMUST00000021217.10	0.449410322	MGI:97356	Nme2
ENSMUST00000028551.3	0.45180781	MGI:1915282	Emc4
ENSMUST00000021668.9	0.452011824	MGI:1915213	Npc2
ENSMUST00000207883.1	0.452395599	MGI:1201690	Map6
ENSMUST00000079828.6	0.454736118	MGI:97384	Ntrk2
ENSMUST00000120381.8	0.456952978	MGI:105124	Stt3a
ENSMUST00000021940.7	0.456997886	MGI:1914140	Lman2
ENSMUST00000121720.1	0.456997886	MGI:106654	Nap1l2
ENSMUST00000027741.11	0.457130231	MGI:97932	Xpr1
ENSMUST00000039431.13	0.457233611	MGI:97385	Ntrk3
ENSMUST00000118152.7	0.457384683	MGI:109372	Cbx5
ENSMUST00000088001.5	0.457626344	MGI:1338758	Adnp
ENSMUST00000093165.11	0.457626344	MGI:1924134	Cyfip2
ENSMUST00000095012.9	0.457626344	MGI:107558	Sema3a
ENSMUST00000160852.7	0.457626344	MGI:6303289	Gm50387

ENSMUST00000164782.9	0.457626344	MGI:1278313	Coch
ENSMUST00000003268.10	0.458074965	MGI:700010	Sh3gl1
ENSMUST00000004133.10	0.458074965	MGI:2443333	Brinp2
ENSMUST00000023830.15	0.458074965	MGI:1196365	Nus1
ENSMUST00000025925.10	0.458074965	MGI:2179715	Plaat3
ENSMUST00000043616.6	0.458074965	MGI:2685277	Zyg11b
ENSMUST00000052236.12	0.458074965	MGI:2686937	Fbxo10
ENSMUST00000106027.8	0.458074965	MGI:2141847	Phrf1
ENSMUST00000109901.8	0.458074965	MGI:109301	Papola
ENSMUST00000113028.1	0.458074965	MGI:1916111	Dipk2a
ENSMUST00000113573.7	0.458074965	MGI:103067	Atrx
ENSMUST00000078124.7	0.460104811	MGI:101763	Cfl2
ENSMUST00000050552.14	0.462540489	MGI:1914132	Bzw1
ENSMUST00000086199.11	0.464515798	MGI:95739	Glul
ENSMUST00000062125.10	0.464910885	MGI:1917023	Timm29
ENSMUST00000036004.15	0.465553797	MGI:104820	Hnrnpa1
ENSMUST00000034988.9	0.466397943	MGI:1916769	Rwdd2a
ENSMUST00000047951.8	0.466397943	MGI:2446216	Fbxo2
ENSMUST00000026016.12	0.468531107	MGI:1919268	Fundc1
ENSMUST00000091556.11	0.468531107	MGI:2385155	Btbd3
ENSMUST00000022875.6	0.471848335	MGI:3045421	Ank
ENSMUST00000027579.16	0.471848335	MGI:1921367	Actr3
ENSMUST00000033695.5	0.471848335	MGI:109533	Abcb7
ENSMUST00000090006.11	0.471848335	MGI:96610	Itgb1
ENSMUST00000108241.7	0.471848335	MGI:2445193	Utp6
ENSMUST00000162875.7	0.471848335	MGI:1919347	2010300C02Rik
ENSMUST00000000759.8	0.472085012	MGI:1920159	Chmp1a
ENSMUST00000018186.15	0.472169123	MGI:94893	Cyb5r3
ENSMUST00000022063.13	0.477714244	MGI:1914683	Ccdc127
ENSMUST00000028807.5	0.477714244	MGI:1929242	Ivd
ENSMUST00000034876.9	0.477714244	MGI:1928098	Tspan3
ENSMUST00000042281.13	0.477714244	MGI:2384931	Dhrs7b
ENSMUST00000053918.8	0.477714244	MGI:1913444	Pycrl
ENSMUST00000066285.5	0.477714244	MGI:1920692	Hspa12a
ENSMUST00000066496.9	0.477714244	MGI:2144158	Nudcd3
ENSMUST00000071898.6	0.477714244	MGI:2679722	Cpsf1
ENSMUST00000086521.10	0.477714244	MGI:104518	Cntn2
ENSMUST00000219706.1	0.477714244	MGI:1914820	Slc39a9
ENSMUST00000022496.8	0.478451947	MGI:1100863	Kpna3

ENSMUST00000068456.7	0.478451947	MGI:95617	Gabra5
ENSMUST00000079278.4	0.478451947	MGI:2684969	Nrsn2
ENSMUST00000111288.3	0.478451947	MGI:2155987	Caln1
ENSMUST00000102871.9	0.478945426	MGI:96721	L1cam
ENSMUST00000022894.13	0.479630625	MGI:109484	Ywhaz
ENSMUST00000036125.9	0.481502801	MGI:1341822	Eif4h
ENSMUST00000131656.1	0.482328002	MGI:1917143	Coa7
ENSMUST00000022286.7	0.482758457	MGI:1343135	Ndufs4
ENSMUST00000035295.5	0.48356947	MGI:1097711	Degs1
ENSMUST00000029414.11	0.48396146	MGI:1914687	Ssr3
ENSMUST00000035045.14	0.48410272	MGI:1100856	Mras
ENSMUST00000051870.7	0.48410272	MGI:1196398	Champ1
ENSMUST00000056136.3	0.48410272	MGI:1194504	Kcnj10
ENSMUST00000111435.8	0.48410272	MGI:1915731	Mpzl1
ENSMUST00000072334.11	0.484146391	MGI:892995	Dnajc5
ENSMUST00000003451.10	0.48668115	MGI:2444878	Rnd1
ENSMUST00000064433.10	0.48668115	MGI:1355335	Tmod2
ENSMUST00000019198.6	0.49056945	MGI:1913687	Fis1
ENSMUST00000220621.1	0.491541932	MGI:2137226	Mrpl32
ENSMUST00000154166.7	0.492796423	MGI:1933830	Enpp5
ENSMUST00000019302.9	0.493690367	MGI:1916344	Tmem160
ENSMUST00000022623.12	0.493690367	MGI:1914104	Trim35
ENSMUST00000031003.10	0.493690367	MGI:2442660	Ppp2r2c
ENSMUST00000079258.6	0.493690367	MGI:894702	Numbl
ENSMUST00000135807.1	0.493690367	MGI:1925031	Epm2aip1
ENSMUST00000222508.1	0.493690367	MGI:1913939	Klhl28
ENSMUST00000237783.1	0.493690367	MGI:104719	Ndst1
ENSMUST00000022451.13	0.49477177	MGI:1338030	Capn7
ENSMUST00000029542.11	0.49477177	MGI:2140050	Ints3
ENSMUST00000077066.7	0.49477177	MGI:2147713	Tmem151a
ENSMUST00000058119.8	0.495930793	MGI:1924226	Arxes2
ENSMUST00000100301.10	0.495944503	MGI:97597	Prkcg
ENSMUST00000047910.14	0.499517921	MGI:1929701	Metap2
ENSMUST00000001240.11	0.502597628	MGI:1336186	Agpat3
ENSMUST00000143567.7	0.502597628	MGI:88111	Atp2b4
ENSMUST00000000199.7	0.504144678	MGI:109166	Ncs1
ENSMUST00000044148.2	0.504144678	MGI:1914731	Alg2
ENSMUST00000034591.10	0.506941553	MGI:1346542	Bace1
ENSMUST00000037001.9	0.506941553	MGI:1915864	Letmd1

ENSMUST00000060210.13	0.510331013	MGI:107672	Gpm6b
ENSMUST00000071103.9	0.510968714	MGI:2144727	Ddx1
ENSMUST00000032454.7	0.512172827	MGI:95627	Slc6a1
ENSMUST00000043396.14	0.512444278	MGI:97357	Mycn
ENSMUST00000069360.13	0.512444278	MGI:104903	Gpc3
ENSMUST00000113658.7	0.512444278	MGI:95698	Gfpt1
ENSMUST00000020640.7	0.513209158	MGI:101849	Rack1
ENSMUST00000018880.13	0.513529219	MGI:1932915	Ndel1
ENSMUST00000022782.9	0.513529219	MGI:1929480	Lrp10
ENSMUST00000026547.8	0.513529219	MGI:1921487	Tubgcp2
ENSMUST00000030170.14	0.513529219	MGI:3051532	Unc13a
ENSMUST00000031144.13	0.513529219	MGI:894407	Tmem165
ENSMUST00000041968.10	0.513529219	MGI:1920009	Tmem135
ENSMUST00000048688.7	0.513529219	MGI:2444639	Fbxo38
ENSMUST00000049005.14	0.513529219	MGI:1338938	Bmpr1a
ENSMUST00000055833.11	0.513529219	MGI:109349	Atf2
ENSMUST00000057669.15	0.513529219	MGI:1346859	Mapk3
ENSMUST00000067190.11	0.513529219	MGI:96788	Lifr
ENSMUST00000071169.8	0.513529219	MGI:1919784	Rcc2
ENSMUST00000082237.6	0.513529219	MGI:1918252	Mex3b
ENSMUST00000087617.10	0.513529219	MGI:1915126	Coq10b
ENSMUST00000094097.11	0.513529219	MGI:1289225	Tmem41b
ENSMUST00000112558.9	0.513529219	MGI:1889008	Atp2c1
ENSMUST00000112601.8	0.513529219	MGI:87911	Acvr1
ENSMUST00000118878.7	0.513529219	MGI:1336878	Taf1
ENSMUST00000150843.7	0.513529219	MGI:1921700	Pank2
ENSMUST00000161354.8	0.513529219	MGI:1890594	Abcg4
ENSMUST00000163949.8	0.513529219	MGI:96968	Mest
ENSMUST00000167487.7	0.513529219	MGI:98935	Vldlr
ENSMUST00000213937.1	0.513529219	MGI:2143099	AI593442
ENSMUST00000023684.13	0.513529219	MGI:95654	Gart
ENSMUST00000023867.7	0.513529219	MGI:1341868	Rfc2
ENSMUST00000025745.9	0.513529219	MGI:2660674	Mrpl21
ENSMUST00000027973.13	0.513529219	MGI:1923580	Sephs1
ENSMUST00000029941.15	0.513529219	MGI:1927489	Pdlim5
ENSMUST00000034076.15	0.513529219	MGI:88281	Cbln1
ENSMUST00000043760.14	0.513529219	MGI:107624	Mvk
ENSMUST00000054048.9	0.513529219	MGI:2441884	Mrgpre
ENSMUST00000069449.6	0.513529219	MGI:1914172	Rras2

ENSMUST00000080008.12	0.513529219	MGI:1924288	Arfgap2
ENSMUST00000099557.9	0.513529219	MGI:2679420	Pak6
ENSMUST00000228366.2	0.513529219	MGI:2385017	Nrbp2
ENSMUST00000005064.13	0.513529219	MGI:2136940	Pias4
ENSMUST00000005490.9	0.513529219	MGI:1096331	Slc1a6
ENSMUST00000014698.9	0.513529219	MGI:1351602	Dguok
ENSMUST00000017908.2	0.513529219	MGI:1919221	Zswim1
ENSMUST00000018002.12	0.513529219	MGI:2387217	Ift52
ENSMUST00000023219.8	0.513529219	MGI:1354705	Fbxl6
ENSMUST00000024783.8	0.513529219	MGI:1858419	Bysl
ENSMUST00000025834.14	0.513529219	MGI:1924963	Peli3
ENSMUST00000026989.14	0.513529219	MGI:1921162	4833439L19Rik
ENSMUST00000028080.11	0.513529219	MGI:1921353	Nebl
ENSMUST00000028356.8	0.513529219	MGI:1913455	Cd302
ENSMUST00000029696.10	0.513529219	MGI:1921450	Khdc4
ENSMUST00000029908.7	0.513529219	MGI:1923382	Faxc
ENSMUST00000032194.10	0.513529219	MGI:1097714	Bhlhe40
ENSMUST00000034239.8	0.513529219	MGI:1921437	Katnb1
ENSMUST00000037190.14	0.513529219	MGI:1919862	Hpf1
ENSMUST00000038584.8	0.513529219	MGI:2441683	Tlk1
ENSMUST00000040560.10	0.513529219	MGI:1913649	Tsfm
ENSMUST00000043305.13	0.513529219	MGI:2685541	Wdtc1
ENSMUST00000063169.9	0.513529219	MGI:2447771	Dleu7
ENSMUST00000066498.7	0.513529219	MGI:3045722	Tmem255a
ENSMUST00000075452.6	0.513529219	MGI:1921527	Chic2
ENSMUST00000086421.8	0.513529219	MGI:1306821	Nck2
ENSMUST00000105287.10	0.513529219	MGI:94872	Dcn
ENSMUST00000106107.2	0.513529219	MGI:2183451	Rab40b
ENSMUST00000116375.1	0.513529219	MGI:1914587	Cstf1
ENSMUST00000118108.1	0.513529219	MGI:2139530	Rabepk
ENSMUST00000123614.7	0.513529219	MGI:1916372	Ptgr2
ENSMUST00000144711.8	0.513529219	MGI:1924662	Wdr17
ENSMUST00000160399.7	0.513529219	MGI:1277169	Echdc1
ENSMUST00000004646.12	0.51811062	MGI:1345964	Coro1c
ENSMUST00000038973.6	0.51811062	MGI:2147006	Gnptg
ENSMUST00000070080.5	0.51811062	MGI:1928380	B4galt6
ENSMUST00000110942.10	0.51811062	MGI:1914466	Mboat2
ENSMUST00000027602.14	0.518831706	MGI:2442544	Dars
ENSMUST00000030491.8	0.520757196	MGI:1913838	Cmpk1

ENSMUST00000043235.7	0.520757196	MGI:1915087	Tiprl
ENSMUST00000084502.6	0.521319749	MGI:1343463	Bub3
ENSMUST00000022842.15	0.521894778	MGI:107185	Cct5
ENSMUST00000003754.7	0.524524532	MGI:101914	Calb2
ENSMUST00000005964.6	0.524524532	MGI:87929	Adh5
ENSMUST00000223396.1	0.525849851	MGI:99845	Gdi2
ENSMUST00000100528.4	0.526485625	MGI:1343160	Ube2z
ENSMUST00000022075.5	0.527652827	MGI:97511	Pcsk1
ENSMUST00000035276.4	0.527652827	MGI:1913672	Dctpp1
ENSMUST00000066091.13	0.527652827	MGI:109452	Smad1
ENSMUST00000015146.15	0.530496199	MGI:1923990	Efr3a
ENSMUST00000018625.9	0.530496199	MGI:1914134	Appbp2
ENSMUST00000023217.10	0.530496199	MGI:1334460	Bop1
ENSMUST00000031793.7	0.530496199	MGI:1927186	Nt5c3
ENSMUST00000037370.13	0.530496199	MGI:1932289	Sorcs2
ENSMUST00000044442.9	0.530496199	MGI:1918711	Ptk7
ENSMUST00000091291.4	0.530496199	MGI:96575	Insr
ENSMUST00000097373.1	0.530496199	MGI:102548	Tsc2
ENSMUST00000102849.10	0.530496199	MGI:1921520	Usp20
ENSMUST00000115657.9	0.530496199	MGI:1298372	Pcdha11
ENSMUST00000115657.9	0.530496199	MGI:1298372	Pcdha11
ENSMUST00000115657.9	0.530496199	MGI:1298372	Pcdha11
ENSMUST00000115657.9	0.530496199	MGI:1298372	Pcdha11
ENSMUST00000117136.1	0.530496199	MGI:1922845	Mzt2
ENSMUST00000170819.1	0.530496199	MGI:1922248	Rab11fip2
ENSMUST00000092907.11	0.530553918	MGI:1915525	Rpa1
ENSMUST00000102759.7	0.532958051	MGI:1929100	Stam2
ENSMUST00000004140.10	0.534025505	MGI:95860	Gstm1
ENSMUST00000022497.14	0.534545747	MGI:1913924	Spryd7
ENSMUST00000034349.9	0.535899662	MGI:2384561	Nae1
ENSMUST00000039655.2	0.535899662	MGI:1916092	Tulp4
ENSMUST00000115099.8	0.535899662	MGI:2442917	Fam171a1
ENSMUST00000023869.14	0.537362286	MGI:1915434	Denr
ENSMUST00000008684.10	0.53791119	MGI:1913850	Mgst1
ENSMUST00000002292.14	0.543098243	MGI:1915727	Rmnd5a
ENSMUST00000006293.4	0.543690037	MGI:104686	Crkl
ENSMUST00000020706.4	0.543690037	MGI:99677	Adcy1
ENSMUST00000022369.8	0.543690037	MGI:98927	Vcl
ENSMUST00000023088.7	0.543690037	MGI:1261422	Naga

ENSMUST00000026265.7	0.543690037	MGI:2143311	Bbs4
ENSMUST00000026902.8	0.543690037	MGI:2179722	Rassf3
ENSMUST00000051691.7	0.543690037	MGI:103241	Slk
ENSMUST00000077271.8	0.543690037	MGI:107339	Gfm1
ENSMUST00000118077.7	0.543690037	MGI:2143698	Vezt
ENSMUST00000135343.1	0.543690037	MGI:1349455	Gprin1
ENSMUST00000194558.5	0.543690037	MGI:1100848	Kpna4
ENSMUST00000023269.4	0.547389912	MGI:1915443	Rpl24
ENSMUST00000032585.7	0.547389912	MGI:1913411	Pop4
ENSMUST00000029142.14	0.549436567	MGI:1196288	Eif6
ENSMUST00000081848.12	0.549436567	MGI:104888	Fdps
ENSMUST00000113600.9	0.549436567	MGI:105068	Rab7
ENSMUST00000031377.8	0.55154841	MGI:1196458	Scarb2
ENSMUST00000119901.8	0.552645445	MGI:104556	Cdkn1a
ENSMUST00000047857.15	0.554243815	MGI:2152883	Fbxl5
ENSMUST00000076431.12	0.554243815	MGI:1918568	Pmpca
ENSMUST00000099571.9	0.554243815	MGI:1316652	Prmt2
ENSMUST00000115258.8	0.554243815	MGI:1919943	Zcchc12
ENSMUST00000085092.11	0.555060197	MGI:1929813	Cacna2d2
ENSMUST00000055518.12	0.555380484	MGI:97583	Pik3r1
ENSMUST00000025570.7	0.559416792	MGI:1913322	Sdhaf2
ENSMUST00000004094.14	0.560083599	MGI:1914220	Ssbp2
ENSMUST00000016463.3	0.562570271	MGI:1353496	Slc25a5
ENSMUST00000023151.5	0.562878083	MGI:107187	Bcl6
ENSMUST00000041616.14	0.562878083	MGI:1351869	Pdxk
ENSMUST00000046122.10	0.562878083	MGI:1914238	Lap3
ENSMUST00000049245.9	0.562878083	MGI:1343045	Rbmxl1
ENSMUST00000095427.11	0.562878083	MGI:1929261	Mtch1
ENSMUST00000106000.9	0.562878083	MGI:1096360	Cd151
ENSMUST00000211765.1	0.562878083	MGI:97388	Nucb1
ENSMUST00000027432.8	0.564431569	MGI:1917497	Psmd1
ENSMUST00000002090.2	0.565832479	MGI:1099464	Ssr4
ENSMUST00000028583.7	0.567607445	MGI:1330839	Lin7c
ENSMUST00000026560.13	0.567800905	MGI:1345192	Psmd13
ENSMUST00000087521.12	0.568677123	MGI:1929485	Nif3l1
ENSMUST00000002923.9	0.569308406	MGI:1098234	Adprh
ENSMUST00000030134.8	0.570072988	MGI:105128	Rad23b
ENSMUST00000113200.7	0.570072988	MGI:2684789	Lrsam1
ENSMUST00000120240.7	0.570072988	MGI:1913290	Nfu1

ENSMUST00000136872.7	0.570072988	MGI:1929260	Mtch2
ENSMUST00000092584.5	0.572212763	MGI:96907	Marcks
ENSMUST00000032196.8	0.572380976	MGI:1914416	Arl8b
ENSMUST00000029358.14	0.575759098	MGI:2140103	Nmd3
ENSMUST00000022281.4	0.576160828	MGI:1919448	Mtrex
ENSMUST00000025714.8	0.576160828	MGI:1859683	Rpp30
ENSMUST00000160197.5	0.576160828	MGI:1923164	Exoc6b
ENSMUST00000061372.6	0.576849886	MGI:1298395	Tspyl1
ENSMUST00000035673.7	0.581378906	MGI:103223	Vhl
ENSMUST00000088935.3	0.581378906	MGI:2444393	Zdhhc9
ENSMUST00000153470.8	0.581378906	MGI:96738	Hikeshi
ENSMUST00000028817.6	0.581821315	MGI:97503	Pcna
ENSMUST00000007959.13	0.581958501	MGI:1096342	Rhoa
ENSMUST00000167824.2	0.583668235	MGI:1914545	Rab3c
ENSMUST00000026841.14	0.585901216	MGI:2136381	Hadhb
ENSMUST00000059596.7	0.585901216	MGI:2681174	Eid2
ENSMUST00000078286.5	0.585901216	MGI:1913479	Rpl7l1
ENSMUST00000099172.4	0.585901216	MGI:109564	Kif5a
ENSMUST00000074267.4	0.586582587	MGI:1333818	Rps7
ENSMUST00000030475.2	0.586745228	MGI:1919431	Nsun4
ENSMUST00000105994.3	0.586745228	MGI:1913835	Snrnp40
ENSMUST00000063192.14	0.587074157	MGI:3041258	Tmem150c
ENSMUST00000020707.11	0.588458728	MGI:95613	Gabra1
ENSMUST00000033012.8	0.589858237	MGI:1917599	Copb1
ENSMUST00000021990.3	0.593349739	MGI:1276575	Ptdss1
ENSMUST00000023630.15	0.593349739	MGI:1860263	Psmg1
ENSMUST00000030398.9	0.593349739	MGI:95755	Slc2a1
ENSMUST00000198518.4	0.593349739	MGI:1861691	Lgi1
ENSMUST00000239015.1	0.594218441	MGI:1916933	Emc10
ENSMUST00000098453.8	0.596453314	MGI:1913570	Tmem208
ENSMUST00000004554.13	0.597292144	MGI:1097682	Rps5
ENSMUST00000022967.6	0.599230218	MGI:1914748	Kcnv1
ENSMUST00000102538.10	0.601729452	MGI:108564	Meis2
ENSMUST00000006912.11	0.603990685	MGI:1298206	Pip4k2a
ENSMUST00000026173.12	0.606046371	MGI:1914090	Wdr45b
ENSMUST00000011492.14	0.609643871	MGI:1914272	Acad9
ENSMUST00000020657.12	0.614040918	MGI:102944	Ube2b
ENSMUST00000154689.7	0.614040918	MGI:1917338	Meaf6
ENSMUST00000018122.13	0.617695862	MGI:2443963	Cadps2

ENSMUST00000022040.13	0.617695862	MGI:2159437	Agtpbp1
ENSMUST00000024854.8	0.617695862	MGI:1919100	Clip4
ENSMUST00000040110.7	0.617695862	MGI:1927166	Chst11
ENSMUST00000042729.15	0.617695862	MGI:2148811	Npnt
ENSMUST00000058488.8	0.617695862	MGI:1913361	Tmed3
ENSMUST00000063520.14	0.617695862	MGI:1919367	Naa50
ENSMUST00000102559.10	0.617695862	MGI:1355329	Mink1
ENSMUST00000112698.7	0.617695862	MGI:1277212	Zfp644
ENSMUST00000147663.7	0.617695862	MGI:2389180	Lrrtm4
ENSMUST00000162280.1	0.617695862	MGI:3057108	Lhfpl4
ENSMUST00000182559.7	0.617695862	MGI:1922665	Arhgap12
ENSMUST00000189772.1	0.617695862	MGI:5579341	Gm28635
ENSMUST00000199708.4	0.617695862	MGI:95820	Grin2a
ENSMUST00000019026.9	0.617695862	MGI:1914286	Mrpl45
ENSMUST00000029629.14	0.617695862	MGI:1913600	Pla2g12a
ENSMUST00000034934.14	0.617695862	MGI:3522097	Aph1b
ENSMUST00000040445.8	0.617695862	MGI:1930964	Thap11
ENSMUST00000042766.5	0.617695862	MGI:2442111	Ppm1k
ENSMUST00000057256.4	0.617695862	MGI:1925127	6030458C11Rik
ENSMUST00000189877.6	0.617695862	MGI:2136810	Lrfn1
ENSMUST00000007993.15	0.617695862	MGI:2655711	Rbm28
ENSMUST00000010201.8	0.617695862	MGI:1914482	Nprl2
ENSMUST00000020523.3	0.617695862	MGI:1919379	Pex13
ENSMUST00000022767.15	0.617695862	MGI:1927165	Mettl3
ENSMUST00000025981.14	0.617695862	MGI:1914840	Tctn3
ENSMUST00000033070.8	0.617695862	MGI:1915023	Kat8
ENSMUST00000034466.9	0.617695862	MGI:1343460	Gnpat
ENSMUST00000038434.3	0.617695862	MGI:1917211	Rpp25l
ENSMUST00000042852.6	0.617695862	MGI:1914000	Fam210a
ENSMUST00000070797.6	0.617695862	MGI:2150982	Pcdha1
ENSMUST00000099153.9	0.617695862	MGI:1914370	Ttc14
ENSMUST00000102780.7	0.617695862	MGI:87979	Ak4
ENSMUST00000102898.4	0.617695862	MGI:1353472	Rpl7a
ENSMUST00000111663.8	0.617695862	MGI:1914779	Fgfr1op2
ENSMUST00000124187.7	0.617695862	MGI:1913546	Haus2
ENSMUST00000154241.7	0.617695862	MGI:97138	Mpv17
ENSMUST00000164472.7	0.617695862	MGI:2448537	Zfp637
ENSMUST00000164822.7	0.617695862	MGI:1917200	Rcbtb2
ENSMUST00000166176.8	0.617695862	MGI:1336894	Mocs2

ENSMUST00000178440.7	0.617695862	MGI:1347053	Git2
ENSMUST00000023615.6	0.618579943	MGI:1206040	Vps26c
ENSMUST00000001513.7	0.621514322	MGI:1915201	Tubb6
ENSMUST00000003622.15	0.621514322	MGI:1345283	Slc25a1
ENSMUST00000025083.13	0.621514322	MGI:1098268	Kif5b
ENSMUST00000025490.9	0.621514322	MGI:1916106	Prrc1
ENSMUST00000028852.12	0.621514322	MGI:1924971	Mrps5
ENSMUST00000048121.12	0.621514322	MGI:2384407	Myrip
ENSMUST00000051256.9	0.621514322	MGI:1925498	Armcx1
ENSMUST00000053819.5	0.621514322	MGI:1890577	Srsf4
ENSMUST00000078752.9	0.621514322	MGI:2443129	Casc4
ENSMUST00000100487.5	0.621514322	MGI:1353448	Eif2ak1
ENSMUST00000144458.7	0.621514322	MGI:894652	Usp10
ENSMUST00000159759.2	0.621514322	MGI:2147598	AI837181
ENSMUST00000161859.7	0.621514322	MGI:1921382	Rnf6
ENSMUST00000169172.4	0.621514322	MGI:2445052	Tbck
ENSMUST00000177962.8	0.621514322	MGI:101917	Bcap29
ENSMUST00000028117.3	0.622081587	MGI:1351651	Yme1l1
ENSMUST00000016138.10	0.624507747	MGI:104683	Fnta
ENSMUST00000076674.3	0.624507747	MGI:1933206	Grin3a
ENSMUST00000099087.7	0.624507747	MGI:1928482	Mbnl1
ENSMUST00000108122.7	0.624507747	MGI:2442298	Lingo2
ENSMUST00000113245.8	0.624507747	MGI:1929475	Irak1bp1
ENSMUST00000196817.4	0.624507747	MGI:106028	Rhoc
ENSMUST00000198878.1	0.624507747	MGI:1915401	Wls
ENSMUST00000033489.7	0.62486502	MGI:1859607	Praf2
ENSMUST00000008907.13	0.626917462	MGI:104676	Man1a2
ENSMUST00000016125.11	0.626917462	MGI:2385336	Stk32c
ENSMUST00000033761.12	0.628899036	MGI:105942	Hcfc1
ENSMUST00000094051.5	0.628899036	MGI:3646999	Gm7324
ENSMUST00000094312.11	0.628899036	MGI:2679262	Rgma
ENSMUST00000186469.6	0.628899036	MGI:1345629	Rhof
ENSMUST00000107505.7	0.630223339	MGI:1859547	Syt11
ENSMUST00000003529.8	0.630557263	MGI:1923988	Paf1
ENSMUST00000039373.13	0.630557263	MGI:1913894	Uba6
ENSMUST00000040668.8	0.630557263	MGI:2442832	Osbpl2
ENSMUST00000109574.7	0.630557263	MGI:103010	Epb41l1
ENSMUST00000122302.7	0.630557263	MGI:98353	Son
ENSMUST00000055261.10	0.631965319	MGI:2442631	Mob1a

ENSMUST00000064196.4	0.631965319	MGI:2147918	B630019K06Rik
ENSMUST00000067512.7	0.631965319	MGI:1927578	Smpd3
ENSMUST00000064922.6	0.633175847	MGI:96647	Junb
ENSMUST00000029722.6	0.634227688	MGI:1202063	Rps3a1
ENSMUST00000062740.14	0.635150126	MGI:87881	Acp1
ENSMUST00000028105.12	0.635965657	MGI:1914210	Mindy3
ENSMUST00000141118.8	0.635965657	MGI:87989	Alas1
ENSMUST00000097522.10	0.637342619	MGI:1924237	Hdhd2
ENSMUST00000078332.12	0.638460472	MGI:1922984	Mff
ENSMUST00000031530.8	0.638944061	MGI:1891433	Sppl3
ENSMUST00000021090.13	0.642130858	MGI:95805	Grb2
ENSMUST00000082429.6	0.642130858	MGI:104887	Gpx1
ENSMUST00000029635.13	0.642913367	MGI:1860604	Gucy1b1
ENSMUST00000021497.15	0.64419671	MGI:1933947	Rtn1
ENSMUST00000151786.7	0.656111882	MGI:1914051	Prkrip1
ENSMUST00000192538.1	0.656310082	MGI:3642386	Gm9774
ENSMUST00000093962.4	0.662014955	MGI:88313	Ccnd1
ENSMUST00000042497.13	0.671108281	MGI:107851	Ndufv1
ENSMUST00000153983.7	0.673242699	MGI:1915822	Mrpl58
ENSMUST00000120128.7	0.674474045	MGI:2441950	Adgrl3
ENSMUST00000147196.2	0.675838468	MGI:1915101	Mtres1
ENSMUST00000089759.8	0.679008431	MGI:1919161	Bdh1
ENSMUST00000024047.11	0.679063298	MGI:1346078	Twf2
ENSMUST00000043937.8	0.680545536	MGI:1913607	Ostc
ENSMUST00000109604.8	0.680987683	MGI:1922960	Rbm12
ENSMUST00000032138.14	0.681475294	MGI:88431	Cnbp
ENSMUST00000000756.5	0.683171993	MGI:105922	Rpl13
ENSMUST00000087204.8	0.685691398	MGI:2443895	Fry
ENSMUST00000168536.8	0.685691398	MGI:2178563	Nt5c2
ENSMUST00000040914.2	0.686283194	MGI:1931526	H1f2
ENSMUST00000027991.11	0.687450289	MGI:108409	Rgs4
ENSMUST00000032809.9	0.688607471	MGI:1924504	Yif1b
ENSMUST00000040383.8	0.688607471	MGI:2384831	Cc2d1a
ENSMUST00000065103.3	0.688607471	MGI:1913473	Mrpl35
ENSMUST00000140485.7	0.688607471	MGI:1924550	Raph1
ENSMUST00000041551.8	0.688693289	MGI:1914189	Aagab
ENSMUST00000190686.6	0.690019848	MGI:1923206	Srrm2
ENSMUST00000037349.7	0.691438684	MGI:1349419	Aifm1
ENSMUST00000022696.7	0.69203047	MGI:1929705	Xpo7

ENSMUST00000049263.8	0.69203047	MGI:1913910	Sltm
ENSMUST00000097715.3	0.69203047	MGI:2137229	Mrpl43
ENSMUST00000166082.7	0.69203047	MGI:1915288	Chid1
ENSMUST00000033139.14	0.696105178	MGI:1333870	Ate1
ENSMUST00000071739.11	0.696105178	MGI:2152453	Gsk3a
ENSMUST00000103018.10	0.696105178	MGI:1919305	Slc38a10
ENSMUST00000103201.7	0.696105178	MGI:108451	Acaca
ENSMUST00000170672.8	0.696105178	MGI:1919805	Shisa9
ENSMUST00000038757.7	0.696356377	MGI:2146027	Csdc2
ENSMUST00000034046.12	0.698260529	MGI:102797	Acsl1
ENSMUST00000042493.9	0.698260529	MGI:1916918	Ccdc115
ENSMUST00000022980.4	0.700325029	MGI:1913468	Ndufb9
ENSMUST00000032451.8	0.701037318	MGI:95630	Slc6a11
ENSMUST00000047479.2	0.701037318	MGI:1891443	Pcdhac2
ENSMUST00000106882.8	0.701037318	MGI:1920344	Sgip1
ENSMUST00000109331.8	0.701037318	MGI:2154239	Plxnb2
ENSMUST00000124992.7	0.701037318	MGI:1926245	Ube2j1
ENSMUST00000028892.10	0.701523185	MGI:2158650	Idh3b
ENSMUST00000023759.5	0.707129395	MGI:1933623	Smarcd1
ENSMUST00000031391.8	0.707129395	MGI:1924295	Bcl7a
ENSMUST00000036884.2	0.707129395	MGI:1333856	Klf9
ENSMUST00000049811.7	0.707129395	MGI:2147298	Cep120
ENSMUST00000065496.11	0.707129395	MGI:2445175	Arhgap20
ENSMUST00000071592.11	0.707129395	MGI:2384879	Prmt7
ENSMUST00000073089.12	0.707129395	MGI:1924567	Miga1
ENSMUST00000080751.8	0.707129395	MGI:1919551	Shisal1
ENSMUST00000081851.3	0.707129395	MGI:1861735	Tmeff2
ENSMUST00000165684.7	0.707129395	MGI:2158394	Med16
ENSMUST00000210780.1	0.707129395	MGI:1330284	Slc16a7
ENSMUST00000102659.1	0.707712486	MGI:1916262	Sestd1
ENSMUST00000108081.8	0.71067373	MGI:2387356	Ggnbp2
ENSMUST00000067646.11	0.713950869	MGI:1339973	Ilf3
ENSMUST00000034164.5	0.714845206	MGI:1919205	Ist1
ENSMUST00000046022.15	0.714845206	MGI:1099835	Skiv2l
ENSMUST00000111432.9	0.714845206	MGI:1344382	Creg1
ENSMUST00000115096.3	0.714845206	MGI:2179061	Plxna4
ENSMUST00000131446.7	0.714845206	MGI:1924182	Arfip2
ENSMUST00000198965.4	0.714845206	MGI:106484	Rufy3
ENSMUST00000005583.11	0.714845206	MGI:108414	Pafah1b3

ENSMUST00000015855.7	0.714845206	MGI:1925152	Prune1
ENSMUST00000033127.11	0.714845206	MGI:1351630	Sergef
ENSMUST00000040421.10	0.714845206	MGI:1098643	Coq5
ENSMUST00000043990.13	0.714845206	MGI:2142951	Edc3
ENSMUST00000051937.8	0.714845206	MGI:1916189	Rasl11b
ENSMUST00000061334.9	0.714845206	MGI:2444136	Mars2
ENSMUST00000072751.12	0.714845206	MGI:1915964	Dohh
ENSMUST00000085684.10	0.714845206	MGI:1923038	Smurf1
ENSMUST00000101499.9	0.714845206	MGI:2144842	Cbll1
ENSMUST00000105230.3	0.714845206	MGI:1913368	Sarnp
ENSMUST00000105686.2	0.714845206	MGI:1917806	Slc25a33
ENSMUST00000107168.7	0.714845206	MGI:1328354	Mpp3
ENSMUST00000107456.3	0.714845206	MGI:3698178	Fam174b
ENSMUST00000114102.9	0.714845206	MGI:1915362	Entr1
ENSMUST00000193117.2	0.714845206	MGI:1924147	Ralyl
ENSMUST00000203187.2	0.714845206	MGI:1921991	C2cd5
ENSMUST00000004565.14	0.714845206	MGI:1927244	Ralb
ENSMUST00000010049.5	0.714845206	MGI:1918000	Kdsr
ENSMUST00000020551.12	0.714845206	MGI:1929749	Asb3
ENSMUST00000027688.14	0.714845206	MGI:1926375	Rassf5
ENSMUST00000033973.13	0.714845206	MGI:2681000	Rwdd4a
ENSMUST00000036807.12	0.714845206	MGI:1921004	Thap1
ENSMUST00000054471.9	0.714845206	MGI:1921197	4930430F08Rik
ENSMUST00000067167.5	0.714845206	MGI:1917817	Fra10ac1
ENSMUST00000091636.4	0.714845206	MGI:2389174	Lrrtm2
ENSMUST00000111289.7	0.714845206	MGI:1350916	Nit1
ENSMUST00000114846.8	0.714845206	MGI:2181202	Pfkfb3
ENSMUST00000180180.7	0.714845206	MGI:1349469	Ecsit
ENSMUST00000182625.1	0.714845206	MGI:98360	Sox12
ENSMUST00000119197.7	0.717597309	MGI:108117	Emd
ENSMUST00000108409.1	0.718335022	MGI:3607779	Tmem145
ENSMUST00000026328.10	0.721679217	MGI:1859815	Prdx4
ENSMUST00000082373.7	0.721679217	MGI:1889615	Luzp2
ENSMUST00000035560.8	0.723652068	MGI:1919732	Acbd6
ENSMUST00000032998.12	0.725362689	MGI:1350917	Rps3
ENSMUST00000200109.4	0.729855662	MGI:2444812	Camkk2
ENSMUST00000033131.11	0.731503874	MGI:1913758	Lamtor1
ENSMUST00000142407.7	0.731503874	MGI:1858216	Ube2k
ENSMUST00000179619.8	0.73336224	MGI:1340051	Adgrg1

ENSMUST00000022504.11	0.73748834	MGI:1346861	Mapk8
ENSMUST00000081775.11	0.740054972	MGI:99421	Nfe2l1
ENSMUST00000165532.2	0.740196849	MGI:1914365	Rpl14
ENSMUST00000053063.6	0.74137788	MGI:2385923	Hexim1
ENSMUST00000064989.11	0.743271651	MGI:97596	Prkcb
ENSMUST00000030464.13	0.744411963	MGI:109277	Pik3r3
ENSMUST00000041022.14	0.744411963	MGI:2673000	Trim46
ENSMUST00000036049.5	0.745987292	MGI:1919802	Hsdl1
ENSMUST00000026538.12	0.751082295	MGI:2136460	Echs1
ENSMUST00000049488.8	0.751082295	MGI:2444223	Serinc5
ENSMUST00000103181.10	0.751082295	MGI:1332236	Cds2
ENSMUST00000026289.9	0.752514746	MGI:1333871	Hsd17b10
ENSMUST00000034547.5	0.754634082	MGI:87870	Acat1
ENSMUST00000090243.7	0.756743928	MGI:1919394	Slc37a3
ENSMUST00000078034.4	0.762118689	MGI:3642682	Rpl9-ps6
ENSMUST00000015391.9	0.762126131	MGI:1913786	Nipsnap3b
ENSMUST00000017629.11	0.762126131	MGI:98791	Top2b
ENSMUST00000027534.12	0.762126131	MGI:1914694	Ilkap
ENSMUST00000038217.13	0.762126131	MGI:2135752	Dtx3
ENSMUST00000040504.11	0.762126131	MGI:2442829	Klhl4
ENSMUST00000056888.12	0.762126131	MGI:1915673	Ankrd13d
ENSMUST00000090682.3	0.762126131	MGI:1914386	Kbtbd4
ENSMUST00000109468.2	0.762126131	MGI:98788	Top1
ENSMUST00000115628.9	0.762126131	MGI:101825	Tango2
ENSMUST00000036376.12	0.763072333	MGI:1919303	Tmub2
ENSMUST00000023405.15	0.763762249	MGI:1927136	Arl6
ENSMUST00000096229.10	0.764435913	MGI:1921256	Dnm1l
ENSMUST00000022629.8	0.770192744	MGI:1349763	Dpysl2
ENSMUST00000102981.9	0.770192744	MGI:1931071	Sec61a2
ENSMUST00000115436.8	0.770192744	MGI:1309489	Cask
ENSMUST00000025711.12	0.773710966	MGI:1915755	Vps51
ENSMUST00000029804.12	0.773710966	MGI:1922874	Metap1
ENSMUST00000031002.9	0.773710966	MGI:1195262	Man2b2
ENSMUST00000057692.10	0.773710966	MGI:2183260	Luc7l2
ENSMUST00000068999.13	0.773710966	MGI:1925756	Micu3
ENSMUST00000090275.4	0.773710966	MGI:1334209	Gjd2
ENSMUST00000023455.13	0.778263868	MGI:2447857	Ppil2
ENSMUST00000030501.14	0.778263868	MGI:1916322	Ebna1bp2
ENSMUST00000068755.13	0.780457078	MGI:1917487	Bhlhb9

ENSMUST00000102594.10	0.780457078	MGI:1915079	1110051M20Rik
ENSMUST00000055647.14	0.783199576	MGI:108426	Kif1b
ENSMUST00000151120.8	0.787489894	MGI:88562	Ctsd
ENSMUST00000149565.7	0.787947104	MGI:1196345	Agpat5
ENSMUST00000160899.7	0.787947104	MGI:1924148	B3gat1
ENSMUST00000163483.1	0.791887939	MGI:97842	Rab1a
ENSMUST00000094280.3	0.796699424	MGI:1261428	Chchd2
ENSMUST00000042096.14	0.803310868	MGI:2443696	Emc1
ENSMUST00000187810.1	0.803310868	MGI:2445172	Arl4c
ENSMUST00000022007.7	0.805863329	MGI:1916565	1700001L19Rik
ENSMUST00000040873.11	0.805863329	MGI:1933527	Srrt
ENSMUST00000048937.5	0.805863329	MGI:2685031	Leo1
ENSMUST00000090678.10	0.805863329	MGI:97852	Rap1a
ENSMUST00000099078.9	0.805863329	MGI:2139354	Arfgef2
ENSMUST00000238932.1	0.805863329	MGI:108090	Ei24
ENSMUST00000004622.6	0.805863329	MGI:1333854	Gab2
ENSMUST00000019183.13	0.805863329	MGI:1915039	Dalrd3
ENSMUST00000021380.9	0.805863329	MGI:1925482	Trappc6b
ENSMUST00000030724.8	0.805863329	MGI:2651874	Sesn2
ENSMUST00000050063.8	0.805863329	MGI:99435	Arf6
ENSMUST00000068140.5	0.805863329	MGI:1919559	Tmem158
ENSMUST00000072857.12	0.805863329	MGI:2443446	Scfd2
ENSMUST00000094695.11	0.805863329	MGI:1915059	Rmdn3
ENSMUST00000109728.7	0.805863329	MGI:101772	Snta1
ENSMUST00000167140.7	0.805863329	MGI:2179733	Mpst
ENSMUST00000168727.2	0.805863329	MGI:95684	Gdf10
ENSMUST00000203633.2	0.805863329	MGI:5753809	Etfbl
ENSMUST00000032191.15	0.805863329	MGI:1889844	Sumf1
ENSMUST00000033289.5	0.805863329	MGI:107476	Stim1
ENSMUST00000037399.15	0.805863329	MGI:2385271	Blvrb
ENSMUST00000041126.8	0.805863329	MGI:2444061	Ss18l1
ENSMUST00000046461.8	0.805863329	MGI:2148865	Dok4
ENSMUST00000057608.4	0.805863329	MGI:2447899	Lrrc3
ENSMUST00000059438.10	0.805863329	MGI:1915527	2310057M21Rik
ENSMUST00000061850.4	0.805863329	MGI:1921903	Pomk
ENSMUST00000068457.14	0.805863329	MGI:1917029	Pphln1
ENSMUST00000115658.5	0.805863329	MGI:1298372	Pcdha11
ENSMUST00000115658.5	0.805863329	MGI:1298372	Pcdha11
ENSMUST00000115658.5	0.805863329	MGI:1298372	Pcdha11

ENSMUST00000115658.5	0.805863329	MGI:1298372	Pcdha11
ENSMUST00000120943.7	0.805863329	MGI:1891471	Spsb3
ENSMUST00000027727.14	0.810564953	MGI:1919924	Adipor1
ENSMUST00000159062.7	0.810564953	MGI:1261912	Fbxo41
ENSMUST00000193414.1	0.810564953	MGI:1935217	Pcdhga5
ENSMUST00000128764.8	0.812808662	MGI:1277978	Sap18
ENSMUST00000057625.2	0.813683709	MGI:1923469	Arxes1
ENSMUST00000027256.11	0.816400482	MGI:1333820	Mrpl30
ENSMUST00000159626.7	0.822595542	MGI:88275	Ctnna2
ENSMUST00000212864.1	0.822595542	MGI:3708786	Gm10358
ENSMUST00000051416.11	0.823555758	MGI:2153181	Hnmt
ENSMUST00000046565.12	0.825257565	MGI:1097716	Arx
ENSMUST00000116584.1	0.825257565	MGI:1351620	Mrpl39
ENSMUST00000163134.7	0.825257565	MGI:1096389	Nrxn3
ENSMUST00000100314.3	0.835816366	MGI:1913101	Cldn10
ENSMUST00000192227.5	0.835816366	MGI:1346089	Rgs7
ENSMUST00000027921.10	0.842898682	MGI:1919586	Iars2
ENSMUST00000113711.2	0.842898682	MGI:1919070	Wdr34
ENSMUST00000131552.4	0.842898682	MGI:97613	Plcb1
ENSMUST00000132855.7	0.842898682	MGI:3044681	Wipf3
ENSMUST00000142129.7	0.842898682	MGI:107434	Cdh8
ENSMUST00000032732.14	0.849934983	MGI:1261791	Apba2
ENSMUST00000033873.8	0.850348109	MGI:2387215	Erlin2
ENSMUST00000109075.7	0.864476675	MGI:1926424	Nelfcd
ENSMUST00000056149.14	0.866369445	MGI:1923442	Abhd12
ENSMUST00000058210.12	0.867468929	MGI:104684	Eps8
ENSMUST00000016143.8	0.880569889	MGI:2658986	Wasf3
ENSMUST00000025270.7	0.885741841	MGI:1914128	Riok3
ENSMUST00000087511.9	0.885741841	MGI:1913559	Tmem128
ENSMUST00000022038.14	0.888058393	MGI:1925939	Naa35
ENSMUST00000092678.9	0.890730723	MGI:1917580	Bclaf1
ENSMUST00000003911.12	0.891478401	MGI:105126	Rad23a
ENSMUST00000005620.9	0.891478401	MGI:1931874	Dnajb1
ENSMUST00000015239.9	0.891478401	MGI:1354731	Fbxw5
ENSMUST00000026927.9	0.891478401	MGI:1858232	Nudt5
ENSMUST00000029771.12	0.891478401	MGI:88381	F3
ENSMUST00000034226.7	0.891478401	MGI:1919637	Fam192a
ENSMUST00000057907.9	0.891478401	MGI:1354744	Fbxo44
ENSMUST00000092891.5	0.891478401	MGI:1932374	Cacng7

ENSMUST00000171155.3	0.891478401	MGI:2668031	Klhl25
ENSMUST00000002259.12	0.891478401	MGI:107472	Clgn
ENSMUST00000015725.15	0.891478401	MGI:109328	Bnip1
ENSMUST00000023535.3	0.891478401	MGI:2443764	Iqcb1
ENSMUST00000040241.14	0.891478401	MGI:2148251	Ddx19b
ENSMUST00000041208.8	0.891478401	MGI:2443767	Aaas
ENSMUST00000044155.14	0.891478401	MGI:1920078	Ubash3b
ENSMUST00000044825.4	0.891478401	MGI:1916737	Ndufaf5
ENSMUST00000066148.11	0.891478401	MGI:1925543	Tbcel
ENSMUST00000091459.11	0.891478401	MGI:1915925	Fam172a
ENSMUST00000093253.9	0.891478401	MGI:2445069	Ccdc85a
ENSMUST00000111913.8	0.891478401	MGI:2385108	Odr4
ENSMUST00000139049.1	0.891478401	MGI:3779453	Prps1l3
ENSMUST00000190997.6	0.891478401	MGI:1918367	Rbbp5
ENSMUST00000022030.10	0.910647128	MGI:1913921	Ccnh
ENSMUST00000042594.12	0.910978262	MGI:2157910	Mlc1
ENSMUST00000061437.4	0.923765209	MGI:1929289	Adrm1
ENSMUST00000030606.13	0.935491182	MGI:1858220	Rcan3
ENSMUST00000121646.7	0.943792048	MGI:1100874	Plagl1
ENSMUST00000063882.11	0.94826301	MGI:2140972	Dcun1d4
ENSMUST00000020896.16	0.951477965	MGI:1913359	Tspan13
ENSMUST00000214685.1	0.971438081	MGI:98373	Sparc
ENSMUST00000084941.11	0.971511669	MGI:107978	Klc1
ENSMUST00000025050.12	0.971781422	MGI:1928484	Nudt3
ENSMUST00000032887.3	0.971781422	MGI:107207	Coq7
ENSMUST00000079510.5	0.971892466	MGI:106417	Cherp
ENSMUST00000116380.8	0.971892466	MGI:98085	Rpn2
ENSMUST00000028592.11	0.97229611	MGI:1351744	Eif3m
ENSMUST00000031805.10	0.97229611	MGI:1926187	Avl9
ENSMUST00000026990.5	0.97229611	MGI:1920916	Thoc3
ENSMUST00000027357.11	0.97229611	MGI:1890215	Rnf25
ENSMUST00000041459.8	0.97229611	MGI:1929280	Cyb561d2
ENSMUST00000043050.8	0.97229611	MGI:1929064	Chst12
ENSMUST00000058393.8	0.97229611	MGI:2140680	A430005L14Rik
ENSMUST00000062962.11	0.97229611	MGI:1922169	Slc35f6
ENSMUST00000148960.7	0.97229611	MGI:1914518	Myl12a
ENSMUST00000002925.5	0.97229611	MGI:1922139	Timmdc1
ENSMUST00000030842.7	0.97229611	MGI:1916401	Lzic
ENSMUST00000049281.11	0.97229611	MGI:1913556	Fam53c

ENSMUST00000064272.9	0.97229611	MGI:1928767	B4galt3
ENSMUST00000183952.1	0.97229611	MGI:1917634	Tmem185b
ENSMUST00000034147.3	0.99959654	MGI:1353574	Zfp330
ENSMUST00000029968.13	1.006338039	MGI:1923596	Rars2
ENSMUST00000074575.10	1.012805601	MGI:98341	Snrnp70
ENSMUST00000038874.11	1.02189402	MGI:2443716	Scai
ENSMUST00000057885.12	1.038255422	MGI:1298373	Rpl9
ENSMUST00000007005.13	1.039024572	MGI:87871	Acat2
ENSMUST00000020284.4	1.048825681	MGI:1919017	Tysnd1
ENSMUST00000028915.5	1.048825681	MGI:1347074	Rbbp9
ENSMUST00000028928.7	1.048825681	MGI:1921783	Gzf1
ENSMUST00000100404.5	1.048825681	MGI:2685903	B3glct
ENSMUST00000116279.9	1.048825681	MGI:1924074	Mtfr1l
ENSMUST00000020949.11	1.048825681	MGI:1346870	Map2k6
ENSMUST00000058787.8	1.048825681	MGI:95748	Glra2
ENSMUST00000183309.7	1.048825681	MGI:1914489	Rpf2
ENSMUST00000093290.11	1.050464251	MGI:1914495	Peli1
ENSMUST00000006669.5	1.053322619	MGI:1926119	Pdk1
ENSMUST00000114086.7	1.055790457	MGI:1935151	Klf7
ENSMUST00000039449.8	1.068160839	MGI:1926163	Ltn1
ENSMUST00000020234.13	1.070220492	MGI:98754	Timp3
ENSMUST00000040519.11	1.072358991	MGI:1915270	Apopt1
ENSMUST00000025541.5	1.084228065	MGI:95776	Gnaq
ENSMUST00000015100.14	1.087190724	MGI:104871	Ppp1cb
ENSMUST00000070733.8	1.090585552	MGI:107418	Ptprn2
ENSMUST00000041331.3	1.098071539	MGI:98239	Scd1
ENSMUST00000036561.7	1.121499329	MGI:1914811	Wdr48
ENSMUST00000008579.13	1.121499329	MGI:1918732	Rdh13
ENSMUST00000047749.6	1.121499329	MGI:1913964	4921524J17Rik
ENSMUST00000044705.11	1.121499329	MGI:1914525	Vstm2b
ENSMUST00000105111.3	1.121499329	MGI:95474	F8a
ENSMUST00000161807.7	1.121499329	MGI:1096365	Psme2
ENSMUST00000059018.13	1.132200685	MGI:1354708	Fbxo31
ENSMUST00000029915.5	1.135490426	MGI:1924282	Tstd3
ENSMUST00000025875.4	1.139165632	MGI:105083	Slc1a1
ENSMUST00000116621.1	1.143037034	MGI:1349919	Ndufb11
ENSMUST00000076605.8	1.148479188	MGI:95774	Gnal
ENSMUST00000017945.14	1.190687045	MGI:108398	Mlx
ENSMUST00000077977.13	1.190687045	MGI:1922035	Miga2

ENSMUST00000089049.3	1.190687045	MGI:2385853	Nudt18
ENSMUST00000100220.4	1.190687045	MGI:106544	Spout1
ENSMUST00000109191.1	1.190687045	MGI:3616086	Kcng1
ENSMUST00000200680.3	1.190687045	MGI:1917036	Tprkb
ENSMUST00000020529.12	1.190687045	MGI:1916133	Ahsa2
ENSMUST00000027380.11	1.190687045	MGI:2442781	Tmem169
ENSMUST00000027601.10	1.190687045	MGI:1298227	Mcm6
ENSMUST00000032635.13	1.190687045	MGI:1913918	Nipa2
ENSMUST00000040416.7	1.190687045	MGI:99526	Ddx19a
ENSMUST00000084650.5	1.190687045	MGI:2685341	Gpr139
ENSMUST00000183404.7	1.190687045	MGI:101877	Tcf12
ENSMUST00000078803.4	1.208475635	MGI:1914164	Vps28
ENSMUST00000028137.9	1.256707997	MGI:1098826	Coq4
ENSMUST00000034060.6	1.256707997	MGI:1913066	Sh3rf1
ENSMUST00000037796.13	1.256707997	MGI:1916330	Gmppa
ENSMUST00000055322.5	1.256707997	MGI:1337072	Ier5
ENSMUST00000087614.10	1.256707997	MGI:1338038	Aebp2
ENSMUST00000102657.9	1.256707997	MGI:1351471	B9d1
ENSMUST00000114848.7	1.256707997	MGI:1916026	Taf11
ENSMUST00000027153.5	1.256707997	MGI:87866	Acadl
ENSMUST00000031311.9	1.256707997	MGI:102726	Dck
ENSMUST00000031845.12	1.256707997	MGI:1889850	Gsdme
ENSMUST00000108567.8	1.256707997	MGI:1923365	Zfp444
ENSMUST00000185675.6	1.256707997	MGI:2136405	Glce
ENSMUST00000041782.3	1.319839432	MGI:1353636	Abt1
ENSMUST00000055458.5	1.319839432	MGI:1929744	Cdc42ep2
ENSMUST00000069195.4	1.319839432	MGI:99192	Zfp46
ENSMUST00000025885.5	1.319839432	MGI:1913482	Znrd2
ENSMUST00000034539.11	1.319839432	MGI:1916555	Dcps
ENSMUST00000160439.7	1.319839432	MGI:1916068	Zfand2b
ENSMUST00000163048.7	1.319839432	MGI:1289274	Krr1
ENSMUST00000210564.2	1.319839432	MGI:1914314	Chmp1b
ENSMUST00000050148.4	1.339412642	MGI:1914322	Cdc37l1
ENSMUST00000061457.6	1.350241091	MGI:2386852	Csrnp2
ENSMUST00000025236.8	1.369579737	MGI:2156764	Stard4
ENSMUST00000020692.6	1.380323708	MGI:108384	Btg2
ENSMUST00000026988.10	1.380323708	MGI:1930788	Arl10
ENSMUST00000095517.11	1.380323708	MGI:2142885	Cog8
ENSMUST00000227920.1	1.380323708	MGI:1101353	Klf10

ENSMUST00000034560.13	1.380323708	MGI:1920949	Ppp2r1b
ENSMUST00000057942.3	1.380323708	MGI:99457	Mc4r
ENSMUST00000117513.7	1.380323708	MGI:1918529	Slc29a3
ENSMUST00000168503.7	1.380323708	MGI:1354743	Fbxo6
ENSMUST00000085984.10	1.438373915	MGI:1913388	Bud23
ENSMUST00000036003.7	1.438373915	MGI:2444786	Klhl42
ENSMUST00000054555.9	1.438373915	MGI:99186	Zfp41
ENSMUST00000109910.8	1.438373915	MGI:97307	Nf2
ENSMUST00000171031.7	1.438373915	MGI:88315	Ccnd3
ENSMUST00000015920.11	1.494178407	MGI:98446	Med22
ENSMUST00000033098.15	1.547904486	MGI:1276534	Bcat2
ENSMUST00000035983.11	1.547904486	MGI:1278340	Rpl21
ENSMUST00000084743.6	1.560440442	MGI:2143424	Pomgnt2
ENSMUST00000029158.3	1.599701427	MGI:1915545	Aar2
ENSMUST00000169282.7	1.599701427	MGI:1915568	Aph1c
ENSMUST00000177963.7	1.599701427	MGI:1918849	Senp8
ENSMUST00000037048.8	1.604212544	MGI:1922354	Mmd2
ENSMUST00000098242.3	1.649702982	MGI:1329035	Ggh
ENSMUST00000177567.7	1.649702982	MGI:1923507	Slc38a3
ENSMUST00000191251.6	1.649702982	MGI:1917625	Ica1l
ENSMUST00000030018.4	1.649702982	MGI:2149820	Nans
ENSMUST00000032480.13	1.649702982	MGI:107307	Ing4
ENSMUST00000102922.9	1.649702982	MGI:1097163	Pold2
ENSMUST00000103217.10	1.698029454	MGI:1927868	Pex14
ENSMUST00000105038.2	1.698029454	MGI:3781727	Gm3550
ENSMUST00000038830.9	1.698029454	MGI:1913456	1110059E24Rik
ENSMUST00000060108.6	1.698029454	MGI:1916405	1810030O07Rik
ENSMUST00000097961.8	1.698029454	MGI:2444031	Alg6
ENSMUST00000088248.12	1.744789448	MGI:1915862	Ube2c
ENSMUST00000072232.8	1.744789448	MGI:1355315	Tmod3
ENSMUST00000030434.4	1.790081337	MGI:95593	Fuca1
ENSMUST00000057208.12	1.790081337	MGI:1916777	Mrps9
ENSMUST00000124549.8	1.833994509	MGI:1916406	Comtd1
ENSMUST00000026976.11	1.833994509	MGI:1914854	Get4
ENSMUST00000090811.10	1.918003545	MGI:1921866	Scrn3
ENSMUST00000211939.1	1.918003545	MGI:109299	Polr2c
ENSMUST00000021204.3	1.95824208	MGI:109331	Nxn
